# Isolation, bonding and reactivity of a monomeric stibine oxide

**DOI:** 10.1038/s41557-023-01160-x

**Published:** 2023-03-23

**Authors:** John S. Wenger, Monica Weng, Graham N. George, Timothy C. Johnstone

**Affiliations:** 1 Department of Chemistry and Biochemistry, University of California Santa Cruz, Santa Cruz, CA USA; 2 Department of Geological Sciences, University of Saskatchewan, Saskatoon, Saskatchewan Canada; 3 Department of Chemistry, University of Saskatchewan, Saskatoon, Saskatchewan Canada

**Keywords:** Chemical bonding, Organometallic chemistry

## Abstract

In contrast to phosphine oxides and arsine oxides, which are common and exist as stable monomeric species featuring the corresponding pnictoryl functional group (Pn=O/Pn^+^–O^−^; Pn = P, As), stibine oxides are generally polymeric, and the properties of the unperturbed stiboryl group (Sb=O/Sb^+^–O^−^) remain unexplored. We now report the isolation of the monomeric stibine oxide, Dipp_3_SbO (where Dipp = 2,6-diisopropylphenyl). Spectroscopic, crystallographic and computational studies provide insight into the nature of the Sb=O/Sb^+^–O^−^ bond. Moreover, isolation of Dipp_3_SbO allows the chemistry of the stiboryl group to be explored. Here we show that Dipp_3_SbO can act as a Brønsted base, a hydrogen-bond acceptor and a transition-metal ligand, in addition engaging in 1,2-addition, O-for-F_2_ exchange and O-atom transfer. In all cases, the reactivity of Dipp_3_SbO differed from that of the lighter congeners Dipp_3_AsO and Dipp_3_PO.

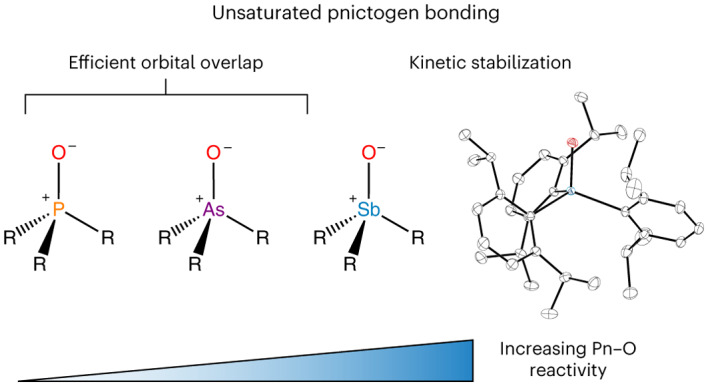

## Abstract (web summary)

In contrast to phosphine and arsine oxides, stibine oxides have been challenging to isolate in monomeric forms as they tend to polymerize. Now, such a SbO moiety has been kinetically stabilized using sterically bulky protecting groups, and its reactivity found to be substantially different to that of its lighter pnictogen counterparts.

## Main

**Fig. 1:**
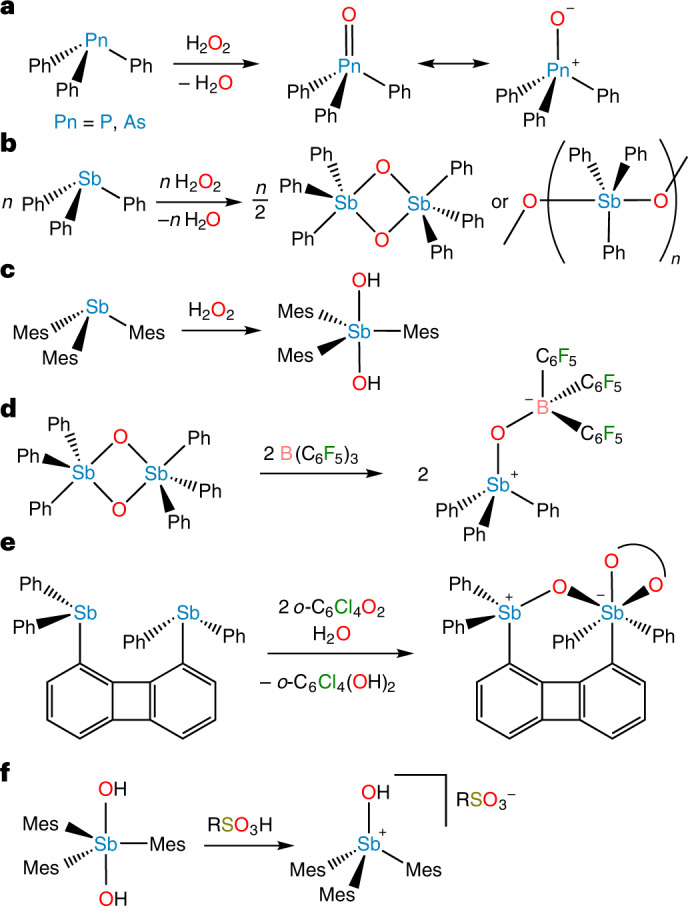
Synthesis of pnictine oxides. **a**, Oxidation of Ph_3_Pn yields monomeric Ph_3_PnO for Pn = P and As. **b**, Oxidation of Ph_3_Sb yields dimers or polymers. **c**, Oxidation of Mes_3_Sb yields *trans*-Sb(OH)_2_Mes_3_ (ref. ^22^). **d**, Lewis-acid-mediated disaggregation of (Ph_3_SbO)_2_ (ref. ^13^). **e**, Synthesis of an intramolecular stiborane-stabilized stibine oxide^14^. The chelating moiety (shown as a curved line) represents *o*-C_6_Cl_4_O_2_. **f**, Treatment of *trans*-Sb(OH)_2_Mes_3_ with sulfonic acids yields a hydroxystibonium salt and not a stibine oxide.

The stability of the pnictogen–oxygen bond in phosphine oxides has been used for over a century to drive chemical reactions such as those discovered by Wittig^1^, Mitsunobu^2^, Appel^3^ and Staudinger^4^. The electronic structure that gives rise to the stability of the P=O/P^+^–O^−^ bond was once a topic of intense debate, but the currently accepted model features a single covalent bond between the P and O atoms strengthened by electrostatic attraction between the P^+^ and O^−^ centres as well as donation from O-centred lone pairs into P–C antibonding orbitals^5^. As the Group 15 element increases in atomic number, however, the pnictogen valence orbitals become more diffuse, overlap with O-based orbitals decreases, and the pnictogen atom becomes increasingly able to expand its coordination sphere. These trends suggest that the heavier congeners of phosphine oxides could exhibit distinct and interesting reactivity^6^. The behaviour and properties of these heavier congeners would also provide a means of validating the bonding model currently used to describe the Pn=O/Pn^+^–O^−^ bond, where Pn is a pnictogen^5^. For As, the variations from P are small enough, possibly as a result of the scandide contraction^7^, that arsine oxides are generally analogous to phosphine oxides: they are monomeric species with As=O/As^+^–O^−^ polar covalent bonds. For example, oxidation of either Ph_3_P or Ph_3_As with H_2_O_2_ readily affords monomeric Ph_3_PnO (Pn = P, As; Fig. 1a). The situation changes substantially for Sb: no molecules containing an unperturbed Sb=O/Sb^+^–O^−^ bond have ever been isolated.

A substance described as triphenylstibine oxide was first reported in 1938^8^, and many other investigators subsequently purported to produce ‘Ph_3_SbO’ by treating Ph_3_Sb with H_2_O_2_ (ref. ^9^). Melting-point measurements and careful molecular-weight determinations showed these different substances to be dimeric or polymeric compounds and their structures were ultimately established with single-crystal X-ray diffraction and Sb extended X-ray absorption fine structure analysis (EXAFS; Fig. 1b)^10–12^.

Although Ph_3_SbO is not stable as a monomer, disaggregation of the polymer can be achieved with the Lewis acid B(C_6_F_5_)_3_ to afford the Lewis acid–base adduct Ph_3_SbOB(C_6_F_5_)_3_ (Fig. 1d)^13^. Another Lewis-acid-stabilized stibine oxide was obtained with a biphenylene-bridged system featuring a stibine oxide intramolecularly coordinated to a stiborane (Fig. 1e)^14^. A final example of Lewis-acid stabilization comprises [(3,5-F_2_C_6_H_3_)_4_SbOSbEt_3_][B(C_6_F_5_)_4_], which formed when a mixture of Et_3_Sb and [(3,5-F_2_C_6_H_3_)_4_Sb][B(C_6_F_5_)_4_] was exposed to oxygen^15^. In these compounds, interaction with a Lewis acid stabilizes the stibine oxide but also perturbs the Sb–O bonding interaction, preventing direct analysis of the periodic bonding trend across the pnictine oxides. These examples highlight that stibine oxides can be non-aggregated, but they raise the question of whether a monomeric stibine oxide is isolable in the absence of a Lewis acid interacting with and stabilizing the Sb=O/Sb^+^–O^−^ bond. Matrix isolation studies afford evidence for the existence of monomeric H_3_SbO (Sb–O stretching frequency (*ν*_SbO_) = 825 cm^−1^), but only in solid argon at 12 K (ref. ^16^).

We sought to explore a kinetic stabilization approach in which the reactive Sb=O/Sb^+^–O^−^ bond is protected by sterically bulky groups, a strategy that has been used with great success in the stabilization of other reactive main-group bonds^17–21^. The bulky mesityl groups of Mes_3_Sb prevent polymerization upon treatment with H_2_O_2_, but not coordination sphere expansion: the product is the stiborane *trans*-Sb(OH)_2_Mes_3_ (Fig. 1c)^22^. Our re-investigation of reports of Mes_3_SbO showed that the reported species is, in fact, a hydroxystibonium cation (Fig. 1f)^23,24^. This work similarly called into question the previously reported (2,6-(MeO)_2_Ph)_3_SbO (ref. ^25^).

## Results

### Synthesis and characterization

**Fig. 2:**
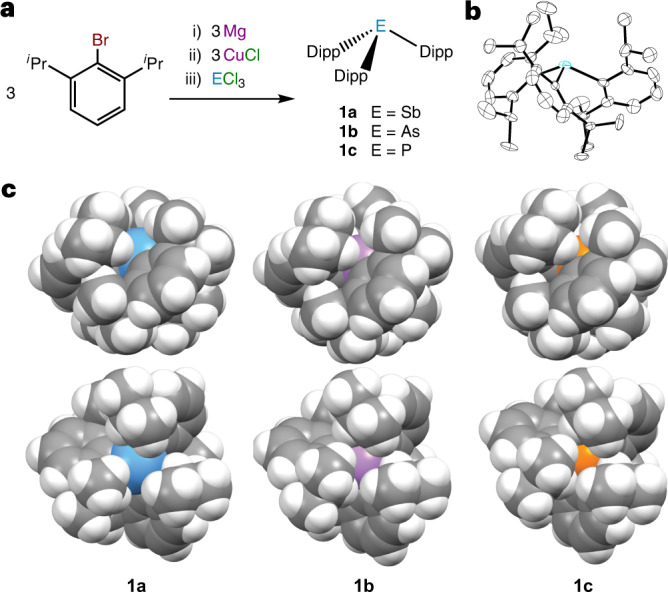
Synthesis of sterically crowded pnictines 1a–c. **a**, Synthesis of **1a**–**c**, Dipp = 2,6-diisopropylphenyl. **b**, Thermal ellipsoid diagram of **1a** at the 50% probability level, with H atoms omitted for clarity. **c**, Space-filling diagrams of **1a**–**c** from views rotated by 90° about the horizontal axis. Colour code: Sb, teal; As, purple; P, orange; C, grey; H, white.

We sought to prepare the even more sterically hindered stibine Dipp_3_Sb, **1a**, where Dipp = 2,6-diisopropylphenyl. Although many R_3_Sb species are readily accessed from SbCl_3_ and either RMgBr or RLi, these strategies do not afford **1a**. We therefore adapted a synthetic strategy developed by Sasaki and colleagues^26,27^, whereby the aryl group is installed on the Sb centre with an organocopper(I) species. In this way, **1a** was isolated as a colourless, crystalline, air-stable solid (Fig. 2a). Although the ^1^H NMR spectrum of **1a** indicates that rotation about the Sb–C and C_Ar_–C_iPr_ bonds is rapid on the NMR timescale at room temperature, the X-ray crystal structure highlights the extremely crowded environment around the Sb atom (Fig. 2b,c). The corresponding arsine (**1b**) and phosphine (**1c**) were similarly prepared (Fig. 2).

**Fig. 3:**
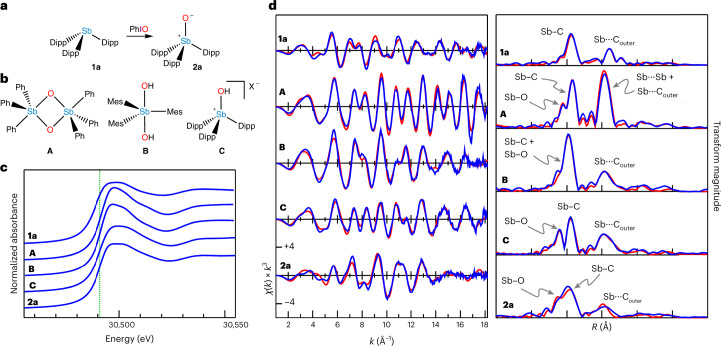
Oxidation of sterically crowded stibine 1a. **a**, Oxidation of **1a** with PhIO to give **2a**. **b**, Model compounds featuring different Sb–O bonding motifs. **A**, A dimeric stibine oxide; **B**, a dihydroxystiborane; **C**, a hydroxystibonium salt with X = O_3_SPh. **c**, Sb K-edge XAS spectra, with a green dotted line indicating where the derivative is maximal for **2a**. Full normalized Sb K-edge XAS spectra are provided in Supplementary Fig. 109. **d**, Sb K-edge EXAFS (left) and Sb–C phase-corrected Fourier transforms (right). Experimental data are shown in blue and fits in red. The relevant EXAFS parameters are provided in Supplementary Table 7. A breakdown of the contributions of the different scatterers to the EXAFS and Sb–C phase-corrected Fourier transforms of **A** and **2a** are shown in Supplementary Figs. 110 and 111, respectively.

Addition of **1a** to a suspension of PhIO in CH_2_Cl_2_ led to rapid consumption of the solid. Solvent was stripped from the reaction mixture and the residue was washed with pentane to yield a colourless solid, **2a** (Fig. 3a). The infrared spectrum of **2a** shows a new band at 779 cm^−1^, which we assign as a *ν*_SbO_ stretching frequency. This value is greater than any of the *ν*_SbO_ values of (Ph_3_SbO)_2_ (643/651 cm^−1^)^10^, *trans*-Sb(OH)_2_Mes_3_ (520 cm^−1^)^22,28^ or [Mes_3_SbOH][O_3_SPh] (612 cm^−1^)^23^. The ^1^H NMR spectrum of **2a** is distinct from that of **1a** and is consistent with a single Dipp environment with restricted rotation about the Sb–C bonds. Exchange spectroscopy (EXSY) and variable temperature (VT) NMR experiments confirmed the chemical exchange and reversible resonance coalescence (Supplementary Figs. 17 and 18).

The oxidation state of **2a** was probed with Sb X-ray absorption spectroscopy (XAS), which we have recently used to shed light on the structures of Sb-containing compounds^29^. The Sb K edge of **2a** is 2 eV higher in energy than that of **1a** (Fig. 3c). A similar shift was seen for a variety of Sb(V) compounds, including a dimeric stibine oxide (Ph_3_SbO)_2_ (**A**), a dihydroxystiborane *trans*-Sb(OH)_2_Mes_3_ (**B**) and a hydroxystibonium salt [Dipp_3_SbOH][O_3_SPh] (**C**, vide infra), indicating that **2a** also contains Sb(V) (Fig. 3c). The K-edge EXAFS data were collected to high resolution to gain further insight into the structure of **2a**. Similar data were collected from **1a**, **A**, **B** and **C** for comparison (Fig. 3d). The Fourier transform of the data from **A** shows a distinct Sb···Sb scattering at 3.148(3) Å (superimposed on an outer-shell carbon backscattering), which is absent for **B** and **C** as well as **2a**, indicating that **2a** does not feature a dioxadistibetane. A detailed fit of the data from **B** shows two O scatters at 2.128(3) Å, whereas **C** is better fit by a single O scatterer at 1.905(1) Å. These values are in excellent agreement with the crystallographically determined structures of these compounds. In contrast, the data from **2a** are best fit with a single O-atom scatterer at a distance of 1.837(2) Å, which is substantially shorter than the Sb–O bonds characterized for any other isolated materials. Fit of a C atom in the place of the short Sb–O gave a worse goodness-of-fit index (*F* = 0.335 for Sb–C versus 0.319 for Sb–O) and a physically unreasonable Debye–Waller factor (*σ*^2^ = 0.0010 Å^2^ for Sb–C versus 0.0021 Å^2^ for Sb–O).

**Fig. 4:**
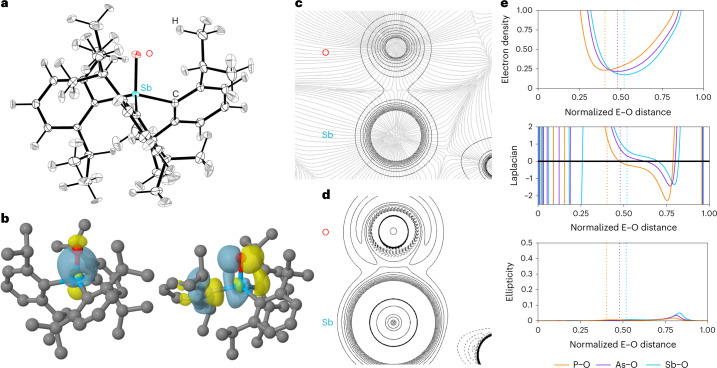
The structure and bonding of 2a, a monomeric stibine oxide. **a**, Thermal ellipsoid plot of **2a** at the 50% probability level. Colour code: Sb, teal; O, red; C, black; H, grey. **b**, Surface plots (isovalue = 0.05) depicting the Sb–O bonding NLMO (left) and overlap of O lone pair and Sb–C antibonding pre-orthogonalized NLMOs (right). Colour code: Sb, teal; O, red; C, grey; H, white. **c**, Contour plot of *ρ* overlaid with the gradient field lines of *ρ* for the Sb–O bond. **d**, Contour plot of ∇^2^*ρ* for the Sb–O bond, with positive values contoured with solid lines and negative values with dashed lines. **e**, Values of *ρ* (e^−^ Å^−3^), ∇^2^*ρ* (e^−^ Å^−5^) and ellipticity *ε* for **2a**–**c** along the Pn–O bond paths, with Pn at the left and O at the right along the horizontal axis. The bond length is normalized to 1.00. The location of the (3, −1) critical point is shown with a dashed vertical line. Source data

Ultimately, we were successful in growing diffraction-quality single crystals of **2a**. The asymmetric unit of the crystal structure features a single molecule of Dipp_3_SbO, which we unambiguously assign as the identity of **2a** (Fig. 4a). Hirshfeld atom refinement (HAR) afforded a Sb–O bond length of 1.8372(5) Å, which is in excellent agreement with the EXAFS distance. The next-nearest Sb···O distance is 9.0791(4) Å; space-filling diagrams highlight the steric shielding provided by the Dipp groups (Supplementary Fig. 99). One of the ^*i*^Pr C–H units is directed at the stiboryl O atom with an O···H distance of 2.132(9) Å (note: HAR affords freely refined H-atom positions similar to those given by neutron diffraction)^30^. The C–H···O bond angle of 148.1(8)° suggests a strong electrostatic contribution to the interaction relative to weaker C–H···O H-bonds, in which isotropic van der Waals forces play a larger role^31^.

The molecular geometry of **2a** from our X-ray crystal structure is in excellent agreement with the one from a theoretical geometry optimization (PBE0/def2-TZVPP), which features an Sb–O bond length of 1.827 Å. Notably, the scaled theoretical *ν*_SbO_ of 781 cm^−1^ at the gas-phase optimized geometry is in excellent agreement with the experimental value for **2a** (779 cm^−1^). These results combine to allow us to conclude that we have isolated an example of a monomeric stibine oxide. For comparison, we similarly synthesized and characterized the lighter congeners Dipp_3_AsO (**2b**) and Dipp_3_PO (**2c**).

### Electronic structure

To gain insight into the nature of the Sb=O/Sb^+^–O^−^ bonding motif, we analysed the topology of the theoretical electron density of **2a** (DKH-PBE0/old-DKH-TZVPP) (Fig. 4c–e). This analysis shows the locations of critical points in the electron density (*ρ*), that is, points in space where the derivative of *ρ* is zero in three mutually orthogonal directions. These critical points are characterized with a pair of numbers (*ω*, *σ*), where *ω* is the number of non-zero eigenvalues of the Hessian and *σ* is the sum of the signs of those eigenvalues^32^. As expected, a (3, −3) critical point is present near the nuclear position of each atom. We also identified (3, −1) critical points, also known as bond critical points, between each of the covalently bonded atoms (Supplementary Fig. 76). Although the values of various real-space functions at a (3, −1) critical point are frequently used to describe the nature of that bonding interaction^32^, for polar covalent bonds, like the Sb^+^–O^−^ bond in a stibine oxide, these functions are more informative when evaluated along the length of the bond path (Fig. 4e)^33^. For the Sb–O bond of **2a**, *ρ* features a single minimum at 0.173 e^−^Å^−3^, approximately halfway along the bond path. In the valence bonding region, the Laplacian (∇^2^*ρ*) exhibits a single O-proximal minimum of −1.346 e^−^Å^−5^. The ellipticity (*ε*) is negligible along the length of the bond path. The decrease in *ρ* and |∇^2^*ρ*| in the internuclear space from **2c** to **2b** to **2a** suggests a systematic weakening of the Pn^+^–O^−^ bond as the Group 15 element increases in atomic number. We note that the topological analysis also located bond paths connecting the pnictoryl O atoms and the ^*i*^Pr C–H units, as suggested by the crystallographic data. The analysis of the bond critical points of these interactions (Supplementary Tables 12 and 13) indicates that hydrogen-bonding interactions are present and that they decrease in magnitude from **2a** to **2b** to **2c**.

**Fig. 5:**
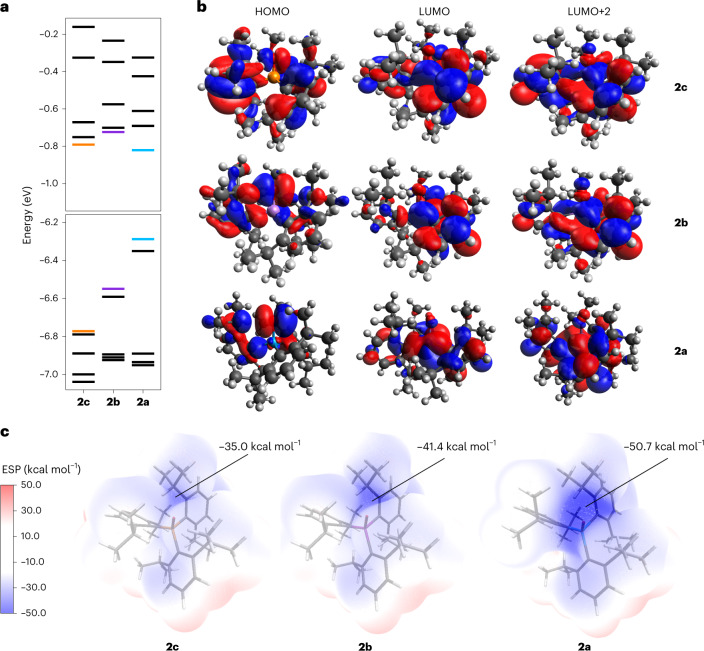
Variation in the electronic structure of the pnictine oxides 2a–c. **a**, Calculated orbital energies (DKH-PBE0/old-DKH-TZVPP//PBE0/def2-TZVPP) in electronvolts, with frontier molecular orbitals shown in colour (P, orange; As, purple; Sb, teal). **b**, Canonical molecular orbital diagrams of **2c**, **2b** and **2a**. Colour code surfaces: red, positive; blue, negative (isovalue = 0.02). **c**, Electrostatic surface potential (ESP) mapped van der Waals surfaces of **2c**–**a** (values in kcal mol^−1^). The value of the surface minimum is indicated. C, grey; H, white; O, red; P, orange; As, purple; Sb, teal. Source data

Further insight into the Sb–O bonding in **2a** was obtained from molecular orbital analyses. The canonical molecular orbitals (CMOs) are, as expected, highly delocalized across the molecule (Fig. 5b). The frontier CMOs feature substantial *π* or π* character from the Dipp substituents. The nearly degenerate highest occupied molecular orbital (HOMO) and HOMO–1 feature a substantial contribution from the lone pairs on the O atom. The lowest unoccupied molecular orbital (LUMO) features a substantial amount of Sb–O σ* character. The Dipp groups block the lobe of this orbital that extends opposite the Sb–O bond, which probably contributes to the stability of this molecule, as designed.

More detailed information was obtained by analysing the natural localized molecular orbitals (NLMOs) of **2a** (Fig. 4b and Supplementary Figs. 91–93). An Sb–O bonding NLMO is present and is polarized 74:25 toward the more electronegative O atom, which uses a hybrid atomic orbital enriched in *p* character (79%) to interact with the Sb. The Sb–O antibonding orbital is correspondingly polarized toward the Sb and exhibits the large lobe opposite the Sb–O bond that was observed in the LUMO CMO. There are two O-centred lone pair natural bond orbitals (NBOs) with nearly pure *p* character and a second-order perturbation theory analysis uncovered donor–acceptor interactions that delocalize electron density from these lone pairs into Sb–C antibonding orbitals (Supplementary Table 14). Similar delocalizations were observed for **2b** and **2c**, and deletion calculations showed that the non-covalent interactions between the O and Dipp_3_Pn fragments decreased from **2c** to **2b** to **2a**. These donor–acceptor interactions strengthen the Pn–O bonds, and the decreased delocalization in **2a** affords the lowest Wiberg Pn–O bond order of the three, but the O atom consequently retains the greatest natural atomic charge (Supplementary Table 15). The variation in charge accumulation is also reflected in the magnitude of the electrostatic surface potential minimum, for which **2c** < **2b** < **2a** (Fig. 5c). The decrease in Pn^+^–O^−^ bond strength (PO > AsO > SbO), is also reflected in the Pn^+^–O^−^ stretch force constants (Supplementary Fig. 75) and the ratio of Δ*E*_orb_/Δ*E*_total_ from an energy decomposition analysis of O and Dipp_3_Pn fragments (Supplementary Tables 8–10). Deformation density analyses show a redistribution of electron density from the Dipp_3_Pn fragment to the O atom to an extent that decreases from Sb to As to P (Supplementary Fig. 90).

Donor–acceptor interactions were also observed from the O-centred lone pairs to the ^*i*^Pr C–H antibonding orbitals for **2a**–**c** (Supplementary Fig. 93), consistent with the presence of the O···H bond paths noted above. Non-covalent interaction analysis of **2a** (Supplementary Fig. 89) uncovered a region with a negative product of *ρ* and the sign of the second-largest eigenvalue of the Hessian of *ρ*, sign(*λ*_2_)*ρ*, between the O and ^*i*^Pr C–H; the value of sign(*λ*_2_)*ρ* was less negative for **2b** and **2c**, indicating that this interaction, which may help to stabilize the Sb^+^–O^−^ bond, is present in **2a** and weakens for **2b** and **2c**. The presence of this hydrogen-bonding interaction in **2a** was further confirmed by NBO perturbation theory and deletion calculations (Supplementary Fig. 93).

### Reactivity

**Fig. 6:**
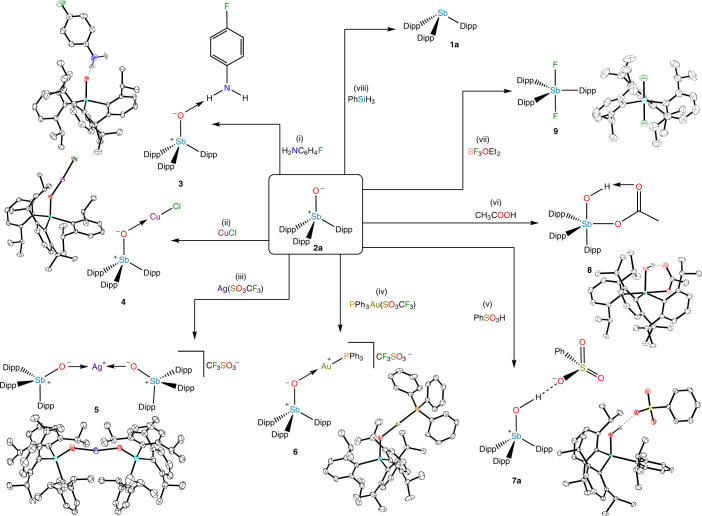
Reactivity of the stibine oxide 2a. (i) Reaction with an aniline to form a hydrogen-bonded adduct. (ii–iv) Reactions with copper(I) chloride, silver(I) triflate and triphenylphosphinegold(I) triflate to yield transition-metal coordination complexes. (v) Reaction with a sulfonic acid to yield a hydroxystibonium salt. (vi) Reaction with acetic acid to yield a *cis*-hydroxyacetatostiborane. (vii) Reaction with BF_3_ to yield a *trans*-difluorostiborane. (viii) Reaction with phenylsilane to yield stibine **1a**. Thermal ellipsoid plots at the 50% probability level are shown next to products; non-polar H atoms are omitted for clarity. Sb, teal; O, red; C, black; H, grey; N, blue; Ag, purple; F, green; S, yellow; Au, gold; P, orange; Cu, mauve.

With an isolated stibine oxide in hand, we next explored its chemistry. The bonding characteristics outlined above suggest that **2a** should exhibit O-centred Lewis-basic behaviour. Cooling a solution of **2a** in neat 4-fluoroaniline affords colourless blocks, which X-ray diffraction analysis confirmed to contain the stibine oxide-aniline hydrogen-bonded adduct **3** (Fig. 6(i)). In the HAR model, the hydrogen-bonding H atom of **3** is located on the N atom with a N–H distance of 1.04(2) Å. The N···O distance of 2.858(1) Å implies that the hydrogen-bonding interaction is of moderate strength. The Sb–O bond remains short at 1.8421(7) Å, but is statistically significantly lengthened as compared to **2a**. Consistent with this bond lengthening, the Sb–O IR stretching frequency decreases slightly from 779 cm^−1^ for **2a** to 762 cm^−1^ for **3**. Neither **2b** nor **2c** affords a similar product, consistent with the lower nucleophilicity of the O atoms in these species.

We next sought to determine whether this Lewis basicity would also manifest in metal ion coordination. Combination of **2a** with 1 equiv. of CuCl yielded the complex (Dipp_3_SbO)CuCl (**4**; Fig. 6(ii)), whereas combination with 0.5 equiv. of AgOTf yielded [(Dipp_3_SbO)_2_Ag][OTf] (**5**; Fig. 6(iii)). If ClAu(PPh_3_) was treated with AgOTf and **2a**, the salt [(Dipp_3_SbO)Au(PPh_3_)][OTf] (**6**; Fig. 6(iv)) was isolated. All three of the complexes were crystallographically characterized, and all three exhibit significantly nonlinear Sb–O–M angles (Supplementary Table 18). Solution of the structure of a second polymorph of **6** showed, however, that the complex can also take on a rigorously linear configuration. The Sb–O–M bending is most probably driven by crystal packing forces. We note that, in all cases, the geometry about the metal centres in **4**–**6** is nearly perfectly linear, as expected. Neither **2b** nor **2c** was able to form analogous complexes; the NMR resonances of these lighter pnictine oxides exhibited only minor shifts upon mixing with the metal precursors (Supplementary Fig. 49–51). We note that the strength of the intramolecular CH_*i*Pr_···O interaction decreases upon coordination of **2a** (Supplementary Tables 13 and 15).

Room-temperature treatment of **2a** with a strong Brønsted acid, PhSO_3_H, resulted in clean formation of the hydroxystibonium salt [Dipp_3_Sb(OH)][O_3_SPh] (**7a**; Fig. 6(v)). Crystallographic analysis of the salt confirmed protonation at the Sb-bound O atom, which lengthens the Sb–O bond to 1.9119(7) Å and decreases *ν*_SbO_ to 611 cm^−1^. Compound **2b** can be similarly protonated to yield [Dipp_3_As(OH)][O_3_SPh] (**7b**; Supplementary Fig. 106). Compound **2c** interacts much more weakly with PhSO_3_H, but titration with up to 10 equiv. of the acid results in a systematic shift in the NMR resonances of **2c**. This behaviour may arise from reversible formation of a hydrogen-bonded adduct in equilibrium with the dissociated species (Supplementary Figs. 59 and 60).

We were surprised to find that, in contrast, acetic acid not only protonates the O atom of **2a**, but adds across the Sb–O bond at room temperature, affording the neutral stiborane *cis*-Sb(OH)(OAc)Dipp_3_ (**8**; Fig. 6(vi)). This 1,2-addition chemistry highlights the unsaturated nature of the stiboryl (Sb=O/Sb^+^–O^−^) group. The *cis* isomer forms despite the expectation that the more sterically bulky Dipp groups would assume the less-crowded equatorial positions and that the more apicophilic hydroxy and acetoxy groups would assume the *trans*-disposed axial positions. An intramolecular hydrogen-bonding interaction is present between the hydroxy and acetoxy groups (O···O = 2.630(2) Å), which may be responsible for the *cis* configuration. Neither **2b** nor **2c** reacts in this manner with acetic acid (Supplementary Figs. 64–66). We have yet to observe any cycloaddition chemistry (Supplementary Fig. 74), but substrates continue to be explored.

Combination of **2a** and BF_3_·OEt_2_ at −78 °C results in rapid and clean conversion to **9**, which does not feature an ^11^B NMR signal, but does exhibit a single sharp ^19^F resonance at −74.35 ppm. X-ray diffraction analysis shows **9** to be the difluorostiborane *trans*-SbF_2_Dipp_3_ (Fig. 6(vii)). This reaction is the first in which we have observed cleavage of the Sb–O bond. The maintenance of the 5+ oxidation state of the Sb centre suggests that the reaction is an oxide transfer in which one oxide is exchanged for two fluoride groups, consistent with the fluorophilicity of organoantimony(V) Lewis acids^34^. We have not fully characterized the by-product, but boranes are known to form stable boroxines with O^2−^ (ref. ^35^). Unlike **8**, **9** features the apicophilic fluoro substituents in the expected *trans* geometry (Sb–F = 1.9673(34) and 1.9706(31) Å), most probably as a result of the increased electronegativity of F and a lack of hydrogen bonding. No deoxygenation was observed upon combination of **2b** or **2c** with BF_3_·OEt_2_.

Finally, we observed that PhSiH_3_ is able to abstract the O atom from **2a** to cleanly afford **1a** (Fig. 6(viii)). The reaction does not proceed at room temperature, but readily reaches completion within 1 h at 50 °C. Under these mild conditions, neither **2b** nor **2c** reacts with PhSiH_3_ (Supplementary Figs. 72 and 73).

## Discussion

The isolation of a monomeric stibine oxide, **2a**, was achieved using a kinetic stabilization approach in which the unsaturated Sb^+^–O^−^ bond is protected by the sterically bulky Dipp groups bound to the Sb centre. The isolation of **2a** has permitted the spectroscopic and crystallographic characterization of this functional group. In combination with these experimental measurements, theoretical calculations provide insight into the nature of the Pn–O bonding interaction, and the variation in this bonding as the pnictogen is varied from Sb to As to P. The increased accumulation of charge on the O atom confers upon **2a** reactivity that differs notably from that of **2b** and **2c**. We have described examples of **2a** acting as a hydrogen-bond acceptor, a transition-metal ligand and a Brønsted base. The unsaturated nature of the Sb^+^–O^−^ bond also allows it to engage in addition chemistry, as exemplified by the reaction with acetic acid. Finally, the Sb–O bond can be cleaved, either with maintenance of the Sb(V) oxidation state, as in the reaction with BF_3_, or with reduction to Sb(III), as in the reaction with PhSiH_3_. We will continue to investigate in greater depth each of these classes of reactions, and others, with an emphasis on comparing and contrasting the reactivity of stibine oxides with that of phosphine and arsine oxides.

## Online content

Any methods, additional references, Nature Portfolio reporting summaries, source data, extended data, supplementary information, acknowledgements, peer review information; details of author contributions and competing interests; and statements of data and code availability are available at 10.1038/s41557-023-01160-x.

## Supplementary information

Supplementary Information Experimental methods, experimental references, Supplementary Figs. 1–111 and Tables 1–31.

Supplementary Data 1 Cartesian coordinates of all computationally optimized molecular structures.

Supplementary Data 2 Source data for graphs in Supplementary Fig. 75.

Supplementary Data 3 Source data for graphs in Supplementary Fig. 85.

Supplementary Data 4 Source data for graphs in Supplementary Fig. 86.

Supplementary Data 5 Source data for graphs in Supplementary Fig. 87.

Supplementary Data 6 Source data for graphs in Supplementary Fig. 88.

Supplementary Data 7 Source data for graphs in Supplementary Fig. 96.

Supplementary Data 8 Crystallographic information file for compound **1a**.

Supplementary Data 9 Crystallographic information file for compound **1b**.

Supplementary Data 10 Crystallographic information file for compound **1c**.

Supplementary Data 11 Crystallographic information file for the orthorhombic polymorph of compound **2a**.

Supplementary Data 12 Crystallographic information file for the monoclinic polymorph of compound **2a**.

Supplementary Data 13 Crystallographic information file for compound **2b**.

Supplementary Data 14 Crystallographic information file for compound **2c**.

Supplementary Data 15 Crystallographic information file for compound **3**.

Supplementary Data 16 Crystallographic information file for compound **4**.

Supplementary Data 17 Crystallographic information file for compound **5**.

Supplementary Data 18 Crystallographic information file for the triclinic polymorph of compound **6**.

Supplementary Data 19 Crystallographic information file for the rhombohedral polymorph of compound **6**.

Supplementary Data 20 Crystallographic information file for compound **7a**.

Supplementary Data 21 Crystallographic information file for compound **7b**.

Supplementary Data 22 Crystallographic information file for compound **8**.

Supplementary Data 23 Crystallographic information file for compound **9**.

## Data Availability

All the data underlying the findings of this study are available in this Article and its Supplementary Information. All the crystallographic data for the structures reported in this Article have been deposited at the Cambridge Crystallographic Data Centre, under deposition numbers CCDC 2133036 (**1a**), 2182475 (**1b**), 2182476 (**1c**), 2182474 (**2a** orthorhombic), 2133037 (**2a** monoclinic), 2182477 (**2b**), 2182478 (**2c**), 2133038 (**3**), 2182479 (**4**), 2133039 (**5**), 2182480 (**6** triclinic), 2182481 (**6** rhombohedral), 2133040 (**7a**), 2182482 (**7b**), 2133041 (**8**) and 2133042 (**9**). Copies of the data can be obtained free of charge via https://www.ccdc.cam.ac.uk/structures/. Source data used to generate graphs in Figs. 4 and 5, as well as Supplementary Figs. 75, 85–88 and 96, are available as Supplementary Data files. The Cartesian coordinates of all computationally optimized molecular structures are provided in Supplementary Tables 19–31 and are also provided as Supplementary Data. Source data are provided with this paper.

## Source data

Source Data Fig. 4 Source data for line graphs in Figure 4.
Source Data Fig. 5 Source data for orbital energy plot in Figure 5.

## References

[1] Wittig G, Schöllkopf U (1954). Über triphenyl-phosphin-methylene als olefinbildende Reagenzien (I. Mitteil.). Chem. Ber..

[2] Mitsunobu O, Yamada M (1967). Preparation of esters of carboxylic and phosphoric acid via quaternary phosphonium salts. Bull. Chem. Soc. Jpn.

[3] Appel R (1975). Tertiary phosphane/tetrachloromethane, a versatile reagent for chlorination, dehydration, and P–N linkage. Angew. Chem. Int. Ed..

[4] Staudinger H, Meyer J (1919). Über neue organische Phosphorverbindungen III. Phosphinmethylenderivate und Phosphinimine. Helv. Chim. Acta.

[5] Yang T, Andrada DM, Frenking G (2018). Dative versus electron-sharing bonding in N-oxides and phosphane oxides R_3_EO and relative energies of the R_2_EOR isomers (E = N, P; R = H, F, Cl, Me, Ph). A theoretical study. Phys. Chem. Chem. Phys..

[6] Lipshultz JM, Li G, Radosevich AT (2021). Main group redox catalysis of organopnictogens: vertical periodic trends and emerging opportunities in group 15. J. Am. Chem. Soc..

[7] Huheey JE, Huheey CL (1972). Anomalous properties of elements that follow ‘long periods’ of elements. J. Chem. Educ..

[8] Mel’nikov NN, Rokilskaya MS (1938). The mechanism of the oxidation of organic compounds compounds by selenium dioxide. III. J. Gen. Chem. USSR.

[9] McEwen WE, Briles GH, Schulz DN (1972). Preparation and reactions of triphenylstibine oxide.. Phosphorus Relat. Group V Elem..

[10] Bordner J, Doak GO, Everett TS (1986). Crystal structure of 2,2,4,4-tetrahydro-2,2,2,4,4,4-hexaphenyl-1,3,2,4-dioxadistibetane (triphenylstibene oxide dimer) and related compounds. J. Am. Chem. Soc..

[11] Carmalt CJ, Crossley JG, Norman NC, Orpen AG (1996). The structure of amorphous Ph_3_SbO: information from EXAFS (extended X-ray absorption fine structure) spectroscopy. Chem. Commun..

[12] Ferguson G, Glidewell C, Kaitner B, Lloyd D, Metcalfe S (1987). Second determination of the structure of dimeric triphenylstibine oxide. Acta Crystallogr. C.

[13] Kather R (2015). Lewis-acid induced disaggregation of dimeric arylantimony oxides. Chem. Commun..

[14] Chen C-H, Gabbaï FP (2018). Coordination of a stibine oxide to a Lewis acidic stiborane at the upper rim of the biphenylene backbone. Dalton Trans..

[15] Coughlin, O. *Structural Manipulation of Organoantimony Cations for Tuneable Lewis Acidity and Reactivity of Palladium Organoantimony Complexes*. PhD thesis, Nottingham Trent Univ. (2021).

[16] Andrews L, Moores BW, Fonda KK (1989). Matrix infrared spectra of reaction and photolysis products of stibine and ozone. Inorg. Chem..

[17] Rivard E, Power PP (2007). Multiple bonding in heavier element compounds stabilized by bulky terphenyl ligands. Inorg. Chem..

[18] Wang Y (2015). Stabilization of elusive silicon oxides. Nat. Chem.

[19] Kobayashi R, Ishida S, Iwamoto T (2019). An isolable silicon analogue of a ketone that contains an unperturbed Si=O double bond. Angew. Chem. Int. Ed..

[20] Li L (2012). Germanone as the first isolated heavy ketone with a terminal oxygen atom. Nat. Chem..

[21] Wang Y (2013). Splitting molecular oxygen en route to a stable molecule containing diphosphorus tetroxide. J. Am. Chem. Soc..

[22] Huber F, Westhoff T, Preut H (1987). Tris(2,4,6-trimethylphenyl)antimony dihydroxide; synthesis and reaction with sulfonic acids RSO_3_H (R = C_6_H_5_, CF_3_). Crystal structure of [2,4,6-(CH_3_)_3_C_6_H_2_]_3_SbO·HO_3_SC_6_H_5_. J. Organomet. Chem..

[23] Wenger JS, Johnstone TC (2021). Unsupported monomeric stibine oxides (R_3_SbO) remain undiscovered. Chem. Commun..

[24] Wenger JS, Wang X, Johnstone TC (2021). H-atom assignment and Sb–O bonding of [Mes_3_SbOH][O_3_SPh] confirmed by neutron diffraction, multipole modeling, and Hirshfeld atom refinement. Inorg. Chem..

[25] Egorova IV, Zhidkov VV, Grinishak IP, Rodionova NA (2016). Novel organoantimony compounds [2,6-(OMe)_2_C_6_H_3_]_3_SbO and [2,6-(OMe)_2_C_6_H_3_]_3_Sb(NCO)_2_ ·0.5(CH_3_)_2_CO. Synthesis and structure. Russ. J. Gen. Chem..

[26] Sasaki S, Sutoh K, Murakami F, Yoshifuji M (2002). Synthesis, structure and redox properties of the extremely crowded triarylpnictogens: tris(2,4,6-triisopropylphenyl)phosphine, arsine, stibine and bismuthine. J. Am. Chem. Soc..

[27] Sasaki S, Sutoh K, Shimizu Y, Kato K, Yoshifuji M (2014). Oxidation of tris(2,4,6-triisopropylphenyl)phosphine and arsine. Tetrahedron Lett..

[28] Westhoff T, Huber F, Rüther R, Preut H (1988). Synthesis and structural characterization of some new triorganoantimony oxides. Molecular and crystal structure of tris(2,4,6-trimemethylphenyl)antimony dihydroxide. J. Organomet. Chem..

[29] Lindquist-Kleissler B, Weng M, Le Magueres P, George GN, Johnstone TC (2021). Geometry of pentaphenylantimony in solution: support for a trigonal bipyramidal assignment from X-ray absorption spectroscopy and vibrational spectroscopic data. Inorg. Chem..

[30] Kleemiss F (2021). Accurate crystal structures and chemical properties from NoSpherA2. Chem. Sci..

[31] Desiraju GR (2002). Hydrogen bridges in crystal engineering: interactions without borders. Acc. Chem. Res..

[32] Bader RFW (1991). A quantum theory of molecular structure and its applications. Chem. Rev..

[33] Lindquist-Kleissler B, Wenger JS, Johnstone TC (2021). Analysis of oxygen-pnictogen bonding with full bond path topological analysis of the electron density. Inorg. Chem..

[34] Pan B, Gabbaï FP (2014). [Sb(C_6_F_5_)_4_][B(C_6_F_5_)_4_]: an air stable, Lewis acidic stibonium salt that activates strong element-fluorine bonds. J. Am. Chem. Soc..

[35] Bhat KL, Markham GD, Larkin JD, Bock CW (2011). Thermodynamics of boroxine formation from the aliphatic boronic acid monomers R–B(OH)_2_ (R = H, H_3_C, H_2_N, HO and F): a computational investigation. J. Phys. Chem. A.

