# Supplementary material for: Isolation, bonding and reactivity of a monomeric stibine oxide
Source: Nat Chem. 2023 Mar 23;15(5):633–40. doi: 10.1038/s41557-023-01160-x (PMC10159848; doi:10.1038/s41557-023-01160-x)
Supplement: Supplementary file 1 — Experimental methods, experimental references, Supplementary Figs. 1–111 and Tables 1–31. [file 41557_2023_1160_MOESM1_ESM.pdf]

nature chemistry

Article https://doi.org/10.1038/s41557-023-01160-x

Isolation, bonding and reactivity of a monomeric stibine oxide

In the format provided by the authors and unedited

CONTENTS

	Page
Experimental methods	4
References	13
Figures 1 - 5: ^{1}H NMR, ^{13}C NMR, IR, ^{1}H-^{13}C HSQC, and ^{1}H-^{13}C HMBC spectra of **1a**	17
Figures 6 - 8: ^{1}H NMR, ^{13}C NMR, and IR spectra of **1b**	19
Figures 9 - 12: ^{1}H NMR, ^{13}C NMR, ^{31}P NMR, IR spectra of **1c**	21
Figures 13 - 16: ^{1}H NMR, ^{13}C NMR, ^{1}H-^{13}C HSQC and ^{1}H-^{13}C HMBC of **2a**	23
Figures 17 - 18: ^{1}H 2D EXSY and variable temperature ^{1}H NMR of **2a**	25
Figures 19 - 20: Experimental and simulated IR spectra of **2a**	27
Figures 21 - 23: ^{1}H NMR, ^{13}C NMR, and IR spectra of **2b**	28
Figures 24 - 28: ^{1}H NMR, ^{13}C NMR, ^{31}P NMR, and IR spectra; PXRD diffractogram of **2c**	29
Figures 29 - 33: ^{1}H NMR, ^{13}C NMR, ^{19}F NMR, and IR spectra; PXRD diffractogram of **3**	32
Figures 34 - 37: ^{1}H NMR, ^{13}C NMR, and IR spectra; PXRD diffractogram of **4**$\cdot(CHCl_3)_2$	34
Figures 38 - 42: ^{1}H NMR, ^{13}C NMR, ^{19}F NMR, and IR spectra; PXRD diffractogram of **5**$\cdot OEt_2$	36
Figures 43 - 48: ^{1}H NMR, ^{13}C NMR, ^{31}P NMR, ^{19}F NMR, and IR spectra; PXRD diffractogram of **6**	39
Figures 49 - 51: ^{1}H NMR and ^{31}P NMR spectra of **2b** + $AuPPh_3CF_3SO_3$; **2c** + $AuPPh_3CF_3SO_3$	42
Figures 52 - 54: ^{1}H NMR, ^{13}C NMR, and IR spectra of **7a**	43
Figures 55 - 58: ^{1}H NMR, ^{13}C NMR, and IR spectra; PXRD diffractogram of **7b**	45
Figures 59 - 60: ^{1}H NMR and ^{31}P NMR spectra of **2c** + benzenesulfonic acid	47
Figures 61 - 63: ^{1}H NMR, ^{13}C NMR, and IR spectra of **8**	48
Figures 64 - 66: ^{1}H NMR and ^{31}P NMR spectra of **2b** + acetic acid; **2c** +acetic acid	49
Figures 67 - 70: ^{1}H NMR, ^{13}C NMR, ^{19}F NMR, and IR spectra of **9**	51
Figures 71 - 73: ^{1}H NMR-monitored reactions between **2a**-**c** and phenylsilane	53
Figure 74: ^{1}H NMR-monitored reactions between **2a** and potential cycloaddition substrates	54
Figure 75: Optimized structures of **2a**-**c**, Pn–O frequencies, surface scans, and force constants	55
Figures 76 - 81: Molecular graphs of **2a**-**c**; intramolecular O···H bond paths of **2a**-**c**	56
Figures 82 - 84: Ball-and-stick representations of atomic coordinates used in theoretical studies	61
Figures 85 - 88: Full bond path topological analyses	63
Figures 89: Non-covalent interaction analysis of **2a**-**c**	66
Figures 90: Deformation densities of **2a**-**c**	67

Figure 91: NLMO plots of Sb–O bonding and antibonding orbitals; O-centered lone pairs in **2a** 68
Figures 92 - 93: Pre-orthogonalized NLMO plots **2a** depicting donor–acceptor interactions 69
Figure 94: Pre-orthogonalized NBO plots for **4**, **5**, **6**-linear, and **6**-bent 71
Figure 95: Thermal ellipsoid plots and space-fill diagrams for **1a**-**c** 72
Figure 96: Thermal ellipsoid plots, bond lengths, and covalent ratios for **2a**-**c** 72
Figures 97 - 99: Thermal ellipsoid plots for **1a**, **2a**; space-fill diagrams of **2a** 73
Figures 100 - 108: Thermal ellipsoid plots: **3**, **4**·$(CHCl_3)_2$, **5**·OEt_2, **6**-linear, **6**-bent, **7a**, **7b**, **8** and **9** 74
Figure 109: Full normalized Sb-K-edge XAS spectra for **1a**, **A**, **B**, **C**, and **2a** 79
Figure 110: Breakdown of major EXAFS components for **A** 79
Figure 111: Breakdown of major EXAFS components for **2a** 80
Tables 1 - 6: Crystallographic refinement parameters 81
Table 7: EXAFS refinement parameters 86
Tables 8 - 10: Energy decomposition analyses of **2a**-**c** 87
Table 11: Bond critical point analyses of Pn–O and O–A interactions for compounds **2a**-**8**, **A**, **B** 88
Tables 12 - 13: Bond critical point analyses of intramolecular O···H interactions 89
Table 14: Select donor–acceptor interactions of **2a** 90
Table 15: NPA, WBI, NLMO, and E2 analyses of compounds **2a**-**8**, **A**, **B** 90
Table 16: Deletion energies of **2a**-**c** 91
Table 17: Force constants of Pn–O stretch in **2a**-**c** 91
Table 18: Selected bond lengths and angles for **4**·$(CHCl_3)_2$, **5**·OEt_2, **6**-linear, and **6**-bent 91
Tables 19 - 31: Cartesian coordinates used for theoretical study of **A**, **B**, **2a**-**8** 92

Experimental methods

General methods. Reagents and solvents were purchased from commercial vendors and used as received unless otherwise specified. Commercially purchased CuCl was purified by dissolution in concentrated HCl followed by dilution with H_2O to precipitate colorless crystals that were washed with ethanol and diethyl ether before being dried *in vacuo*. The dry, colorless crystals of CuCl were ground into a fine powder immediately before use. Tetrahydrofuran (THF), dichloromethane (DCM), and acetonitrile (MeCN) were purified using an Innovative Technology PURE-SOLV solvent purification system. All solvents were dried over 3-Å molecular sieves. All syntheses were performed using flame-dried glassware under a nitrogen atmosphere. NMR spectra were collected using a Bruker Avance III HD 500 spectrometer equipped with a multinuclear Smart Probe. HSQC and HMBC NMR data were collected using a Bruker Avance III HD 800 MHz spectrometer equipped with a TCI cryoprobe. Signals in the ^{1}H and ^{13}C NMR spectra are reported in ppm as chemical shifts from tetramethylsilane and were referenced using the $CHCl_3$ (^{1}H, 7.26 ppm), CHD_2CN (^{1}H, 1.94 ppm), $CHD_2C_6D_5$ (^{1}H, 2.08 ppm), $CDCl_3$ (^{13}C, 77.16 ppm), and CD_3CN (^{13}C, 118.26 ppm) solvent signals. The frequencies of ^{19}F NMR signals are reported in ppm as chemical shifts from $CFCl_3$ (**3**, **5**·OEt_2, and **6** referenced to $BF_3 \cdot OEt_2$ at –152.8 ppm and **9** referenced to 4-fluoroaniline at –126.88 ppm). ^{31}P NMR signals are reported as chemical shifts from 85% H_3PO_4 and were referenced using triphenylphosphine (–5.35 ppm). Infrared (IR) spectra were collected on KBr pellets using a PerkinElmer Spectrum One FT-IR spectrometer. Mass spectrometry measurements were collected using an LTQ-Orbitrap Velos Pro MS instrument. Elemental analyses were performed by Midwest Microlabs (Indianapolis, IN) using an Exeter CE440 analyzer or by NuMega Resonance Labs using a Perkin Elmer PE2400-Series II, CHNS/O analyzer. Melting point data were collected with an electrothermal Mel-Temp apparatus and a partial-immersion thermometer; temperatures are uncorrected.

Synthesis of 1-bromo-2,6-diisopropylbenzene. 1-Bromo-2,6-diisopropylbenzene was synthesized following a literature procedure.[1] In brief, 2,6-diisopropylaniline (22.5 mL, 119 mmol) was added dropwise to rapidly stirring conc. HBr (100 mL) to form a colorless suspension. The reaction mixture was cooled to –50 °C and $NaNO_2$ (13.24 g, 192 mmol) was added in portions to form a viscous brown suspension. Reddish brown gas evolved from the mixture. The reaction mixture was diluted with conc. HBr (33 mL) and water (33 mL) and held at –50 °C for 1 h. The reaction mixture was then diluted with OEt_2 (100 mL) and allowed to warm to –15 °C. The mixture was again cooled to –50 °C and Na_2CO_3 (24.5 g, 231 mmol) was added in portions. The mixture was allowed to stir at room temperature overnight. The following day, the solid was removed by vacuum filtration and the filtrate was extracted with OEt_2 (150 mL). The aqueous phase was then back-extracted with OEt_2 (2 × 50 mL). The combined organic phases were washed with water (2 × 150 mL) and brine (100 mL) before being dried over Na_2SO_4 for 15 min. The solvent was stripped to yield a crude red oil that was purified by flash chromatography (SiO_2/hexanes) to yield the product as a colorless oil. Yield: 9.10 g (32%). ^{1}H NMR spectroscopic data collected from the product agree with those previously reported.[1] **^{1}H NMR (500 MHz, $CDCl_3$)** δ 7.24 (d, *J* = 7.7 Hz, 1H), 7.13 (d, *J* = 7.6 Hz, 2H), 3.51 (hept, *J* = 6.9 Hz, 2H), 1.25 (d, *J* = 6.9 Hz, 12H).

Synthesis of iodosobenzene. Iodosobenzene was synthesized following a literature procedure.[2] In brief, iodobenzene diacetate (2.004 g, 6.22 mmol) was suspended in water (75 mL). A solution of NaOH (1.453 g, 36.4 mmol) in water (75 mL) was added dropwise to form a yellow suspension. The reaction mixture was allowed to stir at room temperature for 2 h. The mixture was decanted and diluted with water (100 mL) three times before the solid was collected by vacuum filtration. The yellow powder was washed with water (3 × 10 mL) and chloroform (2 × 5 mL) and dried en vacuo. Yield: 598 mg (44%). No further characterization data were collected.

Synthesis of triphenylsibine oxide dimer (($(Ph_3SbO)_2$) (A). Triphenylstibine oxide was synthesized according to a literature reference.[3] In brief, triphenylstibine (1.003 g, 2.84 mmol) was dissolved in acetone (27 mL) and 50% H_2O_2 in water (140 µL) was added to the colorless solution. An amorphous precipitate formed and the supernatant was decanted. Colorless crystals grew from the supernatant. The mother liquor was decanted and the crystals were washed with hexanes before being collected by vacuum filtration. Yield: 35 mg (3.5%). ^{1}H NMR spectroscopic data collected from the product agree with those previously reported.[3] **^{1}H NMR (500 MHz, $CDCl_3$)** δ 8.26 – 8.17 (m, 6H), 7.75 (d, *J* = 7.0 Hz, 6H), 7.57 – 7.43 (m, 9H), 7.42 – 7.31 (m, 9H).

Synthesis of trimesitylstibine. Trimesitylstibine was synthesized following a reported protocol.[4] In brief, a dry 250 mL two-necked round bottom flask fitted with a water-jacketed Liebig condenser was charged with a stir bar and magnesium turnings (693 mg, 28.5 mmol). The magnesium turnings were suspended in dry THF (80 mL) and activated with 1,2-dibromoethane (618 µL, 7.2 mmol). 2-Bromomesitylene (3.22 mL, 21.3 mmol) was added dropwise to the mixture and the reaction was refluxed for 3 h. The reaction mixture was cooled to room temperature and a solution of $SbCl_3$ (1.619 g, 7.1 mmol) in THF (18 mL) was added dropwise. The reaction mixture was refluxed overnight. The reaction mixture was cooled to room temperature and diluted with OEt_2 (150 mL) and water (150 mL). The aqueous layer was back-extracted with OEt_2 (2 × 50 mL). The combined organic phases were then washed with water (2 × 150 mL) and with brine (100 mL). The organic layer was dried over Na_2SO_4. The solvent was removed under reduced pressure to yield a crude yellow oil. Ethanol was added dropwise with sonication to precipitate a white powder that was collected via vacuum filtration. The product was washed with ethanol (3 × 5 mL) and dried under vacuum. Yield: 2.14 g (63%). ^{1}H NMR spectroscopic data collected from the product agree with those previously reported.[4] **^{1}H NMR (500 MHz, $CDCl_3$)** δ 6.83 (6 H, s), 2.29 (18 H, s), 2.27 (9 H, s).

Synthesis of *trans*-dihydroxytrimesitylstibine ($Mes_3Sb(OH)_2$) (B). *trans*-Dihydroxytrimesitystiborane was synthesized following a reported protocol.[5] 50% H_2O_2 in water (200 µL) was added to a solution of trimesitylstibine (274 mg, 576 µmol) in acetone (40 mL). The reaction mixture was allowed to stir at room temperature for 1 h. The solvent was stripped to yield a white powder. The product was suspended in hexanes and collected by vacuum filtration. Yield: 240 mg (81%). ^{1}H NMR spectroscopic data collected from the product agree with those previously reported.[5] **^{1}H NMR (500 MHz, $CDCl_3$)** δ 6.97 (6 H, s), 2.57 (18 H, s), 2.31 (9 H, s).

Synthesis of tris(2,6-diisopropylphenyl)stibine ($Dipp_3Sb$) (1a). A dry 250 mL two-necked round bottom flask fitted with a water-jacketed Liebig condenser was charged with a stir bar and magnesium turnings (405 mg, 16.6 mmol). The magnesium turnings were suspended in dry THF (30 mL) and activated with 1,2-dibromoethane (360 µL, 4.2 mmol). 1-Bromo-2,6-diisopropylbenzene (2.6 mL, 12.5 mmol) was then added dropwise and the reaction was refluxed for 3 h. After the magnesium turnings had been consumed, the colorless solution was cooled to –78 °C and CuCl (1.244 g, 12.5 mmol) was added against a backflow of N_2. The reaction mixture was allowed to warm to room temperature and stir overnight. The resulting suspension was cooled to –78 °C and a solution of trichlorostibine (0.950 g, 4.2 mmol) in THF (10 mL) was then added dropwise to the reaction mixture. The reaction mixture was stirred at room temperature for 4 h before being refluxed for 20 h. The reaction mixture was cooled to room temperature and diluted with OEt_2 (150 mL) and water (150 mL). The aqueous layer was back-extracted with OEt_2 (2 × 50 mL). The combined organic phases were then washed with water (2 × 150 mL) and with brine (150 mL). The organic layer was dried over Na_2SO_4. The solvent was removed under reduced pressure. The resulting yellow oil was diluted with hexanes and purified by flash chromatography (SiO_2/hexanes). The collected fractions were stripped of solvent to obtain a white powder. This crude product was of sufficient purity for the

routine synthesis of **2**. Yield: 1.464 g (58%). To obtain analytically pure material, 684 mg of crude product was recrystallized from a mixture of MeCN/OEt_2 to yield 410 mg (60%) of colorless crystals. Crystals suitable for X-ray diffraction were obtained similarly. M.p. 210 °C. **Found:** C, 71.83; H, 8.70%. **Calc. for $C_{36}H_{51}Sb$:** C, 71.40; H, 8.49%. **ESI-MS (*m/z*) [M+H]$^+$** 605.26 (calc 605.3). **^{1}H NMR (500 MHz, $CDCl_3$)** δ 7.27 (t, *J* = 7.5 Hz, 3H), 7.13 (d, *J* = 8.1 Hz, 6H), 3.34 (hept, *J* = 6.6 Hz, 6H), 1.35 (d, *J* = 6.5 Hz, 9H), 1.14 (d, *J* = 6.1, 9H), 1.13 (d, *J* = 6.1, 9H), 0.55 (d, *J* = 6.5 Hz, 9H). **^{13}C{^{1}H} NMR (125 MHz, $CDCl_3$)** δ 155.35, 155.08, 140.95, 129.14, 124.86, 122.99, 37.33, 36.28, 26.08, 25.01, 24.58, 23.32.

Synthesis of tris(2,6-diisopropylphenyl)arsine ($Dipp_3As$) (1b). A dry 100 mL two-necked round bottom flask fitted with a water-jacketed Liebig condenser was charged with a stir bar and magnesium turnings (0.3096 mg, 12.7 mmol). The magnesium turnings were suspended in dry THF (30 mL) and activated with 1,2-dibromoethane (275 μL, 3.2 mmol). 1-Bromo-2,6-diisopropylbenzene (1.97 mL, 9.6 mmol) was then added dropwise and the reaction was refluxed for 3 h. After the magnesium turnings had been consumed, the colorless solution was cooled to –78 °C and CuCl (0.946 g, 9.6 mmol) was added against a backflow of N_2. The reaction mixture was allowed to warm to room temperature and stir overnight. The resulting suspension was cooled to –78 °C and a solution of trichloroarsine (577 mg, 3.2 mmol) in THF (8 mL) was then added dropwise to the reaction mixture. The reaction mixture was allowed to warm to room temperature and then stirred for 30 min before being refluxed for 24 h. The reaction mixture was cooled to room temperature and diluted with OEt_2 (150 mL) and water (150 mL). The aqueous layer was back-extracted with OEt_2 (2 × 50 mL). The combined organic phases were then washed with water (2 × 150 mL) and with brine (150 mL). The organic layer was dried over Na_2SO_4. The solvent was removed under reduced pressure to yield a white solid coated in oil. The residue was suspended in MeCN and collected by vacuum filtration. The powder was recrystallized from a mixture of MeCN and OEt_2 to yield 1.001 g (56%) of colorless crystals. Crystals suitable for X-ray diffraction were obtained similarly. M.p. 220 °C (decomp). **Found:** C, 77.17; H, 9.29%. **Calc. for $C_{36}H_{51}As$:** C, 77.39; H, 9.20%. **ESI-MS (*m/z*) [M+H]$^+$** 558.2 (calc 558.3). **^{1}H NMR (500 MHz, $CDCl_3$)** δ 7.26 (t, *J* = 7.7 Hz, 3H), 7.09 (d, *J* = 7.7 Hz, 6H), 3.43 (hept, *J* = 6.6 Hz, 3H), 3.29 (hept, *J* = 6.6 Hz, 3H), 1.28 (d, *J* = 6.6 Hz, 9H), 1.10 (d, *J* = 6.8 Hz, 9H), 1.04 (d, *J* = 6.7 Hz, 9H), 0.50 (d, *J* = 6.6 Hz, 9H). **^{13}C{^{1}H} NMR (125 MHz, $CDCl_3$)** δ 153.63, 153.20, 139.44, 128.95, 125.24, 122.97, 33.83, 32.95, 25.50, 24.38, 24.15, 22.86.

Synthesis of tris(2,6-diisopropylphenyl)phosphine ($Dipp_3P$) (1c). A dry 100 mL two-necked round bottom flask fitted with a water-jacketed Liebig condenser was charged with a stir bar and magnesium turnings (0.5103 g, 20.9 mmol). The magnesium turnings were suspended in dry THF (30 mL) and activated with 1,2-dibromoethane (0.45 mL, 5.2 mmol). 1-Bromo-2,6-diisopropylbenzene (3.31 mL, 15.7 mmol) was then added dropwise and the reaction was refluxed for 3 h. After the magnesium turnings had been consumed, the colorless solution was cooled to –78 °C and CuCl (1.559 g, 15.7 mmol) was added against a backflow of N_2. The reaction mixture was allowed to warm to room temperature and stir overnight. The resulting suspension was cooled to –78 °C and a solution of trichlorophosphine (0.711 g, 5.2 mmol) in THF (10 mL) was added dropwise to the reaction mixture. The reaction mixture was warmed to room temperature and stirred for 30 min before being refluxed for an additional 24 h. The reaction mixture was cooled to room temperature and diluted with OEt_2 (150 mL) and water (150 mL). The aqueous layer was back-extracted with OEt_2 (2 × 50 mL). The combined organic phases were then washed with water (2 × 150 mL) and with brine (150 mL). The organic layer was dried over Na_2SO_4. The solvent was removed under reduced pressure to yield a pale green solid coated in oil. The residue was suspended in MeCN and collected by vacuum filtration. The solid was recrystallized from a mixture of MeCN and OEt_2 to yield 1.445 g (53%) of pale yellow-green crystals. Crystals suitable for X-ray diffraction were obtained similarly. M.p. 313 °C (decomp). **Found:** C, 83.98; H, 10.01%. **Calc. for $C_{36}H_{51}P$:** C, 84.00; H, 9.99%. **ESI-MS (*m/z*) [M+H]$^+$** 515.4

(calc 515.4). **^{1}H NMR (500 MHz, $CDCl_3$)** δ 7.27 (t, *J* = 7.7 Hz, 3H), 7.08 (dd, *J* = 7.7, 3.3 Hz, 6H), 3.49 (hept, *J* = 6.5 Hz, 3H), 3.48 (hept, *J* = 6.5 Hz, 3H), 1.16 (d, *J* = 6.7 Hz, 18H), 0.71 (d, *J* = 6.7 Hz, 18H). **^{13}C{^{1}H} NMR (125 MHz, $CDCl_3$)** δ 153.38 (d, *J* = 8.75 Hz), 135.13 (d, *J* = 25.6 Hz), 129.18, 124.20 (d, *J* = 4.3 Hz), 32.23 (d, *J* = 17.6 Hz), 24.69, 23.12. **^{31}P{^{1}H} NMR (202 MHz, $CDCl_3$)** δ –50.41.

Synthesis of tris(2,6-diisopropylphenyl)stibine oxide ($Dipp_3SbO$) (2a). 1a (1.35 g, 2.2 mmol) was added to a suspension of iodosobenzene (502 mg, 2.3 mmol) in DCM (20 mL). The solid was rapidly consumed to produce a faintly yellow hazy solution. The reaction mixture was passed through a Celite pad to remove excess iodosobenzene and the filtrate was stripped of its solvent to yield a white powder coated with a pale yellow oil. The crude product was washed with cold pentane to yield **2a** as an analytically pure colorless solid. Yield: 862 mg (62%). Crystals (monoclinic polymorph) suitable for X-ray diffraction were grown from pentane. **2a** (492 mg) was recrystallized from hot MeCN to obtain large colorless block crystals (398 mg, 81%). Crystals (orthorhombic polymorph) suitable for X-ray diffraction were grown similarly. M.p. 181 °C. **Found:** C, 69.22; H, 8.33% **Calc. for $C_{36}H_{51}OSb$:** C, 69.57; H, 8.27%. **ESI-MS (*m/z*) [M+H]$^+$** 621.27 (calc 621.3). **IR (KBr, cm^{-1})** ν_{SbO} 779 (s). **^{1}H NMR (500 MHz, $CDCl_3$)** δ 7.41 (t, *J* = 7.7 Hz, 3H), 7.32 (d, *J* = 6.9 Hz, 3H), 7.17 (d, *J* = 6.8 Hz, 3H), 4.41 (br s, 3H), 3.01 (br s, 3H), 1.45 (br s, 9H), 1.17 (br s, 9H), 0.99 (br s, 9H), 0.71 (br s, 9H). **^{1}H NMR (500 MHz, CD_3CN)** δ 7.47 (t, *J* = 7.7 Hz, 3H), 7.37 (d, *J* = 7.5 Hz, 3H), 7.30 (d, *J* = 7.6 Hz, 3H), 4.22 (hept, *J* = 6.3 Hz, 3H), 3.01 (hept, *J* = 6.2 Hz, 3H), 1.36 (d, *J* = 5.4 Hz, 9H), 1.19 (d, *J* = 5.8 Hz, 9H), 0.97 (d, *J* = 6.0 Hz, 9H), 0.71 (d, *J* = 5.6 Hz, 9H). **^{13}C{^{1}H} NMR (125 MHz, $CDCl_3$)** δ 157.28, 152.01, 142.03, 131.46, 126.72, 125.08, 36.93, 33.90, 27.39 (br s), 24.83 (br s, 2C), 24.42 (br s).

Synthesis of tris(2,6-diisopropylphenyl)arsine oxide ($Dipp_3AsO$) (2b). 1b (511 mg, 0.9 mmol) and *m*CPBA (238 mg, 1.4 mmol) were dissolved in DCM (25 mL) to form a colorless solution that was allowed to stir at room temperature. The reaction was monitored by TLC (hexanes/SiO_2). After 2 h, no starting material was detectable by TLC, and the reaction mixture was diluted with 50 mL DCM and washed with a saturated aqueous solution of $NaHCO_3$ (50 mL) and brine (50 mL). The organic phase was dried over Na_2SO_4 before being stripped of solvent under reduced pressure to yield a white powder. The crude product was dissolved in ethyl acetate and purified by flash chromatography (1:4 ethyl acetate:hexanes/SiO_2). Crystals suitable for X-ray diffraction were grown in bulk from a mixture of OEt_2 and MeCN. The solvent was decanted, and the colorless crystals were washed with MeCN and dried *in vacuo*. Yield: 274 mg (54%) M.p. 298 °C (decomp). **Found:** C, 75.23; H, 8.91%. **Calc. for $C_{36}H_{51}AsO$:** C, 75.24; H, 8.94 %. **ESI-MS (*m/z*) [M+H]$^+$** 575.3 (calc 575.3). **^{1}H NMR (500 MHz, $CDCl_3$)** δ 7.38 (t, *J* = 7.6 Hz, 3H), 7.30 (d, *J* = 7.5 Hz, 3H), 7.14 (d, *J* = 7.5 Hz, 3H), 3.97 (hept, *J* = 6.4 Hz, 3H), 3.25 (hept, *J* = 9.5 Hz, 3H), 1.41 (d, *J* = 6.2 Hz, 9H), 1.11 (d, *J* = 6.5 Hz, 9H), 0.94 (d, *J* = 6.7 Hz, 9H), 0.70 (d, *J* = 6.3 Hz, 9H). **^{13}C{^{1}H} NMR (125 MHz, $CDCl_3$)** δ 155.73, 149.57, 142.76, 130.92, 126.87, 124.96, 33.38, 32.02, 27.54, 25.20, 24.08.

Synthesis of tris(2,6-diisopropylphenyl)phosphine oxide ($Dipp_3PO$) (2c). 1c (91 mg, 0.17 mmol) and *m*CPBA (124 mg, 0.71 mmol) were dissolved in DCM (25 mL) to form a pink solution that was allowed to stir at room temperature. The reaction was monitored by TLC (hexanes/SiO_2). After 2 h, no starting material was detectable by TLC, and the reaction mixture was diluted with DCM (40 mL) and washed with a saturated aqueous solution of $NaHCO_3$ (40 mL) and brine (40 mL). During the washes, the pink color was discharged from the organic phase, which was dried over Na_2SO_4 before being stripped of solvent under reduced pressure to yield a white powder. The crude product was dissolved in ethyl acetate and purified by flash chromatography (1:5 ethyl acetate:hexanes/SiO_2). Crystals suitable for X-ray diffraction were grown in bulk from a mixture of OEt_2 and MeCN. The solvent was decanted, and the colorless crystals were washed with MeCN and dried *in vacuo*. Yield: 51.2 mg (55%) M.p. 228 °C (decomp). **Found:** C, 80.92; H, 9.87%. **Calc. for $C_{36}H_{51}PO$:** C, 81.46; H, 9.69 %. **ESI-MS (*m/z*) [M+H]$^+$** 531.3751 (calc 531.3750). **^{1}H NMR**

(500 MHz, $CDCl_3$) δ 7.37 (t, *J* = 7.7 Hz, 3H), 7.27 (dd, *J* = 4.5, 3.5 Hz, 3H), 7.12 (dd, *J* = 7.6, 4.4 Hz, 3H), 3.37 (hept, *J* = 6.6 Hz, 3H), 3.31 (hept, *J* = 6.6 Hz, 3H), 1.32 (d, *J* = 6.4 Hz, 9H), 1.07 (d, *J* = 6.7 Hz, 9H), 0.94 (d, *J* = 6.8 Hz, 9H), 0.66 (d, *J* = 6.5 Hz, 9H). **$^{13}C\{^1H\}$ NMR (125 MHz, $CDCl_3$)** δ 156.73 (d, *J* = 8.8 Hz), 149.36 (d, *J* = 11.4 Hz), 139.04 (d, *J* = 91.3 Hz), 130.695 (d, *J* = 3.8 Hz), 126.08 (d, *J* = 10 Hz), 124.31 (d, *J* = 10 Hz), 32.34, 32.29, 32.25, 26.86, 24.81, 23.76. **$^{31}P\{^1H\}$ NMR (202 MHz, $CDCl_3$)** δ 25.33. The elemental composition of **2c** found from elemental analysis was consistently outside of the acceptable deviation from the theoretical value. In addition to the NMR spectra, we also obtained PXRD data from freshly prepared bulk solid; these powder data agreed with the simulated powder diffractogram generated from the crystal structure of **2c** (Supplementary Figure 27). Furthermore, high-resolution mass spectrometry data were collected to corroborate the proposed elemental composition.

Synthesis of tris(2,6-diisopropylphenyl)stibine oxide 4-fluoroaniline ($Dipp_3SbO·H_2NPhF$) (3). A solution of **2a** (76 mg, 0.12 mmol) in neat 4-fluoroaniline (200 μL) was cooled to 4 °C to grow colorless crystals suitable for X-ray diffraction. The crystals were collected by vacuum filtration and dried under vacuum. Yield: 4 mg (4%). **IR (KBr, cm^{-1})** ν_{SbO} 762 (s). **^1H NMR (500 MHz, $CDCl_3$)** δ 7.41 (t, *J* = 7.6 Hz, 3H), 7.33 (br s, 3H), 7.18 (br s, 3H), 6.85 (t, *J* = 8.6 Hz, 2H), 6.66-6.58 (m, 2H), 4.42 (br s, 3H), 3.52 (br s, 2H), 3.02 (br s, 3H), 1.44 (br s, 9H), 1.17 (br s, 9H), 1.00 (br s, 9H), 0.72 (br s, 9H). **$^{13}C\{^1H\}$ NMR (125 MHz, $CDCl_3$)** δ 157.40 (br s), 156.67 (d, *J* = 235 Hz), 152.08, 142.58, 142.16, 131.48, 126.76, 125.10, 116.23 (d, *J* = 7.5 Hz), 115.83 (d, *J* = 22.5 Hz), 36.94, 33.91, 27.38, 24.86 (br s, 2C), 24.46. **$^{19}F\{^1H\}$ NMR (470 MHz, $CDCl_3$)** δ –126.88. Compound **3** is thermally unstable and elemental analyses were consistently unsuccessful. In addition to the NMR spectra, we also obtained PXRD data from freshly prepared bulk solid; these powder data agreed with the simulated powder diffractogram generated from the crystal structure of **3** (Supplementary Figure 33).

Synthesis of (tris(2,6-diisopropylphenyl)stibine oxide)chlorocopper(I) chloroform disolvate ($Dipp_3SbOCuCl·(CHCl_3)_2$) (4·($CHCl_3$)$_2$). **2a** (48.5 mg, 0.07 mmol) and CuCl (7.7 mg, 0.07 mmol) were dissolved in MeCN (8 mL) and stirred for 15 min at room temperature. OEt_2 (30 mL) was added to the colorless solution and the reaction mixture was stripped of solvent under reduced pressure. The resulting colorless solid was suspended in pentane and collected by vacuum filtration. The white solid was recrystallized by vapor diffusion of pentane into chloroform. Crystals suitable for X-ray diffraction were grown similarly. Yield: 16 mg (21%). M.p. 202 °C (decomp). **IR (KBr, cm^{-1})** ν_{SbO} 732. **ESI-MS (*m/z*) $[M–Cl]^+$** 683.2253 (calc. 683.2268); **$[M–Cl+2a]^+$** 1303.5243 (calc. 1303.5246). **^1H NMR (500 MHz, $CDCl_3$)** δ 7.49 (t, *J* = 7.6 Hz, 3H), 7.38 (d, *J* = 7.4 Hz, 3H), 7.24 (d, *J* = 7.5 Hz, 3H), 3.80 (br hept, 3H), 2.88 (br hept, 3H), 1.61 (br s, 9H), 1.17 (br s, 9H), 0.99 (br s, 9H), 0.77 (br s, 9H). **$^{13}C\{^1H\}$ NMR (125 MHz, $CDCl_3$)** δ 156.46, 152.10, 140.49, 132.71, 127.85, 126.00, 37.56, 35.35, 28.26 (br s), 25.17 (br s, 2C), 24.59 (br s). Compound **4**·$(CHCl_3)_2$ is unstable in air and combustion microanalyses were consistently unsuccessful. In addition to the NMR spectra, we also obtained PXRD data from freshly prepared bulk solid; these powder data agreed with the simulated powder diffractogram generated from the crystal structure of **4**·$(CHCl_3)_2$ (Supplementary Figure 37). Furthermore, high-resolution mass spectrometry data were collected to corroborate the proposed elemental composition.

Synthesis of bis(tris(2,6-diisopropylphenyl)stibine oxide)silver(I) trifluoromethanesulfonate diethyl ether solvate ($[Ag(Dipp_3SbO)_2](CF_3SO_3)·OEt_2$) (5·$OEt_2$). In the absence of light, **2a** (75 mg, 0.12 mmol) and silver triflate (11 mg, 0.060 mmol) were dissolved in MeCN (3 mL) and stirred for 30 min at room temperature. OEt_2 (30 mL) was added to the colorless solution and the mixture was placed in a –20 °C freezer overnight to grow colorless crystals that were suitable for X-ray diffraction. The solid was collected

by vacuum filtration and washed with OEt_2. Yield: 30 mg (33%). **IR (KBr, cm^{-1})** ν_{SbO} 637. **ESI-MS (*m/z*) [M–2a–CF_3SO_3]$^+$** 727.45 (calc 727.2). **^{1}H NMR (500 MHz, CD_3CN)** δ 7.51 (t, *J* = 7.7 Hz, 6H), 7.37 (d, *J* = 7.6 Hz, 6H), 7.33 (d, *J* = 7.7 Hz, 6H), 4.01 (hept, *J* = 6.1 Hz, 6H), 3.42 (quart, *J* = 7.0 Hz, 4H), 2.95 (hept, *J* = 6.5 Hz, 6H), 1.33 (d, *J* = 5.6 Hz, 18H), 1.19 (d, *J* = 5.9 Hz, 18H), 1.12 (t, *J* = 7.0 Hz, 6H), 0.93 (d, *J* = 6.1 Hz, 18H), 0.72 (d, *J* = 5.7 Hz, 18H). **^{13}C{^{1}H} NMR (125 MHz, CD_3CN)** δ 156.93, 153.24, 142.32, 133.05, 128.01, 126.83, 66.24, 38.19, 35.31, 27.43, 25.09, 24.80, 24.49, 15.59. **^{19}F{^{1}H} NMR (470 MHz, CD_3CN)** δ –79.33. The crystals of **5**·OEt_2 are both thermally and photolytically sensitive and combustion microanalyses were consistently unsuccessful. In addition to the NMR spectra, we also obtained PXRD data from freshly prepared bulk solid; these powder data agreed with the simulated powder diffractogram generated from the crystal structure of **5**·OEt_2 (Supplementary Figure 42).

Synthesis of (tris(2,6-diisopropylphenyl)stibine oxide)(triphenylphosphine)gold(I) trifluoromethanesulfonate ($Dipp_3SbOAuPPh_3CF_3SO_3$) (6). In the absence of light, **2a** (48.5 mg, 0.07 mmol), PPh_3AuCl (48.5 mg, 0.07 mmol), and silver triflate (7.7 mg, 0.07 mmol) were suspended in DCM, sonicated, and stirred at room temperature for 1 h. The reaction mixture was filtered through a plug of laboratory tissue and hexanes were layered on top of the filtrate to grow colorless crystals overnight. The crystals were collected by vacuum filtration, washed with pentane (3 × 3 mL), and dried *in vacuo*. Crystals suitable for X-ray diffraction were grown similarly. Yield: 65 mg (64 %). M.p. 149 °C (decomp). **IR (KBr, cm^{-1})** ν_{SbO} 638. **ESI-MS (*m/z*) [M–CF_3SO_3]$^+$** 1079.3549 (calc 1079.3549). **^{1}H NMR (500 MHz, $CDCl_3$)** δ 7.61-7.50 (br m, 6H), 7.49-7.41 (br m, 7H), 7.35 (d, *J* = 6.0 Hz, 7H), 7.31-7.27 (m, 4H), 3.67 (br s, 3H), 2.87 (br s, 3H), 1.51-0.70 (m, 36H). **^{13}C{^{1}H} NMR (125 MHz, $CDCl_3$)** δ 140.77, 134.03 (d, *J* = 13.75 Hz), 133.80 (d, *J* = 12.5 Hz), 133.60, 132.65 (d, *J* = 2.5 Hz), 132.46, 129.62 (d, *J* = 11.25 Hz), 129.49, 128.18, 127.66, 37.98 (br s, 1C), 35.73 (br s, 1C), 24.75 (br s, 4C). **^{19}F{^{1}H} NMR (470 MHz, CD_3CN)** δ –77.97. **^{31}P{^{1}H} NMR (202 MHz, $CDCl_3$)** δ 25.92, 23.84. Compound **6** is unstable and combustion microanalyses were consistently unsuccessful. In addition to the NMR spectra, we also obtained PXRD data from freshly prepared bulk solid; these powder data agreed with the simulated powder diffractogram generated from the crystal structure of **6**-linear (rhombohedral polymorph) (Supplementary Figure 48). Furthermore, high-resolution mass spectrometry data were collected to corroborate the proposed elemental composition.

Synthesis of tris(2,6-diisopropylphenyl)hydroxystibonium benzenesulfonate ([$Dipp_3SbOH$][O_3SPh]) (7a). A solution of benzenesulfonic acid (18 mg, 0.12 mmol) in DCM (4 mL) was added dropwise to a solution of **2a** (72 mg, 0.12 mmol) in DCM (3 mL). The colorless solution was stirred at room temperature for 30 min. The solvent level was reduced to 2 mL under reduced pressure and hexanes (15 mL) were added to precipitate a white powder. Yield: 69 mg (76%). Diffraction-quality crystals were grown from layering hexanes over a DCM solution of the compound. M.p. 230 °C. **Found:** C 64.57; H 7.53%, **Calc. for $C_{42}H_{57}O_4SSb$:** C, 64.70; H, 7.37%. **ESI-MS (*m/z*) [M–O_3SPh]$^+$** 621.27 (calc 621.3). **IR (KBr, cm^{-1})** ν_{SbO} 611 (s). **^{1}H NMR (500 MHz, $CDCl_3$)** δ 8.22 (br s, 1H), 7.56 (t, *J* = 7.7 Hz, 3H), 7.49 (d, *J* = 7.0 Hz, 2H), 7.40 (dd, *J* = 7.7, 1.1 Hz, 3H), 7.30 (dd, *J* = 7.7, 1.1 Hz, 3H), 7.22 (t, *J* = 7.2 Hz, 1H), 7.19-7.14 (m, 2H), 3.17 (hept, *J* = 6.4 Hz, 3H), 2.82 (hept, *J* = 6.4 Hz, 3H), 1.43 (d, *J* = 6.3 Hz, 9H), 1.21 (d, *J* = 6.6 Hz, 9H), 0.97 (d, *J* = 6.5 Hz, 9H), 0.86 (d, *J* = 6.4 Hz, 9H). **^{13}C{^{1}H} NMR (125 MHz, $CDCl_3$)** δ 156.30, 152.25, 146.71, 137.93, 133.79, 128.70, 128.62, 127.47, 126.49, 126.47, 38.92, 36.96, 26.41, 25.29, 24.83, 24.30.

Synthesis of tris(2,6-diisopropylphenyl)hydroxyarsonium benzenesulfonate ([$Dipp_3AsOH$][O_3SPh]) (7b). Benzenesulfonic acid (15 mg, 0.092 mmol) and **2b** (53 mg, 0.092 mmol) were dissolved in DCM (3 mL). The colorless solution was transferred under a layer of hexanes to grow colorless crystals. The product was washed with pentane (3 × 3 mL) and dried *in vacuo*. Yield: 44 mg (66%). Crystals suitable for X-ray

diffraction were grown similarly. M.p. 221 °C (decomp). **ESI-MS (*m/z*) [M–O_3SPh]$^+$** 575.3228 (calc 575.3228). **^{1}H NMR (500 MHz, $CDCl_3$)** δ 7.56-7.49 (m, *J* = 7.5 Hz, 5H), 7.38 (d, *J* = 7.7 Hz, 3H), 7.29 (d, *J* = 7.6 Hz, 3H), 7.25-7.18 (m, 3H), 3.10 (hept, 3H, *J* = 6.4 Hz), 3.09 (hept, 3H, *J* = 6.4 Hz), 1.62 (br s), 1.41 (d, *J* = 6.2 Hz, 9H), 1.17 (d, *J* = 6.4 Hz, 9H), 0.90 (d, *J* = 6.3 Hz, 9H), 0.81 (d, *J* = 6.1 Hz, 9H). **^{13}C{^{1}H} NMR (125 MHz, $CDCl_3$)** δ 154.73, 150.67, 136.27, 133.88, 129.31, 128.88, 127.61, 126.68, 126.60, 35.19, 33.76, 26.18, 25.36, 24.96, 23.94. The elemental composition of **7b** found from combustion microanalyses was outside of the acceptable deviation from the theoretical value. In addition to the NMR spectra, we also obtained PXRD data from freshly prepared bulk solid; these powder data agreed with the simulated powder diffractogram generated from the crystal structure of **7b** (Supplementary Figure 58). Furthermore, high-resolution mass spectrometry data were collected to corroborate the proposed elemental composition.

Synthesis of *cis*-hydroxyacetatotris(2,6-diisopropylphenyl)stiborane (*cis*-Sb(OH)(OAc)$Dipp_3$) (8). Glacial acetic acid (8.5 μL, 0.15 mmol) was added to a solution of **2a** (93 mg, 0.14 mmol) in DCM (4 mL). The colorless solution was stirred for 10 min at room temperature before being stripped of solvent under reduced pressure to yield the crude product as a white powder. The product was recrystallized from DCM/pentane at –20 °C. The solvent was then decanted, and the crystals were washed with cold pentane and dried under vacuum. Yield: 62 mg (61%). M.p. 188 °C (decomp). **Found:** C, 67.02; H, 7.97%, **Calc. for $C_{39}H_{58}O_3Sb$:** C, 67.24; H, 8.39%. **ESI-MS (*m/z*) [M–OAc]$^+$** 621.27 (calc 621.3). **IR (KBr, cm^{-1})** ν_{SbO} 649 (s). **^{1}H NMR (500 MHz, $CDCl_3$)** δ 7.44 (t, *J* = 7.7 Hz, 3H), 7.34 (d, *J* = 7.4 Hz, 3H), 7.20 (d, *J* = 7.4 Hz, 3H), 5.73 (br s, 3H), 3.97 (br sept, *J* = 6.05 Hz, 3H), 2.94 (br sept, *J* = 5.8 Hz, 3H), 1.95 (s, 3H), 1.40 (br s, 9H), 1.19 (br s, 9H), 0.96 (br s, 9H), 0.76 (br s, 9H). **^{13}C{^{1}H} NMR (125 MHz, $CDCl_3$)** δ 175.04, 156.67, 152.03, 141.32, 132.10, 127.36, 125.62, 37.59, 34.45, 26.78, 25.11, 24.73, 24.62, 22.80.

Synthesis of *trans*-difluorotris(2,6-diisopropylphenyl)stiborane (*trans*-$Dipp_3SbF_2$) (9). BF_3·OEt_2 (19 μL, 0.15 mmol) was added to a solution of **2a** (95 mg, 0.15 mmol) in DCM (2 mL) at –78 °C resulting in rapid precipitation of a white solid. The reaction mixture was allowed to warm to room temperature before being filtered through a Celite pad. The solvent was removed to yield a white powder. The crude product was recrystallized from OEt_2/MeCN. Yield: 57 mg, 59%. Crystals suitable for X-ray diffraction were grown by slow evaporation of MeCN. M.p. 279 °C. **Found:** C, 67.07; H, 8.49%, **Calc. for $C_{36}H_{51}F_2Sb$:** C, 67.19; H, 7.99%. **IR (KBr, cm^{-1})** ν_{SbF} 528 (s). **^{1}H NMR (500 MHz, $CDCl_3$)** δ 7.42 (t, *J* = 7.6 Hz, 3H), 7.30 (d, *J* = 7.6 Hz, 6H), 3.16 (hept, *J* = 6.3 Hz, 6H), 1.18 (d, *J* = 6.1 Hz, 18H), 1.09 (d, *J* = 6.5 Hz, 18H). **^{13}C{^{1}H} NMR (125 MHz, $CDCl_3$)** δ 154.56, 130.93, 125.52, 35.48, 27.13, 24.53. **^{19}F{^{1}H} NMR (470 MHz, $CDCl_3$)** δ –74.35 (s).

Conversion of 2a to 1a by phenylsilane. Phenylsilane (20 μL, 0.16 mmol) was added to a solution of **2a** (98 mg, 0.16 mmol) in toluene (5 mL). The colorless solution was heated to 50 °C for 1.5 h. The reaction mixture was then stripped of solvent to yield a white solid. The crude product was dissolved in hexanes and purified by column chromatography to obtain **1a**. Yield: 76 mg (80%). NMR spectra of the product match those reported above for **1a**. An NMR-scale reaction was also performed to demonstrate > 93% conversion of **2a** to **1a** by one equivalent of phenylsilane at 50 °C for 1 h in d_8-toluene (Supplementary Figure 71). No reaction was observed when **2b** or **2c** were mixed with phenylsilane under similar conditions (Supplementary Figures 72 and 73).

X-ray absorption spectroscopy. The Sb K-edge XAS data were collected at beamline 7-3 at the Stanford Synchrotron Radiation Lightsource (SSRL) using a Si(220) double crystal monochromator. Solid samples were placed into aluminum sample holders sealed with Kapton adhesive tape. Samples were flash frozen in liquid nitrogen and maintained at 10 K using a He-flow cryostat (Oxford instruments, Abingdon, UK). To gain access to the high energies of the Sb K-edge, the beamline was operated in mirrorless mode,

employing a 0.25 mm vertical aperture so as to give adequate energy resolution. The X-ray absorption spectrum was measured using N_2-filled gas-ionization chambers employing a sweeping voltage of 1.8 kV and operating above the recombination region. The incident beam energy was calibrated to the lowest-energy K-edge inflection from Sb foil (assumed to be 30488.0 eV) with a photoionization threshold (at which $k = 0.0$ Å^{-1}) assumed to be 30510 eV. XAS data reduction and analysis were performed using the EXAFSPAK suite of programs[6] and employing the program FEFF (v8.5)[7] for ab-initio phase and amplitude functions to fit the EXAFS. All fitting was performed in *k*-space using the data as shown in Figure 3 of the main paper. The goodness of fit parameter *F* is defined as $F = [\Sigma k^6(\chi_{calc}(k) - \chi_{exp}(k))^2 / \Sigma k^6(\chi_{exp}(k))^2]^{0.5}$ in which $\chi_{exp}(k)$ and $\chi_{calc}(k)$ are the experimental and calculated EXAFS, respectively, and the summations are over all points included within the fitted *k*-range, which in all cases was 1-18.1 Å^{-1}. Values of *F* close to 0.3 are typically considered adequate fits, while 0.4 and above are poor fits. Only the first-shell or Sb-Sb contributions (in the case of **A**) to the EXAFS discussed are shown in the Supplementary Table 7 but, in all cases, the outer C shells were approximated in a similar manner to that reported by Ferreira *et al.* using a rigid benzene ring, assuming a C–C bond-length of 1.381 Å, with multiple scattering included, which was found to be significant (>2% of total amplitude) only for third- and fourth-shell C atoms. Pendant groups on the phenyl rings were approximated using single scattering analysis with interatomic distances grouped into clusters when Sb···C distances were less than the EXAFS resolution of $\pi/2k_{max}$.[8] We note that for the EXAFS fitting of **2a**, fitting a C atom in the place O gave a worse goodness-of-fit index ($F = 0.335$ for Sb–C vs. 0.319 for Sb–O) and a physically unreasonable Debye-Waller factor (σ^2 of 0.0010 Å^2 for Sb–C vs. 0.0021 Å^2 for Sb–O).

Computational experiments. In computational experiments, **5** denotes $(Dipp_3SbO)_2Ag^+$, **6**-bent denotes $Dipp_3SbOAuPPh_3^+$ as it occurs in the $P\overline{1}$ polymorph and **6**-linear refers to $Dipp_3SbOAuPPh_3^+$ as it occurs in the $R\overline{3}$ polymorph. Geometry optimization and frequency DFT calculations were performed using ORCA 4.2.1 or ORCA 5.0.1.[9] Geometry optimization and frequency calculations were performed on **2c**, **2b**, **2a**, **A**, **B**, and **8** at the PBE0/def2-TZVPP level of theory with the RIJCOSX approximation and def2/J auxiliary basis set.[10-14] The TIGHTOPT keyword was used to tighten the optimization convergence criteria for **2c**, **2b**, **A**, and the VERYTIGHTOPT keyword was used for **B**. Geometry optimization and frequency calculations were performed on **5** at the BP86/def2-SVP level of theory with the RI approximation and def2/J auxiliary basis set. Frequency calculations on **2c**, **2b**, and **2a** found small imaginary modes at –13.97 cm^{-1}, –11.37 cm^{-1}, and –11.38 cm^{-1} respectively that arise from an aryl substituent vibration. The frequency calculation of **B** found an imaginary mode at –30.07 cm^{-1} that arises from a rotating *para*-methyl group. The calculated IR vibrational frequencies of **2c**, **2b**, and **2a** were corrected with a 0.96 scalar factor.[15] Single point energy calculations were performed using ORCA 5.0.0 or ORCA 5.0.1 on the optimized structures using the PBE0 hybrid functional and old-DKH-TZVPP all-electron basis set using the RIJCOSX approximation and SARC/J auxiliary basis set.[16-19] Single point energy calculations on **7b**, **7a**, and **3** were performed on the coordinates obtained from Hirschfeld atom refinement at the same level of theory. Single point energy calculations on **4**, **6**-bent, and **6**-linear were performed on the crystallographic coordinates after normalizing C–H bond distances to 1.089 Å at the same level of theory. Gold atoms were treated with the ANO-RCC-TZP basis set. The effects of relativity were introduced using the second order Douglass-Kroll-Hess formalism (DKH).[20] Single point energy calculations generated wavefunctions that were subject to topological and Natural Bond Orbital (NBO) analyses. In the case of **5**, NBO analysis was performed using the BP86 pure functional and def2-SVP basis set. Canonical molecular orbitals were visualized using Avogadro.[21] Topological analysis of the electron density was performed in MultiWFN (version 3.7).[22] Bond paths were visualized in MultiWFN (version 3.7). The values of the real space functions ρ, $\nabla^2\rho$, and ε along interatomic vectors were visualized using R (version 4.0.2) through RStudio (version 1.3.1073). The following R packages were used for analysis and visualization: ggplot2, tidyverse, gridExtra, ggtext, scales, ggbreak, and grid.[23] NBO analysis was performed using the NBO program (version 7.0.7).[24] For **2a**-**c**, the $DEL keylist

was used to calculate the energy of deletion by eliminating all non-Lewis delocalizations of electron density from the oxo unit to the rest of the molecule and vice versa. The energy of deletion of the strongest H-bond in **2a** was calculated by eliminating all delocalizations from the O atom to the C–H fragment and vice versa to be 7.427 kcal/mol. Pre-orthogonalized natural bond orbitals were visualized in JMOL (version 14.31.18). Electrostatic surface potentials, deformation densities, and non-covalent interactions (NCI) were calculated using MultiWFN and the results were visualized in VMD.[25] Energy decomposition analysis was performed using Gaussian 09[26] to perform single point energy calculations on the O atom and $Dipp_3Pn$ fragments using coordinates from the optimized $Dipp_3PnO$ structures (Pn = P, As, Sb). ΔE_{tot} was then obtained by subtracting the energies of the $Dipp_3Pn$ and O fragments from that of the parent $Dipp_3PnO$ molecule. MultiWFN was then used to generate an input file for $Dipp_3PnO$ with an initial guess for the combined O and $Dipp_3Pn$ fragments with no orbital interaction. The energy of the initial guess wavefunction was then subtracted from the final converged energy to obtain ΔE_{orb}. Force constants for the pnictoryl bonds were obtained by diagonalization of the Hessian matrix using ORCA 5.0.1 and by fitting a quadratic equation to the energies obtained from a rigid surface scan of the potential energy surface of the Pn–O stretch/contraction, which was also performed with ORCA 5.0.1.

X-ray crystallography: Independent Atom Model (IAM). Crystals of **1a**, **1b**, **1c**, **2a**, **2b**, **2c**, **3**, **4**·$(CHCl_3)_2$, **5**·OEt_2, **6**, **7a**, **7b**, **8**, and **9** were grown as described above, selected under a microscope, loaded onto a MiTeGen polyimide sample loop using Paratone-N, and mounted onto a Rigaku XtaLAB Synergy-S single crystal diffractometer. Each crystal was cooled to 100 K under a stream of nitrogen. Diffraction of Mo Kα or Cu Kα radiation from a PhotonJet-S microfocus source was detected using a HyPix6000HE hybrid photon counting detector. Screening, indexing, data collection, and data processing were performed with CrysAlisPro.[27] The structures were solved using SHELXT and refined using SHELXL following established strategies.[28-30] All non-H atoms were refined anisotropically. Carbon-bound H atoms were placed at calculated positions and refined with a riding model and coupled isotropic displacement parameters (1.2 × U_{eq} for aryl groups and 1.5 × U_{eq} for methyl groups). In the cases of **7b** and **8**, oxygen-bound H atoms were located in the difference Fourier synthesis; their positional and isotropic displacement parameters were refined freely. In the case of **1a**, the isopropyl groups are disordered across two positions. The extensive disorder and high symmetry of the Laue group lowered the data-to-parameter ratio to 5.99 (B-level CheckCIF alert), which was addressed using similarity, distance, and rigid bond restraints. The F_o–F_c map featured a residual electron density maximum of 2.98 e Å^{-3} located 1.51 Å from the Sb center (B-level CheckCIF alert). This maximum is interpreted as artefactual, consistent with its location on a special position, the presence of the heavy Sb atom on a special position, and the extensive disorder in the structure. In the case of **1b**, there is a similar electron density maximum (B-level CheckCIF alert) near the heaviest atom which is believed to arise for the same reason. In the case of **1c**, which crystallizes in the Sohncke space group *R*3, there is low coverage of Friedel pairs (B-level CheckCIF alert). The high symmetry of the Laue group and long wavelength (Cu) used for collection decreased the coverage of Friedel pairs. We note that the crystal is a racemic twin of the two different conformations of the compound, which simply arise from the differential canting of the aryl rings; these conformations rapidly convert in solution and the molecule does not feature fixed molecular chirality. In the case of **5**·OEt_2, the ether molecule was disordered across two positions and was modelled using similarity, distance, and rigid bond restraints. In the case of the rhombohedral polymorph of **6**, the triflate counterion was disordered across two general positions and the $\overline{3}$ special position.

X-ray crystallography: Hirshfeld atom refinement (HAR). The IAM results for **2a**, **2b**, **2c**, **3**, **7a**, **7b**, and **8** were used as input for the *NoSpherA2* implementation of HAR in *Olex2* (version 1.3 for **2a** and **3**, and version 1.5 for **2b**, **2c**, **7a**, **7b**, and **8**).[31] The quantum chemistry calculations were performed by ORCA (version 4.2.1 for **2a**, version 5.0.0 for **3**, and version 5.0.1 for **2b**, **2c**, **7a**, **7b**, and **8**).[9, 32] A wavefunction

was calculated with high integration accuracy and tight SCF convergence criteria using the PBE0 hybrid functional and the x2c-TZVPP all-electron relativistically contracted basis set.[10-12, 33] The effects of relativity were introduced using the second-order Douglass-Kroll-Hess formalism.[20] Least-squares crystallographic refinement was carried out with the *olex2.refine* engine.[34] The ADPs and positions of all atoms, including H atoms, were freely refined using the aspherical atomic form factors obtained from Hirshfeld stockholder partitioning of the theoretical electron density obtained from the computed wavefunction.[35] The newly refined atomic coordinates were used as the input for a new density functional theory (DFT) calculation, from which new aspherical form factors were obtained. This procedure was iterated until it had converged. In the cases **2b**, **2c**, **7a**, and **8**, a preliminary round of HAR was performed on the IAMs using the PBE functional and x2c-SVP basis set with low integration accuracy and sloppy SCF convergence criteria. The resulting preliminary model contained non-positive definite H-atom ADPs. Similarity and rigid bond restraints were applied to H-atom ADPs and the preliminary HAR model was used as an input for the higher-level iterative HAR described above. In the case of **7b**, H atoms were refined freely and isotropically. The structure of **2c** featured a low data-to-parameter ratio (A-level CheckCIF alert), the effects of which were mitigated by introducing extensive distance similarity restraints between the two components of the disorder as describe above for **1c**.

Powder X-ray diffraction (PXRD). Bulk samples of **1c**, **3**, **4**·$(CHCl_3)_2$, **5**·OEt_2, **6**, and **7b** were ground using an agate mortar and pestle. The fine white powders were each loaded onto a MiTeGen polyimide sample loop using Paratone-N and mounted onto a Rigaku XtaLAB Synergy-S single-crystal diffractometer. The powder was cooled to 100 K under a stream of nitrogen. The diffraction of Cu Kα radiation was collected while the sample underwent a Gandolfi scan. Data collection and processing were performed using CrysAlisPro. Simulated PXRD diffractograms were generated from the crystal structures of **1c**, **3**, **4**·$(CHCl_3)_2$, **5**·OEt_2, **6**-linear (rhombohedral polymorph), and **7b** using Mercury and compared to the experimentally determined diffractograms.

References

1. Warsitz, M., Doye, S. Linear Hydroaminoalkylation Products from Alkyl-Substituted Alkenes. *Chem.–Eur. J.* 2020, **26**(66)**:** 15121-15125.

2. Li, K., Weber, A. E., Tseng, L., Malcolmson, S. J. Diastereoselective and Enantiospecific Synthesis of 1,3-Diamines via 2-Azaallyl Anion Benzylic Ring-Opening of Aziridines. *Org. Lett.* 2017, **19**(16)**:** 4239-4242.

3. Bordner, J., Doak, G. O., Everett, T. S. Crystal structure of 2,2,4,4-tetrahydro-2,2,2,4,4,4-hexaphenyl-1,3,2,4-dioxadistibetane (triphenylstibene oxide dimer) and related compounds. *J. Am. Chem. Soc.* 1986, **108**(14)**:** 4206-4213.

4. Wenger, J. S., Johnstone, T. C. Unsupported monomeric stibine oxides (R_3SbO) remain undiscovered. *Chem. Commun.* 2021, **57:** 3484-3487.

5. Huber, F., Westhoff, T., Preut, H. Tris(2,4,6-trimethylphenyl)antimony dihydroxide; synthesis and reaction with sulfonic acids RSO_3H (R = C_6H_5, CF_3). Crystal structure of $[2,4,6\text{-}(CH_3)_3C_6H_2]_3SbO \cdot HO_3SC_6H_5$. *J. Organomet. Chem.* 1987, **323**(2)**:** 173-180.

6. George, G. N., Pickering, I. J. EXAFSPAK: A suite of computer programs for analysis of X-ray absorption spectra. Stanford Synchrotron Radiation Laboratory, Stanford, CA, USA; 1995.

7. Rehr, J. J., Albers, R. C. Theoretical approaches to x-ray absorption fine structure. *Rev. Mod. Phys.* 2000, **72**(3)**:** 621-654.

8. Ferreira, G. C., Franco, R., Mangravita, A., George, G. N. Unraveling the Substrate−Metal Binding Site of Ferrochelatase: An X-ray Absorption Spectroscopic Study. *Biochemistry* 2002, **41**(15)**:** 4809-4818.

9. Neese, F. The ORCA program package. *Wiley Interdiscip. Rev.-Comput. Mol. Sci* 2012, **2:** 73-78.

10. Perdew, J. P., Burke, K., Ernzerhof, M. Generalized Gradient Approximation Made Simple. *Phys. Rev. Lett.* 1996, **77**(18)**:** 3865-3868.

11. Perdew, J. P., Ernzerhof, M., Burke, K. Rationale for mixing exact exchange with density functional approximations. *J. Chem. Phys.* 1996, **105**(22)**:** 9982-9985.

12. Weigend, F., Ahlrichs, R. Balanced basis sets of split valence, triple zeta valence and quadruple zeta valence quality for H to Rn: Design and assessment of accuracy. *Phys. Chem. Chem. Phys.* 2005, **7**(18)**:** 3297-3305.

13. Weigend, F. Accurate Coulomb-fitting basis sets for H to Rn. *Phys. Chem. Chem. Phys.* 2006, **8**(9)**:** 1057.

14. Neese, F., Wennmohs, F., Hansen, A., Becker, U. Efficient, approximate and parallel Hartree–Fock and hybrid DFT calculations. A 'chain-of-spheres' algorithm for the Hartree–Fock exchange. *Chem. Phys.* 2009, **356**(1-3)**:** 98-109.

15. NIST, NIST Computational Chemistry Comparison and Benchmark Database; NIST Standard Reference Database Number 101, Release 21 (August 2020). <http://cccbdb.nist.gov/>.

16. Pantazis, D. A., Chen, X.-Y., Landis, C. R., Neese, F. All-Electron Scalar Relativistic Basis Sets for Third-Row Transition Metal Atoms. *J. Chem. Theory Comput.* 2008, **4**(6)**:** 908-919.

17. Pantazis, D. A., Neese, F. All-Electron Scalar Relativistic Basis Sets for the Lanthanides. *J. Chem. Theory Comput.* 2009, **5**(9)**:** 2229-2238.

18. Pantazis, D. A., Neese, F. All-Electron Scalar Relativistic Basis Sets for the Actinides. *J. Chem. Theory Comput.* 2011, **7**(3)**:** 677-684.

19. Pantazis, D. A., Neese, F. All-electron scalar relativistic basis sets for the 6p elements. *Theor. Chem. Acc.* 2012, **131**(11)**:** 1292.

20. Wolf, A., Reiher, M., Hess, B. A. The generalized Douglas–Kroll transformation. *J. Chem. Phys.* 2002, **117**(20)**:** 9215-9226.

21. Hanwell, M. D., Curtis, D. E., Lonie, D. C., Vandermeersch, T., Zurek, E., Hutchison, G. R. Avogadro: an advanced semantic chemical editor, visualization, and analysis platform. *J. Cheminformatics* 2012, **4**(1)**:** 17.

22. Lu, T., Chen, F. Multiwfn: A multifunctional wavefunction analyzer. *J. Comput. Chem.* 2012, **33**(5)**:** 580-592.

23. Xu, S., Chen, M., Feng, T., Zhan, L., Zhou, L., Yu, G. Use ggbreak to Effectively Utilize Plotting Space to Deal With Large Datasets and Outliers. *Front. Genet.* 2021, **12:** 774846.

24. Glendening, E. D., Landis, C. R., Weinhold, F. NBO 7.0 : New vistas in localized and delocalized chemical bonding theory. *J. Comput. Chem.* 2019, **40**(25)**:** 2234-2241.

25. Humphrey, W., Dalke, A., Schulten, K. VMD: Visual molecular dynamics. *J. Mol. Graph.* 1996, **14**(1)**:** 33-38.

26. M. J. Frisch, G. W. T., H. B. Schlegel, G. E. Scuseria, M. A. Robb, J. R. Cheeseman, G. Scalmani, V. Barone, G. A. Petersson, H. Nakatsuji, X. Li, M. Caricato, A. Marenich, J. Bloino, B. G. Janesko, R. Gomperts, B. Mennucci, H. P. Hratchian, J. V. Ortiz, A. F. Izmaylov, J. L. Sonnenberg, D. Williams-Young, F. Ding, F. Lipparini, F. Egidi, J. Goings, B. Peng, A. Petrone, T. Henderson, D. Ranasinghe, V. G. Zakrzewski, J. Gao, N. Rega, G. Zheng, W. Liang, M. Hada, M. Ehara, K. Toyota, R. Fukuda, J. Hasegawa, M. Ishida, T. Nakajima, Y. Honda, O. Kitao, H. Nakai, T. Vreven, K. Throssell, J. A. Montgomery, Jr., J. E. Peralta, F. Ogliaro, M. Bearpark, J. J. Heyd, E. Brothers, K. N. Kudin, V. N. Staroverov, T. Keith, R. Kobayashi, J. Normand, K. Raghavachari, A. Rendell, J. C. Burant, S. S. Iyengar, J. Tomasi, M. Cossi, J. M. Millam, M. Klene, C. Adamo, R. Cammi, J. W. Ochterski, R. L. Martin, K. Morokuma, O. Farkas, J. B. Foresman, and D. J. Fox. Gaussian 09, Revision A.02. Wallingford CT: Gaussian Inc; 2016.

27. Rigaku Oxford Diffraction. *CrysAlis^{Pro}* 2020.

28. Sheldrick, G. M. SHELXT– Integrated space-group and crystal-structure determination. *Acta Crystallogr. Sect. A* 2015, **71**(1)**:** 3-8.

29. Sheldrick, G. M. Crystal structure refinement withSHELXL. *Acta Crystallogr. Sect. C* 2015, **71**(1)**:** 3-8.

30. Müller, P. Practical suggestions for better crystal structures. *Crystallogr. Rev.* 2009, **15**(1)**:** 57-83.

31. Kleemiss, F., Dolomanov, O. V., Bodensteiner, M., Peyerimhoff, N., Midgley, L., Bourhis, L. J.*, et al.* Accurate crystal structures and chemical properties from NoSpherA2. *Chem. Sci.* 2021, **12:** 1675-1692.

32. Neese, F. *WIREs Comput. Mol. Sci* 2018, **8:** e1327.

33. Pollak, P., Weigend, F. Segmented Contracted Error-Consistent Basis Sets of Double- and Triple-ζ Valence Quality for One- and Two-Component Relativistic All-Electron Calculations. *J. Chem. Theory Comput.* 2017, **13**(8)**:** 3696-3705.

34. Dolomanov, O. V., Bourhis, L. J., Gildea, R. J., Howard, J. A. K., Puschmann, H. OLEX2: a complete structure solution, refinement and analysis program. *J. Appl. Crystallogr.* 2009, **42**(2)**:** 339-341.

35. Hirshfeld, F. L. Bonded-atom fragments for describing molecular charge densities. *Theor. Chim. Acta* 1977, **44**(2)**:** 129-138.

36. Pyykkö, P. Additive Covalent Radii for Single-, Double-, and Triple-Bonded Molecules and Tetrahedrally Bonded Crystals: A Summary. *J. Phys. Chem. A* 2015, **119**(11)**:** 2326-2337.

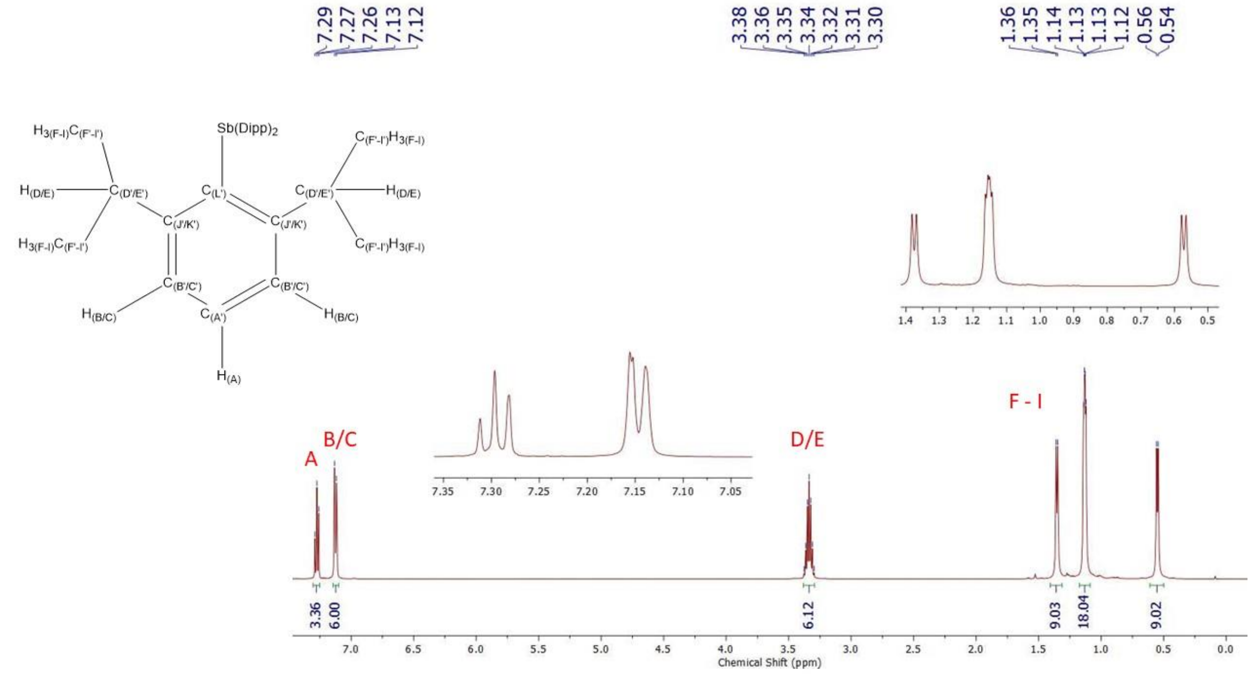

Figure 1. ^{1}H NMR spectrum ($CDCl_3$, 500 MHz) of **1a** at room temperature.

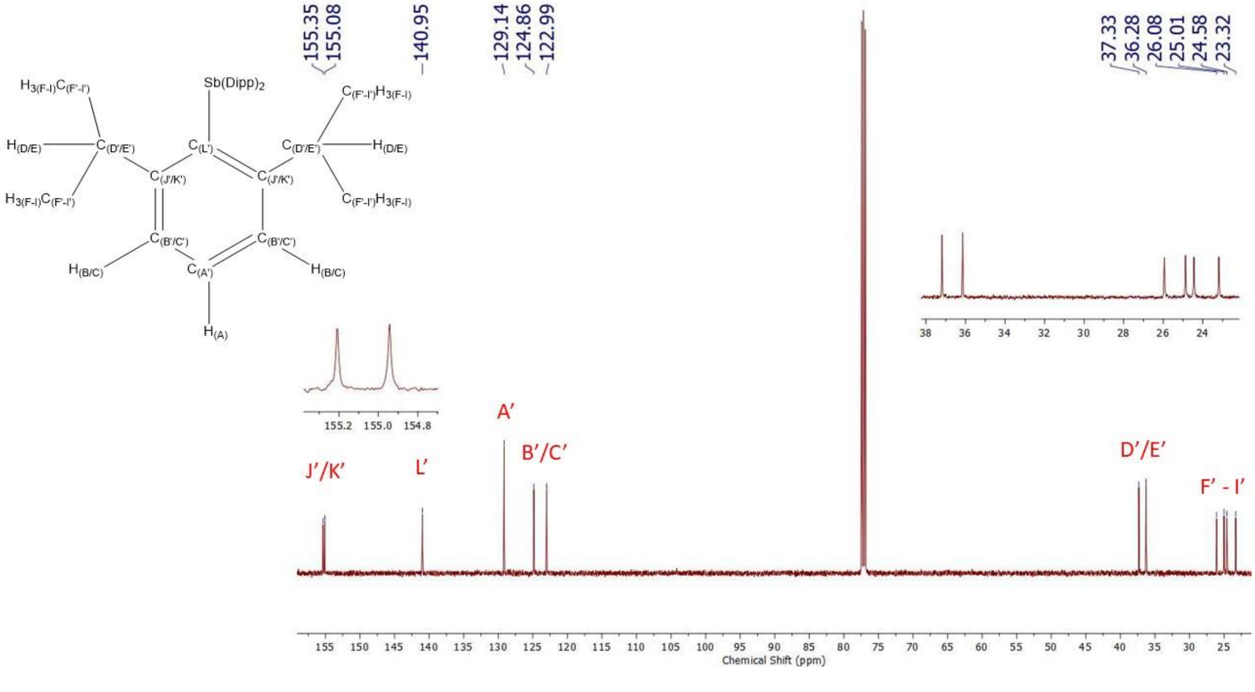

Figure 2. $^{13}C\{^{1}H\}$ NMR spectrum ($CDCl_3$, 125 MHz) of **1a** at room temperature. Peaks are assigned based on HSQC and HMBC experiments (*vide infra*).

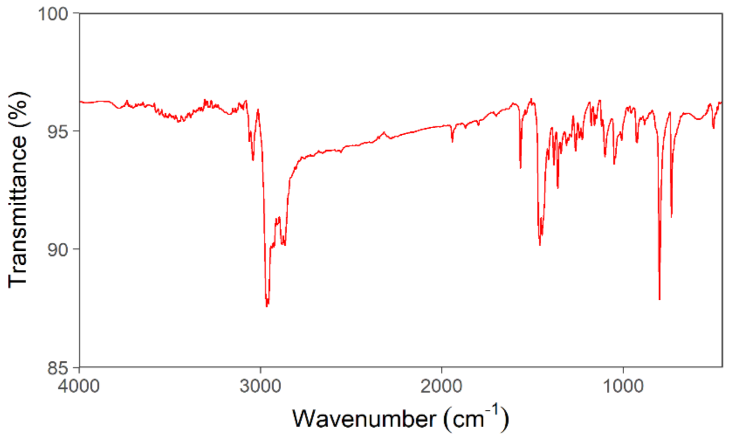

Figure 3. Experimental IR spectrum (KBr pellet) of **1a**.

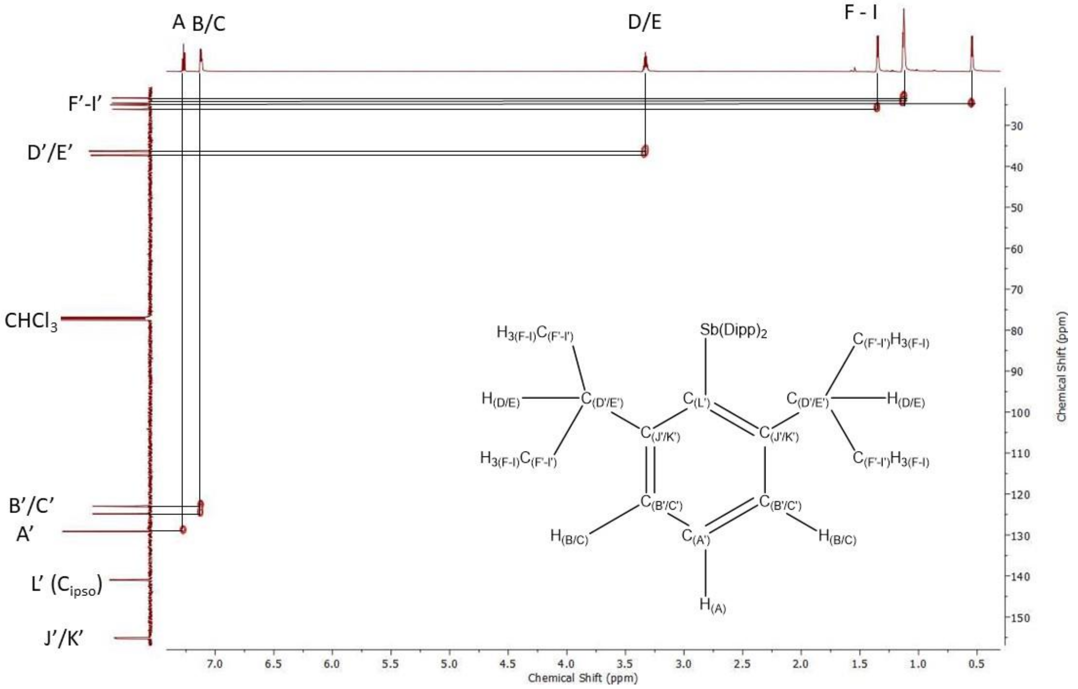

Figure 4. ^{1}H-^{13}C HSQC (800 MHz, $CDCl_3$) spectrum of **1a** at room temperature.

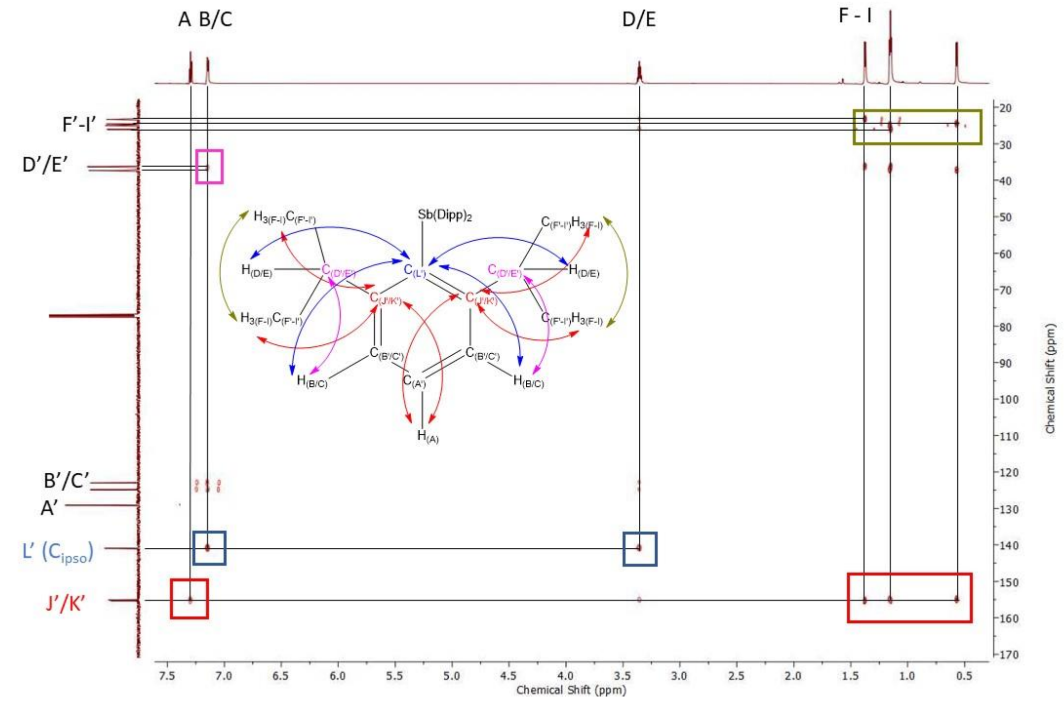

Figure 5. ^{1}H-^{13}C HMBC (800 MHz, $CDCl_3$) spectrum of **1a** at room temperature.

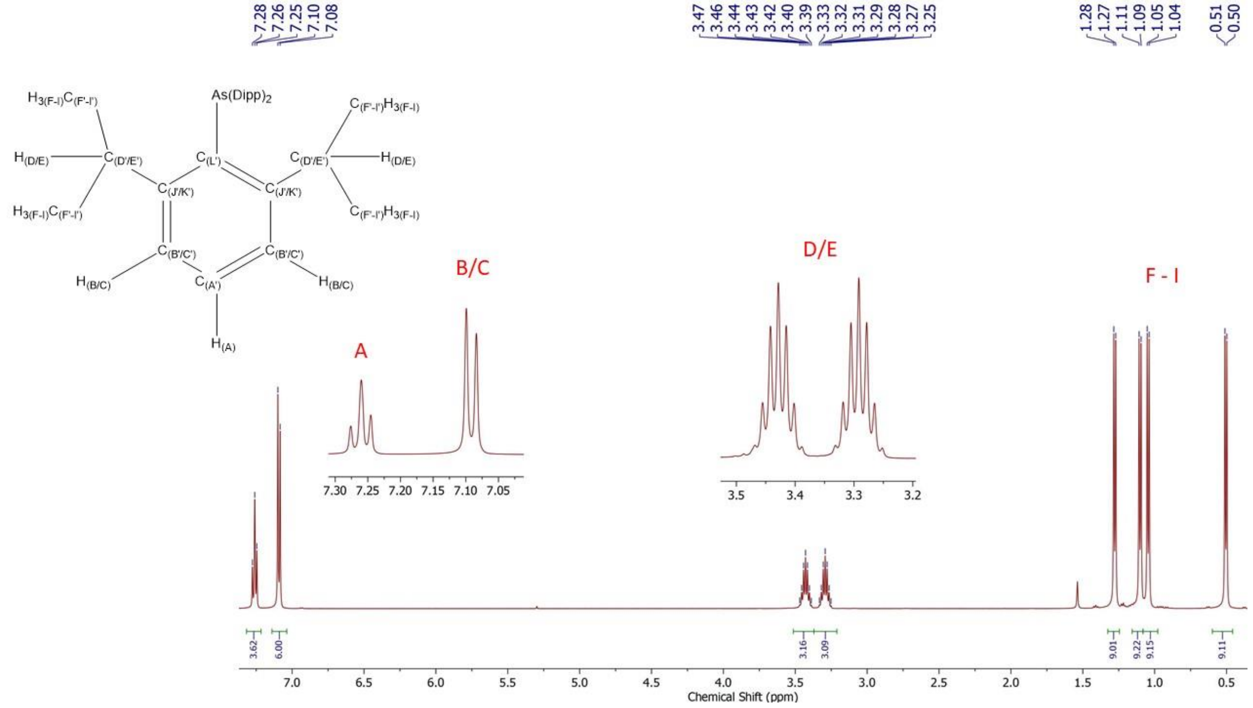

Figure 6. ^{1}H NMR spectrum ($CDCl_3$, 500 MHz) of **1b** at room temperature.

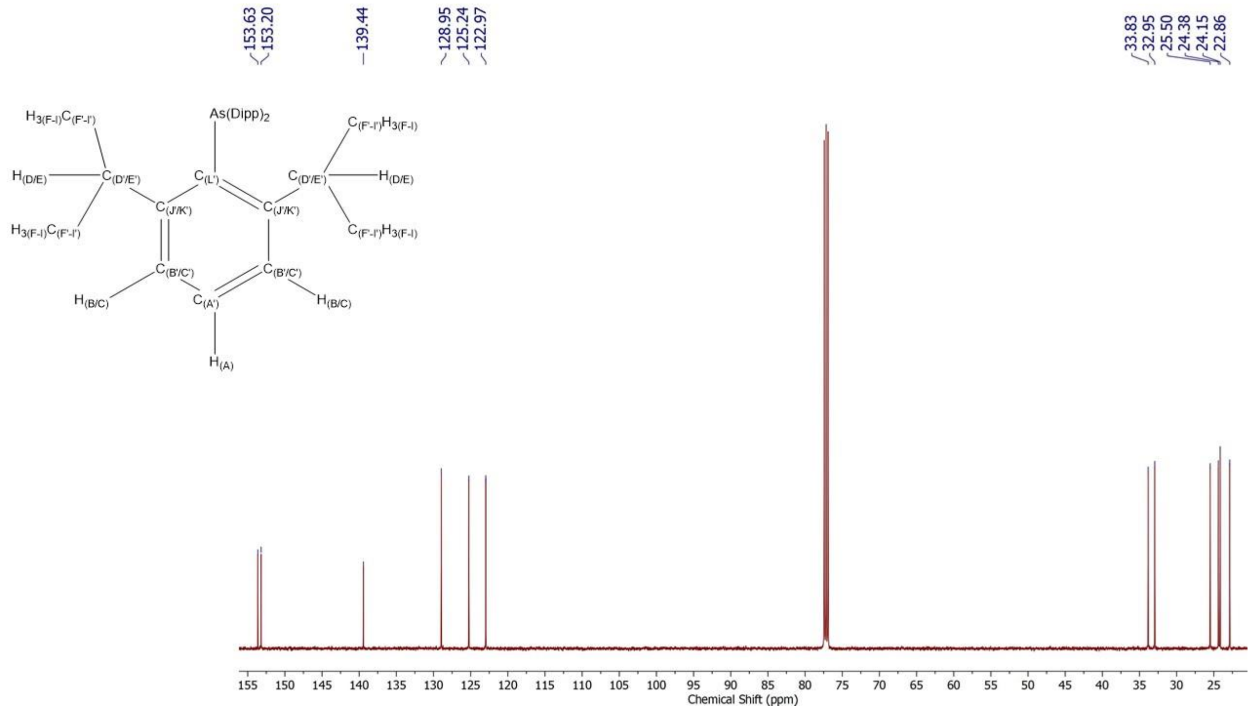

Figure 7. $^{13}C\{^1H\}$ NMR spectrum ($CDCl_3$, 125 MHz) of **1b** at room temperature.

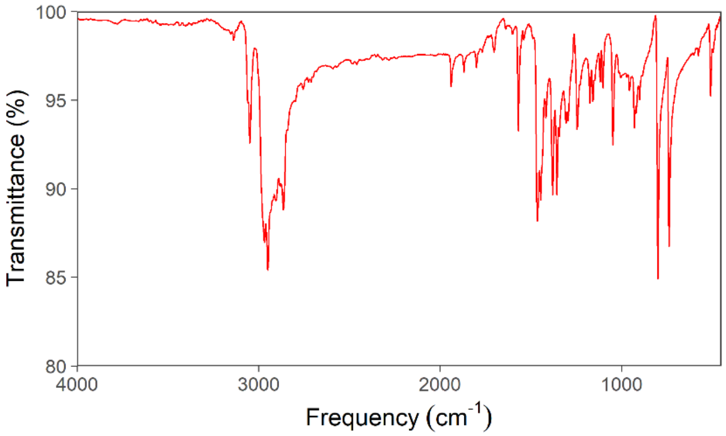

Figure 8. Experimental IR spectrum (KBr pellet) of **1b**.

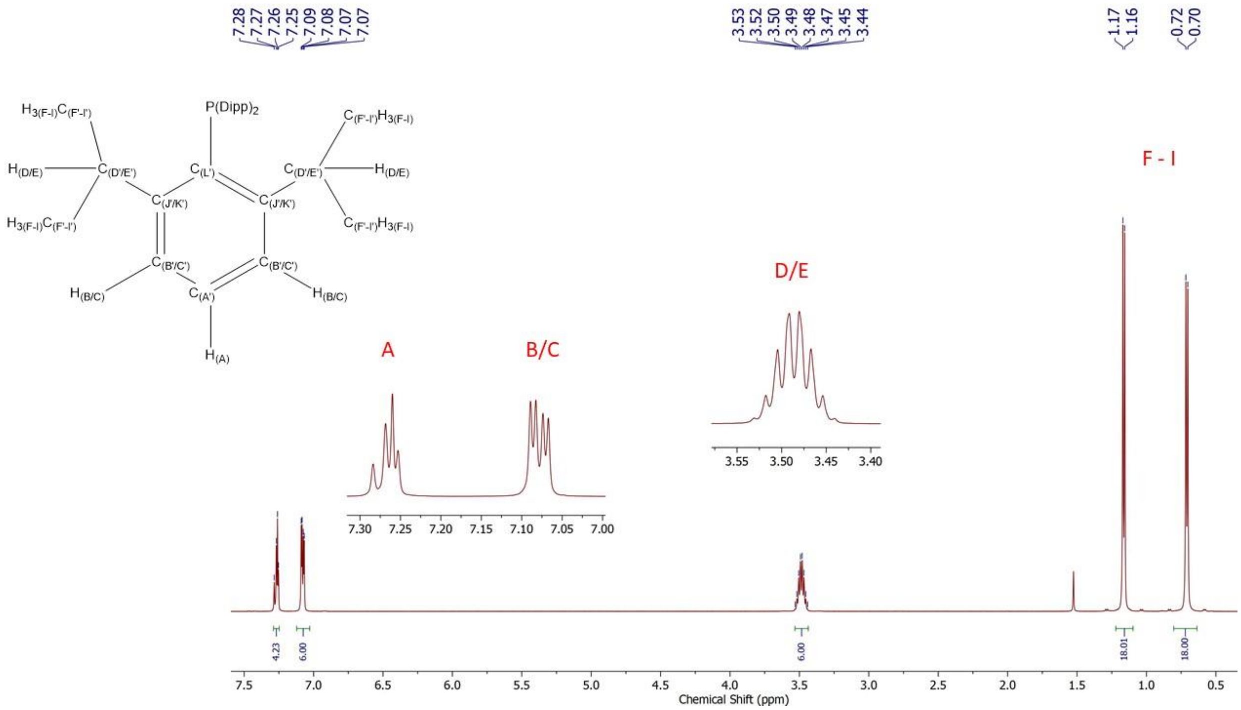

Figure 9. ^{1}H NMR spectrum ($CDCl_3$, 500 MHz) of **1c** at room temperature.

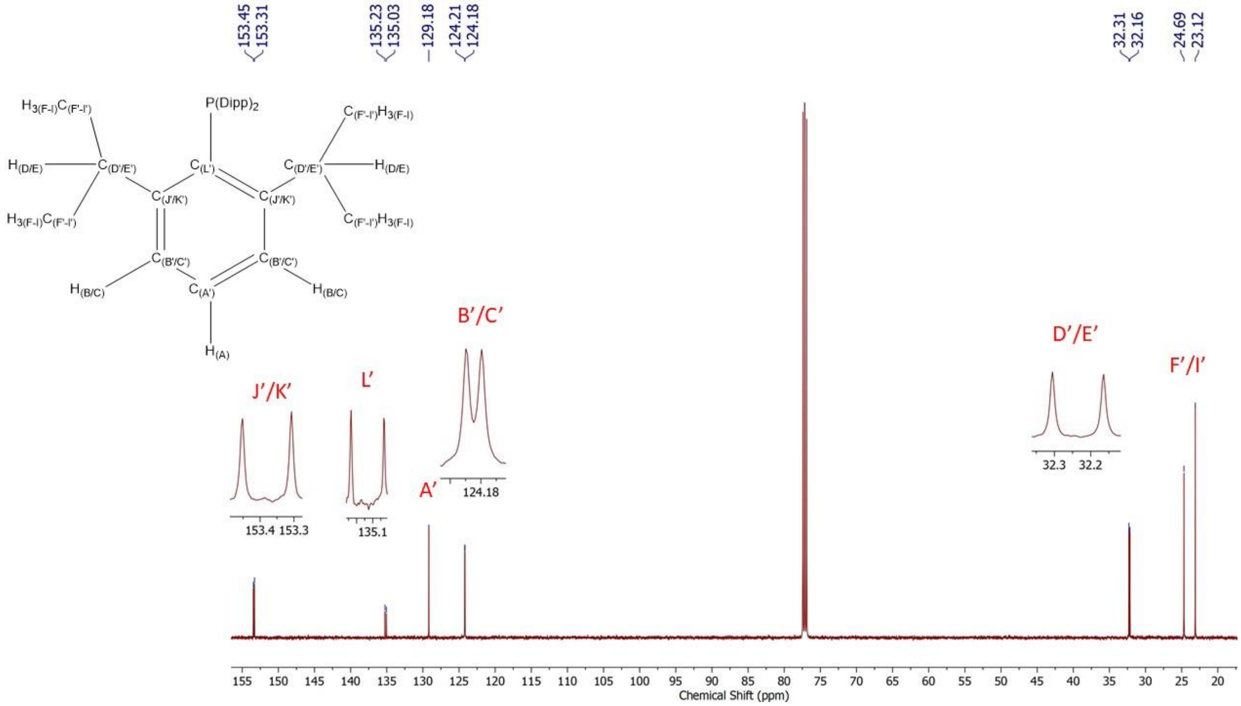

Figure 10. $^{13}C\{^{1}H\}$ NMR spectrum ($CDCl_3$, 125 MHz) of **1c** at room temperature.

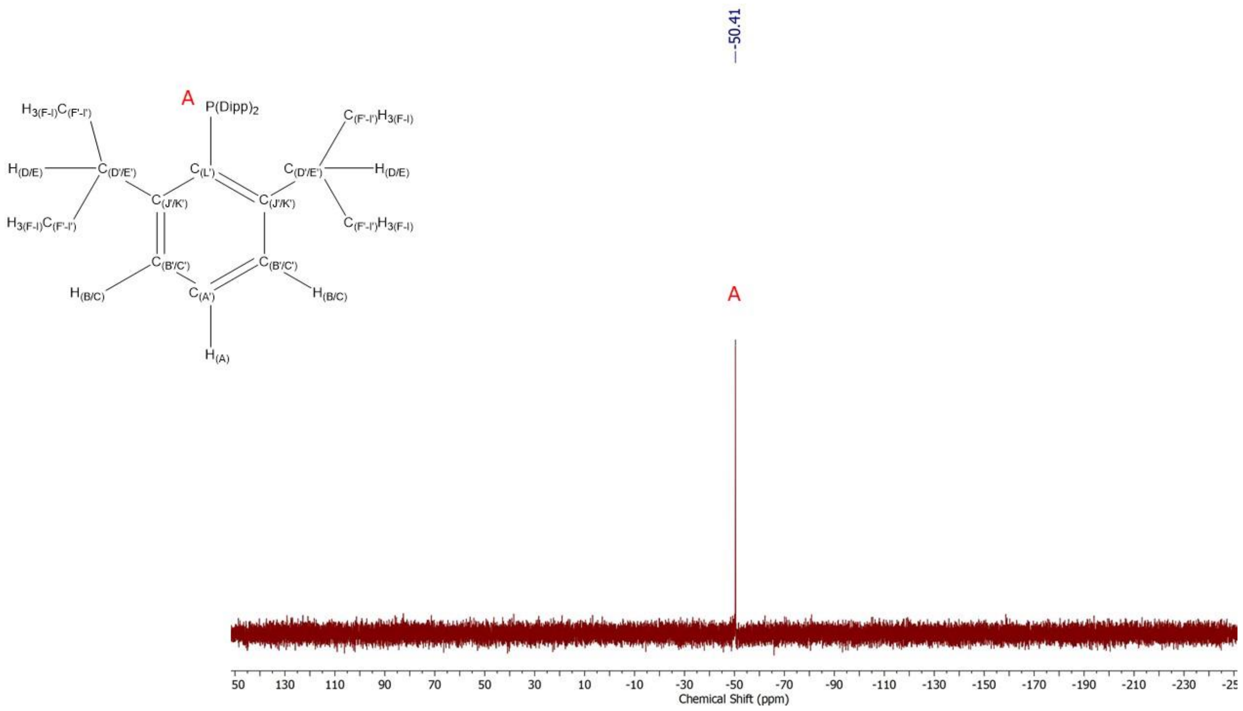

Figure 11. $^{31}P\{^{1}H\}$ NMR spectrum ($CDCl_3$, 202 MHz) of **1c** at room temperature.

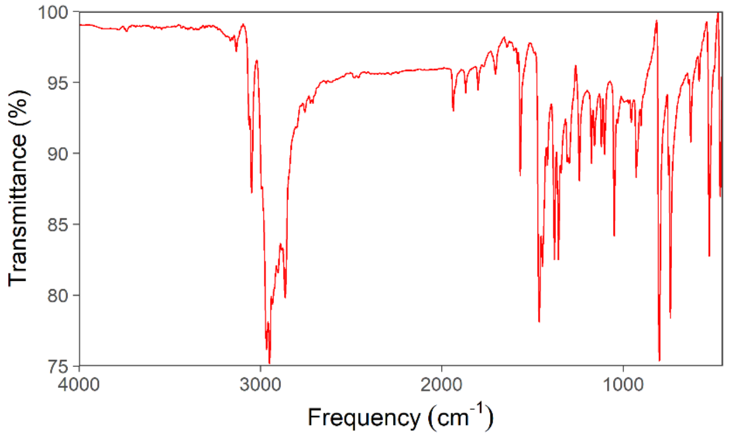

Figure 12. Experimental IR spectrum (KBr pellet) of **1c**.

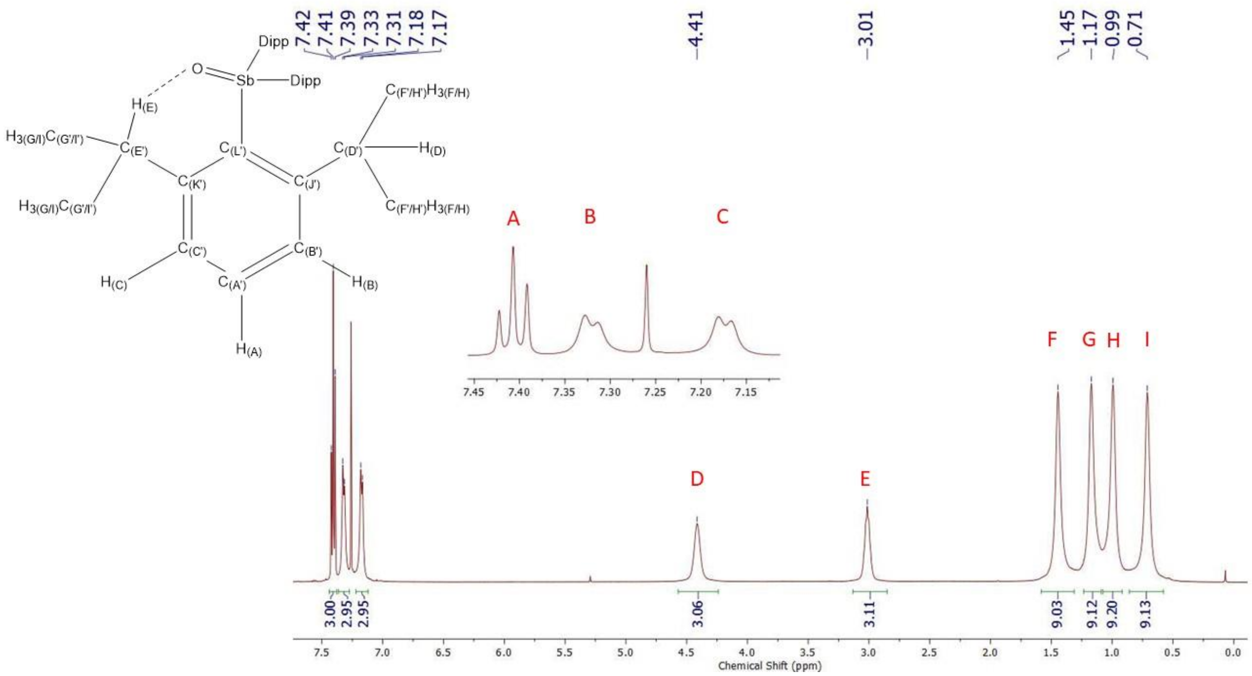

Figure 13. ^{1}H NMR spectrum ($CDCl_3$, 500 MHz) of **2a** at room temperature. Peaks are assigned based on HSQC and HMBC experiments (*vide infra*).

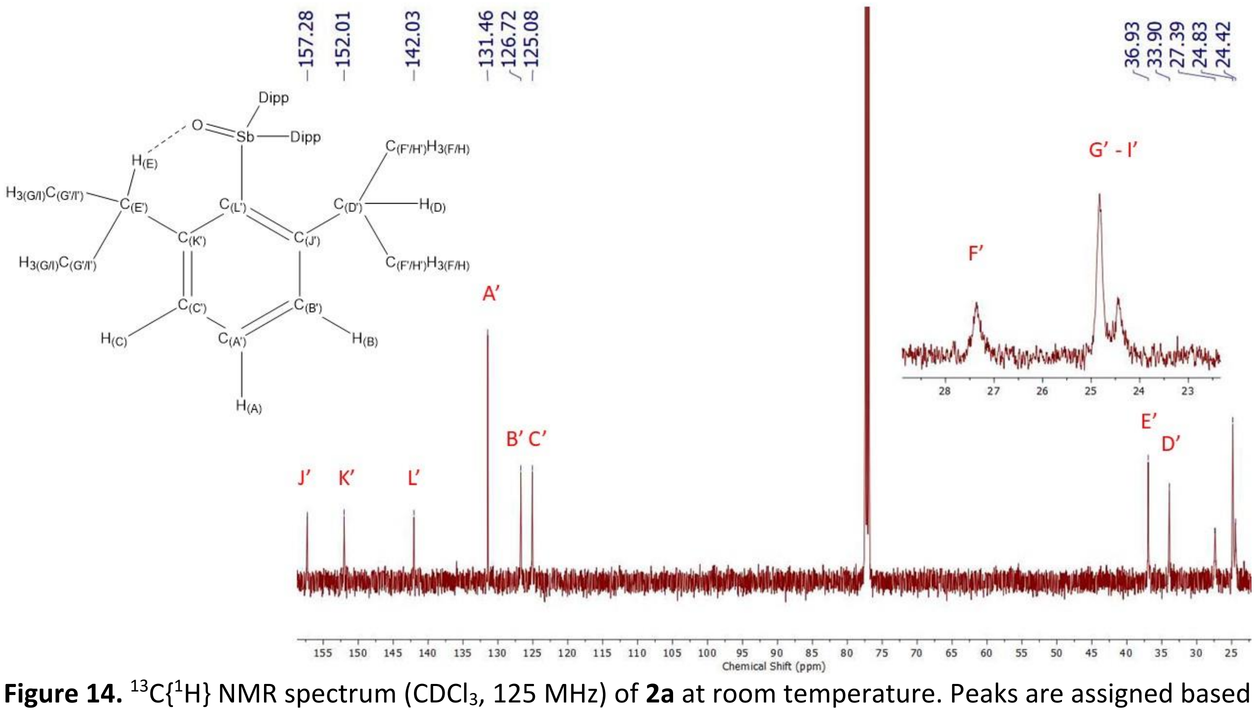

Figure 14. $^{13}C\{^{1}H\}$ NMR spectrum ($CDCl_3$, 125 MHz) of **2a** at room temperature. Peaks are assigned based on HSQC and HMBC experiments (*vide infra*).

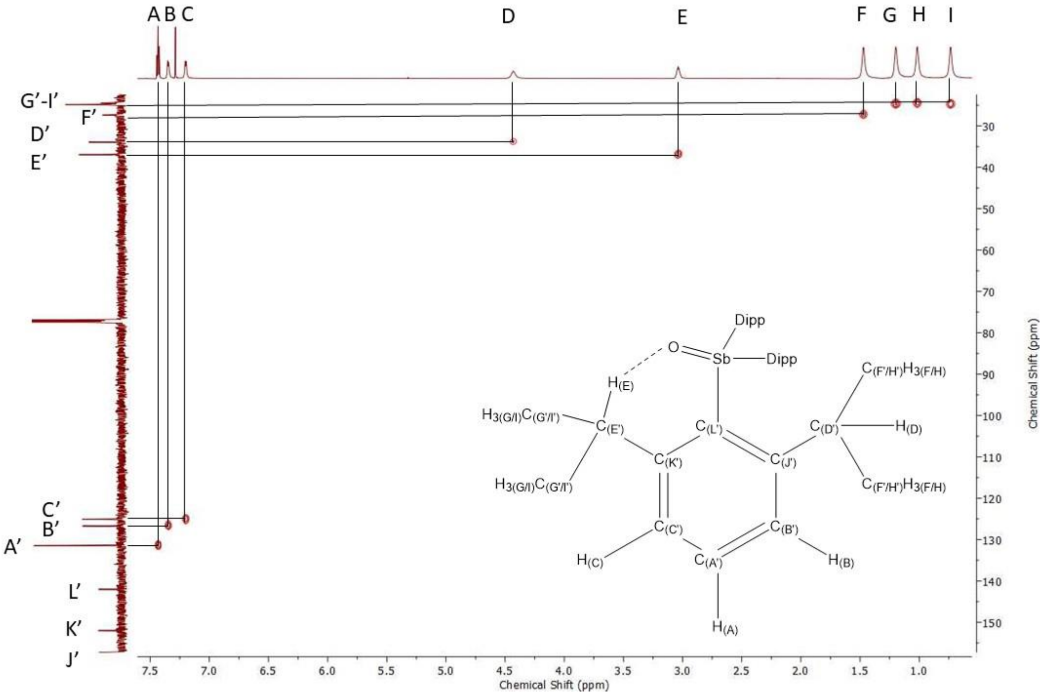

Figure 15. ^{1}H-^{13}C HSQC (800 MHz, $CDCl_3$) spectrum of **2a** at room temperature.

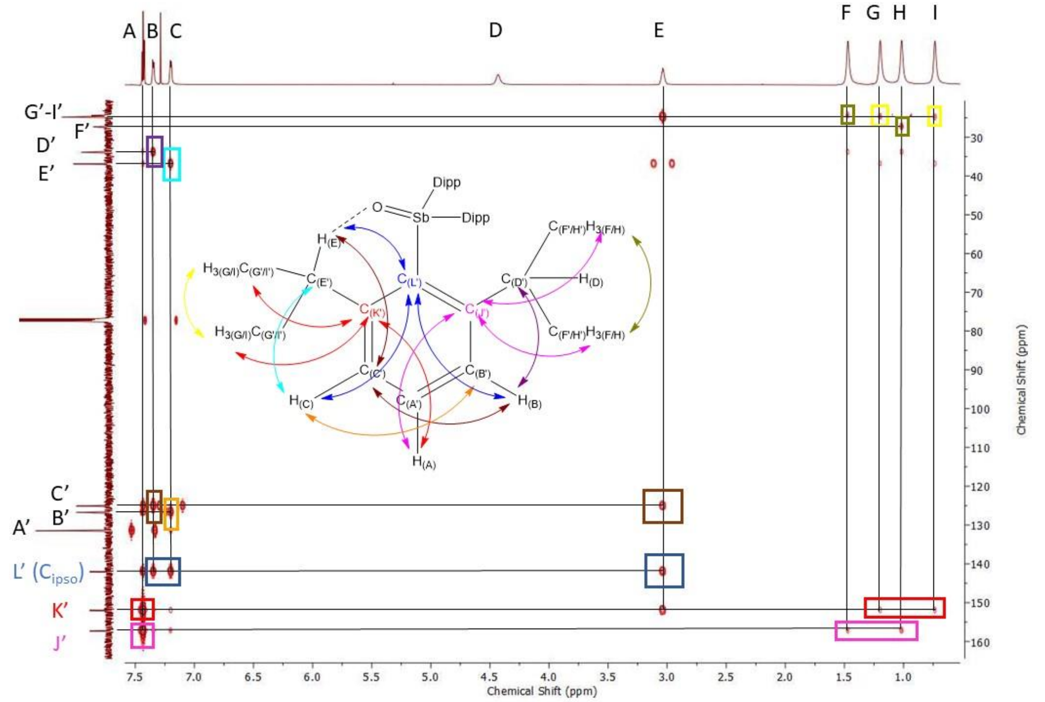

Figure 16. ^{1}H-^{13}C HMBC (800 MHz, $CDCl_3$) spectrum of **2a** at room temperature.

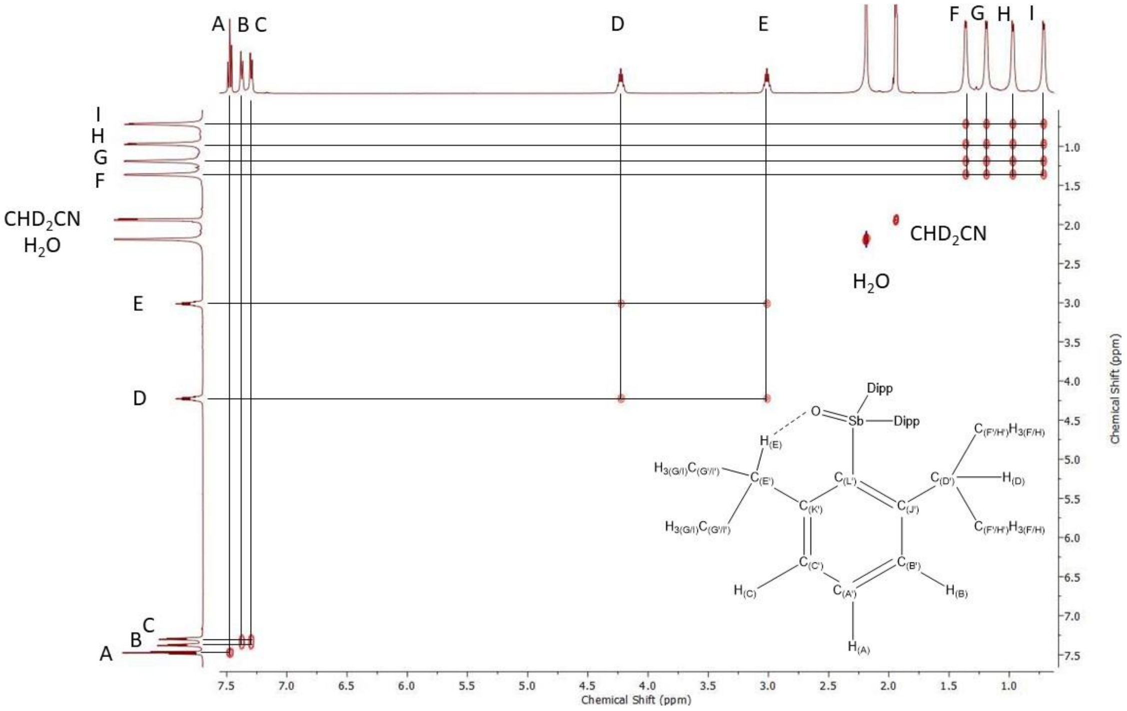

Figure 17. ^{1}H 2D EXSY (500 MHz, CD_3CN) of **2a** at room temperature.

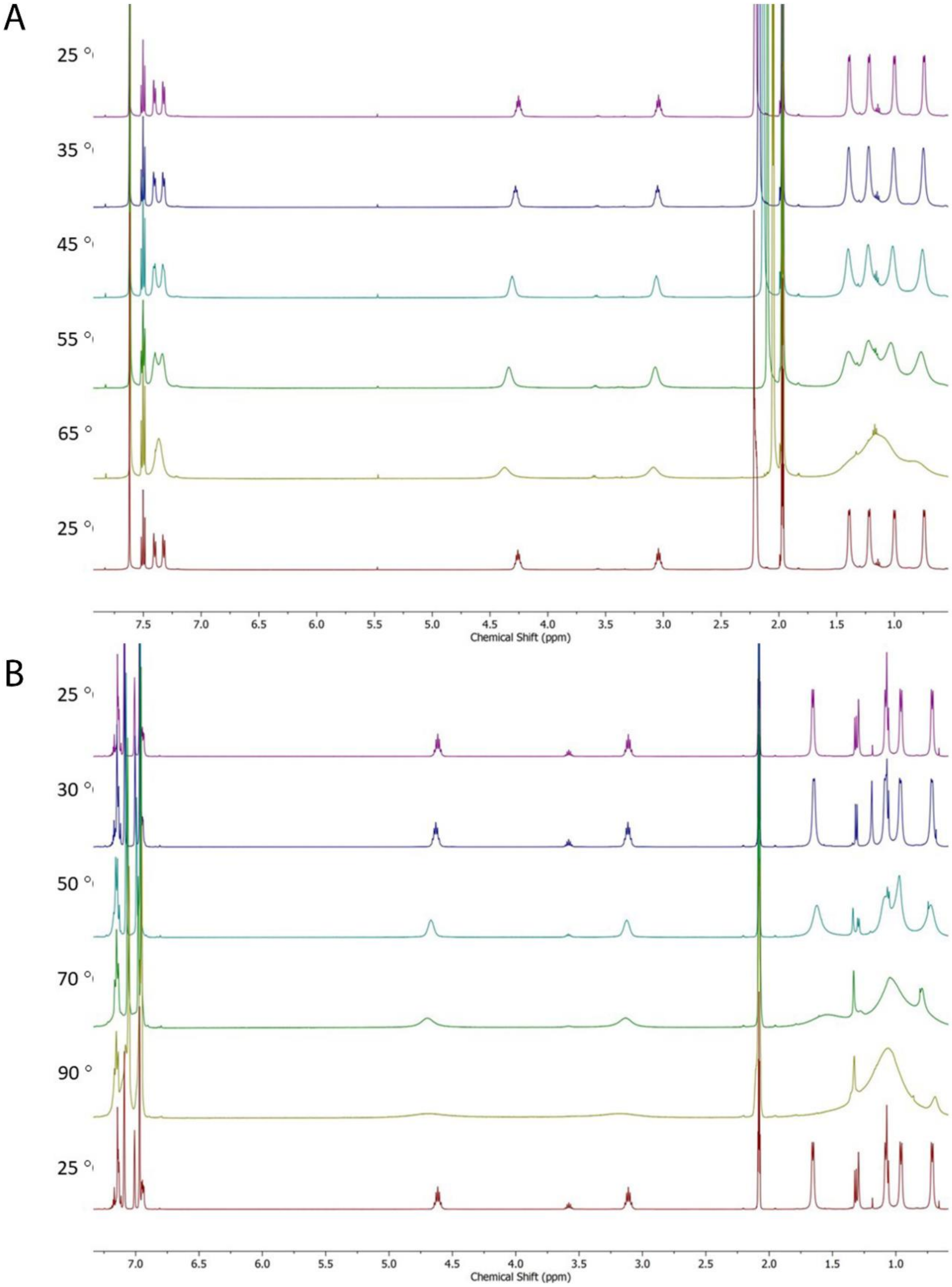

Figure 18. A) Variable temperature ^{1}H NMR (500 MHz, CD_3CN) spectra of **2a** from 25 °C to 65 °C, followed by cooling back to 25 °C in 10 °C increments. B) Variable temperature ^{1}H NMR (500 MHz, d_8-toluene) spectra of **2a** from 30 °C to 90 °C in 20 °C increments followed by cooling back to 25 °C. Intensities are scaled arbitrarily for viewing.

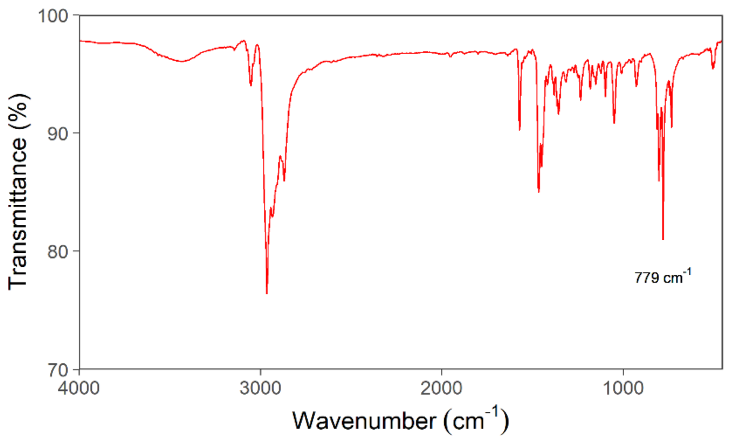

Figure 19. Experimental IR spectrum (KBr pellet) of **2a** (ν_{SbO} = 779 cm^{-1}).

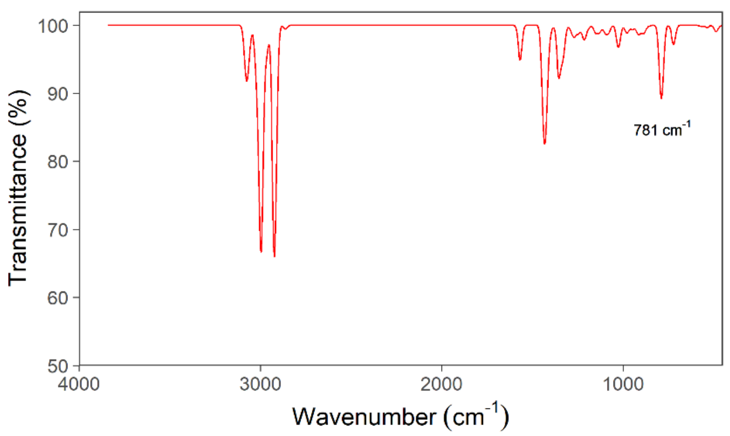

Figure 20. Calculated IR spectrum (PBE0/def2-TZVPP) of **2a** (ν_{SbO} = 781 cm^{-1}). The calculated IR vibrational frequencies were corrected with a 0.96 scalar factor.[15]

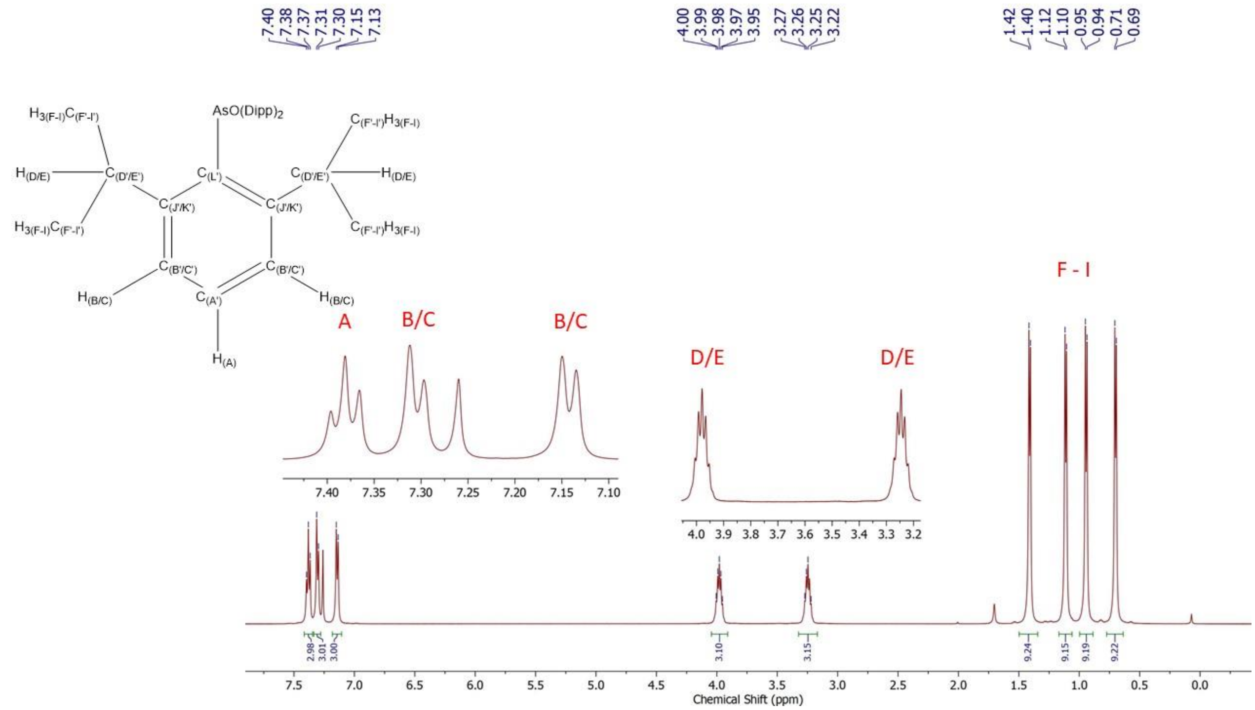

Figure 21. ^{1}H NMR spectrum ($CDCl_3$, 500 MHz) of **2b** at room temperature.

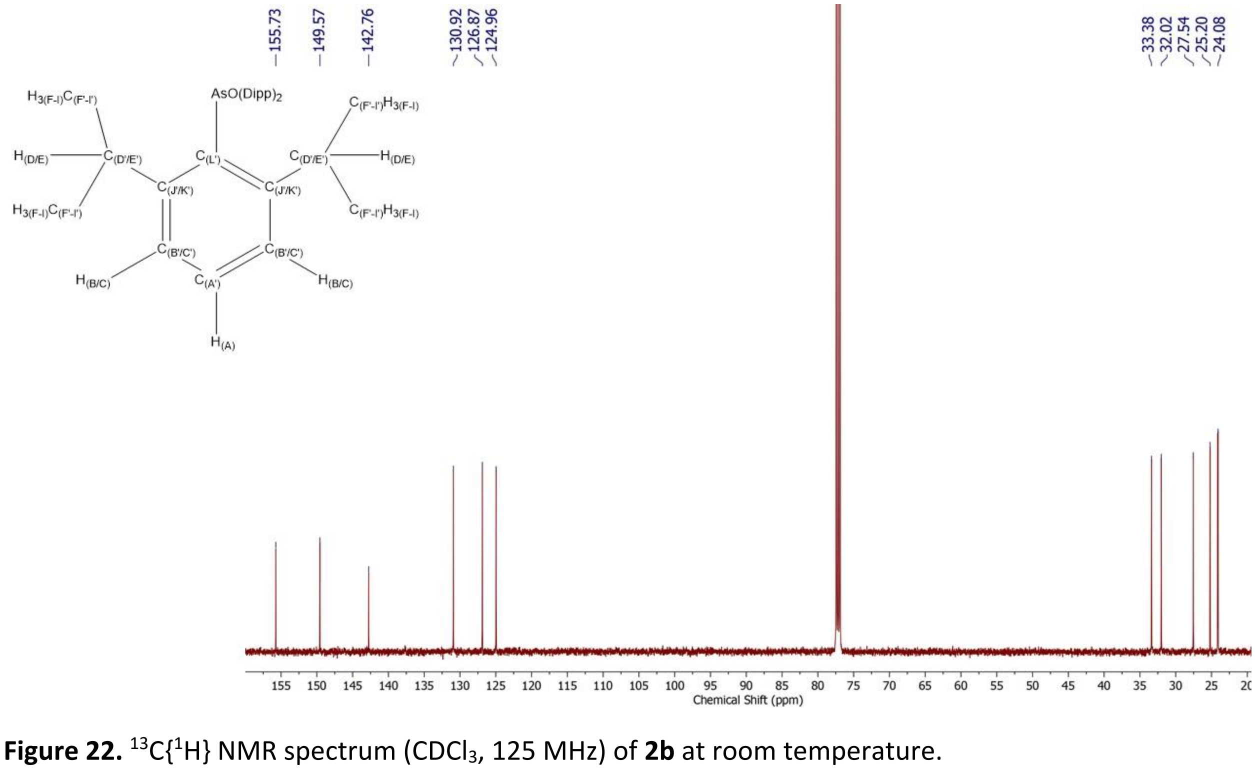

Figure 22. $^{13}C\{^{1}H\}$ NMR spectrum ($CDCl_3$, 125 MHz) of **2b** at room temperature.

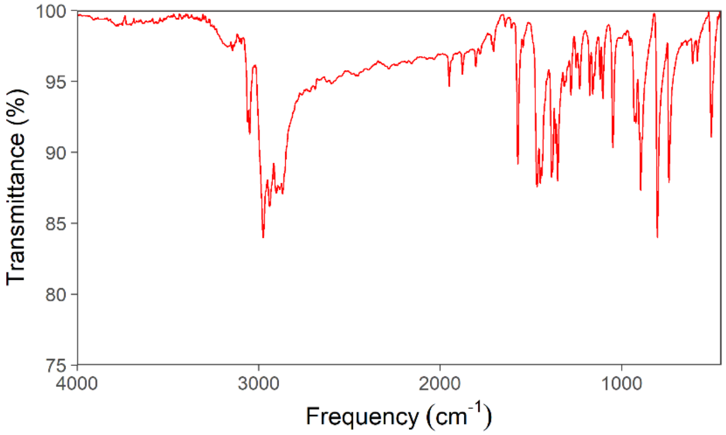

Figure 23. Experimental IR spectrum (KBr pellet) of **2b**.

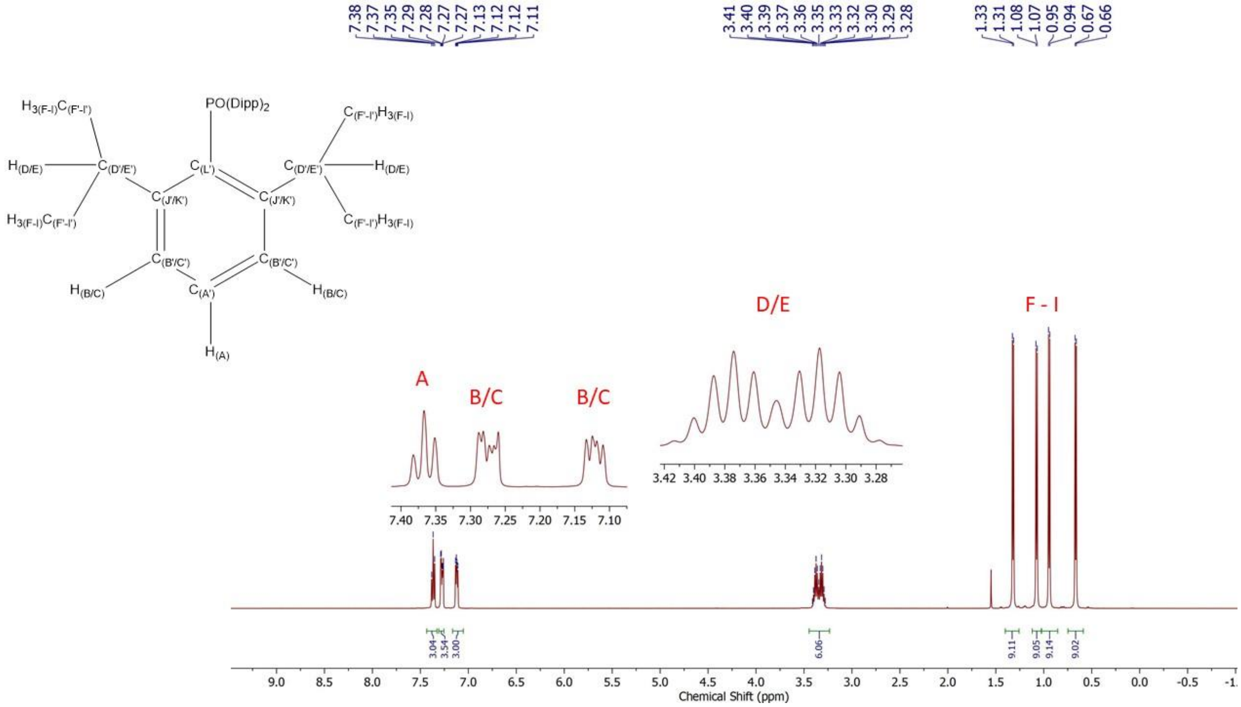

Figure 24. ^{1}H NMR spectrum ($CDCl_3$, 500 MHz) of **2c** at room temperature.

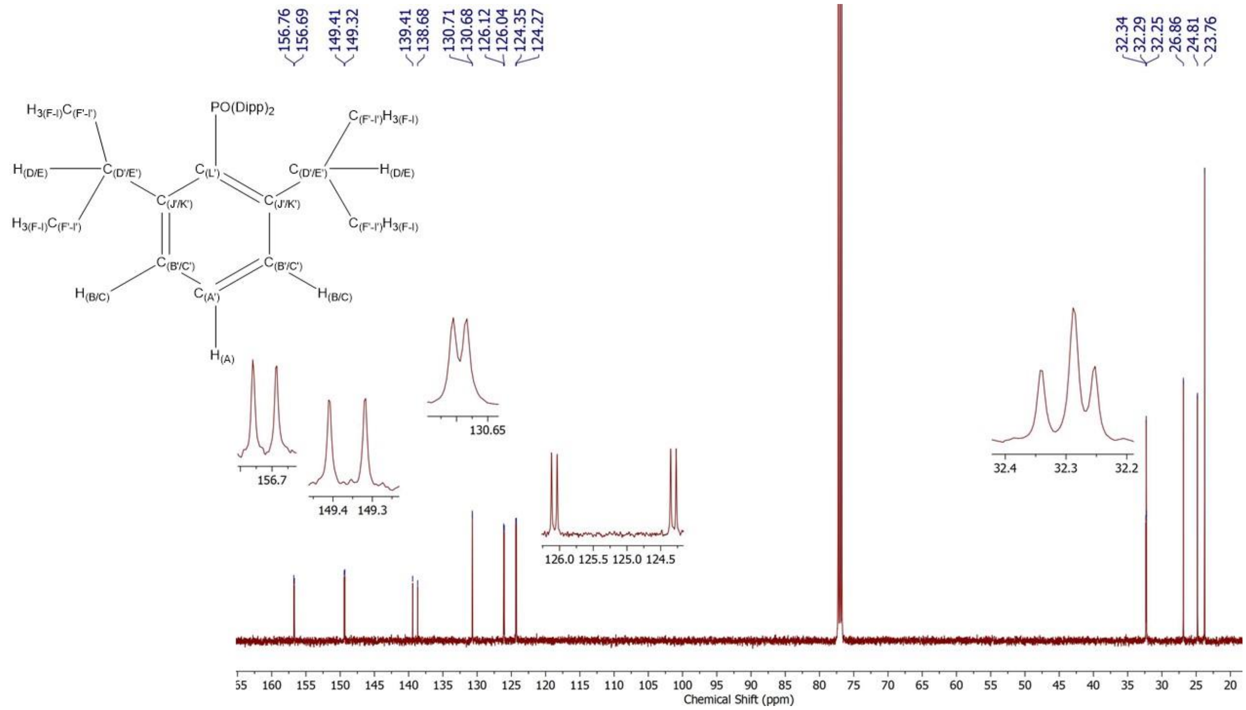

Figure 25. $^{13}C\{^{1}H\}$ NMR spectrum ($CDCl_3$, 125 MHz) of **2c** at room temperature.

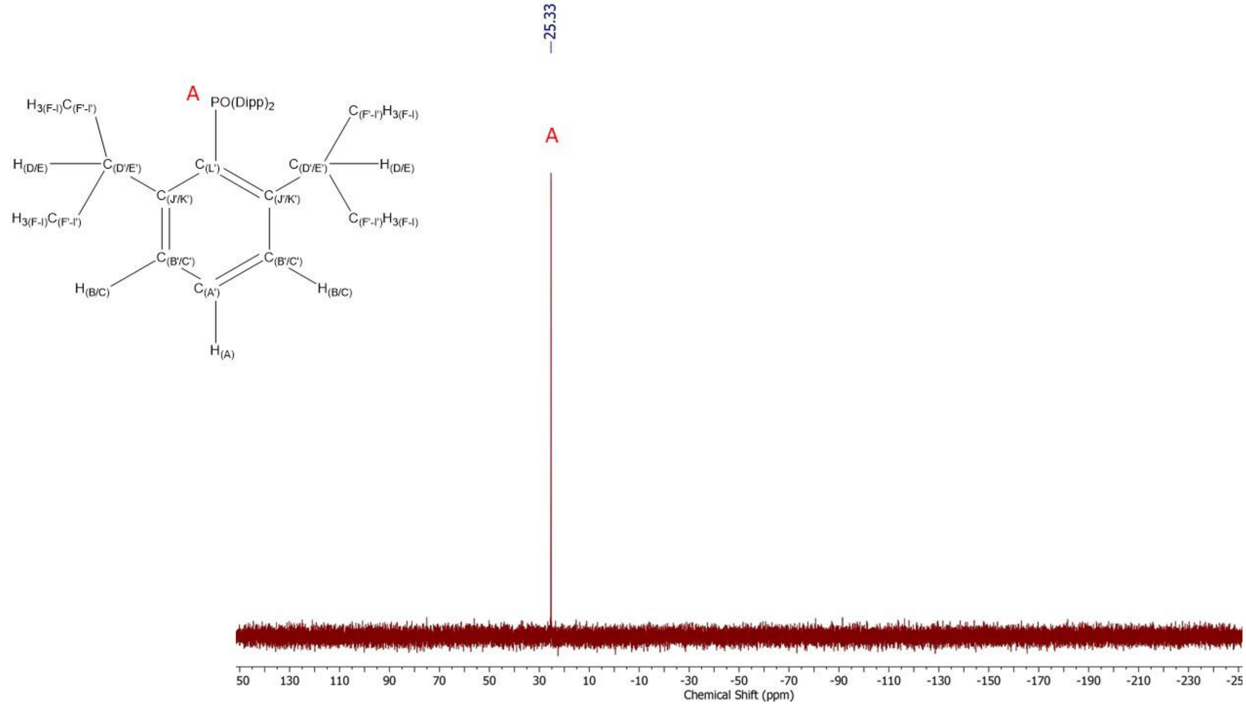

Figure 26. $^{31}P\{^{1}H\}$ NMR spectrum ($CDCl_3$, 202 MHz) of **2c** at room temperature.

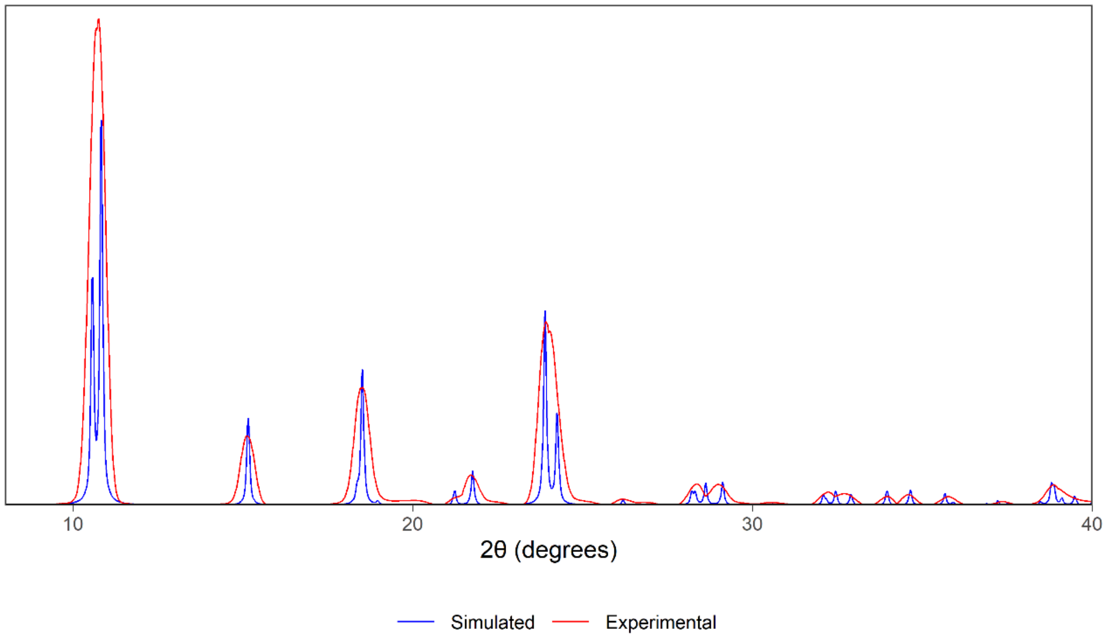

Figure 27. Simulated and experimental PXRD diffractogram of **2c**.

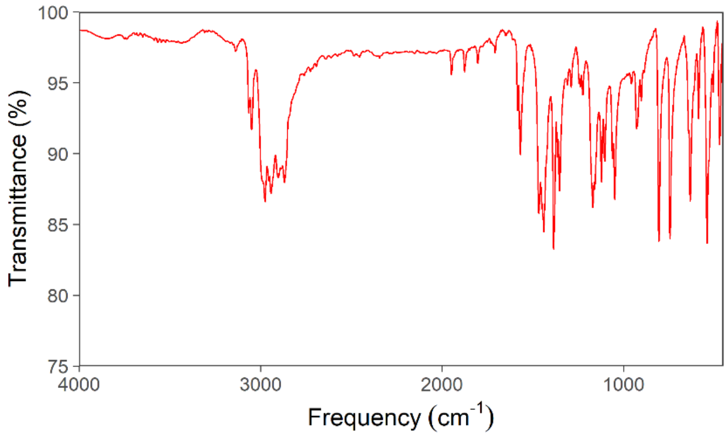

Figure 28. Experimental IR spectrum (KBr pellet) of **2c**.

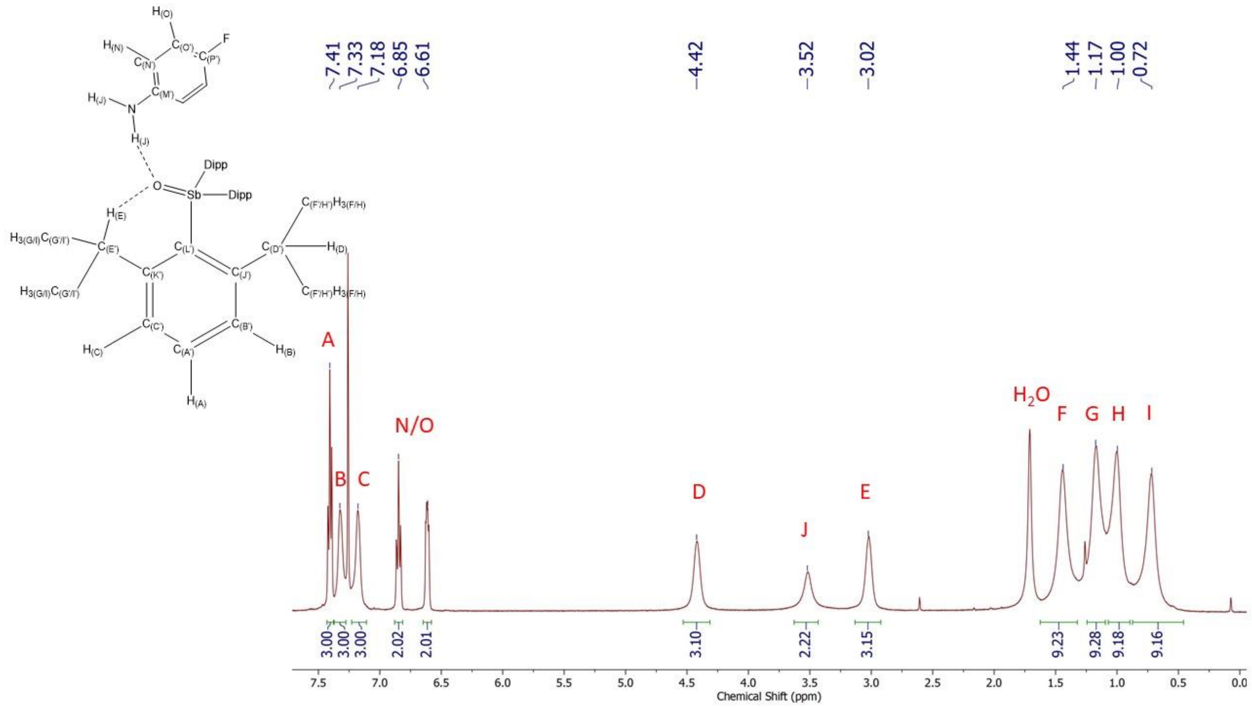

Figure 29. ^{1}H NMR spectrum ($CDCl_3$, 500 MHz) of **3** at room temperature.

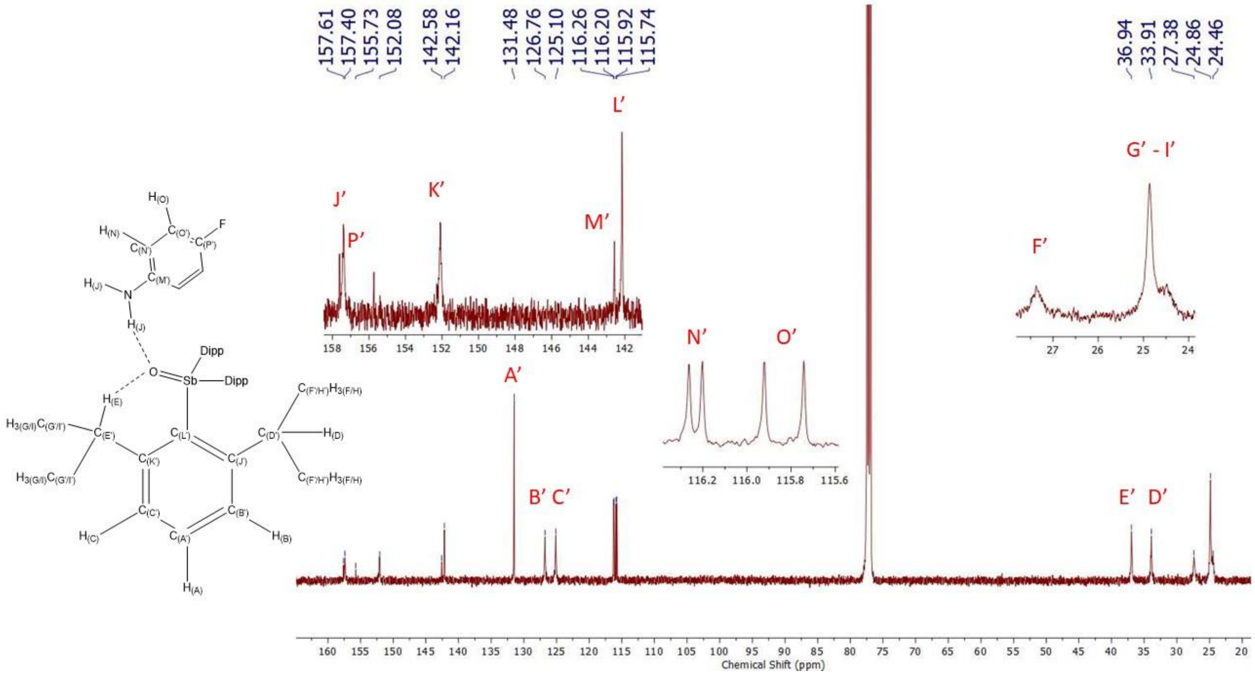

Figure 30. $^{13}C\{^{1}H\}$ NMR spectrum ($CDCl_3$, 125 MHz) of **3** at room temperature.

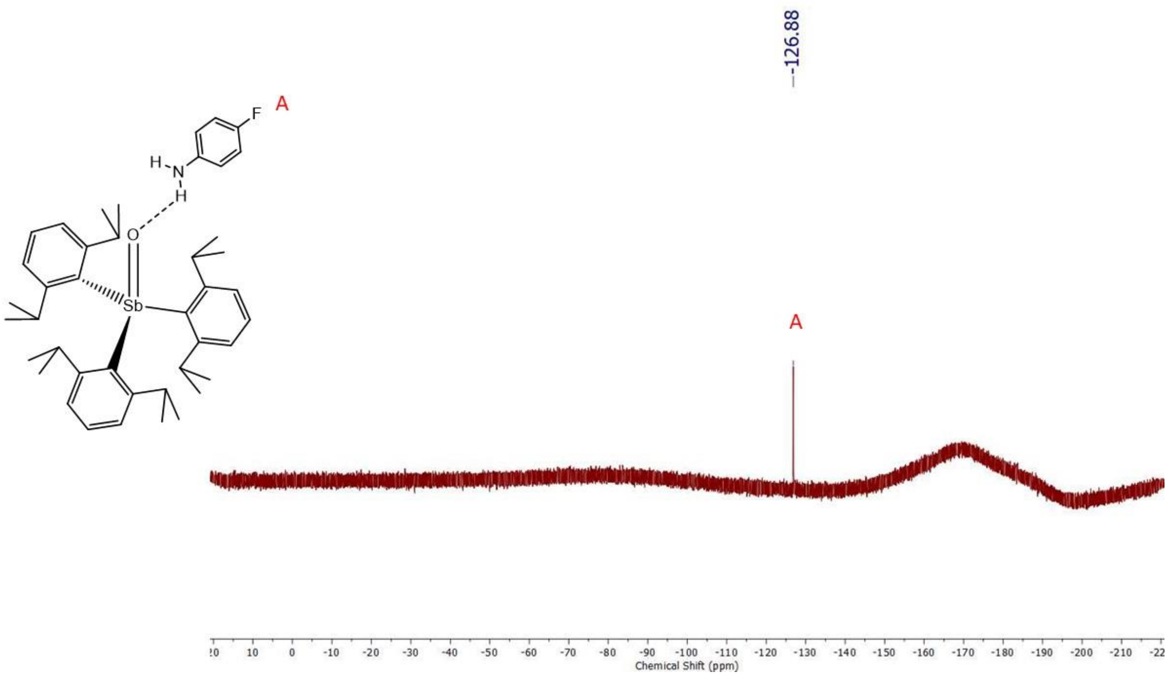

Figure 31. ^{19}F NMR spectrum ($CDCl_3$, 470 MHz) of **3** at room temperature.

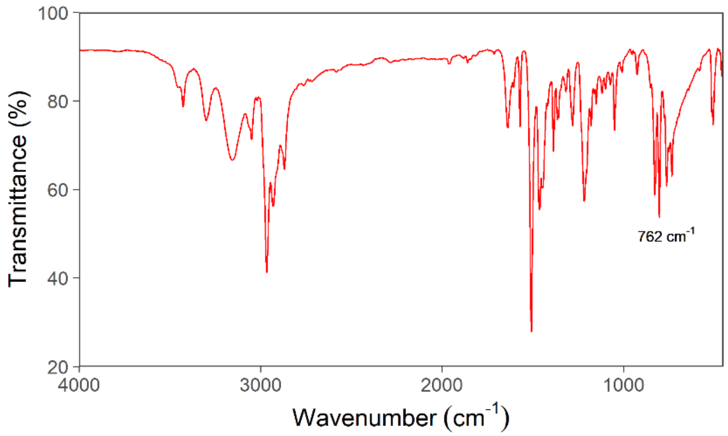

Figure 32. Experimental IR spectrum (KBr pellet) of **3** (ν_{SbO} = 762 cm^{-1}).

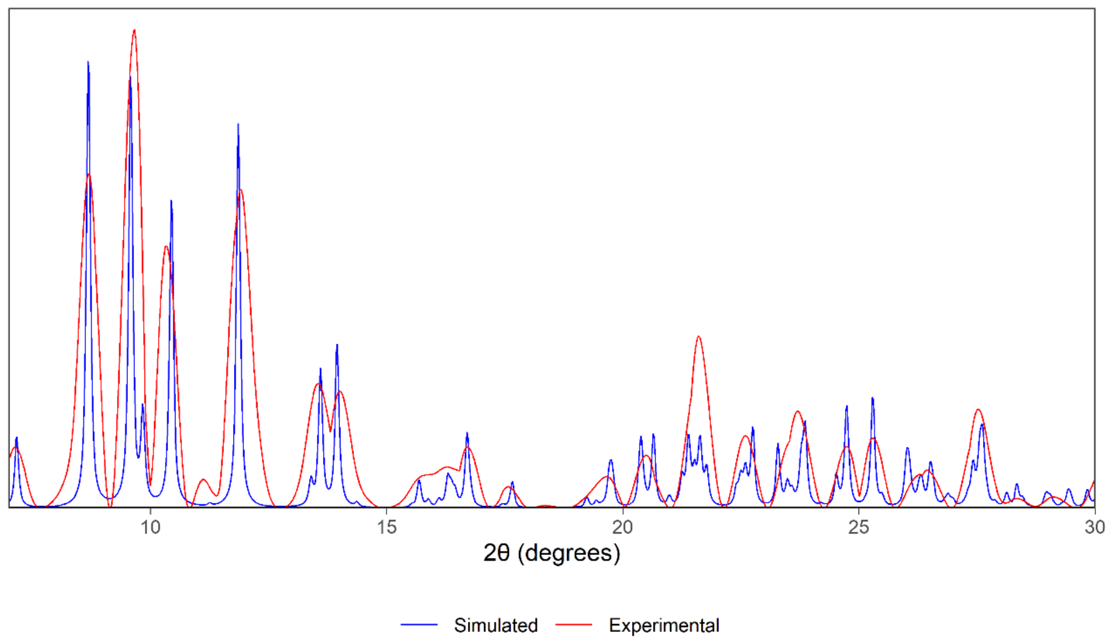

Figure 33. Simulated and experimental PXRD diffractogram of **3**.

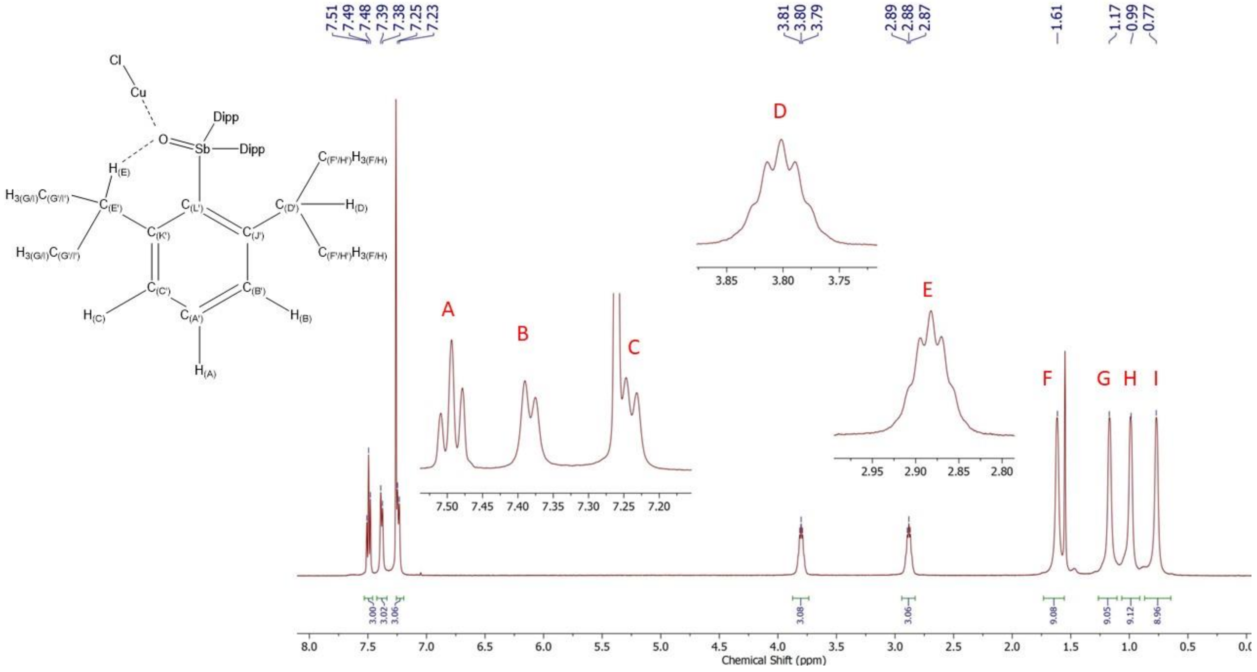

Figure 34. ^{1}H NMR spectrum ($CDCl_3$, 500 MHz) of **4**·$(CHCl_3)_2$ at room temperature.

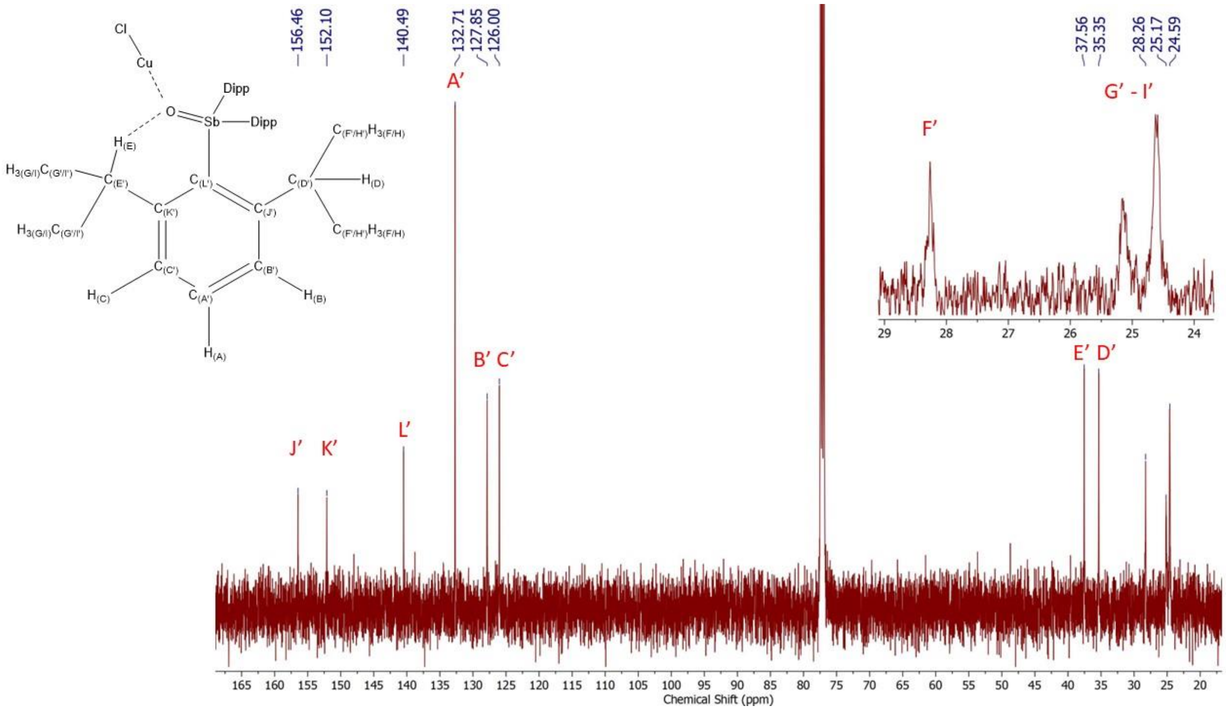

Figure 35. $^{13}C\{^1H\}$ NMR spectrum ($CDCl_3$, 125 MHz) of **4**·$(CHCl_3)_2$ at room temperature.

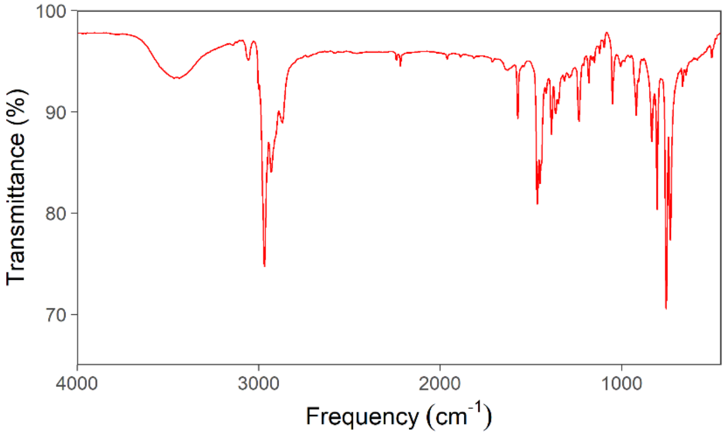

Figure 36. Experimental IR spectrum (KBr pellet) of **4**·$(CHCl_3)_2$.

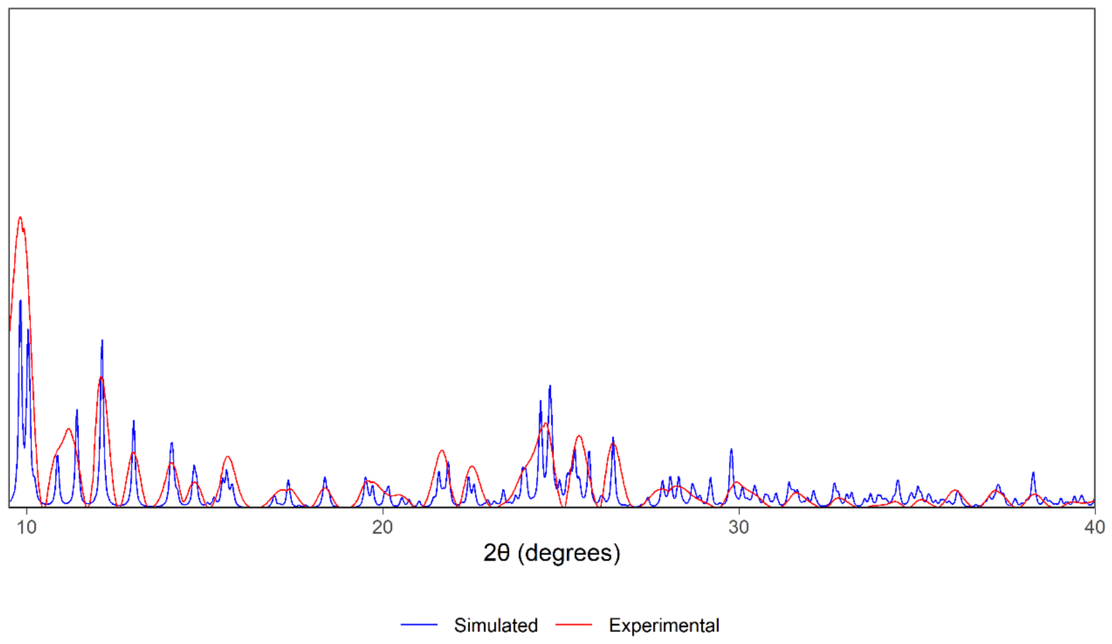

Figure 37. Simulated and experimental PXRD diffractogram of **4**·$(CHCl_3)_2$.

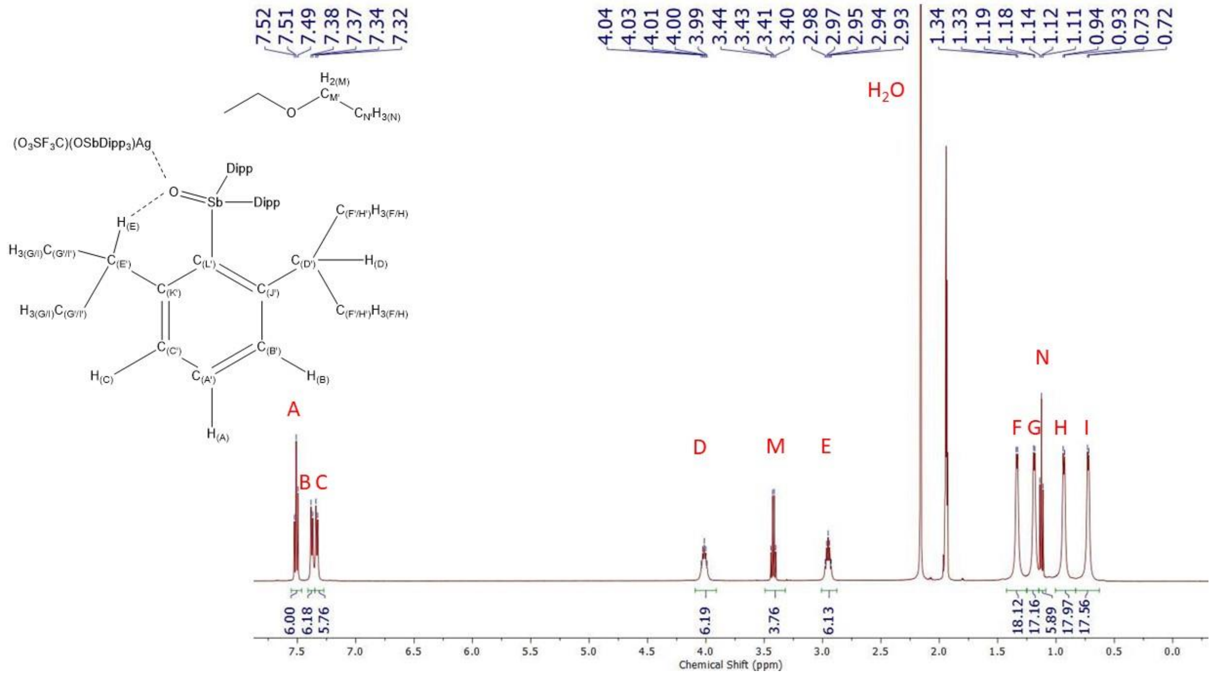

Figure 38. ^{1}H NMR spectrum (CD_3CN, 500 MHz) of **5**·OEt_2 at room temperature.

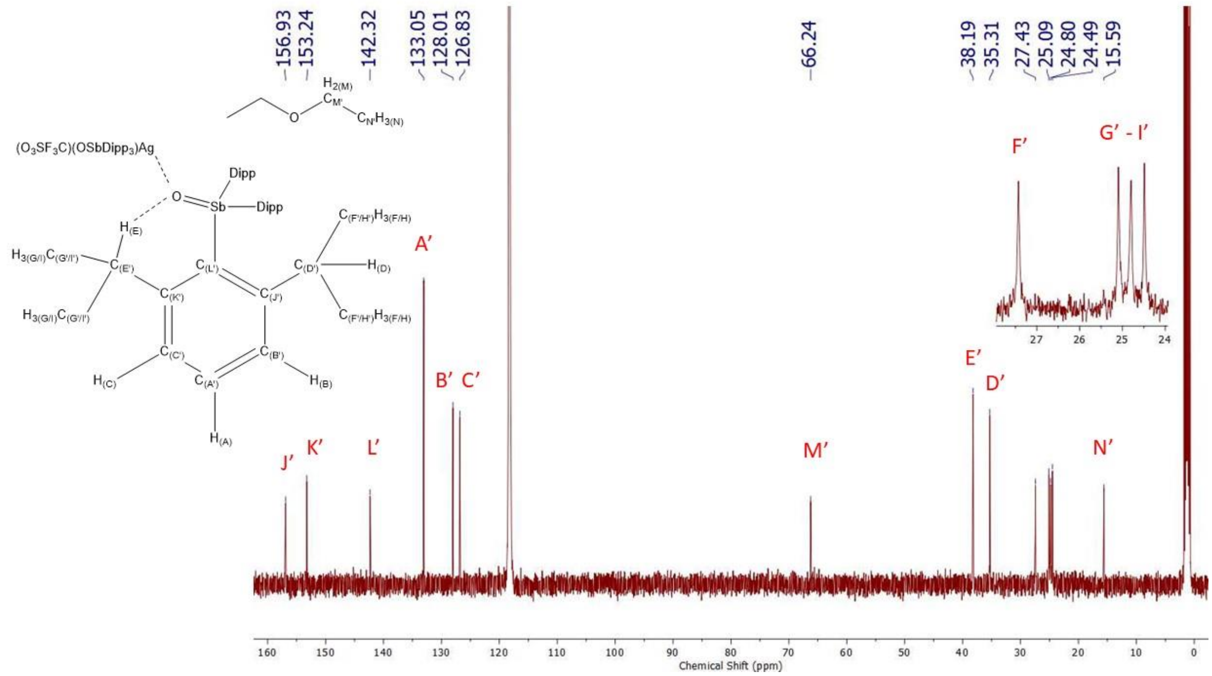

Figure 39. $^{13}C\{^{1}H\}$ NMR spectrum (CD_3CN, 125 MHz) of **5**·OEt_2 at room temperature.

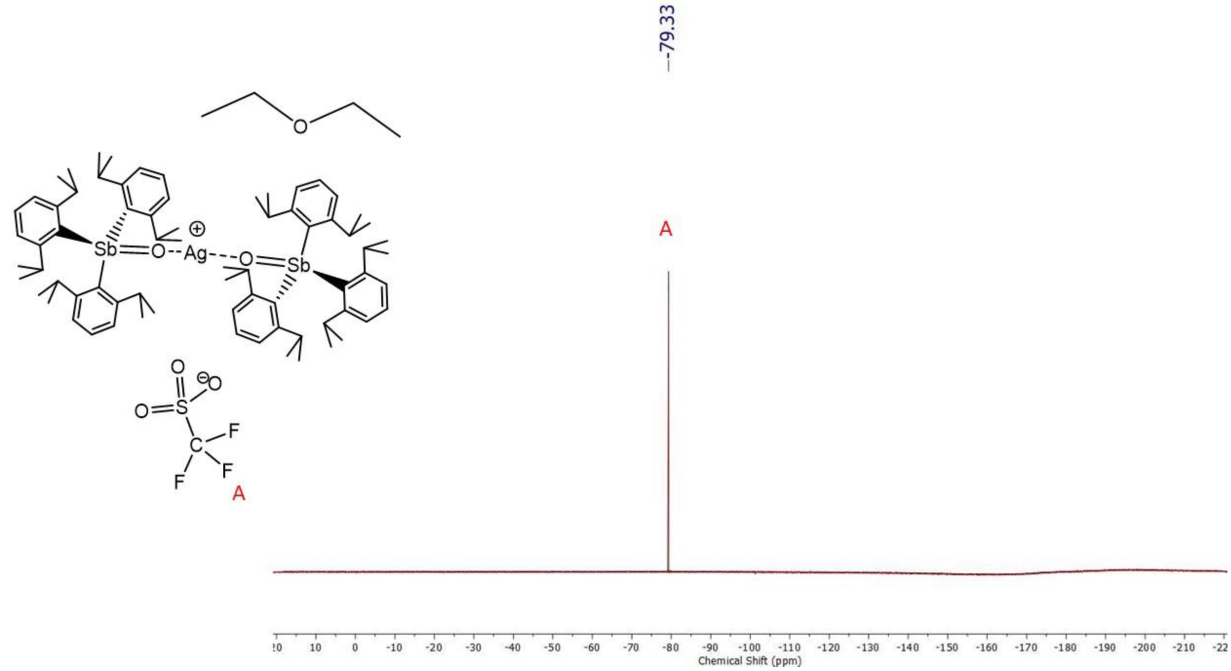

Figure 40. ^{19}F NMR spectrum ($CDCl_3$, 470 MHz) of **5**·OEt_2 at room temperature.

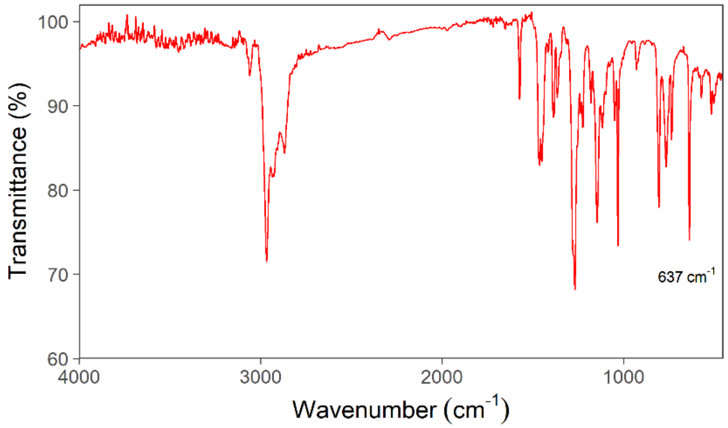

Figure 41. Experimental IR spectrum (KBr pellets) of **5**·OEt_2 (ν_{SbO} = 637 cm^{-1}).

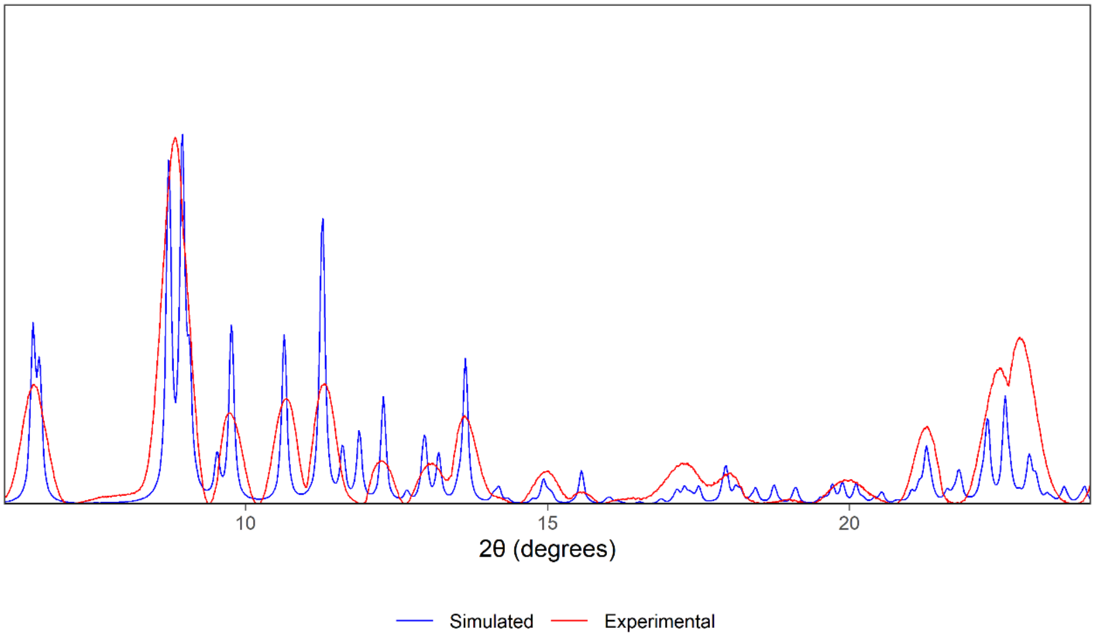

Figure 42. Simulated and experimental PXRD diffractogram of **5**·OEt_2.

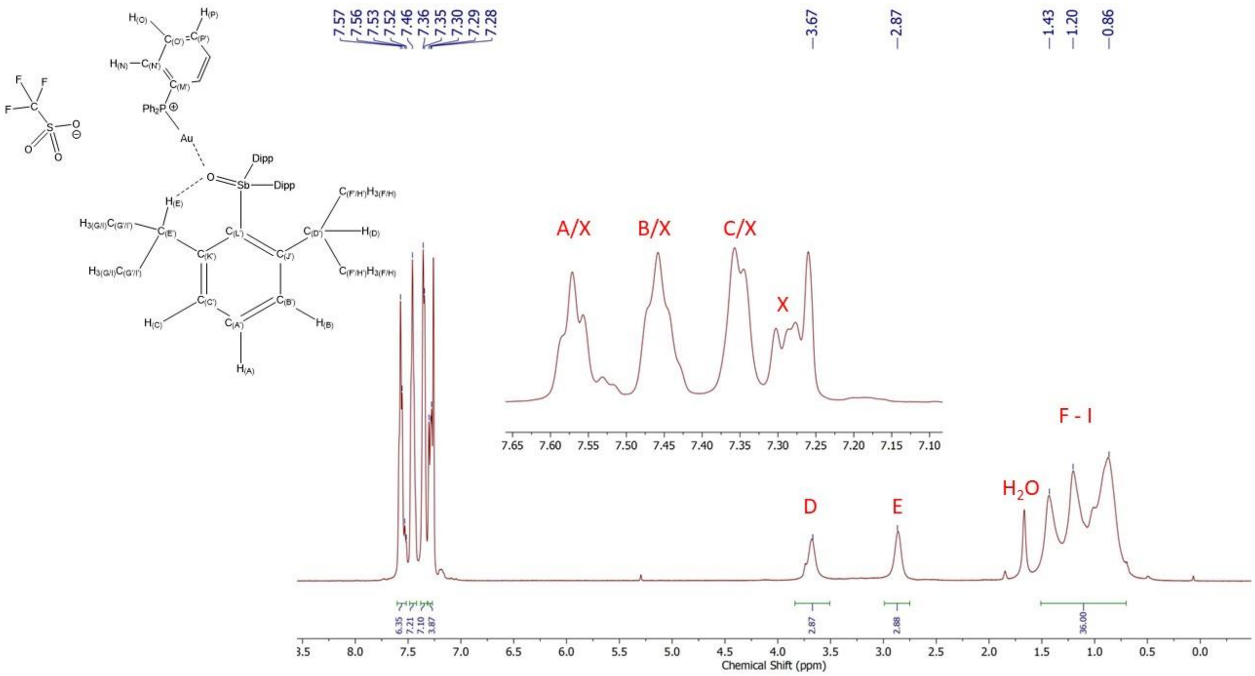

Figure 43. ^{1}H NMR spectrum ($CDCl_3$, 500 MHz) of **6** at room temperature. Peaks that correspond to **6** but that cannot be unambiguously assigned are labelled with an "X".

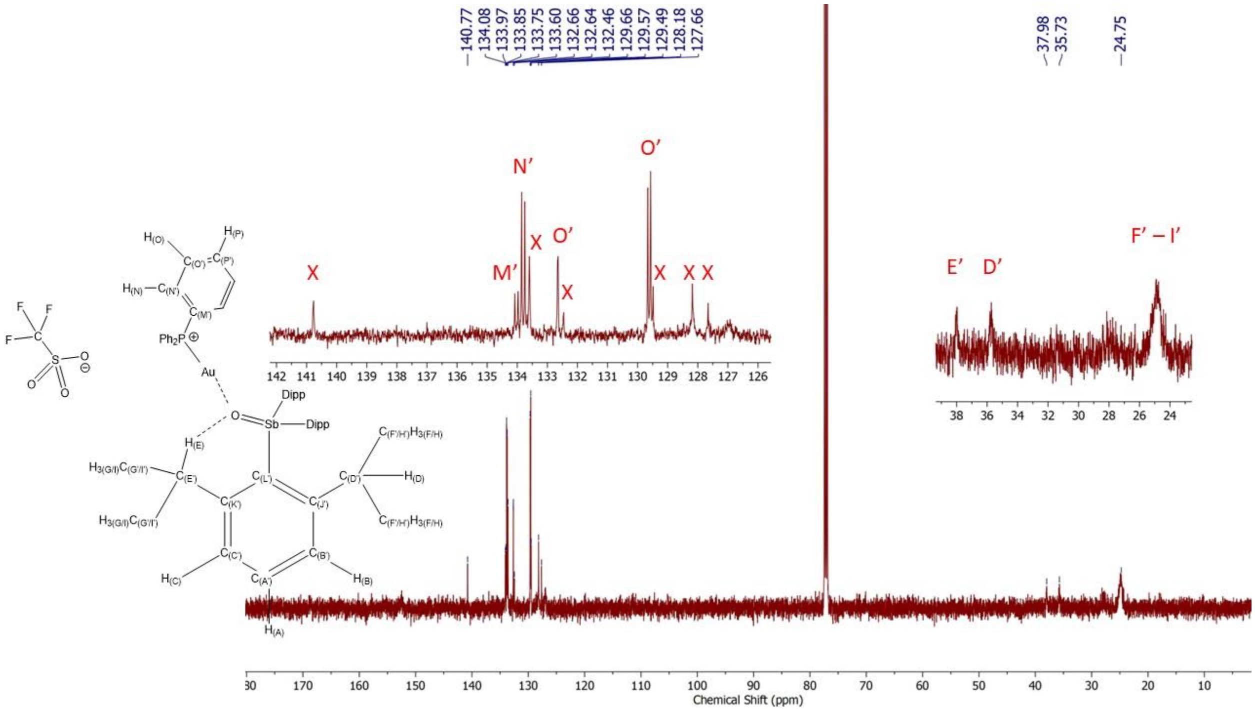

Figure 44. $^{13}C\{^{1}H\}$ NMR spectrum ($CDCl_3$, 125 MHz) of **6** at room temperature. Peaks that correspond to **6** but that cannot be unambiguously assigned are labelled with an "X".

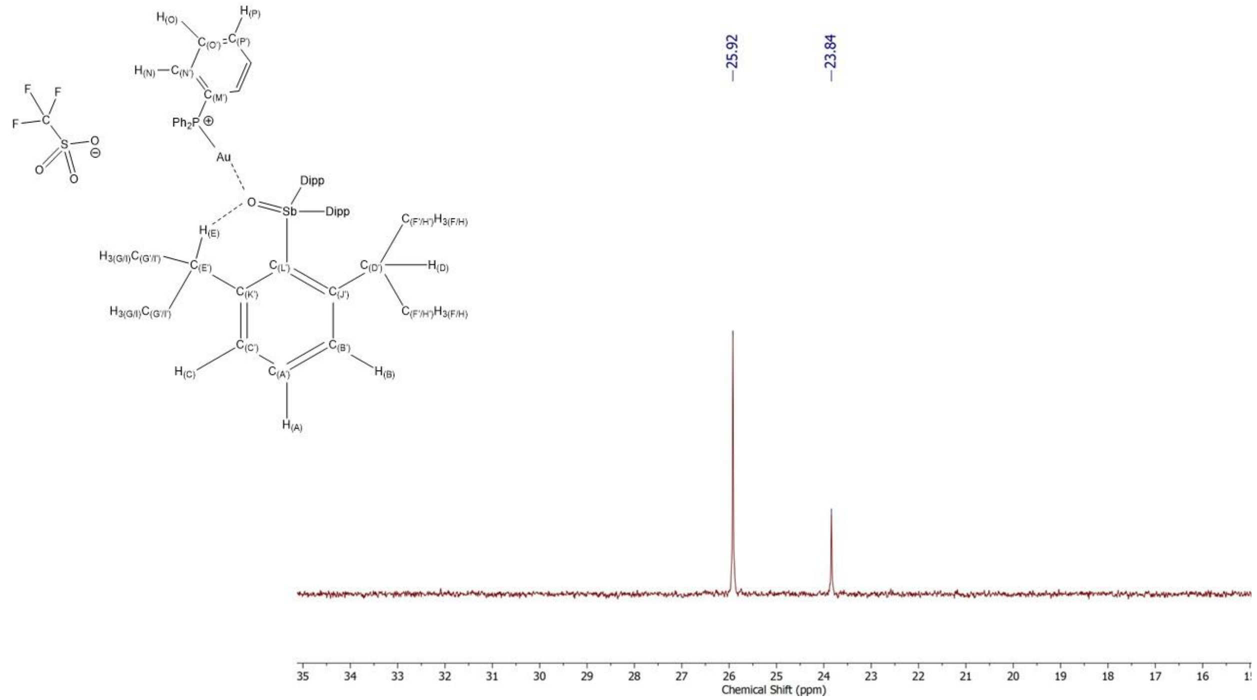

Figure 45. $^{31}P\{^{1}H\}$ NMR spectrum ($CDCl_3$, 202 MHz) of **6** at room temperature.

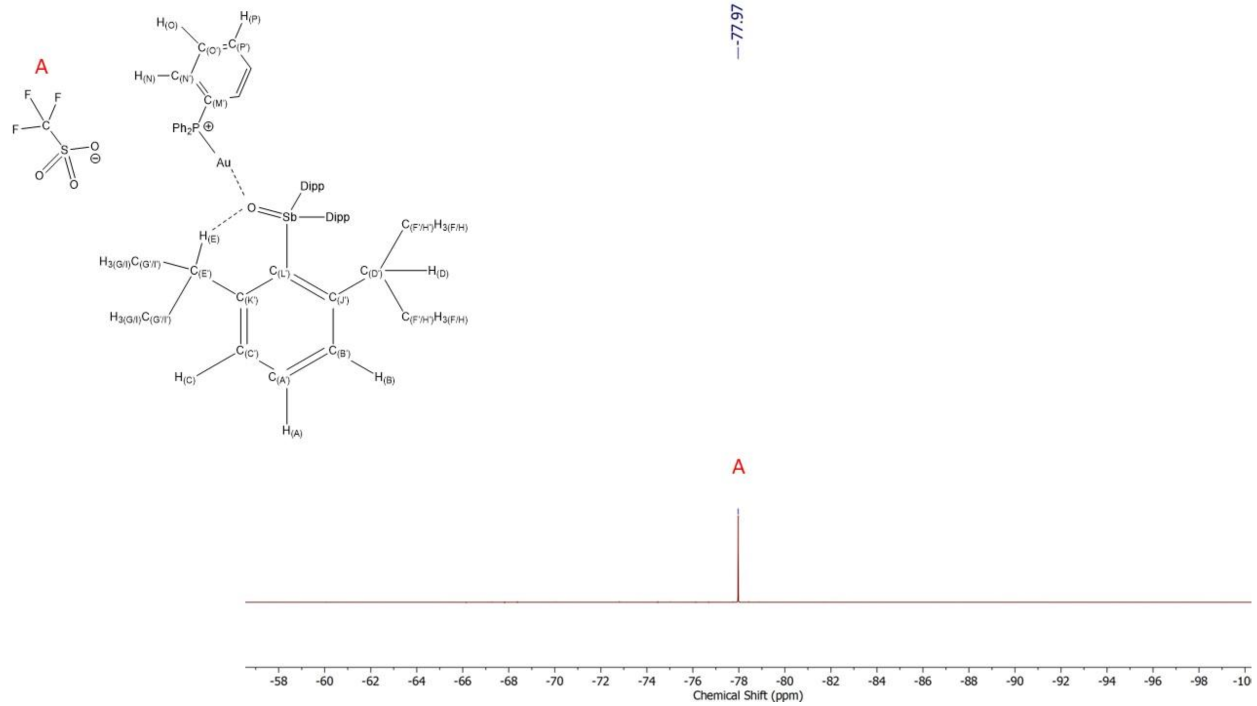

Figure 46. ^{19}F NMR spectrum ($CDCl_3$, 470 MHz) of **6** at room temperature.

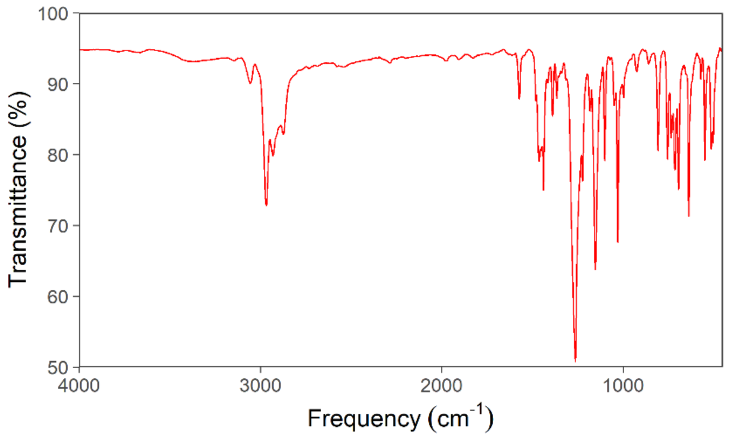

Figure 47. Experimental IR spectrum (KBr pellet) of **6**.

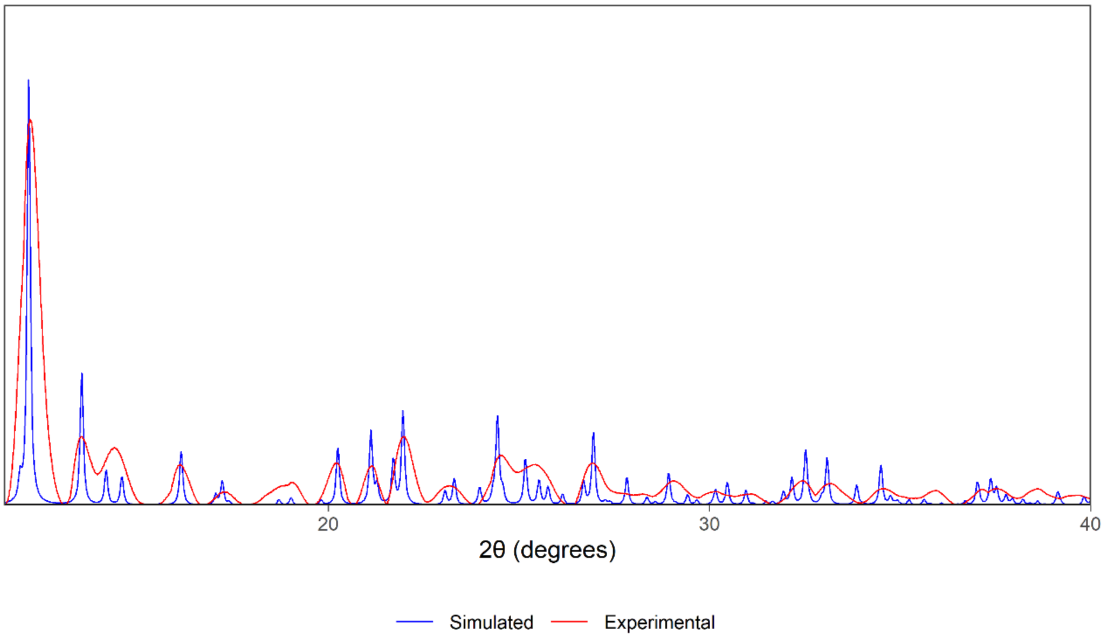

Figure 48. Simulated and experimental PXRD diffractogram of **6**. The simulated diffractogram was simulated from the linear polymorph.

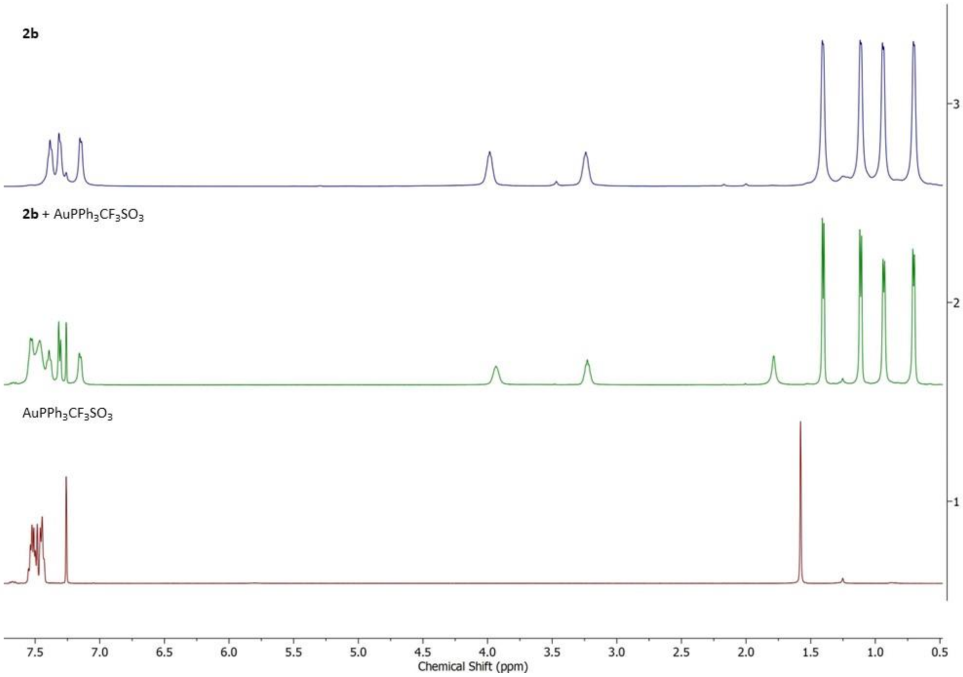

Figure 49. ^{1}H NMR spectra ($CDCl_3$, 500 MHz) of **2b** (*top*), a 1:1 mixture of **2b** and $AuPPh_3CF_3SO_3$ (*middle*), and $AuPPh_3CF_3SO_3$ (*bottom*) at room temperature.

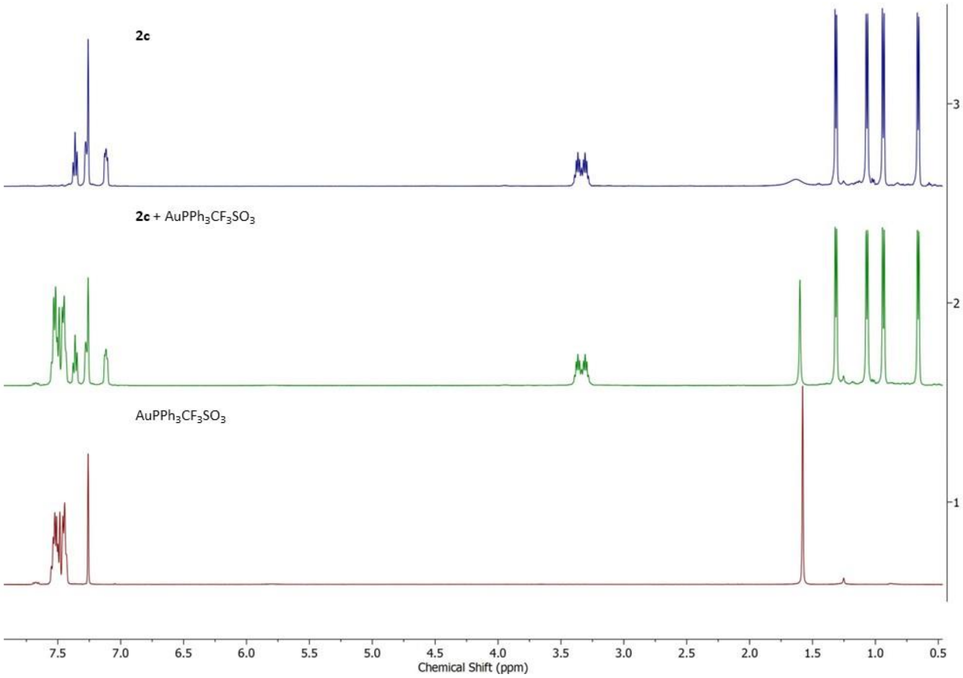

Figure 50. ^{1}H NMR spectra ($CDCl_3$, 500 MHz) of **2c** (*top*), a 1:1 mixture of **2c** and $AuPPh_3CF_3SO_3$ (*middle*), and $AuPPh_3CF_3SO_3$ (*bottom*) at room temperature.

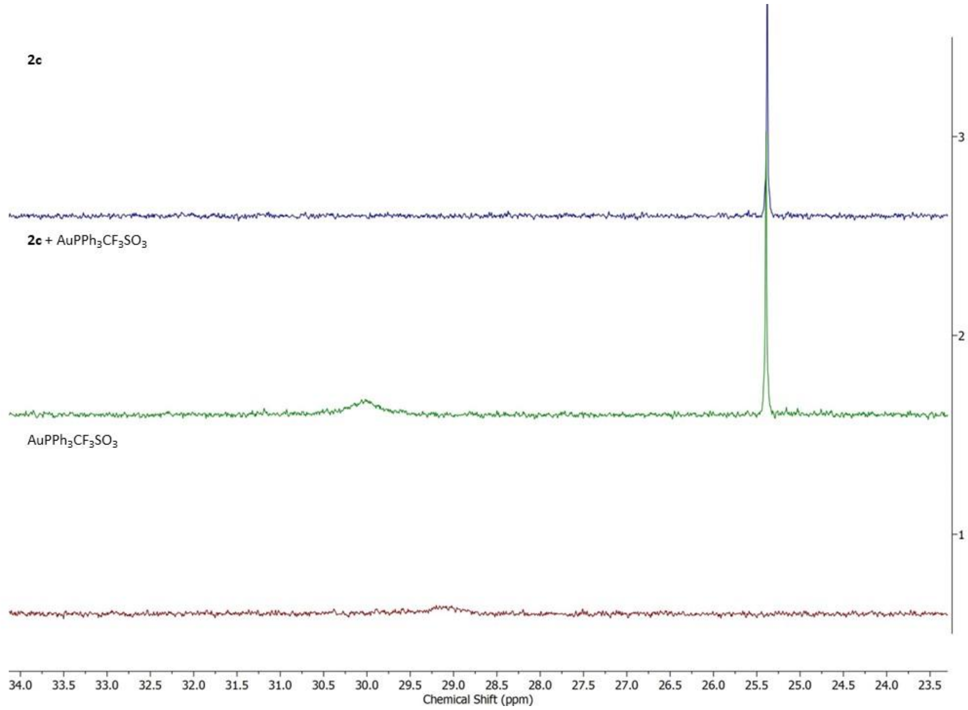

Figure 51. $^{31}P\{^{1}H\}$ NMR spectrum ($CDCl_3$, 202 MHz) of **2c** (*top*), a 1:1 mixture of **2c** and $AuPPh_3CF_3SO_3$ (*middle*), and $AuPPh_3CF_3SO_3$ (*bottom*) at room temperature.

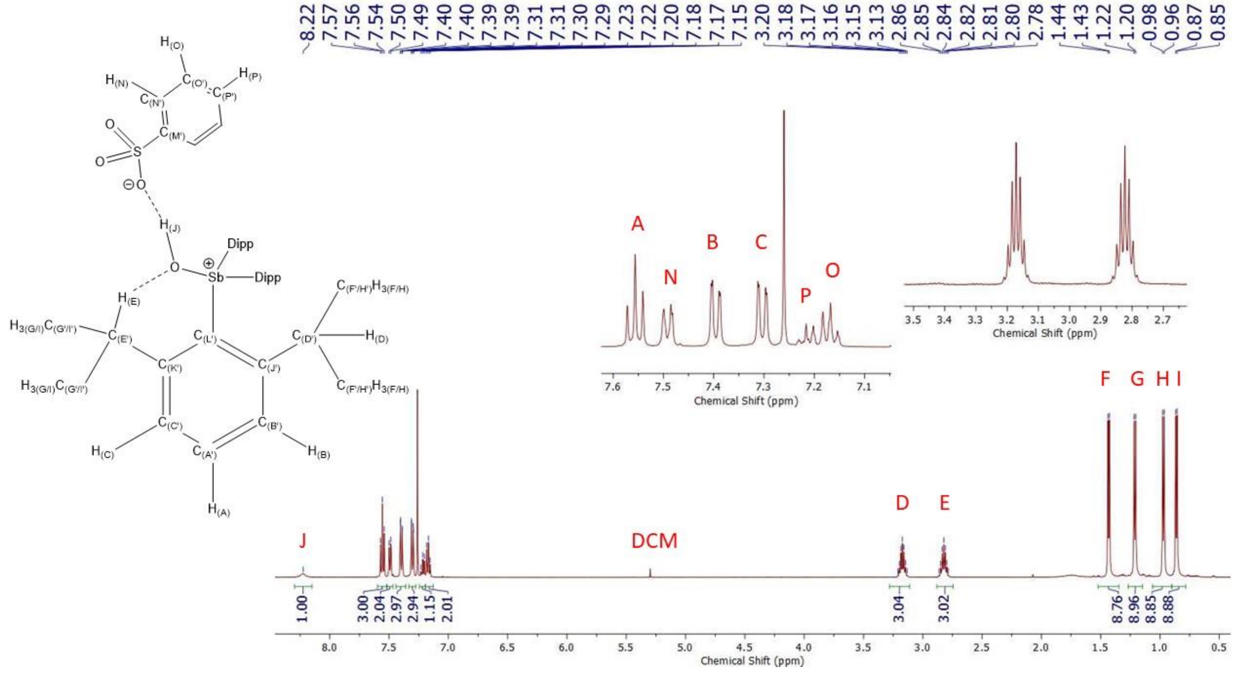

Figure 52. ^{1}H NMR spectrum ($CDCl_3$, 500 MHz) of **7a** at room temperature.

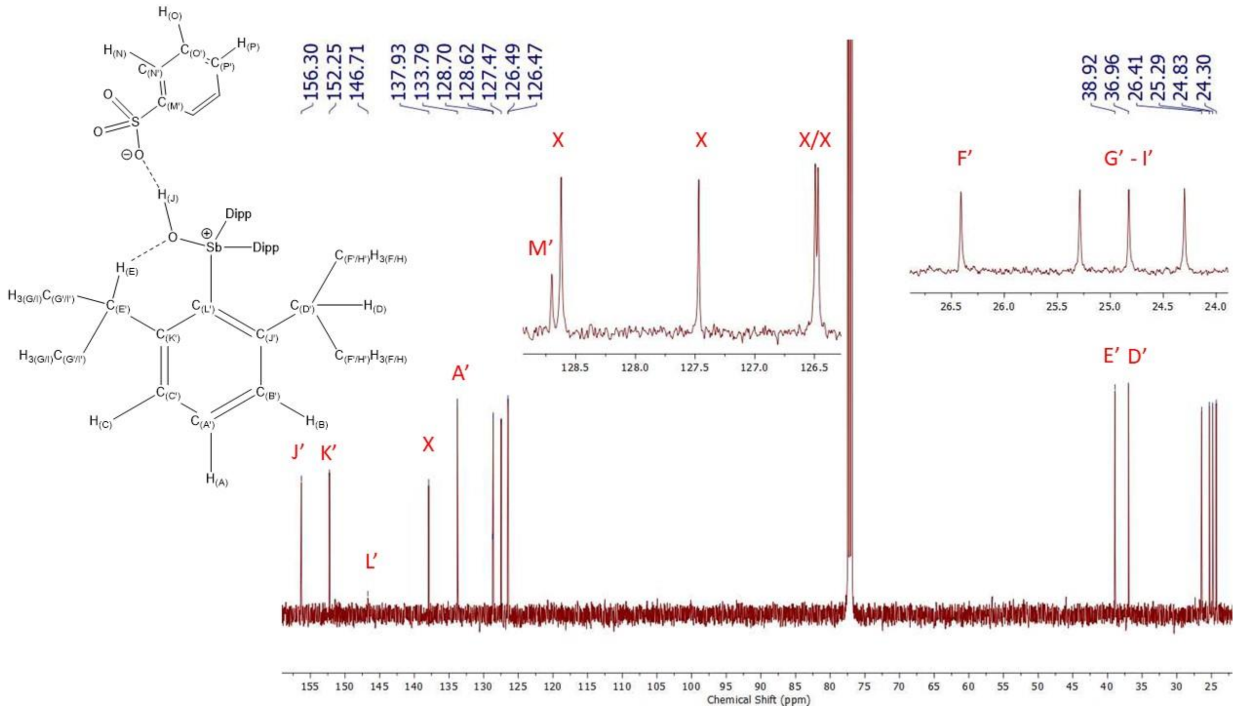

Figure 53. $^{13}C\{^1H\}$ NMR spectrum ($CDCl_3$, 125 MHz) of **7a** at room temperature. Peaks that correspond to **7a** but that cannot be unambiguously assigned are labelled with an "X".

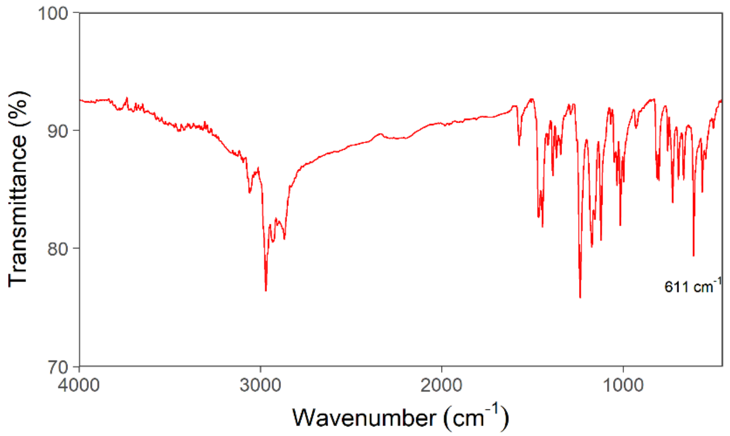

Figure 54. Experimental IR spectrum (KBr pellet) of **7a** (ν_{SbO} = 611 cm^{-1}).

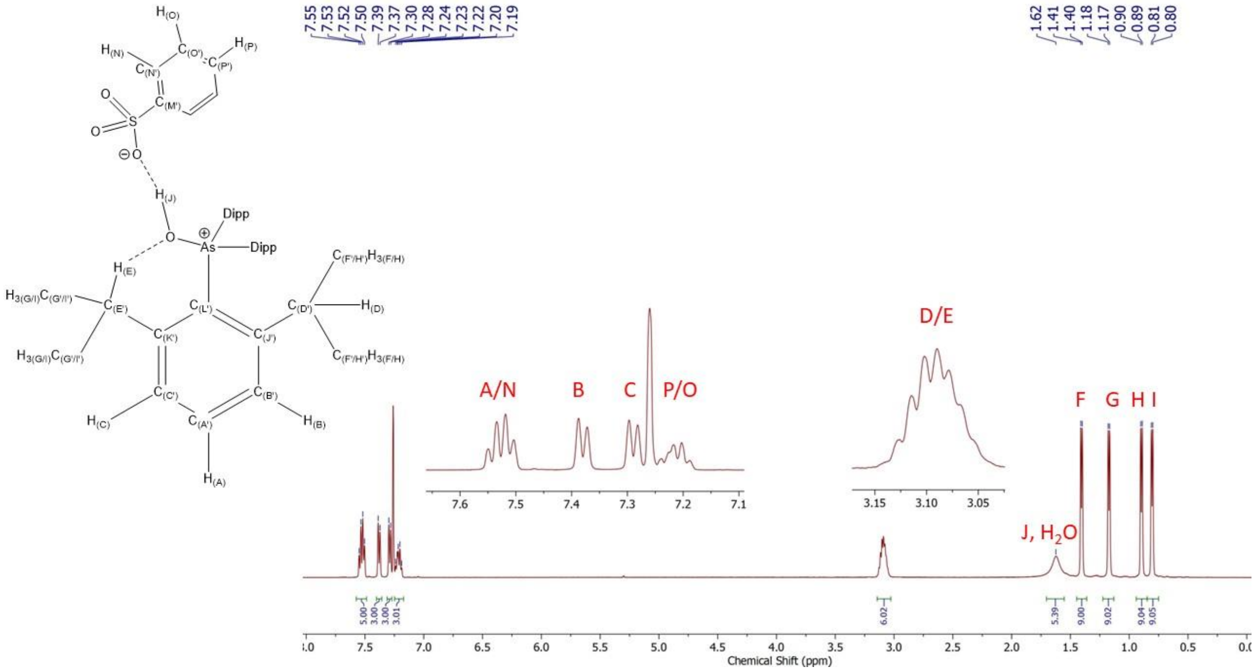

Figure 55. ^{1}H NMR spectrum ($CDCl_3$, 500 MHz) of **7b** at room temperature.

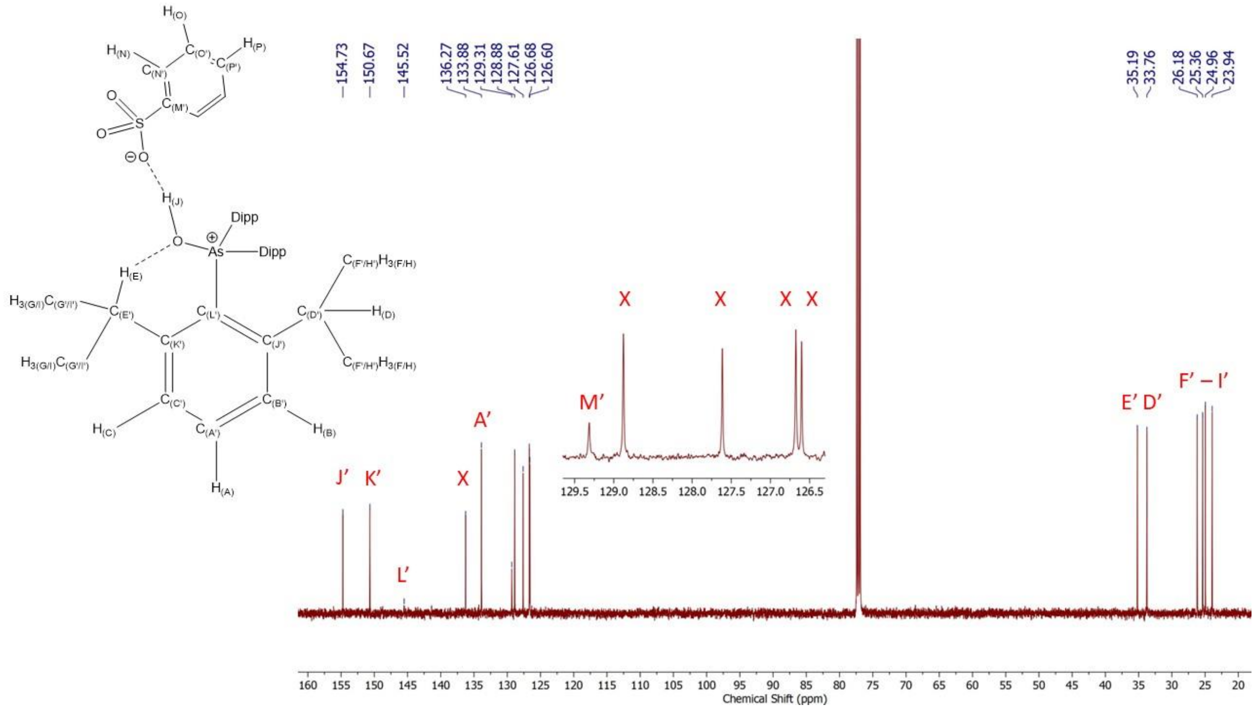

Figure 56. $^{13}C\{^{1}H\}$ NMR spectrum ($CDCl_3$, 125 MHz) of **7b** at room temperature. Peaks that correspond to **7b** but that cannot be unambiguously assigned are labelled with an "X".

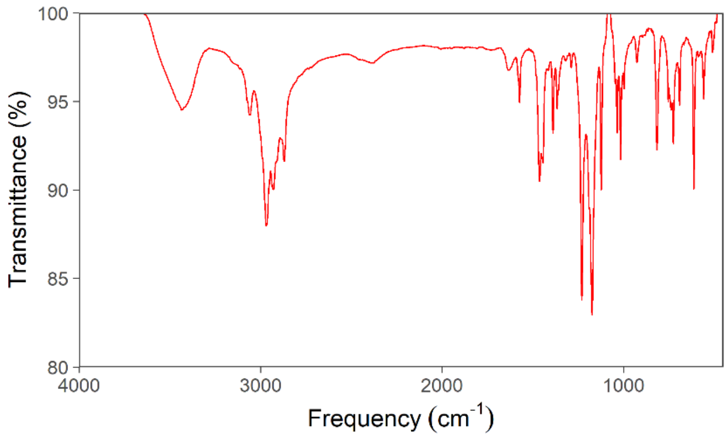

Figure 57. Experimental IR spectrum (KBr pellet) of **7b**.

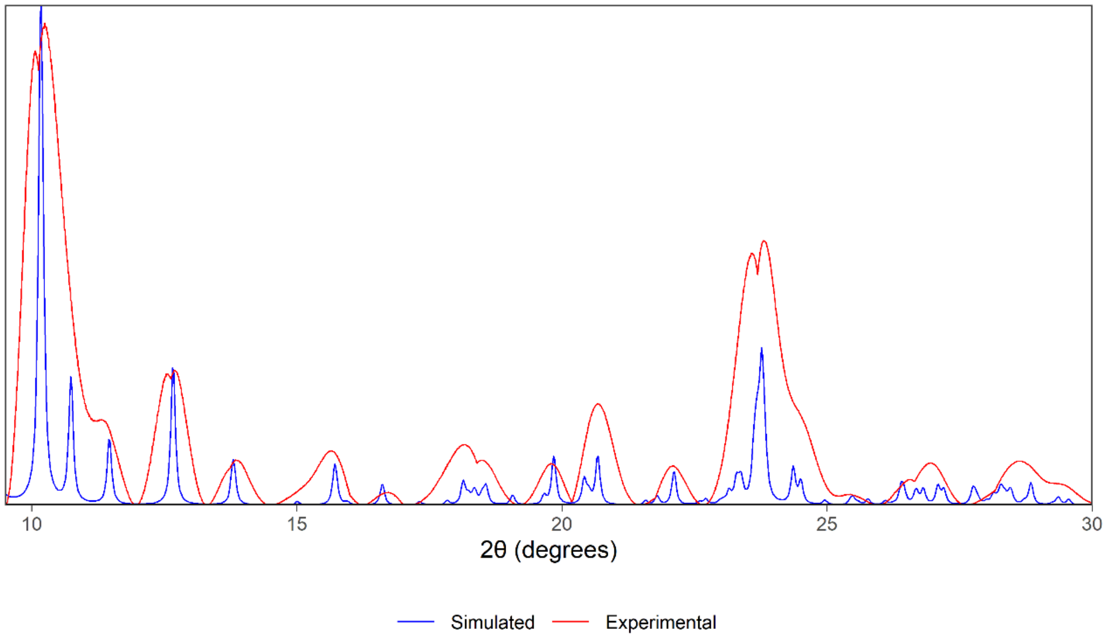

Figure 58. Simulated and experimental PXRD diffractogram of **7b**.

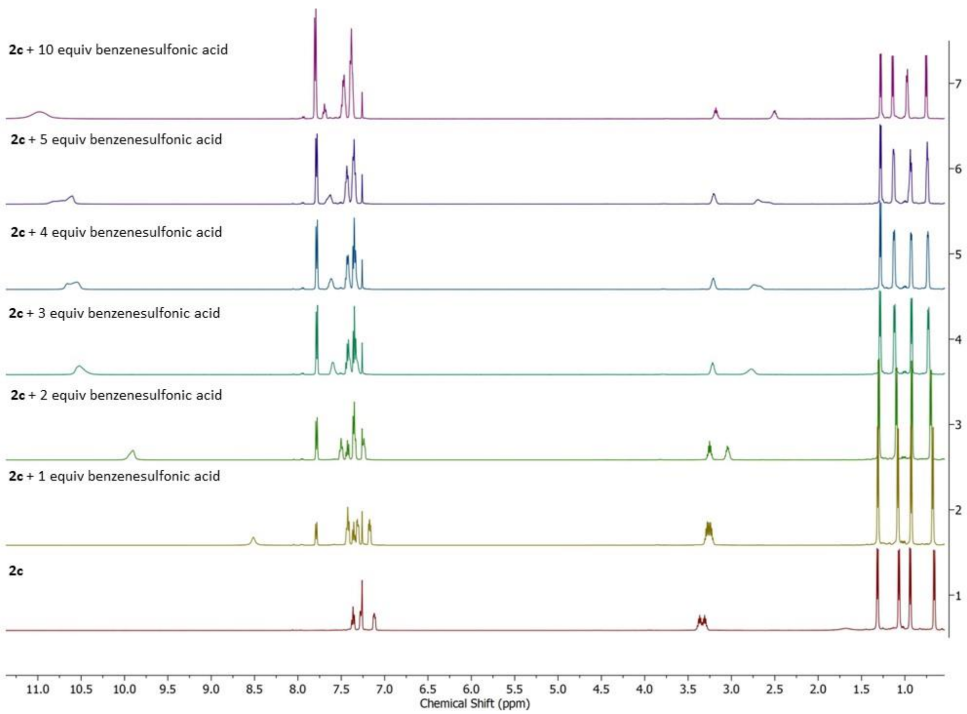

Figure 59. Stacked plots of ^{1}H NMR spectra ($CDCl_3$, 500 MHz) of **2c** with 0, 1, 2, 3, 4, 5, and 10 equivalents of benzenesulfonic acid (*bottom* to *top*).

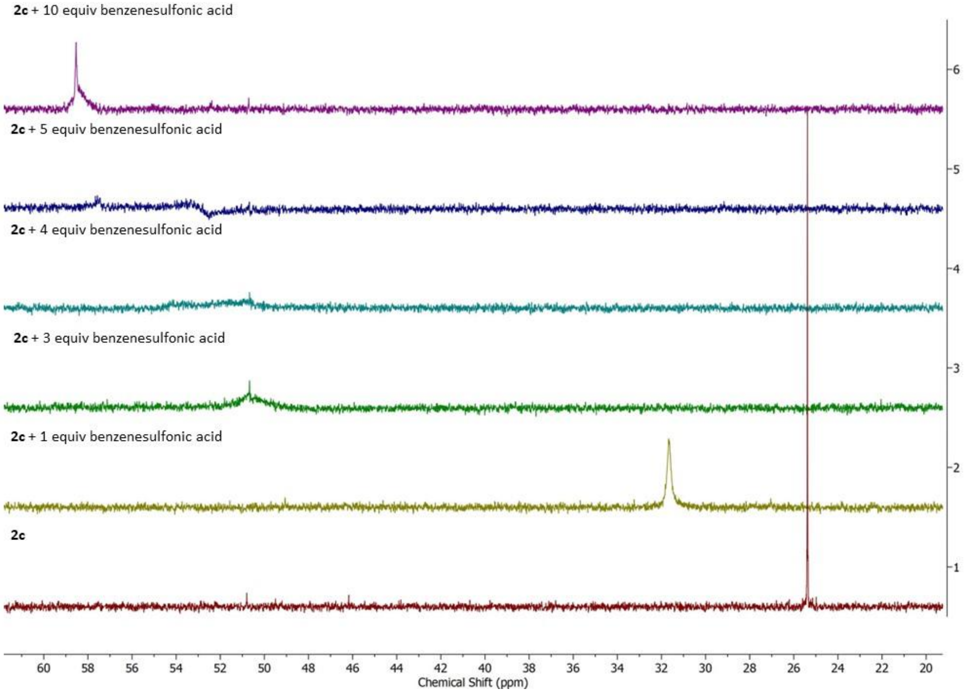

Figure 60. Stacked plots of $^{31}P\{^{1}H\}$ NMR spectra ($CDCl_3$, 202 MHz) of **2c** with 0, 1, 3, 4, 5, and 10 equivalents of benzenesulfonic acid (*bottom* to *top*).

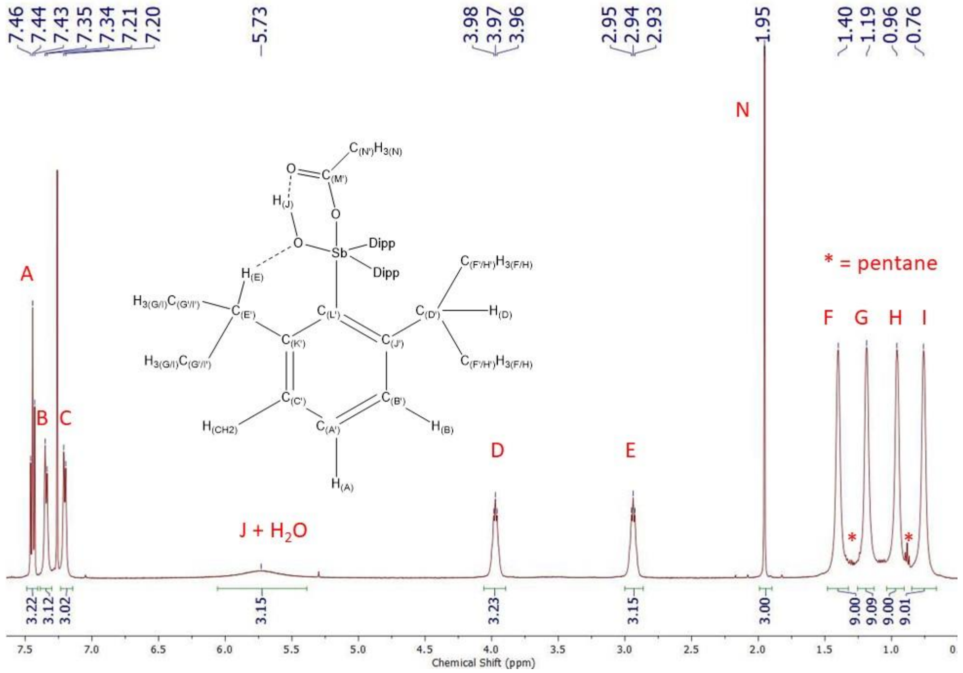

Figure 61. ^{1}H NMR spectrum ($CDCl_3$, 500 MHz) of **8** at room temperature.

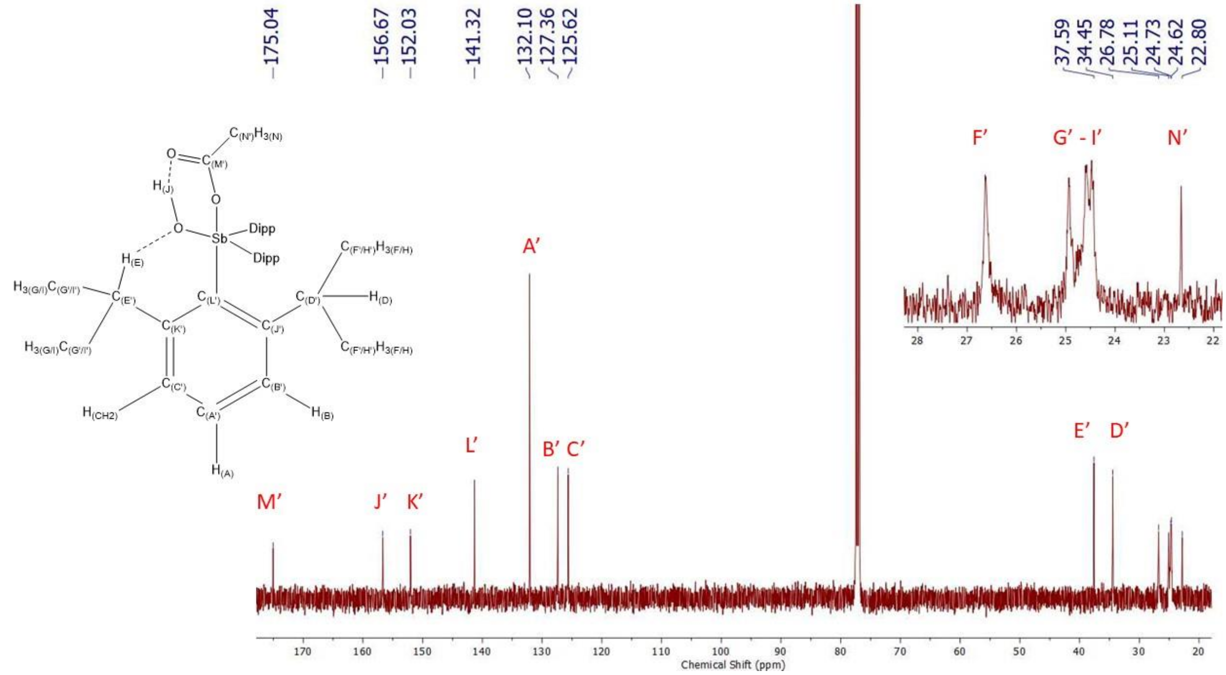

Figure 62. $^{13}C\{^{1}H\}$ NMR spectrum ($CDCl_3$, 125 MHz) of **8** at room temperature.

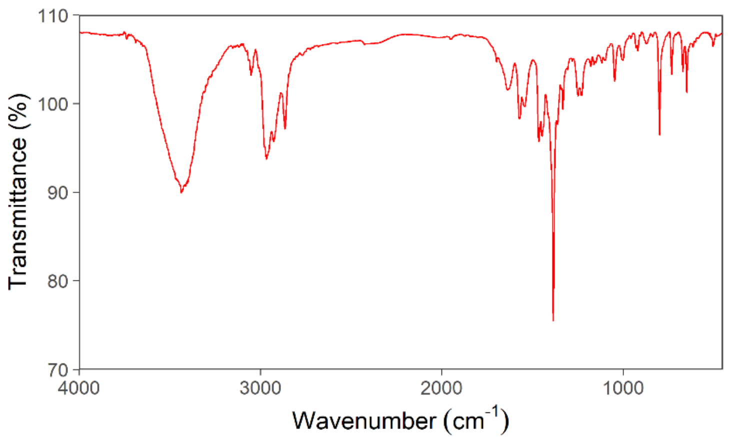

Figure 63. Experimental IR spectrum (KBr pellet) of **8** (ν_{SbO} = 649 cm^{-1}).

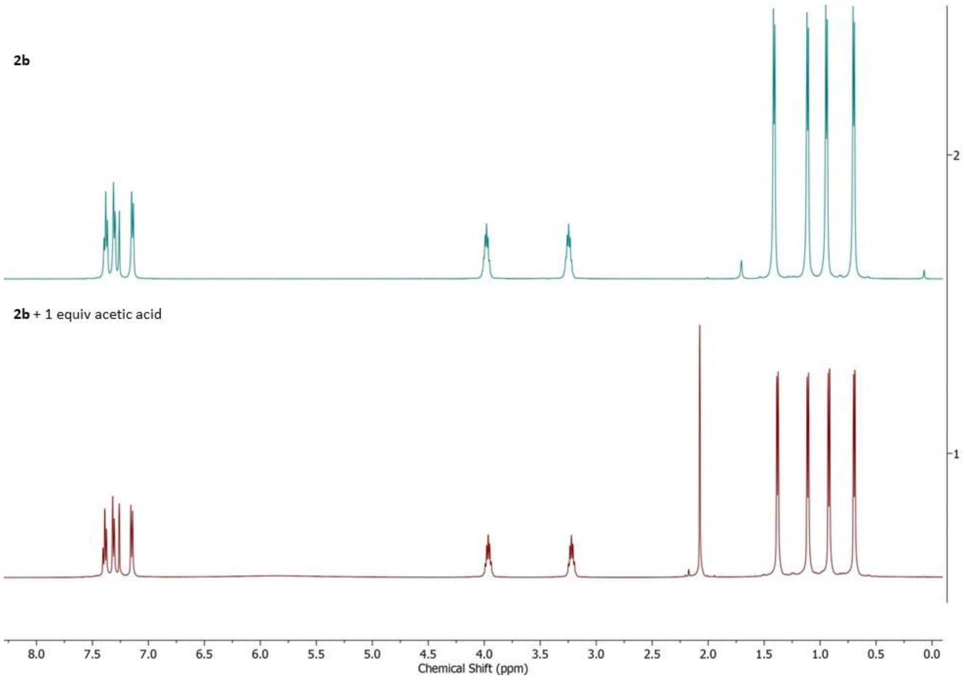

Figure 64. ^{1}H NMR spectra ($CDCl_3$, 500 MHz) of **2b** (*top*) and a 1:1 mixture of **2b** and acetic acid (*bottom*) at room temperature.

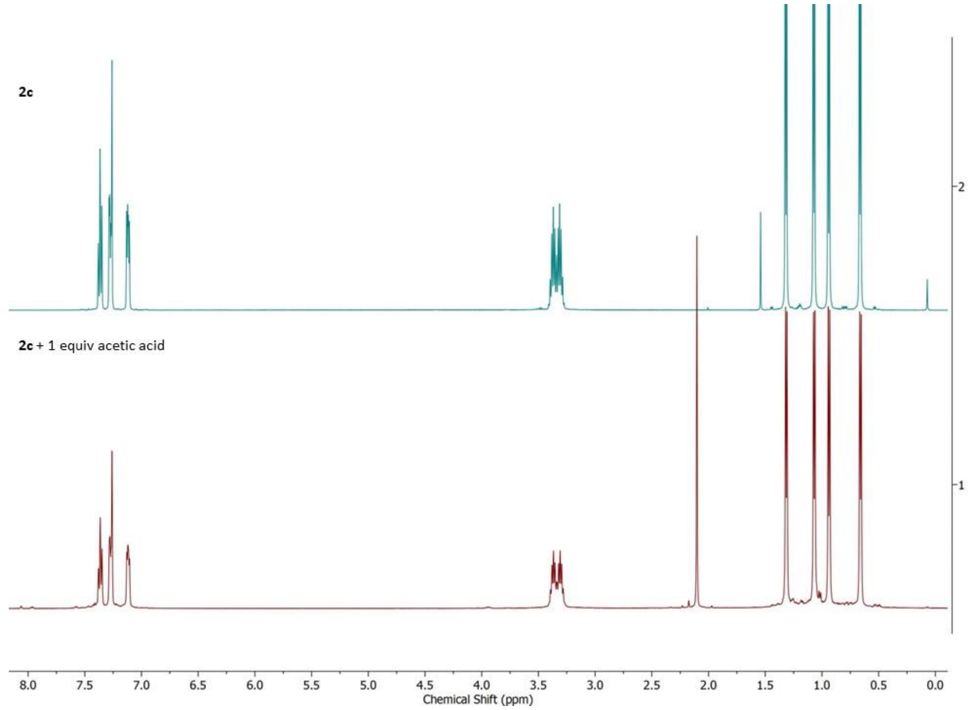

Figure 65. ^{1}H NMR spectra ($CDCl_3$, 500 MHz) of **2c** (*top*) and a 1:1 mixture of **2c** and acetic acid (*bottom*) at room temperature.

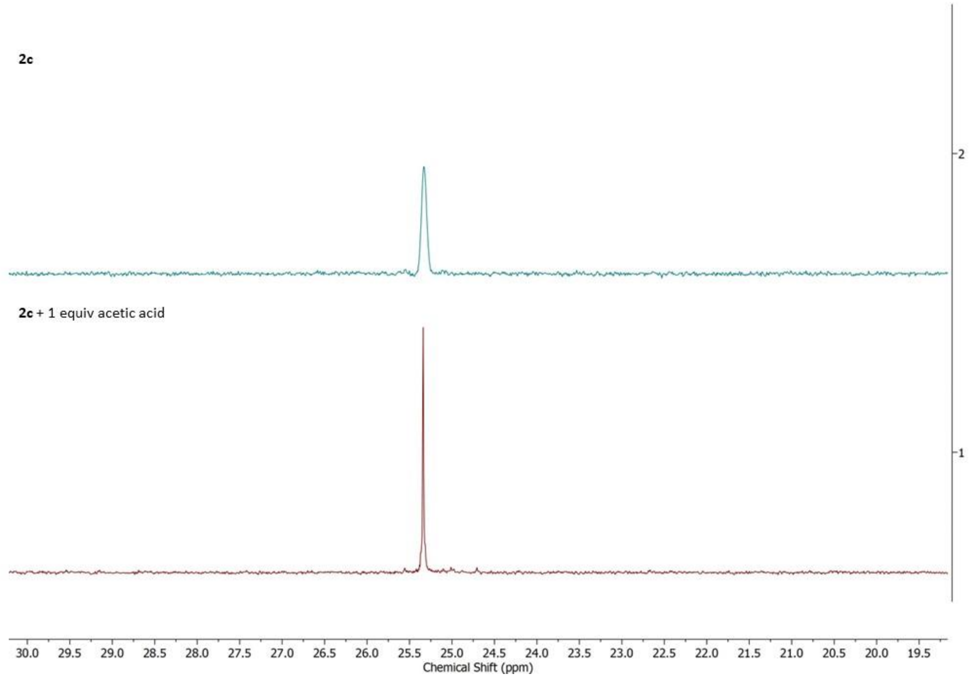

Figure 66. $^{31}P\{^{1}H\}$ NMR spectra ($CDCl_3$, 202 MHz) of **2c** (*top*) and a 1:1 mixture of **2c** and acetic acid (*bottom*) at room temperature.

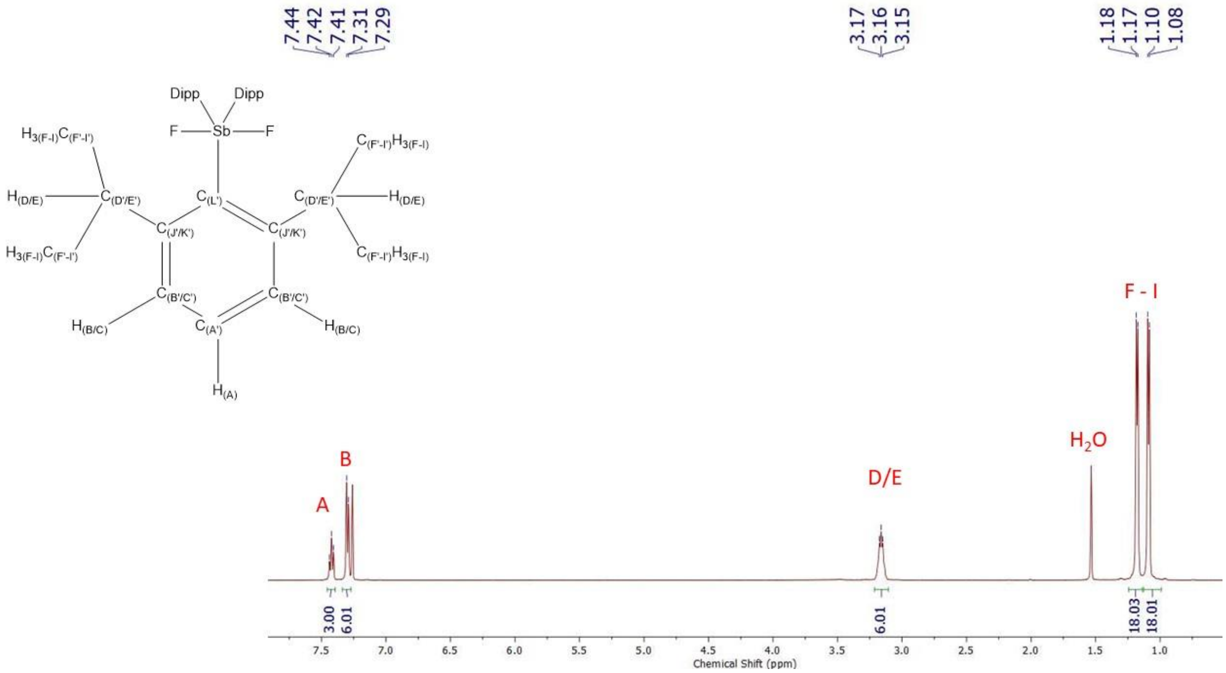

Figure 67. ^{1}H NMR spectrum ($CDCl_3$, 500 MHz) of **9** at room temperature.

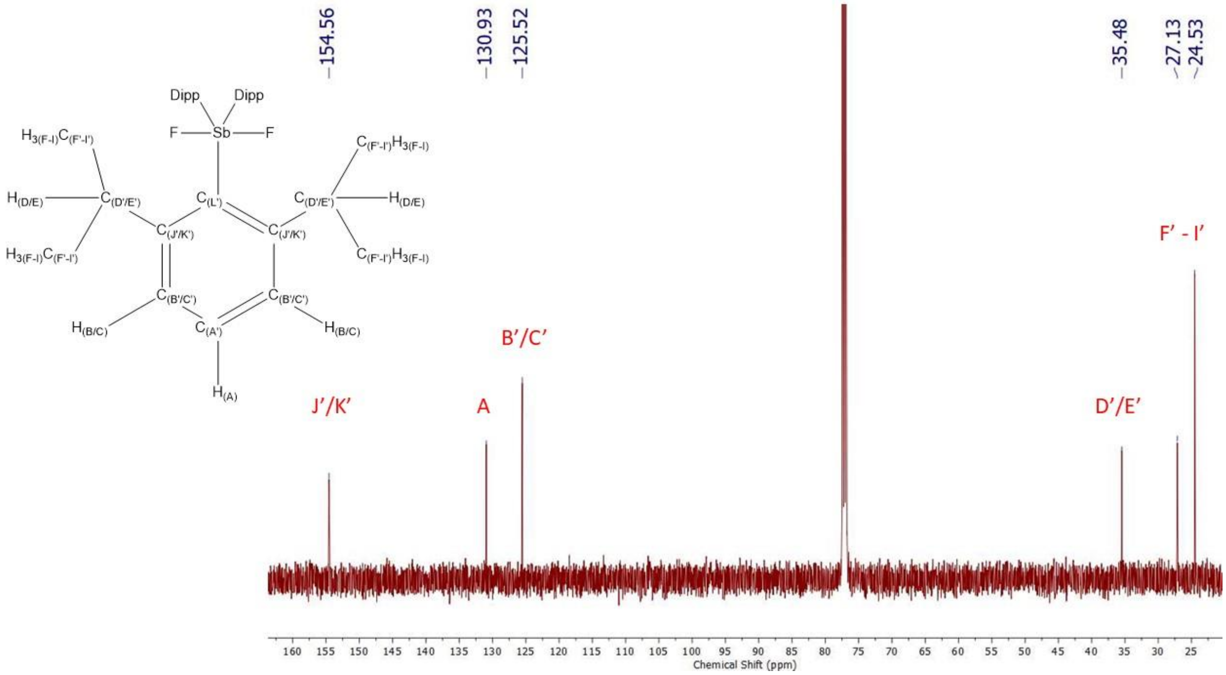

Figure 68. $^{13}C\{^{1}H\}$ NMR spectrum ($CDCl_3$, 125 MHz) of **9** at room temperature.

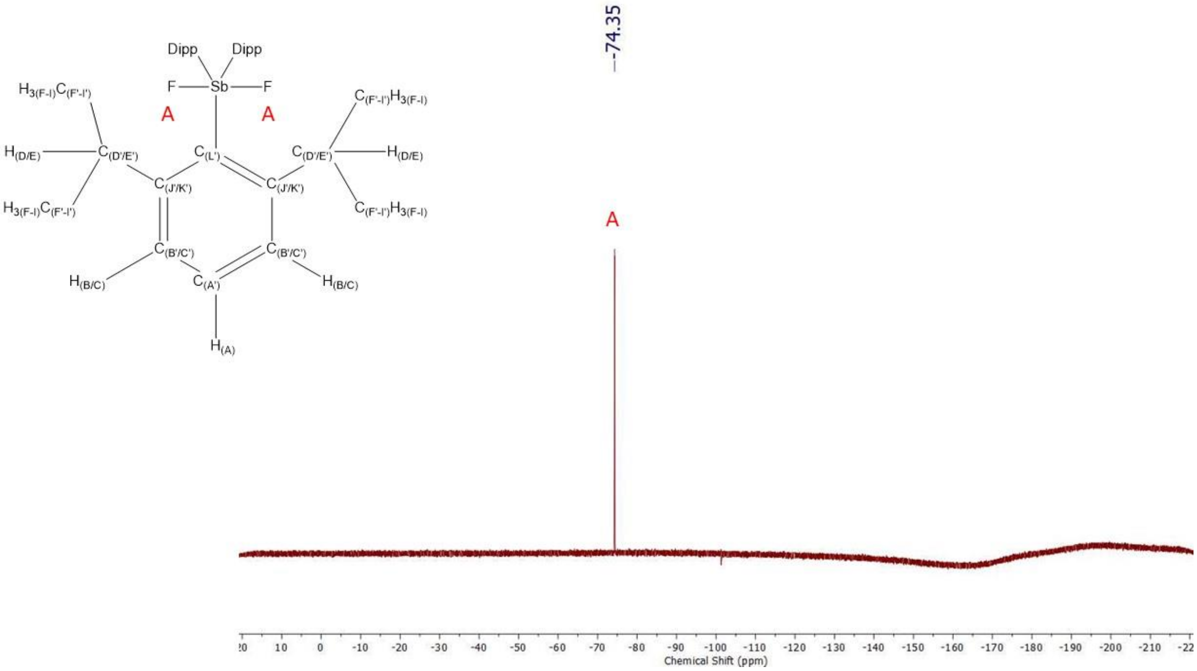

Figure 69. ^{19}F NMR spectrum ($CDCl_3$, 470 MHz) of **9** at room temperature.

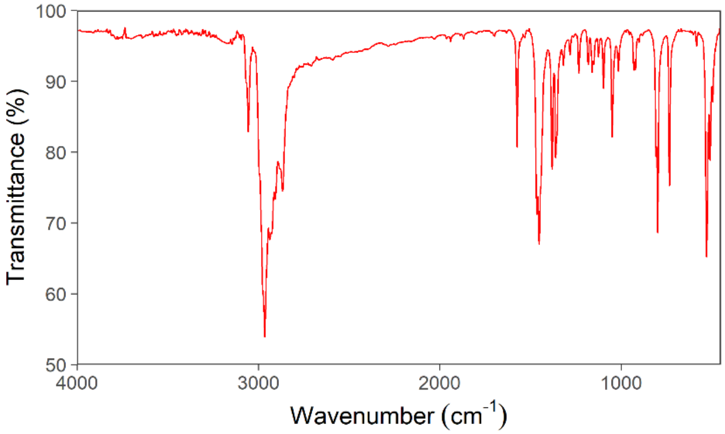

Figure 70. Experimental IR spectrum (KBr pellet) of **9** (ν_{SbF} = 528 cm^{-1}).

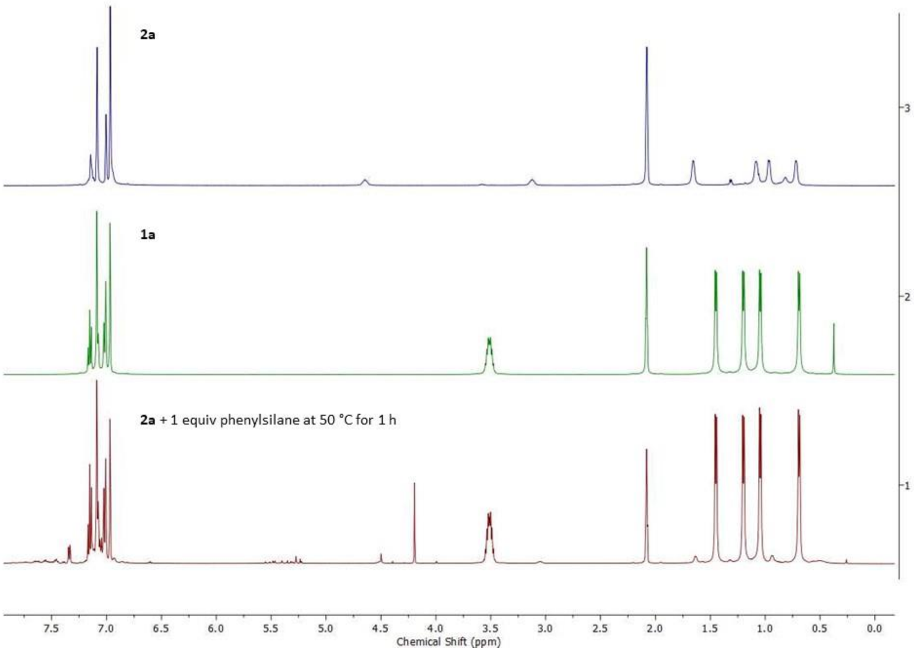

Figure 71. ^{1}H NMR spectra (d_8-toluene, 500 MHz) of **2a** (*top*), **1a** (*middle*), and a reaction mixture of phenylsilane and **2a** (1:1 mixture) after 1 h at 50 °C (*bottom*), collected at room temperature.

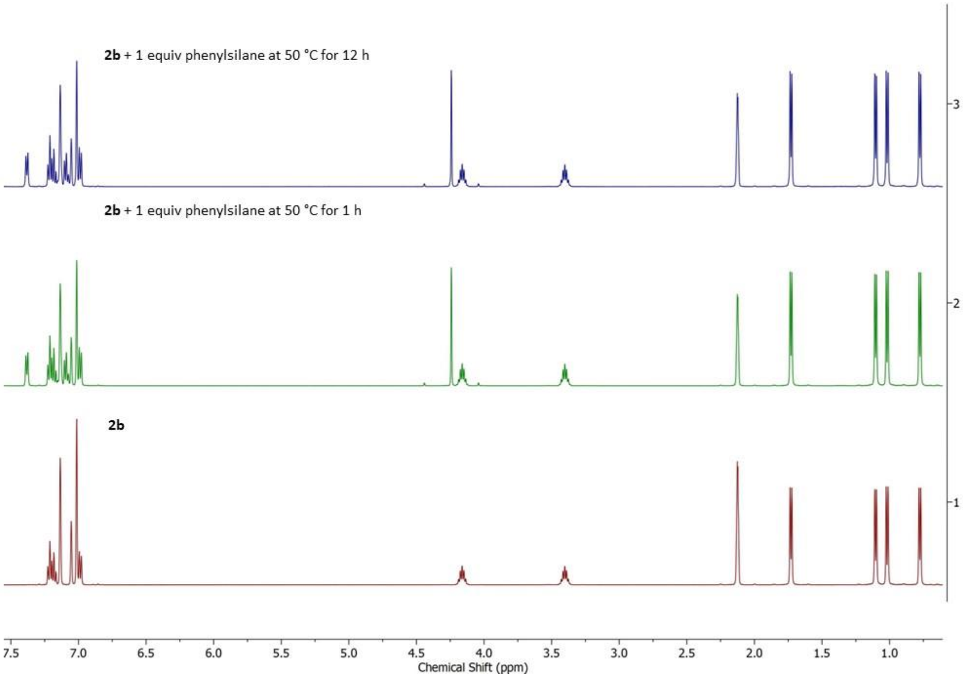

Figure 72. ^{1}H NMR spectra (d_8-toluene, 500 MHz) of **2b** (*bottom*), and a mixture of phenylsilane and **2b** (1:1 mixture) after 1 h (*middle*) and 12 h (*top*) at 50 °C, collected at room temperature.

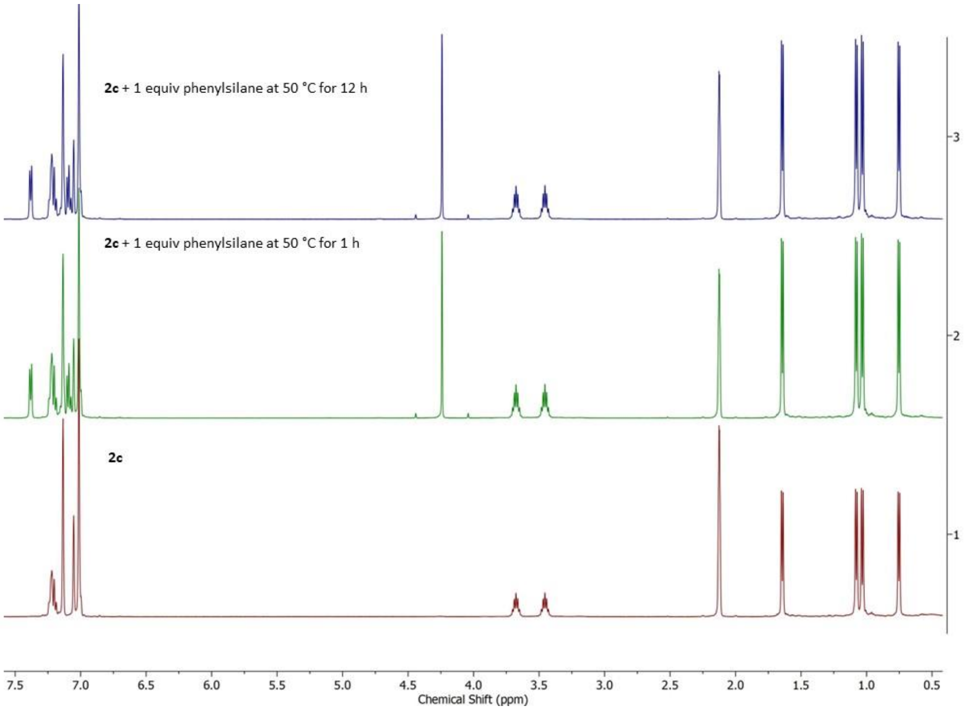

Figure 73. ^{1}H NMR spectra (d_8-toluene, 500 MHz) of **2c** (*bottom*), and a mixture of phenylsilane and **2c** (1:1 mixture) after 1 h (*middle*) and 12 h (*top*) at 50 °C, collected at room temperature.

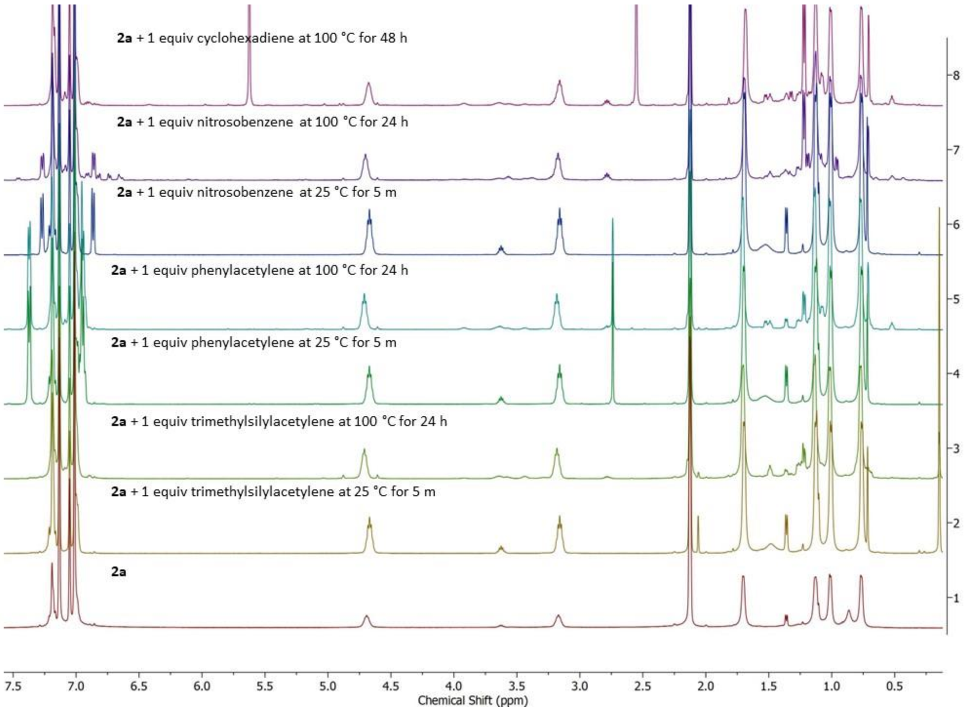

Figure 74. ^{1}H NMR spectra (d_8-toluene, 500 MHz) of **2a** and mixtures of 1 equivalent trimethylsilylacetylene, phenylacetylene, nitrosobenzene, and cyclohexadiene (*bottom* to *top*) at different time points.

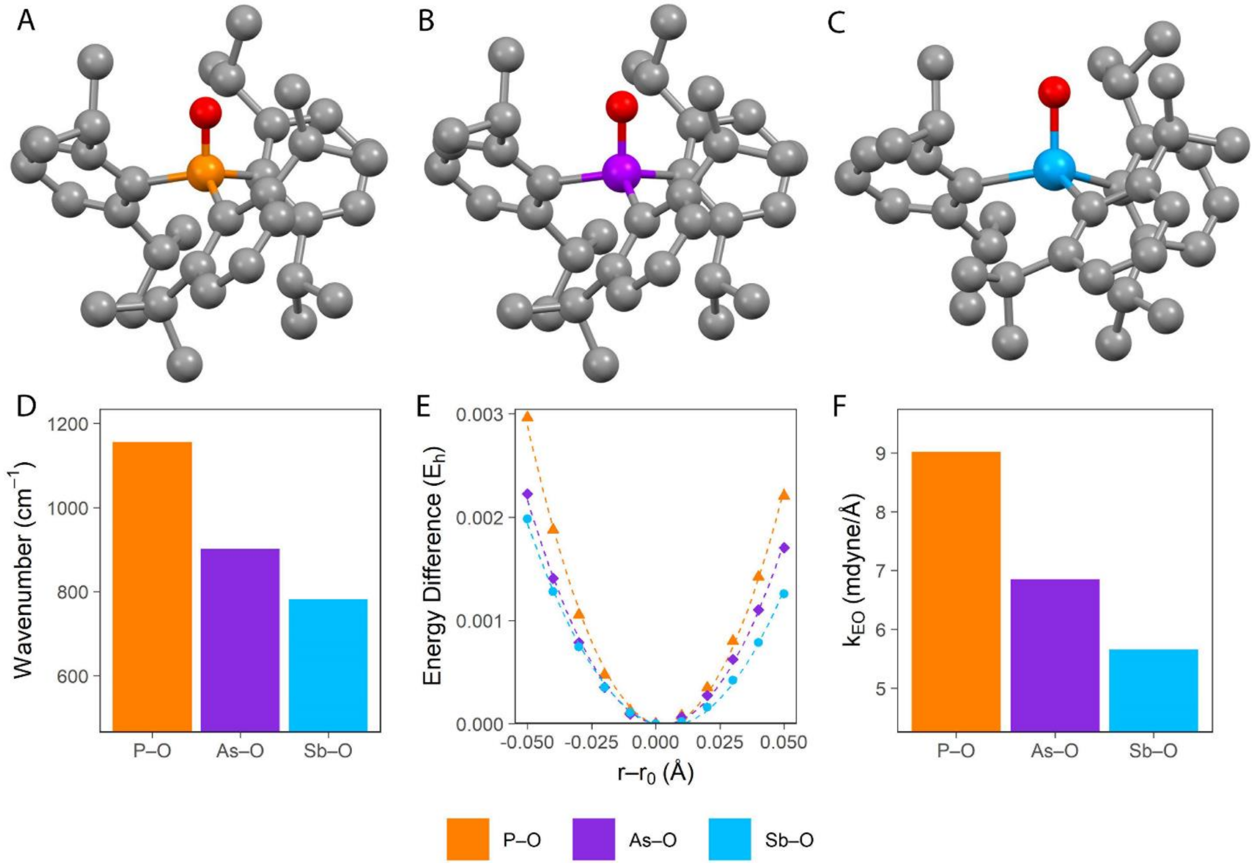

Figure 75. Geometry-optimized (PBE0/def2-TZVPP) structures of (A) **2c**, (B) **2b**, and (C) **2a** (H atoms are omitted for clarity). (D) Calculated vibrational frequency for the nominal Pn^{+}–O^{-} stretching mode. I Unrelaxed surface scan (DKH-PBE0/old-DKH-TZVPP) energies plotted as a function of Pn^{+}–O^{-} bond contraction/elongation from the equilibrium bond length (r_0). Dashed lines represent the best quadratic fit to the points. (F) Force constants for the Pn^{+}–O^{-} stretching modes obtained from the quadratic fit shown in panel (E). Force constants shown here and those obtained from diagonalization of the Hessian matrix with respect to potential energy are tabulated in Supplementary Table 17.

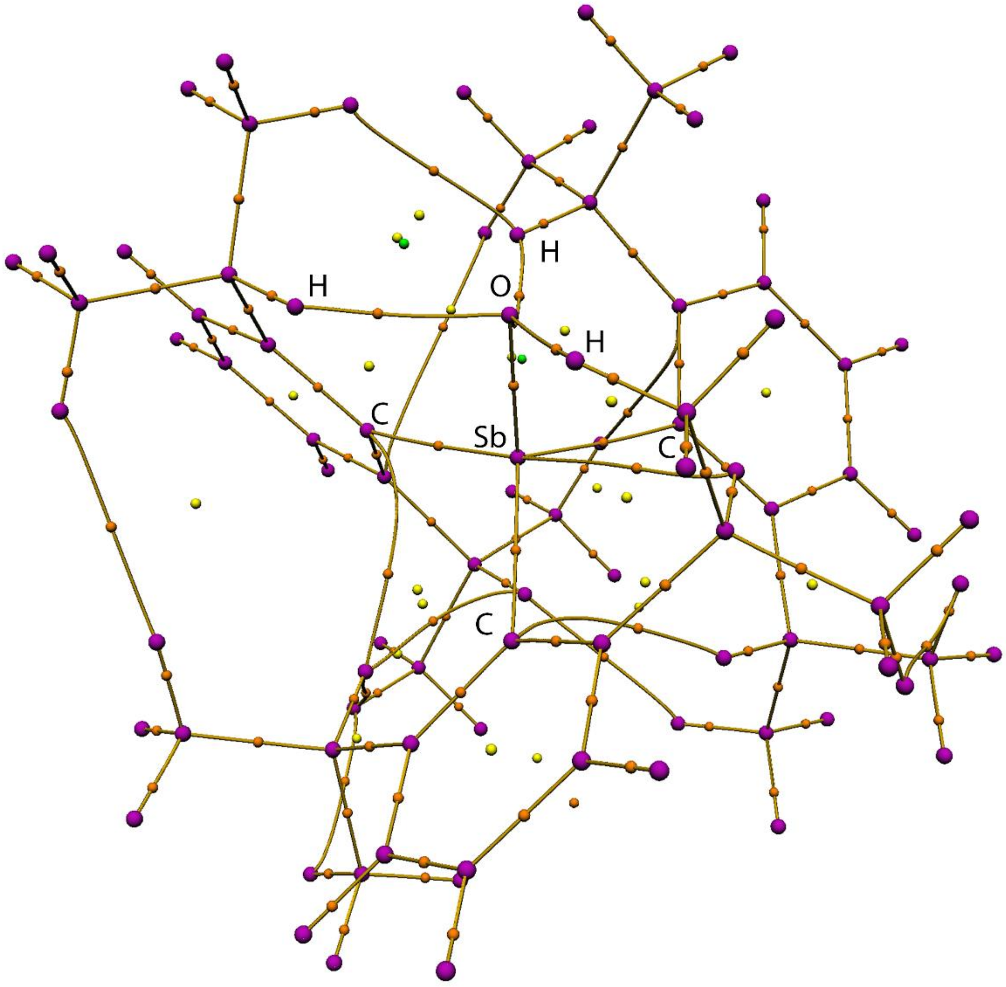

Figure 76. Molecular graph of **2a** derived from theoretical electron density (DKH-PBE0/old-DKH-TZVPP //PBE0/def2-TZVPP) depicting critical points and bond paths between (3, –3) and (3, –1) critical points. Color code: (3, –3) purple, (3, –1) orange, (3, +1) yellow, (3, +3) green.

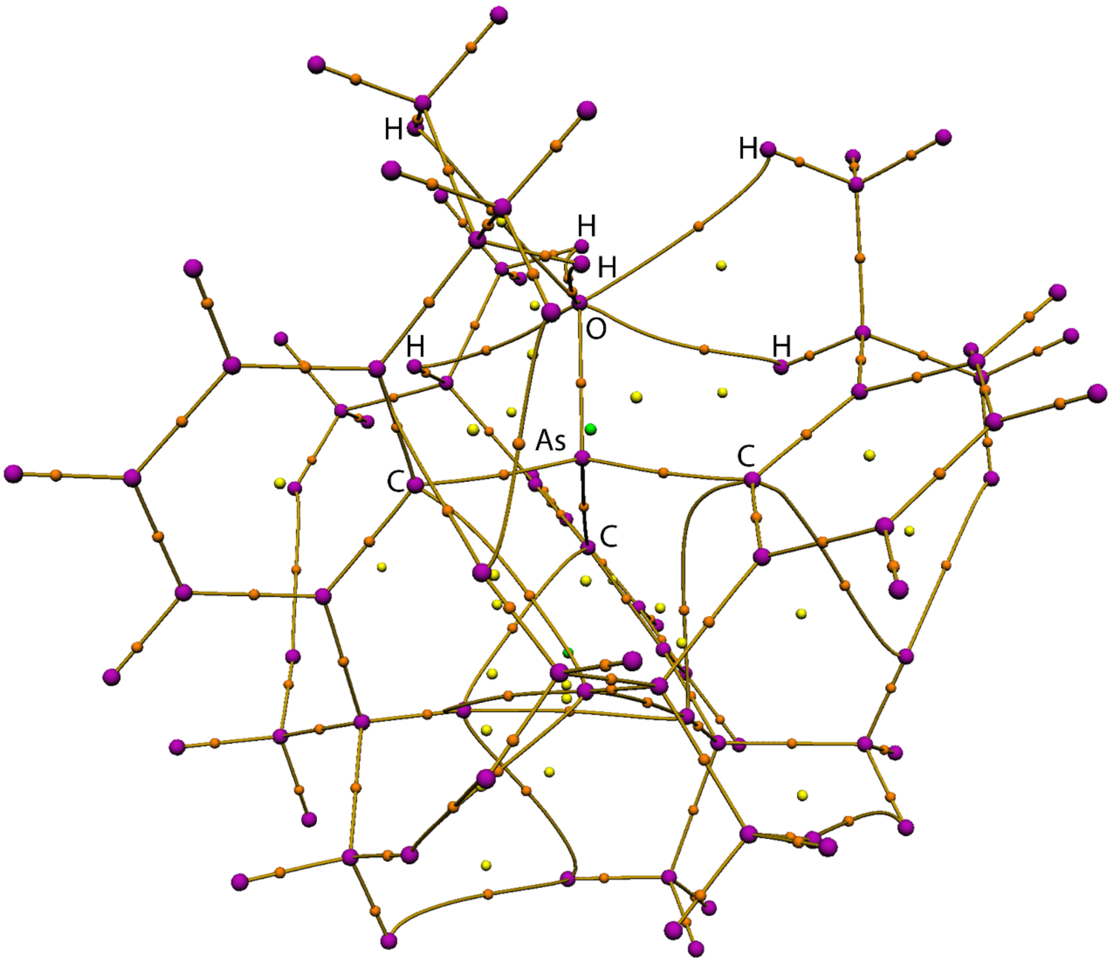

Figure 77. Molecular graph of **2b** derived from theoretical electron density (DKH-PBE0/old-DKH-TZVPP //PBE0/def2-TZVPP) depicting critical points and bond paths between (3, –3) and (3, –1) critical points. Color code: (3, –3) purple, (3, –1) orange, (3, +1) yellow, (3, +3) green

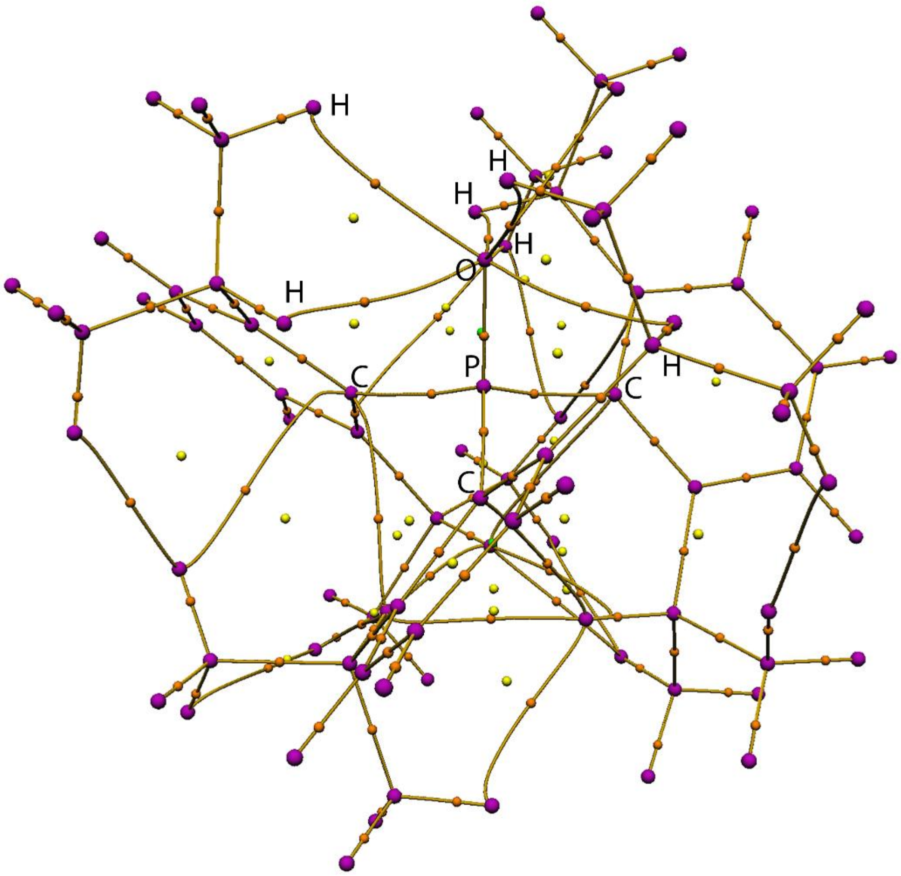

Figure 78. Molecular graph of **2c** derived from theoretical electron density (DKH-PBE0/old-DKH-TZVPP //PBE0/def2-TZVPP) depicting critical points and bond paths between (3, –3) and (3, –1) critical points. Color code: (3, –3) purple, (3, –1) orange, (3, +1) yellow, (3, +3) green.

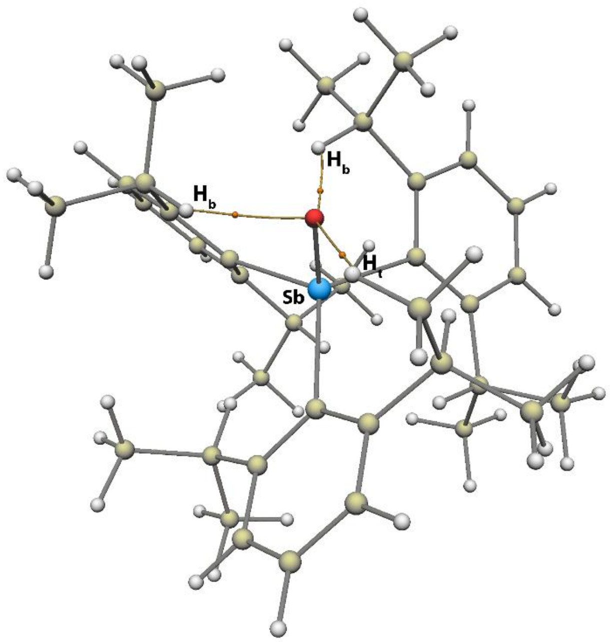

Figure 79. Intramolecular O···H bond paths and bond critical points in **2a** derived from theoretical electron density (DKH-PBE0/old-DKH-TZVPP//PBE0/def2-TZVPP) overlaid on the optimized molecular structure of **2a**. Color code: O red, C tan, H white, Sb teal, bond grey, bond path yellow, (3, –1) critical point orange. Benzylic H atoms are denoted "H_b" and terminal H atoms are denoted "H_t."

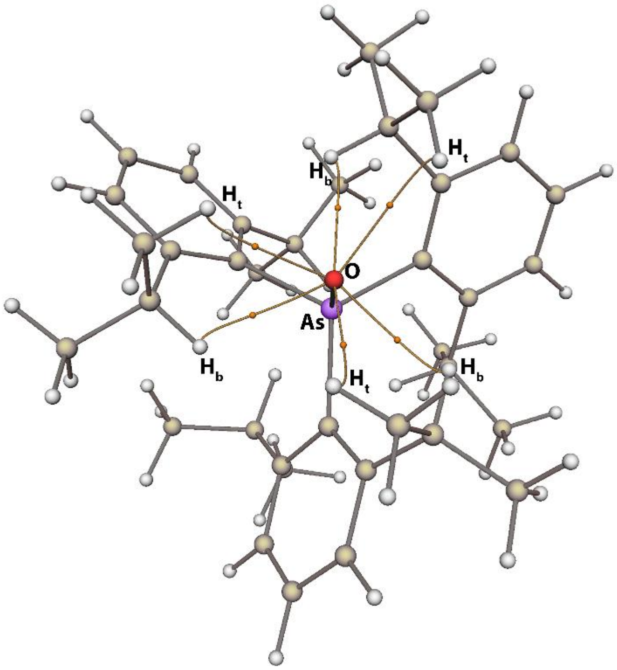

Figure 80. Intramolecular O···H bond paths and bond critical points in **2b** derived from theoretical electron density (DKH-PBE0/old-DKH-TZVPP//PBE0/def2-TZVPP) overlaid on the optimized molecular structure of

2b. Color code: O red, C tan, H white, As violet, bond grey, bond path yellow, (3, –1) critical point orange. Benzylic H atoms are denoted "H_b" and terminal H atoms are denoted "H_t."

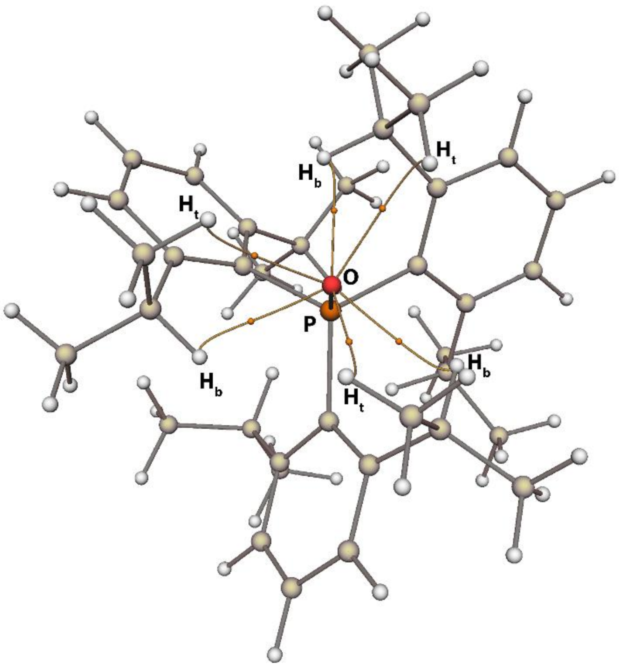

Figure 81. Intramolecular O···H bond paths and bond critical points in **2c** derived from theoretical electron density (DKH-PBE0/old-DKH-TZVPP//PBE0/def2-TZVPP) overlaid on the optimized molecular structure of **2c**. Color code: O red, C tan, H white, P orange, bond grey, bond path yellow, (3, –1) critical point orange. Benzylic H atoms are denoted "H_b" and terminal H atoms are denoted "H_t."

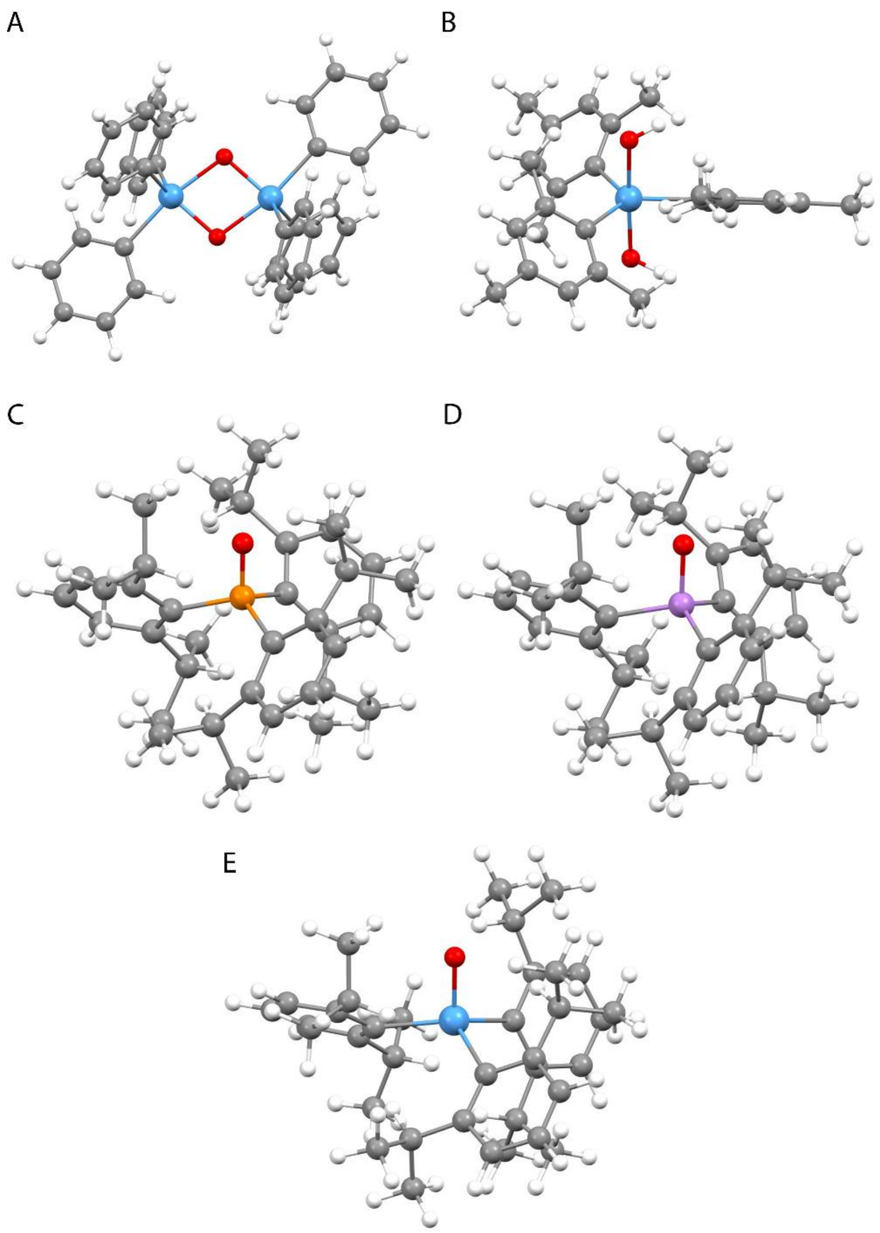

Figure 82. Ball-and-stick representations of atomic coordinates of molecules used in theoretical studies. Geometry optimized (PBE0/def2-TZVPP) structure of (A) **A**, (B) **B**, (C) **2c**, (D) **2b**, (E) **2a**. Color code: C grey, H white, O red, Sb teal, As violet, P orange.

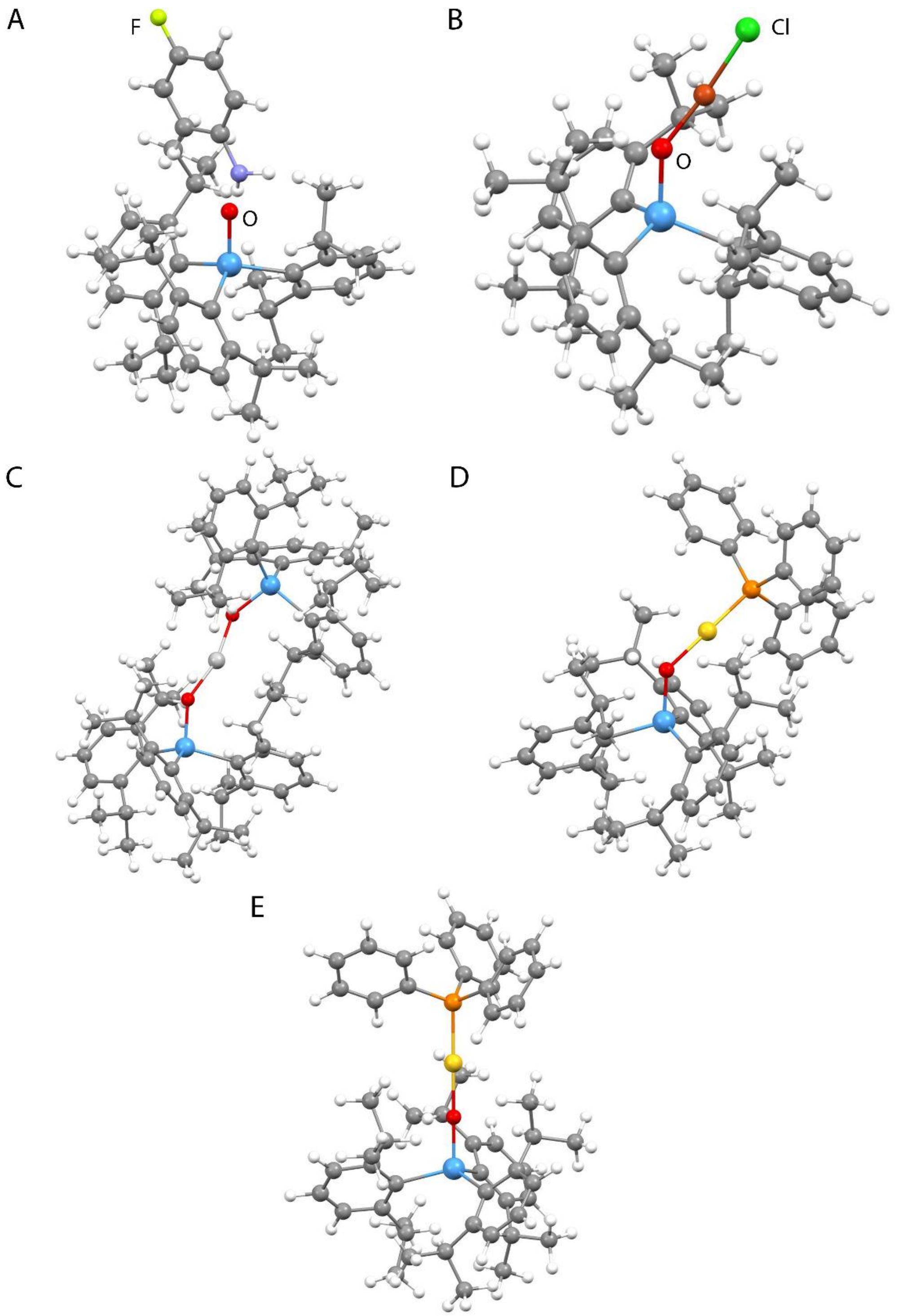

Figure 83. Ball-and-stick representations of atomic coordinates of molecules used in theoretical studies. (A) HAR model of **3**. (B) IAM model of **4** with H-bond lengths normalized to 1.089 Å. (C) Geometry optimized (BP86/def2-SVP) structure of **5** (triflate counteranion is omitted). IAM model of **6** in either the (D) triclinic polymorIor (E) rhombohedral polymorph with H-bond lengths normalized to 1.089 Å (triflate counteranion is omitted). Color code: C grey, H white, O red, Sb teal, P orange, Au gold, Ag silver, Cu brown, F yellow-green, Cl green, N light-purple.

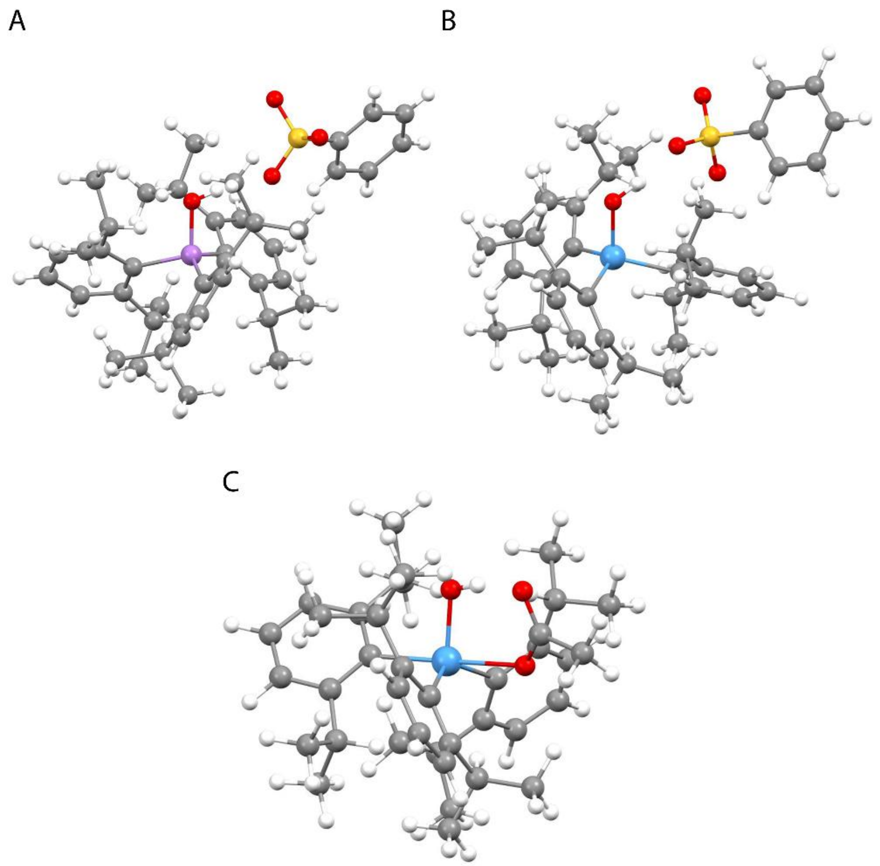

Figure 84. Ball-and-stick representations of atomic coordinates of molecules used in theoretical studies. HAR model of (A) **7b** and (B) **7a**. (C) Geometry optimized (PBE0/def2-TVPPP) structure of **8**. Color code: C grey, H white, O red, Sb teal, As violet, S yellow.

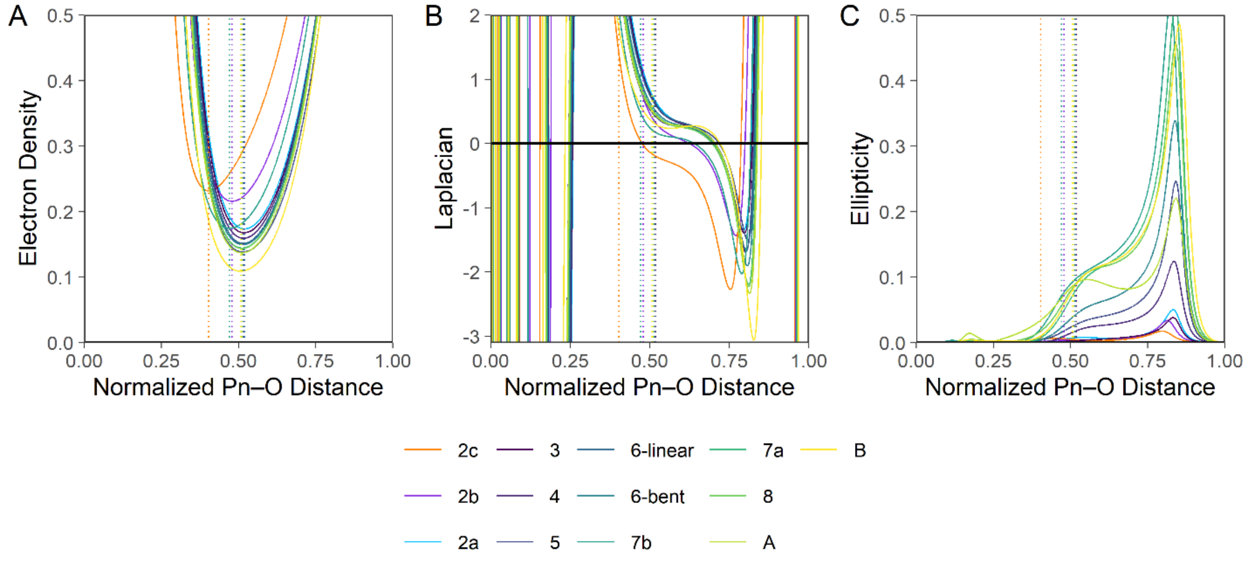

Figure 85. Plots of ρ ($e^- Å^{-3}$), $\nabla^2\rho$ ($e^- Å^{-5}$), and ε along the Pn–O interatomic vector for compounds **2a**-**8**, **A**, **B**.

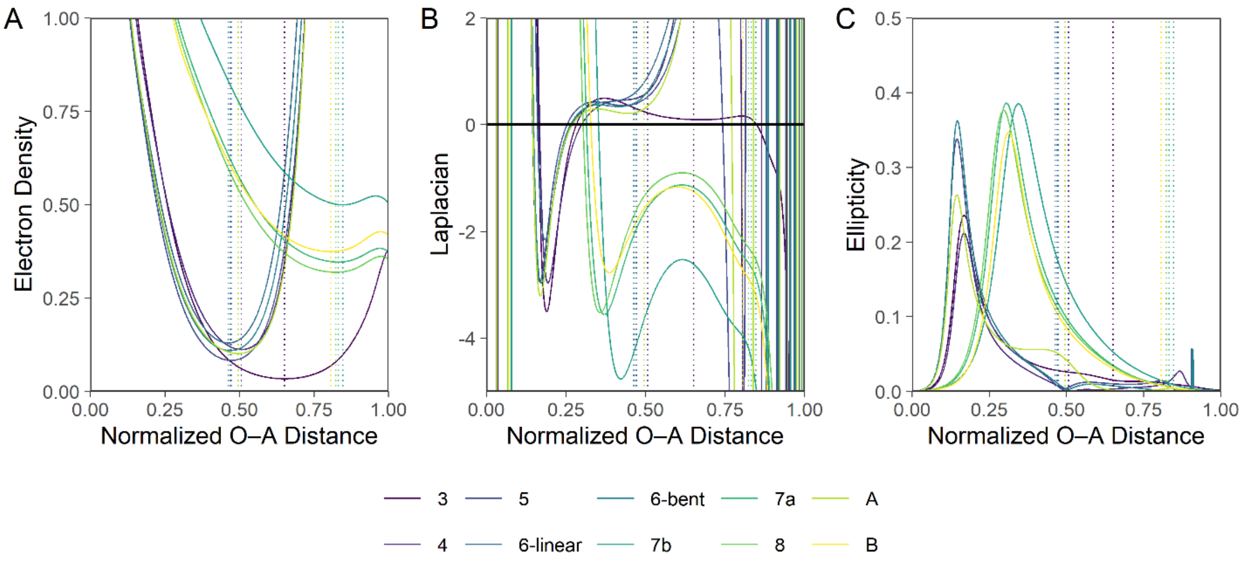

Figure 86. Plots of ρ (e⁻ Å^{-3}), ∇^2ρ (e⁻ Å^{-5}), and ε along the O–A interatomic vector for compounds **3-8, A, B** where "A" is the coordinated Lewis Acid.

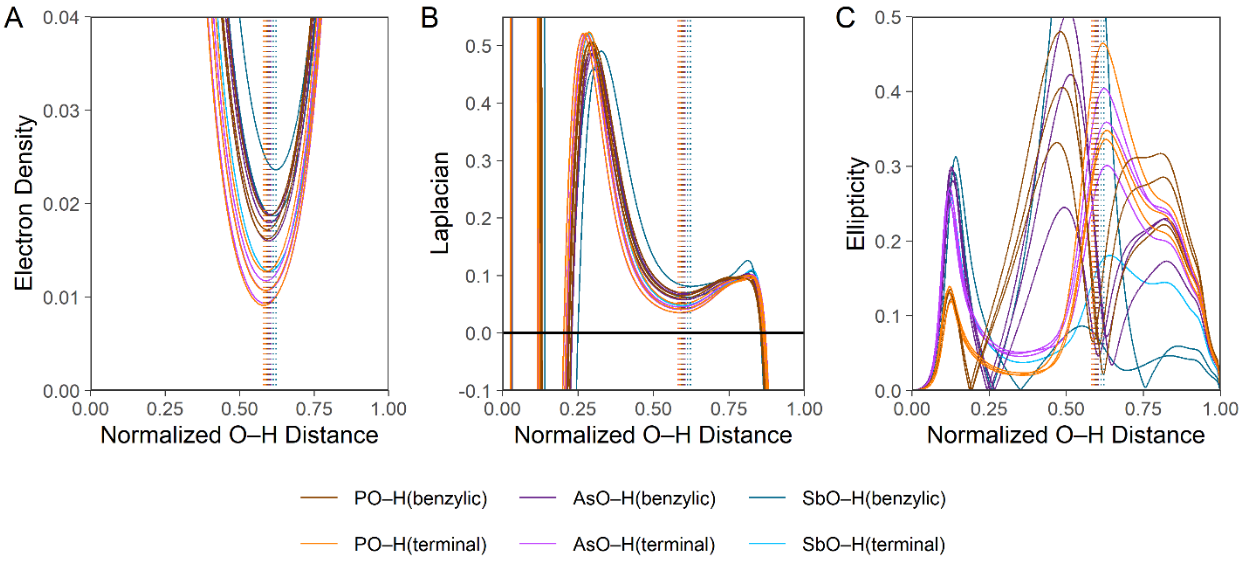

Figure 87. Plots of ρ (e⁻ Å^{-3}), ∇^2ρ (e⁻ Å^{-5}), and ε along the O···H interatomic vector for all intramolecular H-bonding interactions of **2a**-**c**.

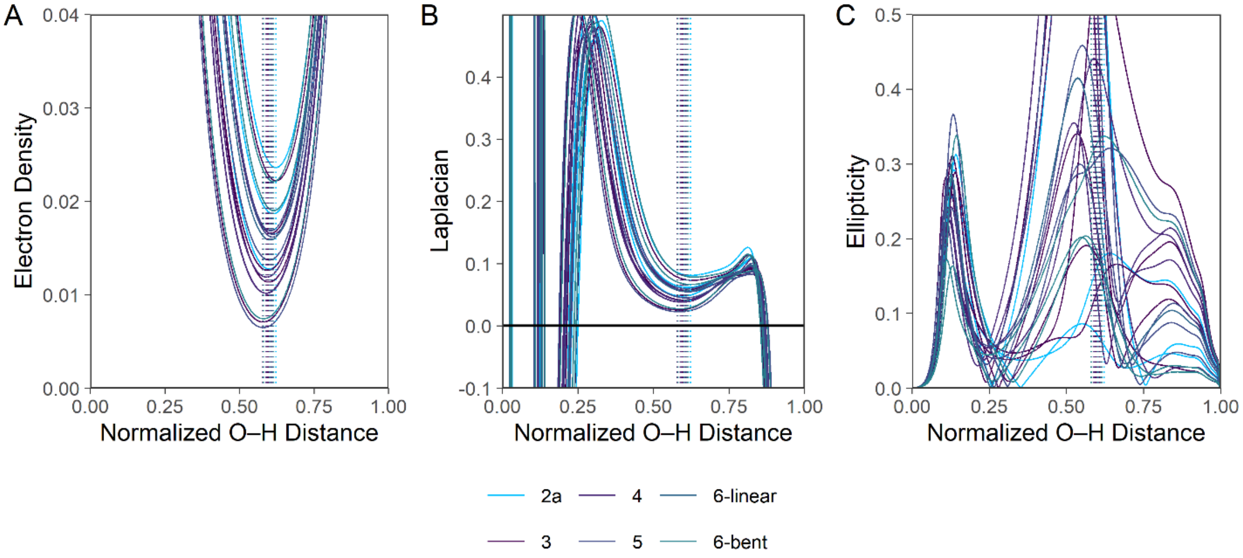

Figure 88. Plots of ρ (e^{-} $Å^{-3}$), $\nabla^2\rho$ (e^{-} $Å^{-5}$), and ε along the O···H interatomic vector for all intramolecular H-bonding interactions of **2a**, **3**, **4**, **5**, **6**-linear and **6**-bent.

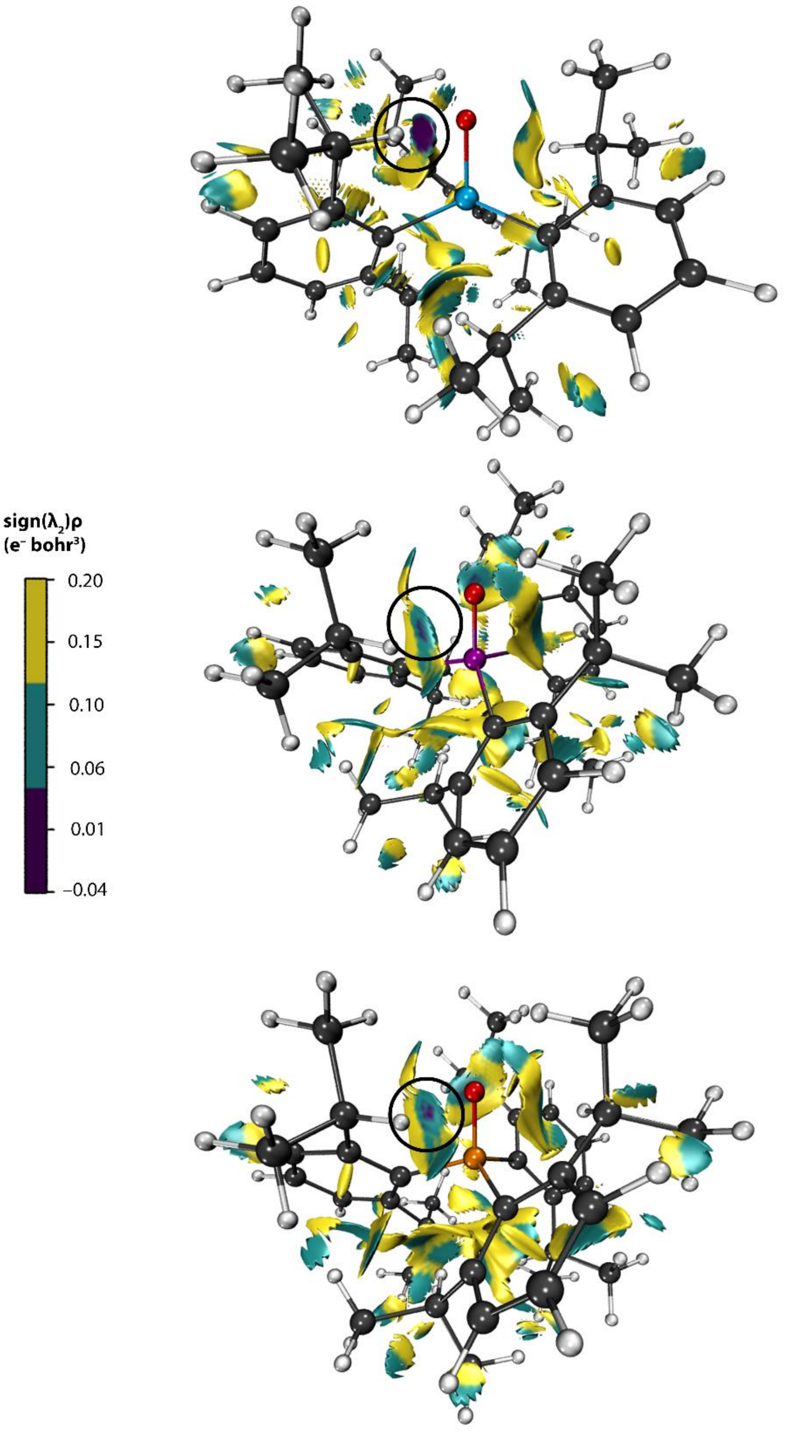

Figure 89. NCI analysis of **2a** (*top*), **2b** (*middle*), and **2c** (*bottom*) depicting reduced gradient surfaces (isovalue = 0.45 a.u.) with the function sign(λ_2)ρ, where λ_2 is the second-largest eigenvalue of the Laplacian, color-mapped on the surface. Purple is indicative of H-bonding interactions, aqua is indicative of van der Waals interactions, and yellow is indicative of steric repulsions.

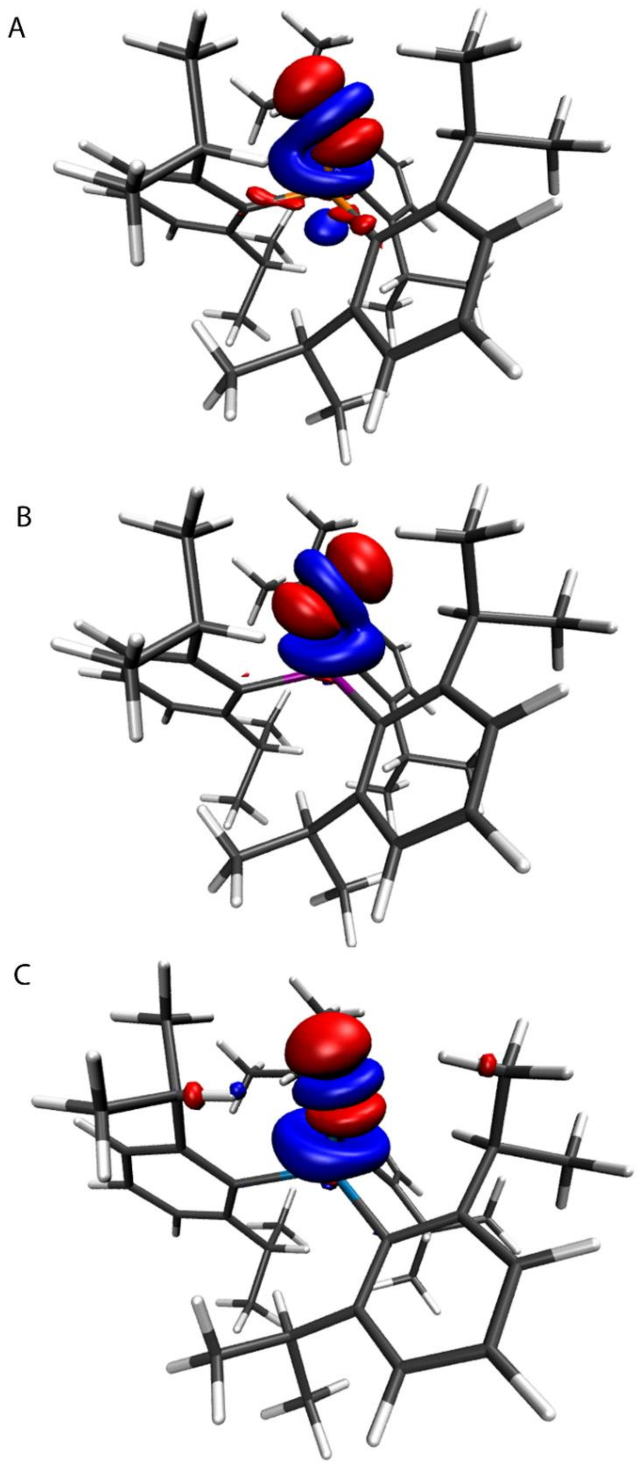

Figure 90. Deformation density (DKH-PBE0/old-DKH-TZVPP) obtained by subtracting non-interacting pnictine and O atom wavefunctions from the corresponding pnictine oxide wavefunction at the optimized geometry of the pnictine oxide (PBE0/def2-TZVPP) in (A) **2c**, (B) **2b**, (C) **2a** (isovalue = –0.006 blue, 0.006 red e^{-} $Å^{3}$). Color code: C grey, H white, O red, P orange, As purple, Sb teal.

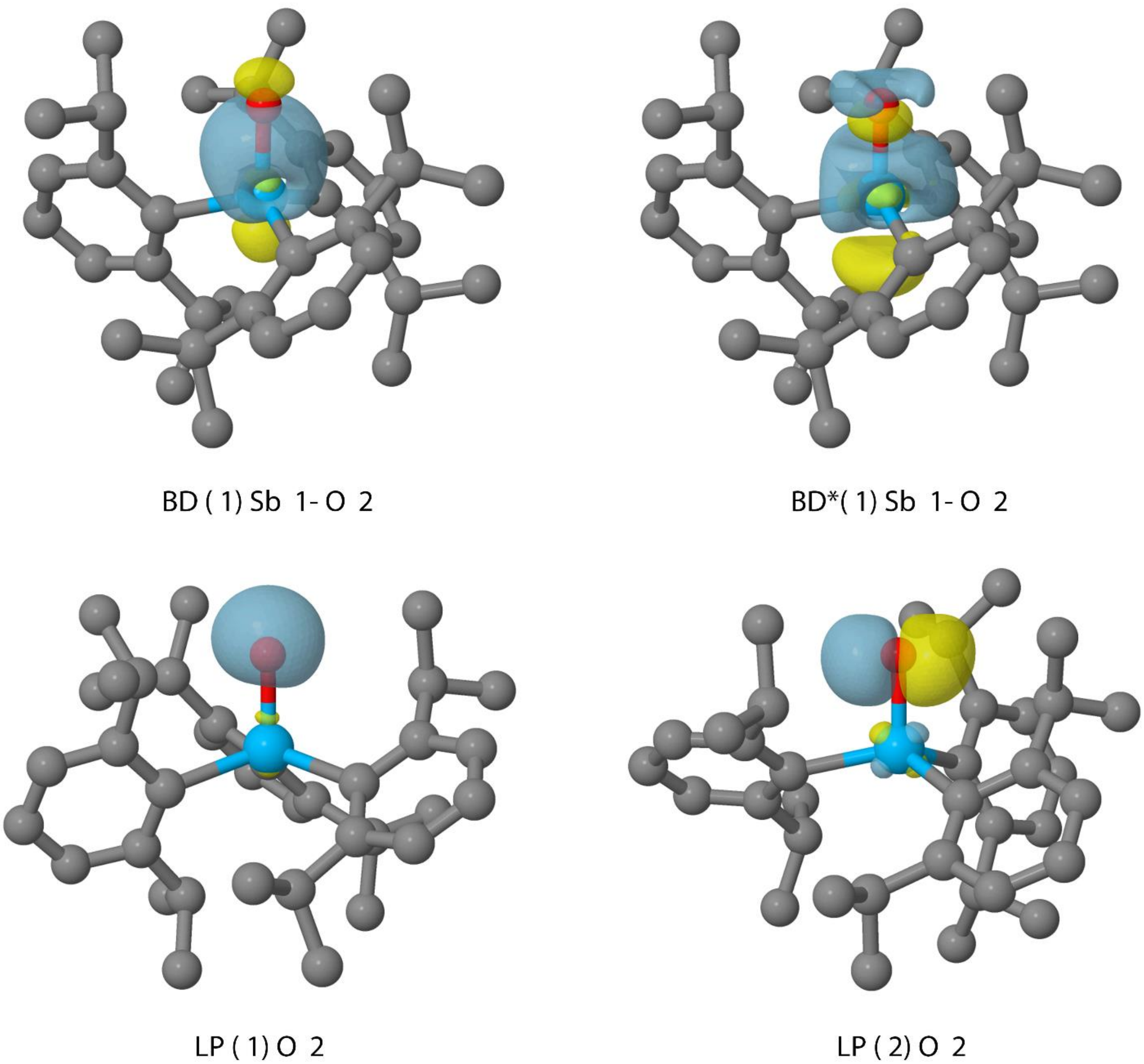

Figure 91. NLMO plots for **2a** (DKH-PBE0/old-DKH-TZVPP//PBE0/def2-TZVPP) of Sb–O bonding and antibonding orbitals and O-centered lone pairs (isosurface = 0.05).

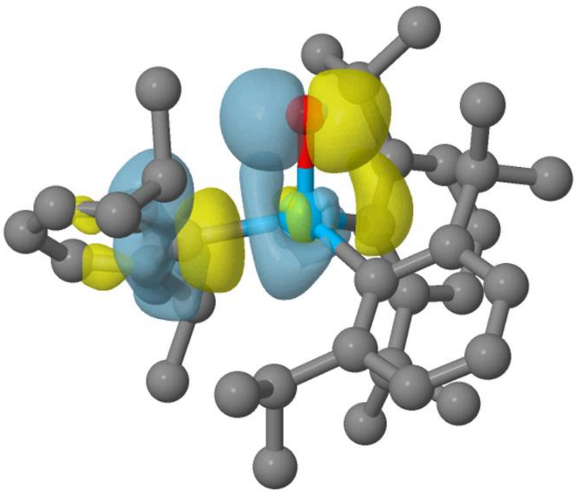

LP (2) O 2 -> BD*(1)Sb 1- C 15
11.30 kcal/mol

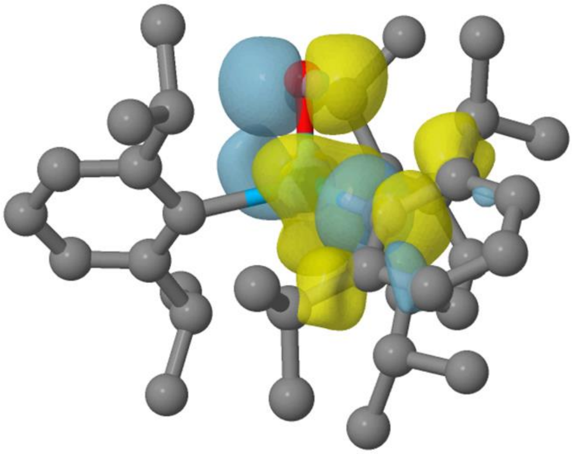

LP (2) O 2 -> BD*(1)Sb 1- C 3
4.04 kcal/mol

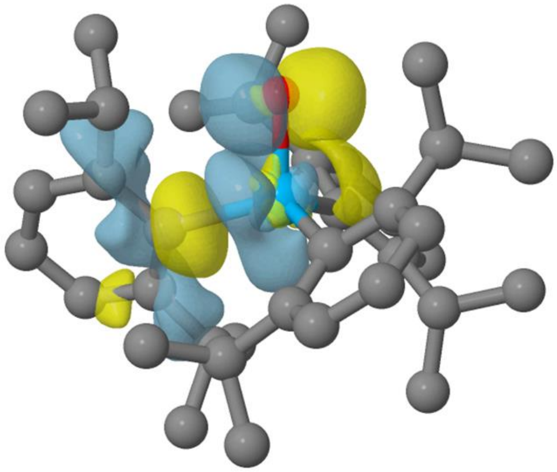

LP (3) O 2 -> BD*(1)Sb 1- C 27
9.45 kcal/mol

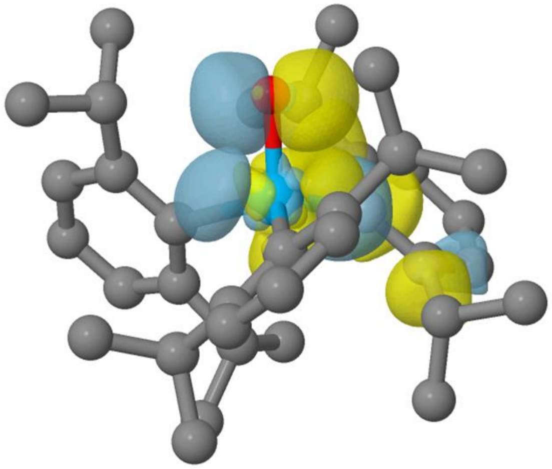

LP (3) O 2 -> BD*(1)Sb 1- C 3
8.46 kcal/mol

Figure 92. Pre-orthogonalized NLMO plots for **2a** (DKH-PBE0/old-DKH-TZVPP//PBE0/def2-TZVPP) of select O-centered lone pairs and Sb–C antibonding orbitals involved in donor-acceptor interactions (isosurface = 0.05). The energy of stabilization afforded by the interaction is displayed. H atoms are omitted for clarity.

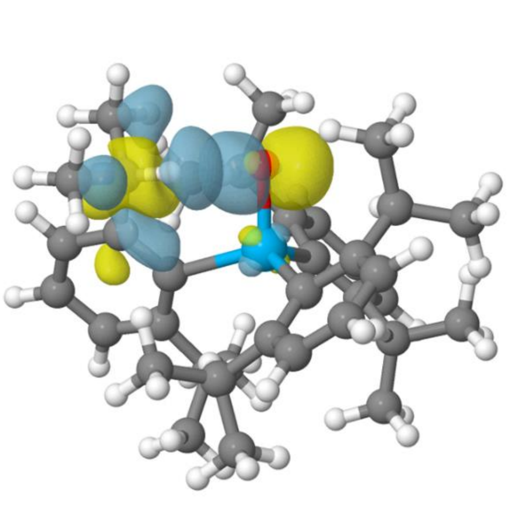

LP (3) O 2 -> BD*(1) C 33- H 73
3.38 kcal/mol
(side view)

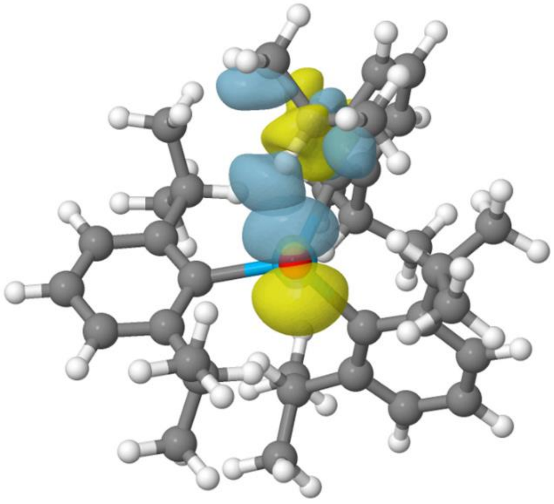

LP (3) O 2 -> BD*(1) C 33- H 73
3.38 kcal/mo
(top view)

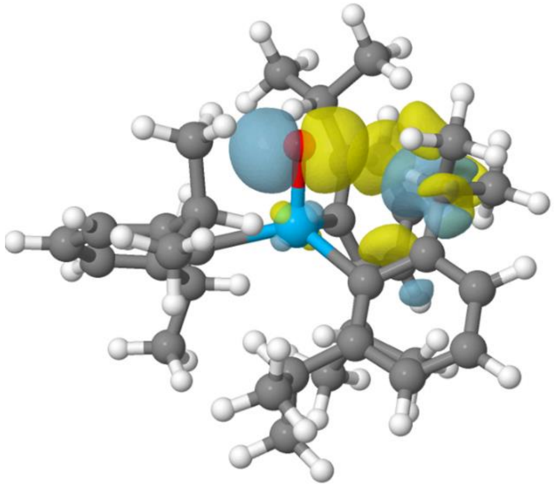

LP (2) O 2 -> BD*(1) C 9- H 41
1.46 kcal/mol

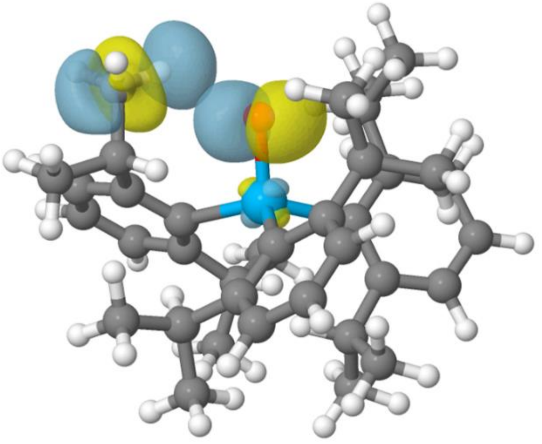

LP (2) O 2 -> BD*(1) C 23- H 61
0.68 kcal/mol

Figure 93. Pre-orthogonalized NLMO plots for **2a** (DKH-PBE0/old-DKH-TZVPP//PBE0/def2-TZVPP) of select O-centered lone pairs and C–H antibonding orbitals involved in donor-acceptor interactions (isosurface = 0.05). The energy of stabilization afforded by the interaction is displayed. An NBO deletion calculation for the strength the interaction between O2 and the C33–H73 unit affords an energy of 7.427 kcal/mol.

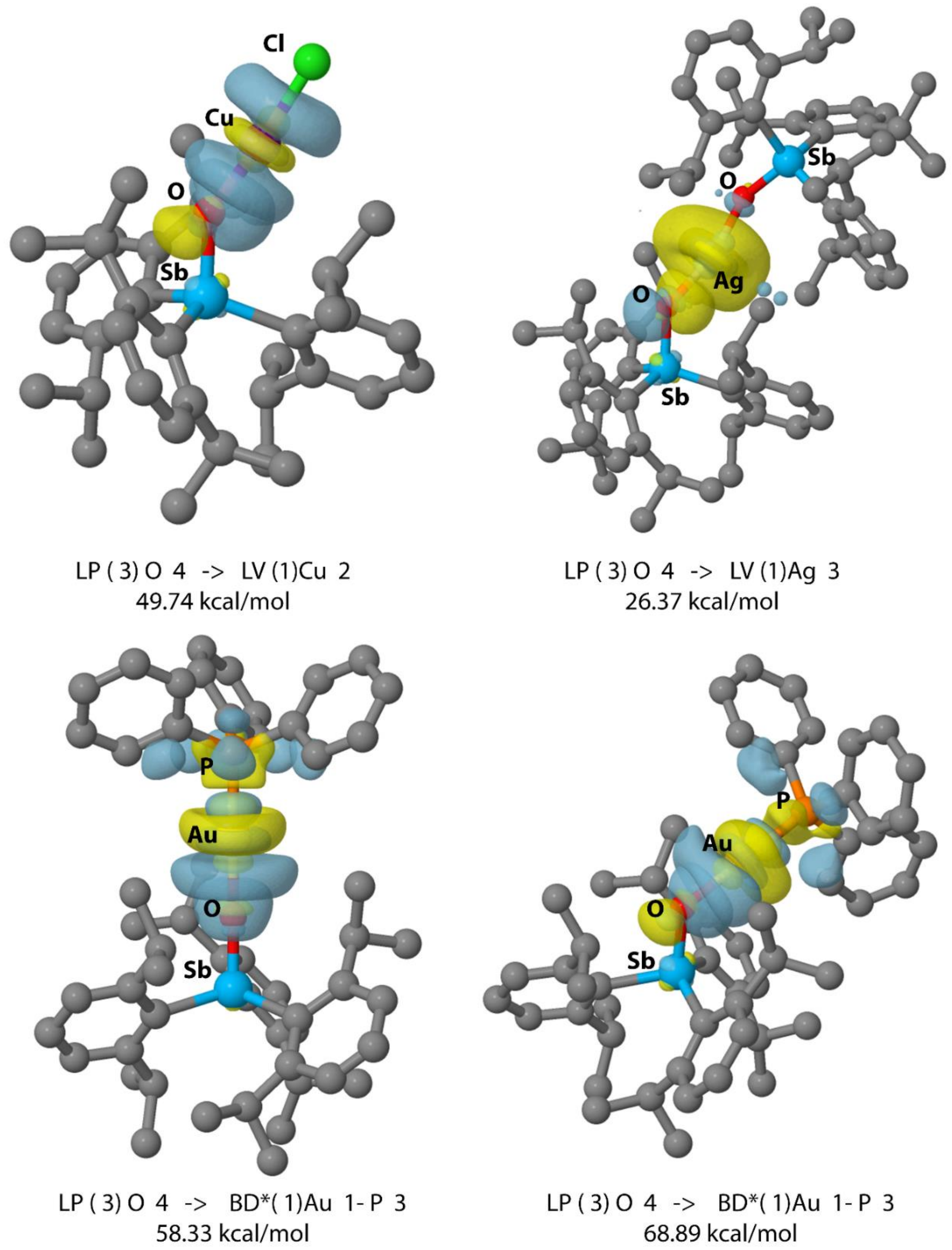

Figure 94. Pre-orthogonalized NBO plots for **4** (*top left*), **5** (*top right*), **6**-linear (*bottom left*), and **6**-bent (*bottom right*) (DKH-PBE0/old-DKH-TZVPP//PBE0/def2-TZVPP) of the O-centered lone pairs and metal-centered orbitals involved in donor-acceptor interactions (isosurface = 0.05). The energy of stabilization afforded by the interaction is displayed.

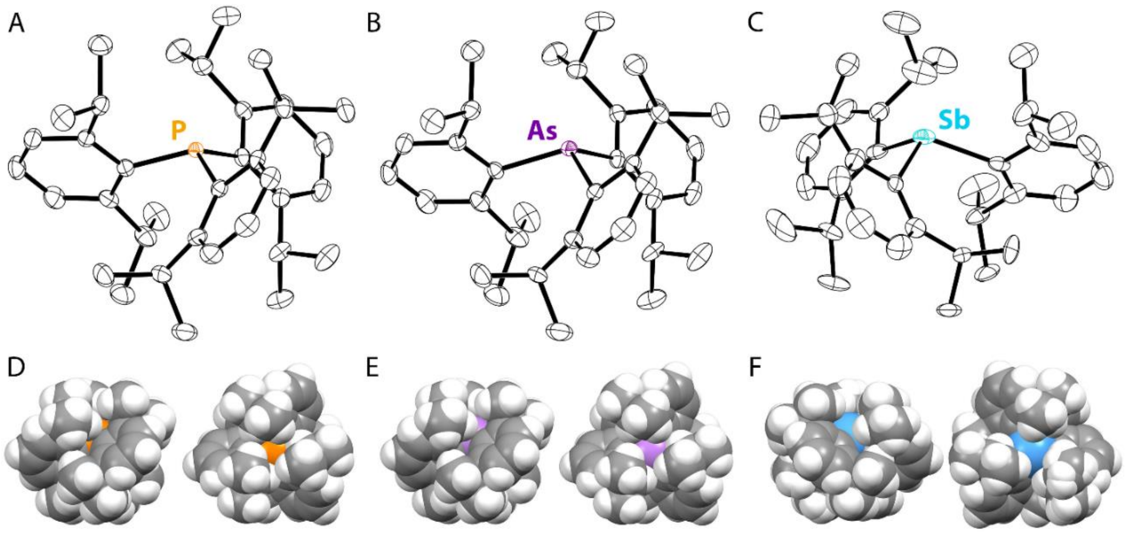

Figure 95. Thermal ellipsoid plots (50% probability level) of (A) **1c**, (B) **1b**, and (C) **1a**. H atoms are omitted for clarity. Space-fill diagrams of (D) **1c**, (E) **1b**, and (F) **1a**. Color code: P orange, As purple, Sb teal, C grey, H white.

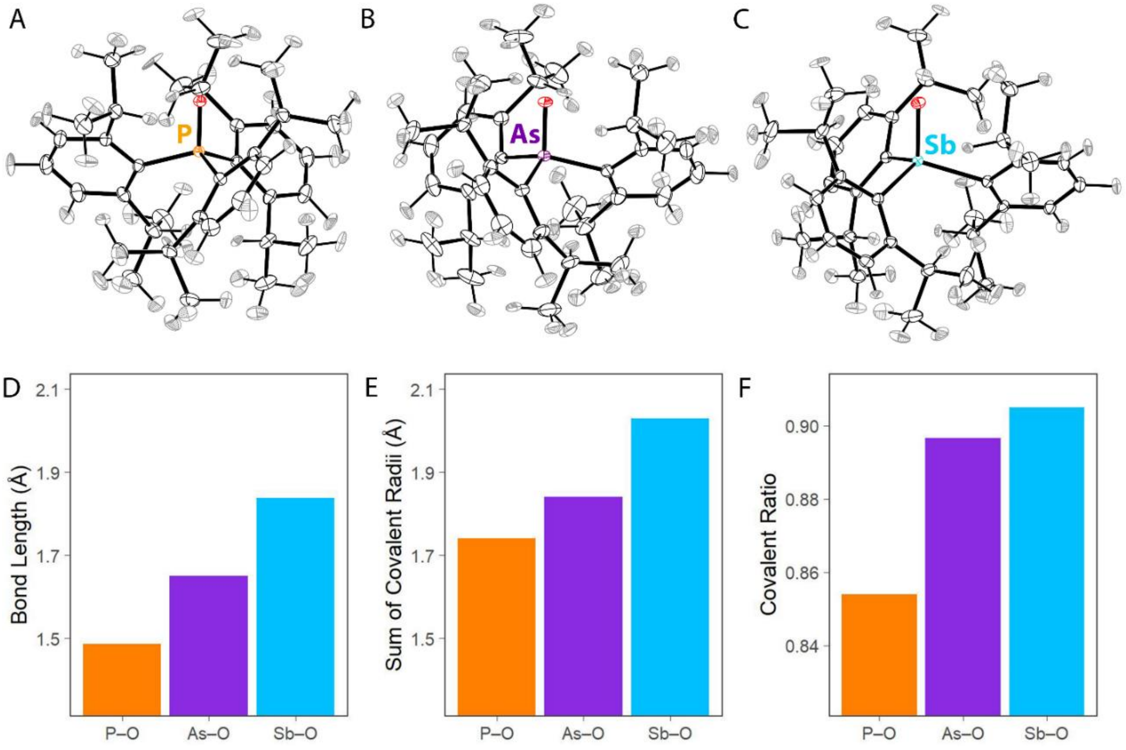

Figure 96. Thermal ellipsoid plots (50% probability level) of (A) **2c**, (B) **2b**, and (C) **2a** (monoclinic). (D) Pn–O bond lengths, (E) sum of Pn and O covalent radii,[36] and (F) covalent ratios. Color code: P orange, As purple, Sb teal, C black, H grey.

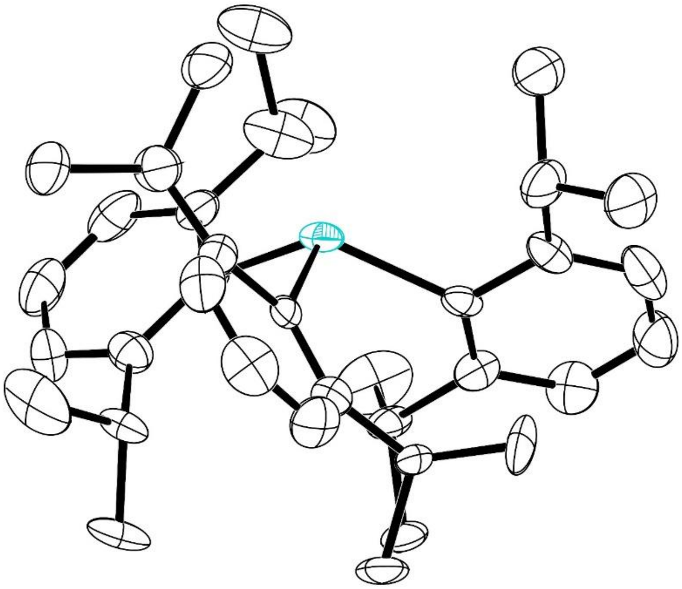

Figure 97. Thermal ellipsoid plot (50% probability) of **1a**. H atoms and disordered components are omitted for clarity. Color code: Sb teal, C black. CCDC 2133036.

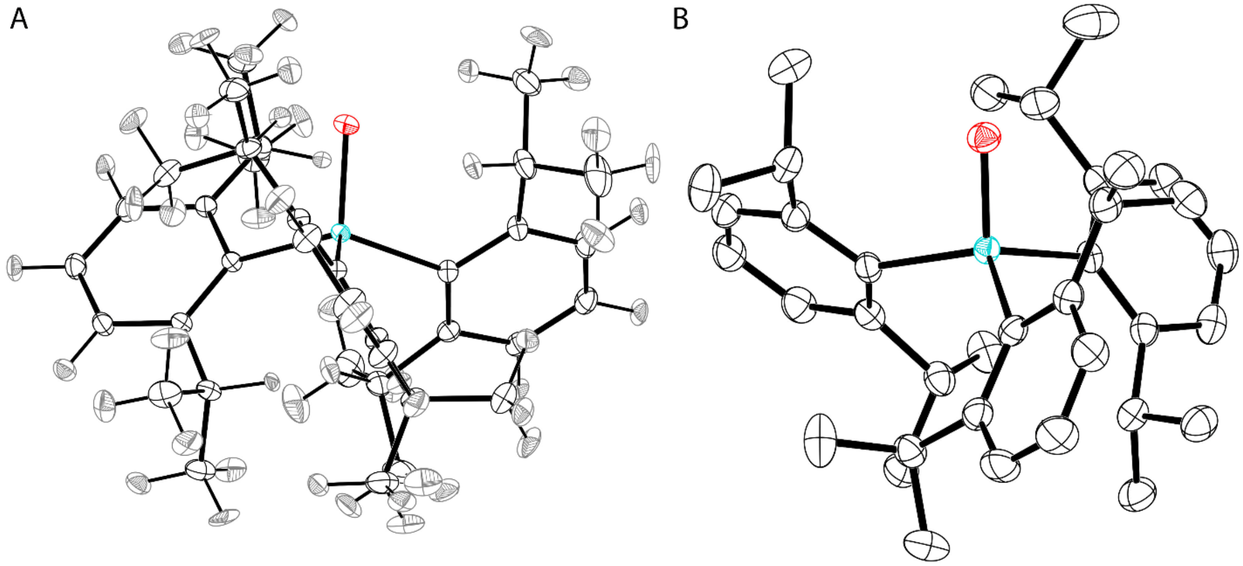

Figure 98. Thermal ellipsoid plot (50% probability) of (A) **2a** (monoclinic) and (B) **2a** (orthorhombic). **2a** (monoclinic) was refined using the HAR method, and **2a** (orthorhombic) was refined using the IAM method. In panel B, H atoms are omitted for clarity. Color code: Sb teal, O red, C black, H grey. The Sb–O bond length in **2a** (monoclinic) is 1.8372(5) Å and the Sb–O bond length in **2a** (orthorhombic) is 1.8428(14) Å. CCDC 2133037 (A) and 2182474 (B).

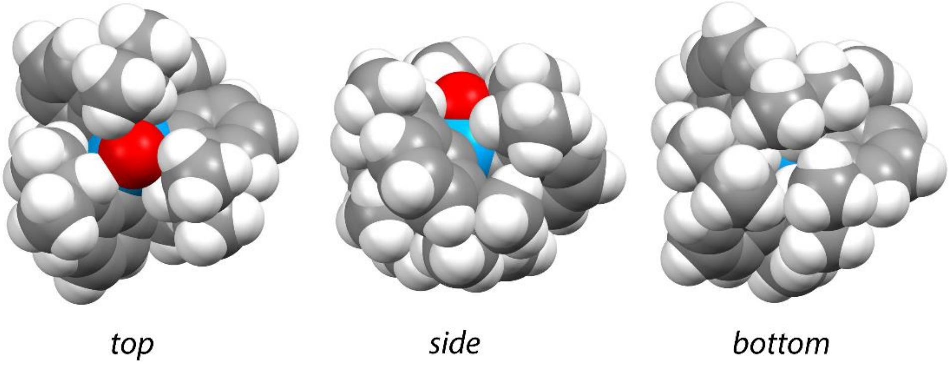

Figure 99. Space-filling diagrams of **2a** from views rotated successively by 90° about the horizontal axis. Color code: Sb teal, O red, C grey, H white.

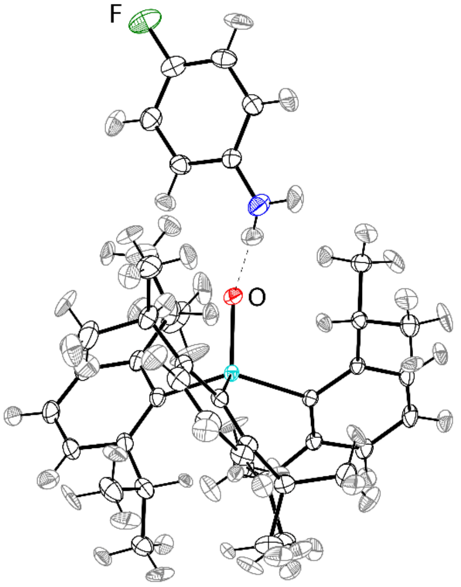

Figure 100. Thermal ellipsoid plot (50% probability) of **3**. Color code: Sb teal, O red, C black, H grey, F green, N blue. The Sb–O bond length in **3** is 1.8421(7) Å. CCDC 2133038.

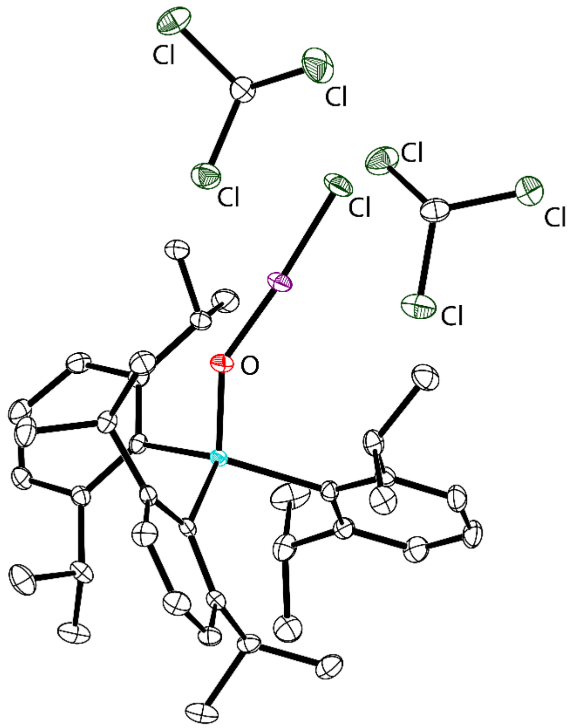

Figure 101. Thermal ellipsoid plot (50% probability) of **4**·$(CHCl_3)_2$. H atoms are omitted for clarity. Color code: Sb teal, O red, C black, Cl dark green, Cu mauve. The Sb–O bond length in **4**·$(CHCl_3)_2$ is 1.8591(14) Å. CCDC 2182479.

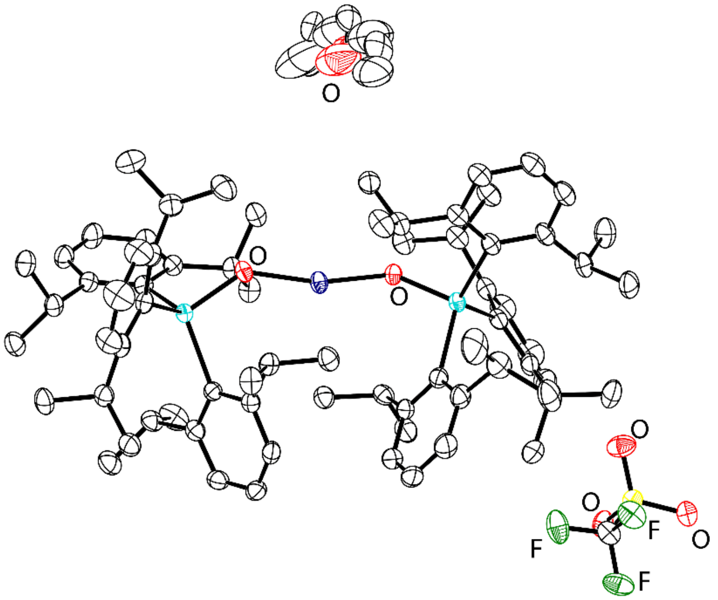

Figure 102. Thermal ellipsoid plot (50% probability) of **5**·OEt_2. H atoms and disordered components are omitted for clarity. Color code: Sb teal, O red, C black, F green, S yellow, Ag blue. The Sb–O bond lengths in **5**·OEt_2 are 1.8716(17) and 1.8670(17) Å. CCDC 2133039.

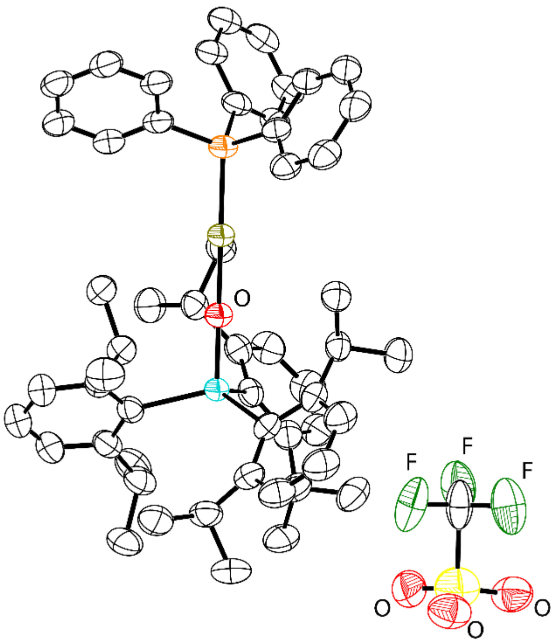

Figure 103. Thermal ellipsoid plot (50% probability) of **6**-linear (rhombohedral polymorph). H atoms are omitted for clarity. Color code: Sb teal, O red, C black, H grey, P orange, Au gold, S yellow, F green. The Sb–O bond length in **6**-linear is 1.850(7) Å. The O–Au bond length in **6**-linear is 1.982(7) Å. CCDC 2182481.

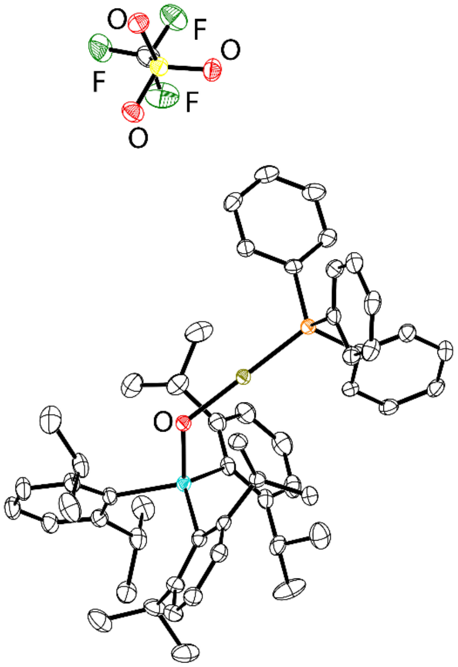

Figure 104. Thermal ellipsoid plot (50% probability) of **6**-bent (triclinic polymorph). H atoms are omitted for clarity. Color code: Sb teal, O red, C black, H grey, P orange, Au gold, S yellow, F green. The Sb–O bond length in **6**-bent 1.8932(17) Å. The O–Au bond length in **6**-bent is 2.0582(16) Å. CCDC 2182480.

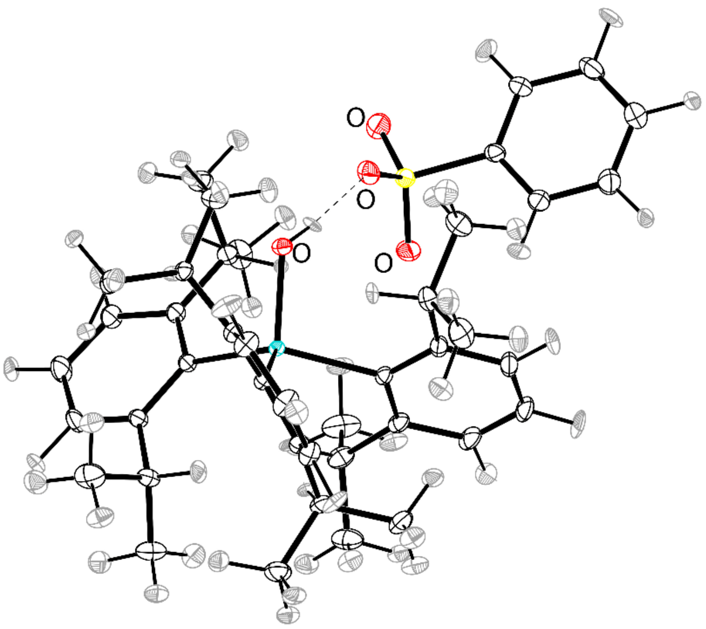

Figure 105. Thermal ellipsoid plot (50% probability) of **7a**. Color code: Sb teal, O red, C black, H grey, S yellow. The Sb–O bond length in **7a** is 1.9119(7) Å. CCDC 2133040.

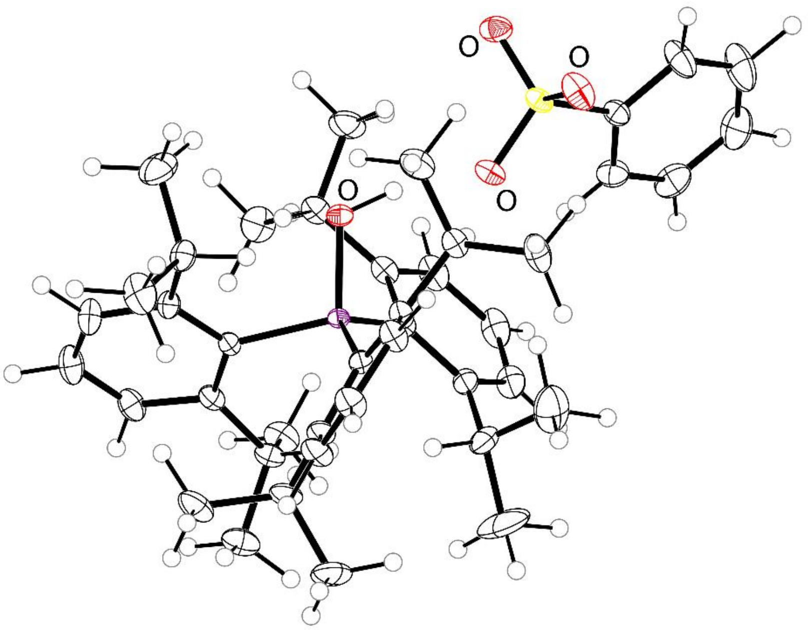

Figure 106. Thermal ellipsoid plot (50% probability) of **7b**. Color code: As purple, O red, C black, H grey, S yellow. The As–O bond length in **7b** is 1.7386(19) Å. CCDC 2182482.

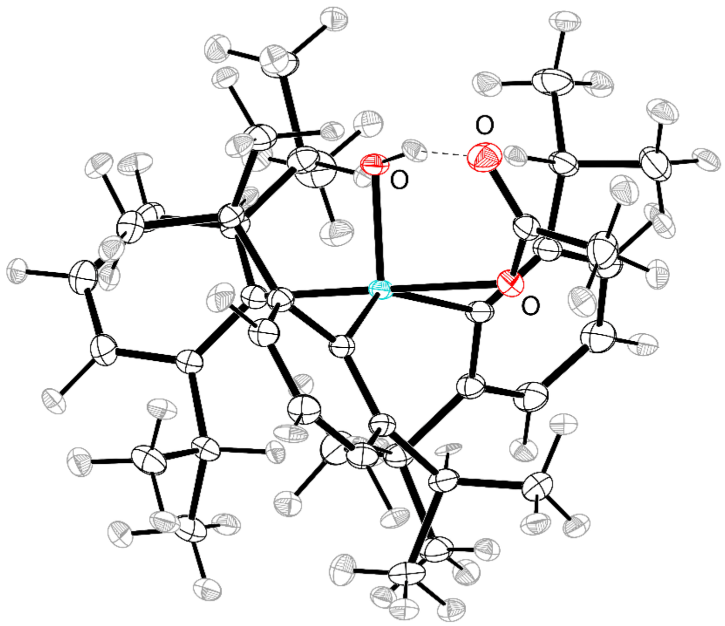

Figure 107. Thermal ellipsoid plot (50% probability) of **8**. Color code: Sb teal, O red, C black, H grey. The Sb–O bond lengths in **8** are 1.916(1) Å (equatorial hydroxo) and 2.320(1) Å (axial acetato). CCDC 2133041.

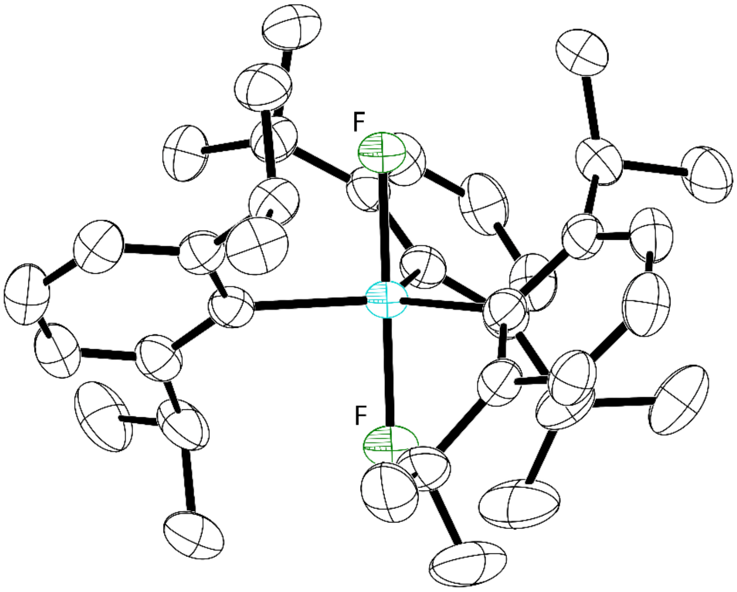

Figure 108. Thermal ellipsoid plot (50% probability) of **9**. H atoms omitted for clarity. Color code: Sb teal, C black, F green. CCDC 2133042.

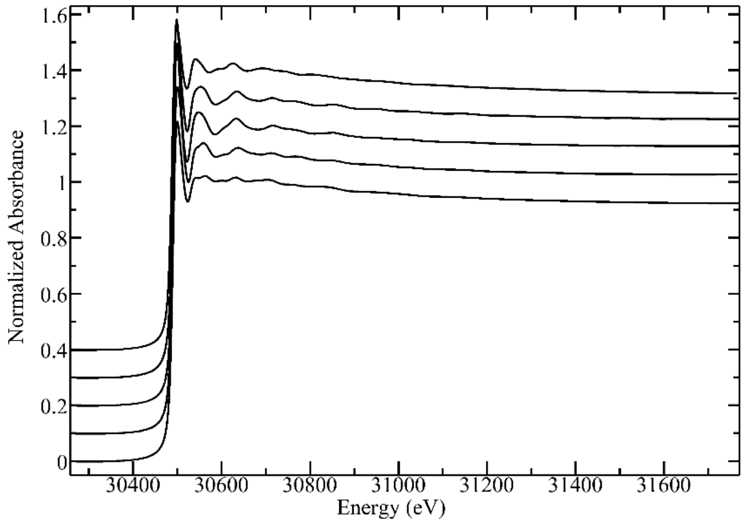

Figure 109. Full normalized Sb K-edge XAS spectra for (*top to bottom*) **1a**, **A**, **B**, **C**, **2a**.

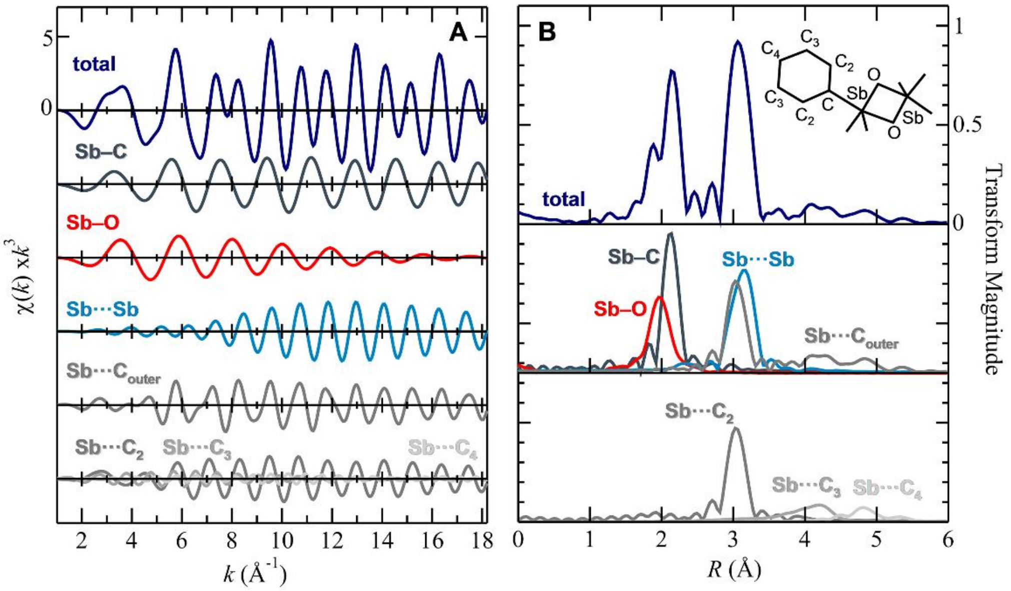

Figure 110. Breakdown of EXAFS curve-fitting components (A) and corresponding Sb–C phase-corrected Fourier transforms (B) for compound **A**, showing major components and summed outer-shell C components; the inset in B shows the core used to model the spectra. The overlap of the Sb···Sb interaction and the Sb···C_2 gives rise to the intense Fourier transform peak at 3.1 Å.

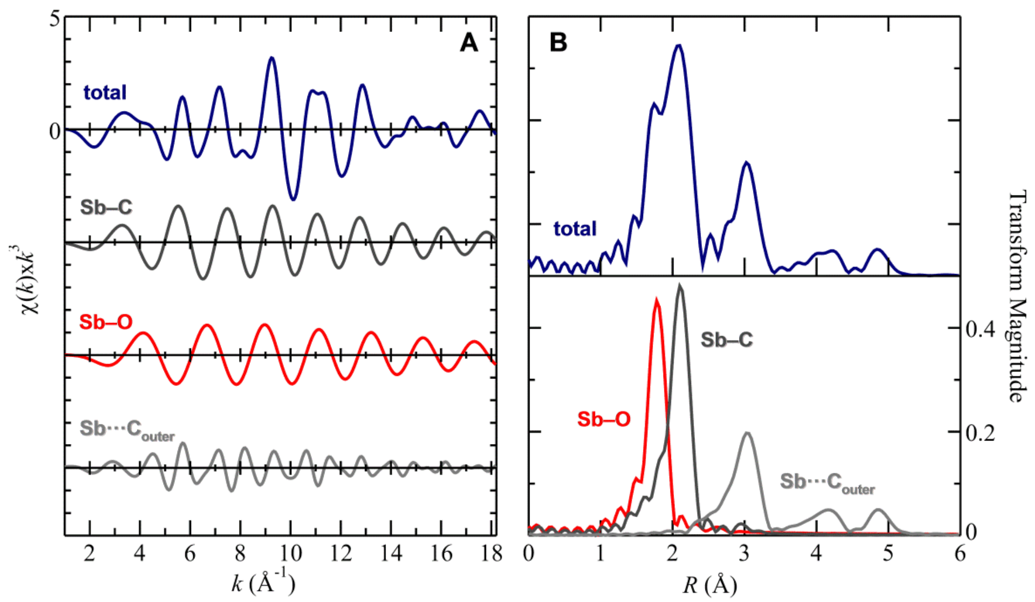

Figure 111. Breakdown of EXAFS curve-fitting components (A) and corresponding Sb–C phase-corrected Fourier transforms (B) for compound **2a**, showing major components and summed outer-shell C components.

Table 1. Crystallographic details for **1a**-**c**, **2a**.

Compound	**1a**	**1b**	**1c**	**2a**(orthorhombic)
Empirical formula	$C_{36}H_{51}Sb$	$C_{36}H_{51}As$	$c_{36}H_{51}P$	$C_{36}H_{51}OSb$
Formula weight	605.51	558.68	514.73	621.51
Temperature (K)	100.0(1)	100.0(1)	100.0(1)	100.0(1)
Wavelength (Å)	1.54184	1.54184	1.54184	1.54184
Crystal system	Cubic	Trigonal	Trigonal	Orthorhombic
Space group	$I\bar{4}3d$	*R*3	*R*3	*Pbca*
a (Å)	23.57660(10)	16.5325(3)	16.4276(3)	17.4672(2)
b (Å)				17.2872(1)
c (Å)		10.1989(2)	10.2126(2)	21.0673(2)
Volume (Å^3)	13105.20(17)	2414.13(10)	2386.79(10)	21.0673(2)
Z	16	3	3	8
ρ_{calc} (Mg/m^3)	1.228	1.153	1.074	1.298
Crystal size (mm^3)	0.1×0.09×0.07	0.09×0.06×0.05	0.1×0.07×0.05	0.15×0.11×0.07
θ range (°)	4.594 to 66.958	5.325 to 67.071	5.332 to 67.070	4.165 to 77.356
Total reflections	43467	9258	5257	43495
Unique reflections	1955	1908	1267	6653
Method	IAM	IAM	IAM	IAM
Parameters	176	117	117	355
Completeness	100	100.0	99.7	100.0
R_{int}	0.0499	0.0438	0.0265	0.0368
R_1 ($I > 2\sigma$)	0.0403	0.0301	0.0277	0.0250
R_1 (all data)	0.0404	0.0302	0.0281	0.0287
wR_2 ($I > 2\sigma$)	0.1038	0.0768	0.0725	0.0620
wR_2 (all data)	0.1039	0.0768	0.0727	0.0640
Goodness of fit, *S*	1.159	1.055	1.115	1.082

Table 2. Crystallographic details for **2a-b**.

Compound	**2a** (monoclinic)		**2b**	
Empirical formula	$C_{36}H_{51}OSb$		$C_{36}H_{51}AsO$	
Formula weight	621.51		574.68	
Temperature (K)	100.0(1)		100.0(1)	
Wavelength (Å)	0.71073		1.54184	
Crystal system	Monoclinic		Trigonal	
Space group	$P2_1/c$		$R3$	
a (Å)	9.8768(1)		16.7925(3)	
b (Å)	16.3435(2)			
c (Å)	20.0481(3)		9.8907(3)	
β (°)	101.240(1)			
Volume (Å^3)	3174.12(7)		2415.40(11)	
Z	4		3	
ρ_{calc} (Mg/m^3)	1.301		1.185	
Crystal size (mm^3)	0.59×0.39×0.23		0.13×0.07×0.05	
θ range (°)	2.417 to 30.033		5.268 to 66.963	
Total reflections	93669		7302	
Unique reflections	9259		1848	
Method	IAM	HAR	IAM	HAR
Parameters	355	802	120	269
Completeness	99.9	99.9	100.0	100.0
R_{int}	0.0385	0.0385	0.0402	0.0402
R_1 ($I > 2\sigma$)	0.0185	0.0124	0.0251	0.0233
R_1 (all data)	0.0196	0.0135	0.0252	0.0233
wR_2 ($I > 2\sigma$)	0.0446	0.0244	0.0611	0.0534
wR_2 (all data)	0.0451	0.0246	0.0611	0.0534
Goodness of fit, *S*	1.054	1.0903	1.046	1.0810

Table 3. Crystallographic details for **2c**, **3**.

Compound	**2c**		**3**	
Empirical formula	$C_{36}H_{51}PO$		$C_{42}H_{57}FNOSb$	
Formula weight	530.73		732.63	
Temperature (K)	100.0(1)		100.0(1)	
Wavelength (Å)	1.54184		0.71073	
Crystal system	Trigonal		Monoclinic	
Space group	*R*3		*C*2/*c*	
a (Å)	16.7252(3)		21.5840(4)	
b (Å)			16.9212(2)	
c (Å)	9.8790(2)		22.4080(4)	
β (°)			109.562(2)	
Volume (Å^3)	2393.24(10)		7711.6(2)	
Z	3		8	
ρ_{calc} (Mg/m^3)	1.105		1.262	
Crystal size (mm^3)	0.1×0.06×0.04		0.37×0.10×0.05	
θ range (°)	5.289 to 66.828		2.209 to 30.508	
Total reflections	7135		81207	
Unique reflections	1894		11781	
Method	IAM	HAR	IAM	HAR
Parameters	120	269	435	928
Completeness	100.0	100.0	99.9	99.9
R_{int}	0.0253	0.0253	0.0439	0.0439
R_1 (I > 2σ)	0.0231	0.0133	0.0240	0.0189
R_1 (all data)	0.0233	0.0136	0.0293	0.0240
wR_2 (I > 2σ)	0.0591	0.0290	0.0599	0.0407
wR_2 (all data)	0.0592	0.0291	0.0620	0.0422
Goodness of fit, *S*	1.067	1.0849	1.047	1.0253

Table 4. Crystallographic details for **4**-**6**.

Compound	**4**·$(CHCl_3)_2$	**5**·OEt_2	**6**-bent	**6**-linear
Empirical formula	$C_{38}H_{53}Cl_7CuOSb$	$C_{77}H_{112}AgF_3O_6SSb_2$	$C_{55}H_{66}AuF_3O_4PSSb$	$C_{55}H_{66}AuF_3O_4PSSb$
Formula weight	959.24	1574.09	1229.82	1229.82
Temperature (K)	100.0(1)	100.0(1)	100.0(1)	100.0(1)
Wavelength (Å)	1.54184	1.54184	1.54184	1.54184
Crystal system	Triclinic	Triclinic	Triclinic	Trigonal
Space group	$P\overline{1}$	$P\overline{1}$	$P\overline{1}$	$R\overline{3}$
a (Å)	10.4183(3)	13.9579(2)	11.91490(1)	14.5599(2)
b (Å)	12.3198(2)	16.4020(2)	14.9286(2)	
c (Å)	18.2971(4)	17.5587(2)	15.2073(2)	44.5120(8)
α (°)	75.022(2)	104.8710(10)	99.7460(1)	
β (°)	79.759(2)	100.3270(10)	90.0020(10)	
γ (°)	67.747(2)	93.9180(10)	96.7000(1)	
Volume (Å^3)	2091.30(9)	3794.53(9)	2647.18(6)	8171.9(3)
Z	2	2	2	6
ρ_{calc} (Mg/m^3)	1.523	1.378	1.543	1.499
Crystal size (mm^3)	0.09×0.07×0.05	0.22×0.09×0.07	0.17×0.06×0.05	0.22×0.17×0.09
θ range (°)	2.510 to 67.074	2.660 to 70.074	2.949 to 67.076	2.978 to 67.075
Total reflections	27551	93501	79065	23949
Unique reflections	7464	14385	9456	3258
Method	IAM	IAM	IAM	IAM
Parameters	445	881	607	227
Completeness	99.9	99.9	100.0	99.9
R_{int}	0.0422	0.0623	0.0468	0.0609
R_1 ($I > 2\sigma$)	0.0236	0.0324	0.0206	0.0466
R_1 (all data)	0.0253	0.0351	0.0218	0.0586
wR_2 ($I > 2\sigma$)	0.0562	0.0841	0.0504	0.1167
wR_2 (all data)	0.0570	0.0863	0.0511	0.1330
Goodness of fit, *S*	1.054	1.030	1.033	1.076

Table 5. Crystallographic details for **7a**-**b**.

Compound	**7a**		**7b**	
Empirical formula	$C_{42}H_{57}O_4SSb$		$C_{42}H_{57}AsO_4S$	
Formula weight	779.68		732.85	
Temperature (K)	100.0(1)		100.0(1)	
Wavelength (Å)	0.71073		0.71073	
Crystal system	Monoclinic		Orthorhombic	
Space group	$P2_1/n$		$Pna2_1$	
a (Å)	10.5230(2)		20.4704(6)	
b (Å)	17.9547(4)		11.7243(3)	
c (Å)	20.0882(4)		16.4581(5)	
α (°)				
β (°)	92.246(2)			
γ (°)				
Volume ($Å^3$)	3792.49(13)		3949.96	
Z	4		4	
ρ_{calc} (Mg/m^3)	1.366		1.232	
Crystal size (mm^3)	0.24×.2×0.13		0.44×0.24×0.1	
θ range (°)	2.43 to 29.57		2.343 to 29.57	
Total reflections	49940		51691	
Unique reflections	10627		12957	
Method	IAM	HAR	IAM	HAR
Parameters	449	946	450	606
Completeness	99.9	99.9	99.9	99.9
R_{int}	0.0462	0.0462	0.0504	0.0527
R_1 ($I > 2\sigma$)	0.0264	0.0217	0.0517	0.0456
R_1 (all data)	0.0320	0.0272	0.0665	0.0520
wR_2 ($I > 2\sigma$)	0.0588	0.0414	0.1218	0.1117
wR_2 (all data)	0.0611	0.0432	0.13	0.1170
Goodness of fit, *S*	1.060	1.0263	1.031	1.0054

Table 6. Crystallographic details for **8**-**9**.

Compound	**8**		**9**
Empirical formula	$C_{38}H_{55}O_3Sb$		$C_{36}H_{51}F_2Sb$
Formula weight	681.57		643.51
Temperature (K)	100.0(1)		100.0(1)
Wavelength (Å)	0.71073		1.54184
Crystal system	Monoclinic		Cubic
Space group	$P2_1/c$		$I\bar{4}3d$
a (Å)	10.1367(3)		23.7603(2)
b (Å)	18.3322(4)		
c (Å)	19.0827(5)		
β (°)	102.117(3)		
Volume ($Å^3$)	3467.09(15)		13413.9(3)
Z	4		16
ρ_{calc} (Mg/m^3)	1.306		1.275
Crystal size (mm^3)	0.171×0.087×0.074		0.21×0.14×0.13
θ range (°)	2.183 to 33.142		4.558 to 66.925
Total reflections	51848		16123
Unique reflections	13207		1998
Method	IAM	HAR	IAM
Parameters	396	874	123
Completeness	99.9	99.9	100.0
R_{int}	0.0529	0.0529	0.0239
R_1 ($I > 2\sigma$)	0.0355	0.0324	0.0222
R_1 (all data)	0.0578	0.0547	0.0229
wR_2 ($I > 2\sigma$)	0.0747	0.0609	0.0561
wR_2 (all data)	0.0820	0.0675	0.0565
Goodness of fit, *S*	1.024	1.0457	1.121

Table 7. EXAFS parameters for **1a**, **A**, **B**, **C**, and **2a**.[a]

**Sample**	**Backscatterer**[b]	***N***	***R* (Å)**	**σ^2 ($Å^2$)**	**ΔE_0 (eV)**	***F*** [c]
**1a**	C	3	2.200(2)	0.0026(1)	−13.3(6)	0.316
**A**	O	1	1.967(4)	0.0025(2)	−15.8(4)	0.221
	O	1	2.049(5)	0.0028(2)		
	C	3	2.150(1)	0.0022(1)		
	Sb	1	3.148(3)	0.0025(3)		
**B**	O	2	2.128(3)	0.0019(2)	−13.5(5)	0.222
	C	3	2.077(4)	0.0052(3)		
**C**	O	1	1.905(1)	0.0019(1)	−16.5(4)	0.249
	C	3	2.136(1)	0.0024(1)		
**2a**	O	1	1.837(2)	0.0021(1)	−15.7(6)	0.319
	C	3	2.165(2)	0.0031(1)		

[a] *N*, coordination number; *R*, interatomic distance; σ^2, mean-square deviation in *R* (Debye-Waller factor); ΔE_0, threshold energy shift. Values in parentheses are estimated standard deviations. [b] Only the first-shell or Sb-Sb contributions (in the case of **A**) are shown in the table. Outer C shells were as described in the experimental section. [c] $F = [\Sigma k^6(\chi_{calc}(k) - \chi_{exp}(k))^2 / \Sigma k^6(\chi_{exp}(k))^2]^{0.5}$ in which $\chi_{exp}(k)$ and $\chi_{calc}(k)$ are the experimental and calculated EXAFS, respectively, and the summations are over all points included within the fitted *k*-range, which in all cases was 1-18.1 $Å^{-1}$.

Table 8. Energy Decomposition Analysis (PBE0/QZVP) of **2a**.[h]

	E_{SCF}(first, 2a)[a]	E_{SCF}(last, 2a)[b]	E_{SCF}($Dipp_3Sb$)[c]	E_{SCF}(O)[d]	ΔE_{tot}[e]	ΔE_{orb}[f]	ΔE_{steric}[g]
Energy (a.u)	-3550.882	-3552.812	-3476.568	-74.910	-1.333	-1.930	0.597
Energy (kJ/mol)	-9322688	-9327755	-9127580	-196674	-3501	-5068	1567

[a] Energy of **2a** with no orbital interaction between O atom and $Dipp_3Sb$ fragments. [b] Energy of **2a**. [c] Energy of $Dipp_3Sb$ at optimized coordinates for **2a**. [d] Energy of O atom. [e] Difference in energy of **2a** and the sum of the O and $Dipp_3Sb$ fragments; the total energy afforded by interaction of the fragments. [f] Difference in energy between **E_{SCF}(last, 2a)** and **E_{SCF}(first, 2a)**; the energy of stabilization afforded by orbital interaction between the $Dipp_3Sb$ and O atom fragments. [g] Difference between ΔE_{tot} and ΔE_{orb}. [h] $\Delta E_{orb}/\Delta E_{tot}$ = 1.45.

Table 9. Energy Decomposition Analysis (PBE0/TZVP) of **2b**.[h]

	E_{SCF}(first, 2b)[a]	E_{SCF}(last, 2b)[b]	E_{SCF}($Dipp_3As$)[c]	E_{SCF}(O)[d]	ΔE_{tot}[e]	ΔE_{orb}[f]	ΔE_{steric}[g]
Energy (a.u)	-3711.344	-3712.025	-3636.863	-74.908	-0.255	-0.681	0.427
Energy (kJ/mol)	-9743974	-9745763	-9548428	-196666	-669	-1789	1120

[a] Energy of **2b** with no orbital interaction between O atom and $Dipp_3As$ fragments. [b] Energy of **2b**. [c] Energy of $Dipp_3As$ at optimized coordinates for **2b**. [d] Energy of O atom. [e] Difference in energy of **2b** and the sum of the O and $Dipp_3As$ fragments; the total energy afforded by interaction of the fragments. [f] Difference in energy between **E_{SCF}(last, 2b)** and **E_{SCF}(first, 2b)**; the energy of stabilization afforded by orbital interaction between the $Dipp_3As$ and O atom fragments. [g] Difference between ΔE_{tot} and ΔE_{orb}. [h] $\Delta E_{orb}/\Delta E_{tot}$ = 2.67.

Table 10. Energy Decomposition Analysis (PBE0/TZVP) of **2c**.[h]

	E_{SCF}(first, 2c)[a]	E_{SCF}(last, 2c)[b]	E_{SCF}($Dipp_3P$)[c]	E_{SCF}(O)[d]	ΔE_{tot}[e]	ΔE_{orb}[f]	ΔE_{steric}[g]
Energy (a.u)	-1816.690	-1817.690	-1742.494	-74.908	-0.289	-1.000	0.711
Energy (kJ/mol)	-4769642	-4772268	-4574843	-196667	-759	-2626	1867

[a] Energy of **2c** with no orbital interaction between O atom and $Dipp_3P$ fragments. [b] Energy of **2c**. [c] Energy of $Dipp_3P$ at optimized coordinates for **2c**. [d] Energy of O atom. [e] Difference in energy of **2c** and the sum of the O and $Dipp_3P$ fragments; the total energy afforded by interaction of the fragments. [f] Difference in energy between **E_{SCF}(last, 2c)** and **E_{SCF}(first, 2c)**; the energy of stabilization afforded by orbital interaction between the $Dipp_3P$ and O atom fragments. [g] Difference between ΔE_{tot} and ΔE_{orb}. [h] $\Delta E_{orb}/\Delta E_{tot}$ = 3.46.

Table 11. Values of ρ ($e^- Å^{-3}$), $\nabla^2\rho$ ($e^- Å^{-5}$), ε, and normalized distance at the Pn–O and O–A bond critical points for compounds **2a**-**8**, **A**, **B**.

Compound	Bond	Density	Laplacian	Ellipticity	Distance
**2c**	P–O	0.231744	1.386415	0.005527	0.403333
**2b**	As–O	0.215627	0.718006	0.003327	0.479333
**2a**	Sb–O	0.173485	0.649753	0.007443	0.519
**3**	Sb–O	0.167596	0.618702	0.002694	0.518
**3**	O–H	0.033441	0.094247	0.014997	0.651333
**4**	Sb–O	0.159136	0.613314	0.017536	0.516
**4**	O–Cu	0.111688	0.617602	0.001759	0.506667
**5**	Sb–O	0.138281	0.444832	0.026744	0.513667
**5**	Sb–O	0.138001	0.441971	0.027642	0.513667
**5**	O–Ag	0.082517	0.404382	0.006623	0.472667
**5**	O–Ag	0.082408	0.403461	0.00692	0.472667
**6-linear**	Sb–O	0.151297	0.624267	1.96E-05	0.515
**6-linear**	O–Au	0.129511	0.581037	3.71E-05	0.463
**6-bent**	Sb–O	0.149506	0.536553	0.041721	0.513667
**6-bent**	O–Au	0.108848	0.417859	0.011227	0.469
**7b**	O–H	0.499385	-4.89632	0.008756	0.846666
**7b**	As–O	0.174469	0.473733	0.071716	0.471
**7a**	Sb–O	0.144227	0.508145	0.075903	0.511333
**7a**	O–H	0.346279	-2.67294	0.005965	0.832334
**8**	Sb–O	0.14362	0.497205	0.086585	0.510667
**8**	O–H	0.318796	-2.30282	0.006293	0.823333
**A**	Sb–O	0.136971	0.48016	0.088189	0.510667
**A**	Sb–O	0.136974	0.480125	0.088186	0.510667
**A**	O–Sb	0.100908	0.300082	0.043538	0.495667
**A**	O–Sb	0.100914	0.300104	0.043552	0.495667
**B**	Sb–O	0.10889	0.344495	0.080336	0.506667
**B**	Sb–O	0.110226	0.354613	0.082158	0.506667
**B**	O–H	0.374205	-2.72717	0.007813	0.806333
**B**	O–H	0.374768	-2.72953	0.007735	0.806001

Table 12. Values of ρ ($e^- Å^{-3}$), $\nabla^2\rho$ ($e^- Å^{-5}$), ε, and normalized distance at the O···H bond critical points for compounds **2a**-**c**. Benzylic H atoms are denoted "H_b" and terminal H atoms are denoted "H_t."

Compound	Bond	Density	Laplacian	Ellipticity	Distance
**2a**	O–H_b	0.018671	0.061694	0.63093	0.613
**2a**	O–H_t	0.012762	0.047758	0.161681	0.599667
**2a**	O–H_b	0.023615	0.081046	0.057021	0.622333
**2b**	O–H_b	0.01884	0.06796	0.164366	0.604333
**2b**	O–H_t	0.011732	0.044857	0.312139	0.588
**2b**	O–H_b	0.01601	0.057627	0.180082	0.597333
**2b**	O–H_t	0.00933	0.035151	0.352238	0.584
**2b**	O–H_b	0.017945	0.065737	0.048447	0.599667
**2b**	O–H_t	0.010691	0.040523	0.258594	0.589333
**2c**	O–H_b	0.018748	0.069759	0.083155	0.6
**2c**	O–H_t	0.012736	0.050405	0.299645	0.588666
**2c**	O–H_b	0.016223	0.060218	0.091032	0.593333
**2c**	O–H_t	0.009108	0.035358	0.402915	0.581
**2c**	O–H_b	0.017205	0.064809	0.071469	0.592667
**2c**	O–H_t	0.010698	0.041732	0.286604	0.586667

Table 13. Values of ρ ($e^- Å^{-3}$), $\nabla^2\rho$ ($e^- Å^{-5}$), ε, and normalized distance at the O···H bond critical points for compounds **2a**, **3**, **4**, **5**, **6**-bent, and **6**-linear.

Compound	Bond	Density	Laplacian	Ellipticity	Distance
**2a**	O–H_b	0.018671	0.061694	0.63093	0.613
**2a**	O–H_t	0.012762	0.047758	0.161681	0.599667
**2a**	O–H_b	0.023615	0.081046	0.057021	0.622333
**3**	O–H	0.007079	0.02545	0.510233	0.579
**3**	O–H	0.016597	0.056964	0.187168	0.606
**3**	O–H	0.011361	0.040819	0.128828	0.598333
**3**	O–H	0.012608	0.041409	0.450714	0.595
**3**	O–H	0.022207	0.07324	0.150897	0.622667
**4**	O–H	0.010153	0.038057	0.164826	0.590333
**4**	O–H	0.011889	0.040616	0.635793	0.590667
**4**	O–H	0.010164	0.038137	0.441307	0.588667
**4**	O–H	0.016469	0.055848	0.582978	0.612333
**5**	O–H	0.016853	0.059598	0.201279	0.604333
**5**	O–H	0.01618	0.054692	0.317991	0.610667
**5**	O–H	0.00648	0.022634	0.296603	0.579667
**5**	O–H	0.013719	0.044987	0.882015	0.599667
**6-linear**	O–H	0.015856	0.05566	0.259779	0.602333
**6-linear**	O–H	0.015854	0.055679	0.259967	0.602333
**6-linear**	O–H	0.015858	0.055662	0.25959	0.602333
**6-bent**	O–H	0.019006	0.066428	0.139138	0.615
**6-bent**	O–H	0.007431	0.027378	0.310596	0.580667
**6-bent**	O–H	0.022128	0.07885	0.163286	0.614334

Table 14. Select donor–acceptor interactions found from 2nd order perturbation theory analysis (DKH-PBE0/old-DKH-TZVPP//PBE0/def2-TZVPP).

Donor NBO	Acceptor NBO	Energy of stabilization (kcal/mol)
62. LP (2) O 2	165. BD*(1)Sb 1- C 3	4.04
62. LP (2) O 2	166. BD*(1)Sb 1- C 15	11.30
62. LP (2) O 2	167. BD*(1)Sb 1- C 27	2.61
63. LP (3) O 2	165. BD*(1)Sb 1- C 3	8.46
63. LP (3) O 2	167. BD*(1)Sb 1- C 27	9.45
63. LP (3) O 2	248. BD*(1) C 33- H 73	3.38
62. LP (2) O 2	184. BD*(1) C 9- H 41	1.46
62. LP (2) O 2	220. BD*(1) C 23- H 61	0.68

Table 15. Select results from NBO analysis of compounds **2a**-**8**, **A**, **B**.

Compound	NPA Pn[a]	NPA O[b]	NPA A[c]	WBI PnO[d]	WBI OA[e]	NLMO %Pn[f]	NLMO %O[g]	NLMO Pn%s[h]	NLMO Pn%p[i]	NLMO O%s[j]	NLMO O%p[k]	E2 PnC[l]	E2 CH[m,q]	E2 OA[n]
**2c**	1.913	-1.126	NA	1.09	NA	25	74	32	67	38	62	62	0	NA
**2b**	1.844	-1.144	NA	1.03	NA	28	71	34	65	25	75	46	0.50	NA
**2a**	2.157	-1.241	NA	0.94	NA	25	74	39	61	21	79	36	5.52	NA
**3**	2.218	-1.278	0.441	0.88	0.04	24	75	37	62	21	78	31	4.65	13
**4**	2.235	-1.331	0.675	0.75	0.18	20	79	33	66	26	74	22	1.06	63
**5**[o]	1.975	-1.139	0.766	0.85	0.21	24	74	36	64	18	82	21	3.05	39
**6-linear**	2.252	-1.350	0.466	0.65	0.25	17	81	31	68	21	79	21	1.23	58
**6-bent**	2.228	-1.276	0.437	0.70	0.26	20	79	32	68	24	76	18	4.16	79
**7b**	1.844	-0.944	0.504	0.76	0.71	24	75	25	74	25	75	17	0	NA
**7a**	2.208	-1.097	0.539	0.65	0.64	20	79	28	72	25	75	14	0	NA
**8**	2.269	-1.125	0.510	0.61	0.61	19	80	31	69	25	75	15	3.11	NA
**A**	2.405	-1.274	2.405	0.61	0.38	19	80	39	61	18	82	19	0	107[r]
**B**[p]	2.341	-1.133	0.467	0.42	0.77	11	86	31	68	26	74	33	2.32	NA

[a] Natural population of pnictogen. [b] Natural population of oxygen. [c] Natural population of A (coordinated Lewis acid atom). [d] Wiberg bond index of Pn–O bond. [e] Wiberg bond index of O–A bond. [f] Pn-atom contribution to the pnictoryl bonding NLMO. [g] O-atom contribution to the pnictoryl bonding NLMO. [h] %s character of the Pn-atom contribution to the pnictoryl bonding NLMO. [i] %p character of the Pn-atom contribution to the pnictoryl bonding NLMO. [j] %s character of the O-atom contribution to the pnictoryl bonding NLMO. [k] %p character of the O-atom contribution to the pnictoryl bonding NLMO. [l] Energy of stabilization afforded by delocalization of electron density from O-centered lone pairs to Pn–C σ* orbitals. [m] Energy of stabilization afforded by delocalization of electron density from O-centered lone pairs to C–H σ* orbitals. [n] Energy of stabilization afforded by delocalization of electron density from O-centered lone pairs to A orbitals (Rydburg orbitals not included). [o] NBO analysis of **5** was performed at BP86/def2-SVP level of theory. [p] No Pn–O bonding orbital is present; the primary O-centered lone pair is described. [q] No delocalizations below 0.50 kcal/mol printed. [r] Delocalizations from O-centered lone pairs to Sb–C and Sb–O σ* orbitals.

Table 16. Deletion energies of **2a**-**c**.

Compound	2c	2b	2a
**Deletion Energy (kcal/mol)**[a]	172.11	116.68	92.28

[a] Energy of destabilization upon deleting non-covalent interactions between the O atom and $Dipp_3Pn$ molecular fragments.

Table 17. Force constants of Pn–O bond stretch in **2a**-**c**.

Compound	2c	2b	2a
**Force constant (mdyne/Å)**[a]	9.014	6.852	5.656
**Force constant (mdyne/Å)**[b]	8.293	6.338	5.362

[a] Force constants obtained by fitting a quadratic function to a rigid surface scan of Pn–O stretching (DKH-PBE0/old-DKH-TZVPP). [b] Force constants obtained by diagonalization of the Hessian matrix with respect to potential energy (PBE0/def2-TZVPP).

Table 18. Selected bond lengths and angles for **4**·$(CHCl_3)_2$, **5**·OEt_2, **6**-linear, and **6**-bent.

	**4**·$(CHCl_3)_2$	**5**·OEt_2	**6**-linear	**6**-bent
**Sb–O–M angle (°)**	145.40(9)	130.42(10)/ 130.51(9)	180.0	122.15(8)
**O–M–X angle (°)**[a]	175.27(5)	166.78(7)	180.0	179.89(6)
**Sb–O distance (Å)**	1.8591(14)	1.8716(17)/ 1.8670(17)	1.850(7)	1.8932(17)
**O–M distance (Å)**	1.8286(14)	2.0939(17)/ 2.0926(17)	1.982(7)	2.0582(16)

[a] X denotes the coordinated atom of the second ligand; Cl in the case of **4**·$(CHCl_3)_2$, O in the case of **5**·OEt_2, P in the case of **6**-linear and **6**-bent.

Table 19. Cartesian coordinates (Å) of the optimized (PBE0/def2-TZVPP) structure of **A**.

Sb	3.76673	0.585327	0.057147
O	5.521398	1.066032	-0.59709
C	3.05376	2.071397	2.662345
H	3.155995	2.957399	2.04694
C	3.244194	0.808839	2.112468
C	3.694225	3.321699	-1.43477
H	4.706163	3.03876	-1.69701
C	1.861391	4.876663	-1.55232
H	1.442433	5.809839	-1.91021
C	2.551843	-2.08245	-0.87992
H	3.263726	-2.49102	-0.17476
C	2.494027	-0.70417	-1.07201
C	2.733864	2.205764	4.007194
H	2.597682	3.193376	4.431833
C	1.102021	4.036639	-0.74963
H	0.089251	4.311658	-0.47843
C	2.941879	2.47215	-0.62935
C	3.113484	-0.31931	2.917351
H	3.294461	-1.30186	2.500798
C	1.721315	-2.92047	-1.60963
H	1.770124	-3.99199	-1.45459
C	3.156689	4.517864	-1.89386
H	3.752685	5.171728	-2.52026
C	1.60909	-0.17793	-2.0076
H	1.561001	0.891162	-2.17445
C	1.641178	2.841951	-0.29241
H	1.033987	2.197925	0.336613
C	2.588137	1.079203	4.801775
H	2.333042	1.184084	5.849706
C	2.775675	-0.18299	4.255502
H	2.666733	-1.0649	4.875807
C	0.837749	-2.39184	-2.54
H	0.193645	-3.04937	-3.11198
C	0.785696	-1.02106	-2.74172
H	0.104393	-0.60287	-3.47329
Sb	6.659713	-0.58568	-0.05778
O	4.904866	-1.06671	0.595741
C	7.377973	-2.07744	-2.65821
H	7.282998	-2.96157	-2.03899
C	7.179873	-0.81391	-2.11319
C	6.733278	-3.31932	1.439179
H	5.720311	-3.03762	1.698783
C	8.568673	-4.87073	1.56397

H	8.988481	-5.80241	1.924743
C	7.877671	2.084226	0.869546
H	7.166952	2.490818	0.162097
C	7.933283	0.706674	1.067544
C	7.695988	-2.21522	-4.00315
H	7.838205	-3.20363	-4.42393
C	9.32834	-4.03091	0.761335
H	10.34226	-4.30453	0.493001
C	7.485899	-2.47	0.633822
C	7.300893	0.311694	-2.92314
H	7.113643	1.294568	-2.5102
C	8.708704	2.924163	1.596448
H	8.66166	3.995071	1.43672
C	7.271966	-4.51361	1.901853
H	6.675758	-5.16731	2.528219
C	8.816359	0.18318	2.006415
H	8.862684	-0.88522	2.177991
C	8.788053	-2.83813	0.300542
H	9.39558	-2.19417	-0.32823
C	7.831923	-1.09105	-4.80284
H	8.085401	-1.19859	-5.85089
C	7.6366	0.172068	-4.26149
H	7.737713	1.052087	-4.8858
C	9.590476	2.398223	2.530054
H	10.23493	3.057267	3.099886
C	9.640195	1.028267	2.737801
H	10.32004	0.61224	3.471958

Table 20. Cartesian coordinates (Å) of the optimized (PBE0/def2-TZVPP) structure of **B**.

Sb	4.052522	7.584467	4.503861
O	6.081732	7.587697	4.706445
C	3.91635	8.7147	6.339074
C	4.02486	5.440581	4.437107
C	4.594489	9.938851	6.472751
C	3.15495	8.216934	7.412493
C	5.386879	10.59173	5.373733
H	6.206126	11.17488	5.796896
H	4.759584	11.28746	4.809682
H	5.809851	9.87831	4.669081
C	3.10435	8.945697	8.596398
H	2.528536	8.545218	9.425588
C	3.32386	4.787266	3.407085
C	2.372026	6.933148	7.374833
H	1.321876	7.124921	7.142132

H	2.407695	6.446492	8.350947
H	2.723452	6.224577	6.627966
C	2.537155	5.505469	2.350224
H	1.673015	6.006452	2.786388
H	2.198393	4.802569	1.589262
H	3.124351	6.278724	1.85039
C	3.766961	10.15468	8.75289
C	4.500671	10.63112	7.678747
H	5.025713	11.57668	7.773086
C	3.353558	3.39781	3.357186
H	2.819576	2.899045	2.554385
C	4.059315	1.138951	4.200456
H	3.08757	0.74071	3.903152
H	4.339817	0.683573	5.150783
H	4.785794	0.812042	3.451054
C	4.031113	2.633836	4.297166
C	3.703755	10.90377	10.04888
H	4.120423	11.9063	9.950241
H	4.266942	10.38377	10.82836
H	2.674852	10.99566	10.40268
H	6.316647	7.817366	5.606194
C	4.715999	4.692617	5.399451
C	5.4999	5.285247	6.535033
H	6.511407	5.535109	6.210129
H	5.57143	4.567798	7.353565
H	5.05294	6.195448	6.936003
C	4.692291	3.301518	5.314094
H	5.214536	2.727646	6.073315
O	2.022478	7.553981	4.403861
C	4.255019	8.612842	2.635145
C	3.4872	9.755138	2.365771
C	5.176836	8.145619	1.682118
C	2.445052	10.31402	3.291735
H	1.509677	9.760996	3.188575
H	2.251193	11.35985	3.050841
H	2.736221	10.26623	4.341906
C	5.309257	8.840549	0.483781
H	6.019965	8.47238	-0.24952
C	6.037518	6.931839	1.87459
H	6.82836	7.128027	2.598533
H	6.486146	6.636329	0.926143
H	5.475125	6.078271	2.257355
C	4.576259	9.983423	0.201639
C	3.675184	10.4229	1.158116
H	3.09043	11.31705	0.965162

C	4.735083	10.70256	-1.10303
H	4.476751	11.75814	-1.0095
H	4.081074	10.27212	-1.86689
H	5.7585	10.63195	-1.47432
H	1.649488	8.260778	4.930876

Table 21. Cartesian coordinates (Å) of the optimized (PBE0/def2-TZVPP) structure of **2a**.

Sb	8.291943	2.375781	15.16397
O	9.84566	2.858241	15.99513
C	6.718716	3.657776	15.95964
C	6.671747	3.672213	17.37002
C	5.661053	4.401301	17.99473
C	4.718022	5.095857	17.26342
C	4.795646	5.10144	15.88536
C	5.797921	4.407174	15.20801
C	7.679468	2.98218	18.26765
C	8.599354	4.003097	18.92859
C	7.03272	2.047932	19.2825
C	5.825518	4.557672	13.69883
C	4.586365	3.959557	13.02984
C	5.947622	6.033836	13.31042
C	8.84705	2.517337	13.05389
C	9.598629	3.661757	12.70599
C	10.24809	3.672331	11.4744
C	10.18719	2.596206	10.61199
C	9.434346	1.494302	10.95576
C	8.73598	1.438479	12.1615
C	9.780039	4.890523	13.5784
C	9.50209	6.187008	12.81751
C	11.18985	4.948015	14.16417
C	7.880436	0.213743	12.399
C	6.804495	0.088547	11.31955
C	8.712555	-1.06445	12.48166
C	7.698594	0.422947	15.95831
C	8.646424	-0.32981	16.68561
C	8.197131	-1.43712	17.40458
C	6.866426	-1.7984	17.43603
C	5.950973	-1.06287	16.71164
C	6.3422	0.039908	15.95635
C	10.13512	-0.03713	16.77778
C	10.97825	-1.20976	16.27948
C	10.52549	0.321475	18.21401

C	5.252885	0.730606	15.16225
C	4.579231	-0.23662	14.19055
C	4.193329	1.368172	16.05607
H	5.616858	4.424633	19.07737
H	4.068926	5.66921	15.31673
H	8.346202	2.375475	17.65687
H	9.335263	3.487661	19.55055
H	9.142797	4.570742	18.17227
H	8.042781	4.695274	19.56565
H	6.390167	2.580487	19.98737
H	6.432693	1.28094	18.78919
H	7.804857	1.541567	19.86607
H	6.705991	4.039203	13.31108
H	3.683723	4.476548	13.36242
H	4.646912	4.070219	11.94474
H	4.461023	2.900327	13.26173
H	6.770663	6.520057	13.83239
H	6.109107	6.139008	12.23499
H	5.032435	6.577795	13.55478
H	10.82533	4.543005	11.19117
H	9.375505	0.657279	10.27071
H	9.079526	4.836856	14.41741
H	9.5606	7.03181	13.50816
H	10.25169	6.360078	12.04328
H	8.523882	6.19641	12.3375
H	11.42746	4.025341	14.68827
H	11.91998	5.128431	13.36991

Table 22. Cartesian coordinates (Å) of the optimized (PBE0/def2-TZVPP) structure of **2b**.

As	8.141645	2.419801	15.33253
O	9.379692	2.901876	16.29818
C	6.607685	3.557093	15.85653
C	6.514465	3.827172	17.24251
C	5.619091	4.808398	17.66168
C	4.837845	5.512242	16.76664
C	4.893621	5.193409	15.42516
C	5.74893	4.201746	14.94949
C	7.293587	3.110942	18.33262
C	8.298626	4.04523	18.99968
C	6.377051	2.475466	19.37672
C	5.615174	3.833289	13.48565

C	4.197175	3.334508	13.18815
C	5.952788	4.984823	12.54448
C	8.851472	2.621076	13.48795
C	9.841947	3.617611	13.31339
C	10.63806	3.552182	12.17268
C	10.46764	2.573117	11.21498
C	9.448134	1.655457	11.36004
C	8.621178	1.66447	12.481
C	10.1166	4.779631	14.25398
C	10.08853	6.119666	13.5185
C	11.45806	4.623097	14.9688
C	7.475465	0.671876	12.49527
C	6.556348	0.878546	11.28999
C	7.937679	-0.78128	12.53954
C	7.779811	0.546604	15.86209
C	8.905285	-0.1792	16.30808
C	8.69031	-1.29899	17.10671
C	7.416381	-1.70938	17.44814
C	6.321947	-1.04669	16.92524
C	6.478011	0.068606	16.10634
C	10.34381	0.143429	15.94371
C	10.99171	-1.03291	15.21019
C	11.17044	0.528403	17.16532
C	5.237159	0.620786	15.42975
C	4.615648	-0.44832	14.52761
C	4.183149	1.164193	16.38585
H	5.539673	5.028213	18.71987
H	4.247115	5.711438	14.72704
H	7.872043	2.308614	17.88323
H	8.882431	3.484343	19.73487
H	8.986507	4.455888	18.26244
H	7.798444	4.865128	19.522
H	5.797318	3.214793	19.93393
H	5.67712	1.769354	18.92892
H	6.983638	1.92699	20.10157
H	6.312907	3.022147	13.27067
H	3.475014	4.146872	13.29168
H	4.128299	2.958284	12.16457
H	3.88949	2.53741	13.86383
H	6.982987	5.322001	12.66876
H	5.81256	4.672425	11.50686
H	5.296185	5.840272	12.71897
H	11.41877	4.290263	12.03771
H	9.284127	0.914858	10.58655
H	9.347676	4.801	15.02342

H	10.27479	6.924576	14.23401
H	10.86961	6.186088	12.75786
H	9.131692	6.30806	13.0273
H	11.52336	3.65763	15.4654
H	12.2891	4.735323	14.26625
H	11.56376	5.397225	15.73267
H	6.885693	0.853832	13.393
H	6.268623	1.923346	11.16304
H	7.044596	0.554909	10.3684
H	5.64836	0.283378	11.40652
H	7.091	-1.45234	12.37833
H	8.671646	-0.98881	11.75802
H	8.383088	-1.02779	13.50197
H	9.545487	-1.85484	17.47236
H	5.323592	-1.40817	17.14549
H	10.35306	1.001027	15.27355
H	11.13997	-1.88506	15.87716
H	10.3928	-1.37112	14.36432
H	11.97121	-0.73709	14.82683
H	11.25844	-0.30976	17.86198
H	12.17899	0.814124	16.85564
H	10.71797	1.376912	17.67554
H	5.547199	1.446612	14.7894
H	3.803953	-0.01911	13.93578
H	5.345804	-0.88225	13.84448
H	4.194921	-1.26259	15.12024
H	4.558804	2.010878	16.95565
H	3.307078	1.503133	15.82767
H	3.847628	0.39719	17.08616
H	4.169414	6.289451	17.11772
H	11.11553	2.539617	10.34699
H	7.276941	-2.5688	18.09298

Table 23. Cartesian coordinates (Å) of the optimized (PBE0/def2-TZVPP) structure of **2c**.

P	8.151636	2.393286	15.33281
O	9.284308	2.822133	16.19781
C	6.715901	3.471565	15.80395
C	6.619537	3.757374	17.19384
C	5.762423	4.771891	17.60997
C	5.003718	5.50035	16.71608
C	5.042175	5.166553	15.37877
C	5.861122	4.144218	14.90542
C	7.356631	3.019939	18.30281
C	8.391574	3.919049	18.97409

C	6.396588	2.443079	19.344
C	5.687099	3.778615	13.44421
C	4.23756	3.365252	13.16238
C	6.08128	4.906007	12.49537
C	8.774827	2.584453	13.59489
C	9.731906	3.620398	13.40871
C	10.55627	3.565945	12.28978
C	10.45586	2.555469	11.35414
C	9.464975	1.607055	11.49093
C	8.60502	1.611238	12.58624
C	9.932037	4.820309	14.32416
C	9.892061	6.138722	13.54947
C	11.25264	4.745247	15.09098
C	7.477671	0.597032	12.56749
C	6.592039	0.791622	11.33415
C	7.952741	-0.85094	12.62245
C	7.774676	0.64104	15.80762
C	8.895921	-0.11629	16.23217
C	8.682733	-1.22518	17.04418
C	7.412159	-1.61245	17.4216
C	6.318318	-0.93932	16.91286
C	6.471942	0.167087	16.08223
C	10.33695	0.166727	15.83647
C	10.95207	-1.04143	15.12492
C	11.19355	0.558725	17.0356
C	5.215274	0.712737	15.42652
C	4.569246	-0.37112	14.55835
C	4.180639	1.269616	16.39696
H	5.693315	4.998356	18.66728
H	4.407323	5.697557	14.68012
H	7.898269	2.182791	17.8742
H	8.95524	3.340253	19.71144
H	9.093668	4.312797	18.24111
H	7.917133	4.754603	19.49533
H	5.856272	3.216736	19.89353
H	5.659828	1.777239	18.89329
H	6.964835	1.863845	20.07613
H	6.329033	2.925591	13.22704
H	3.568501	4.223657	13.24778
H	4.141799	2.969191	12.14819
H	3.880545	2.605784	13.85642
H	7.127076	5.18899	12.61514
H	5.924112	4.597407	11.45898
H	5.469758	5.794405	12.6688
H	11.30609	4.335028	12.15357

H	9.348847	0.845626	10.72921
H	9.129096	4.842086	15.05826
H	9.995087	6.968419	14.25339
H	10.71978	6.219991	12.84158
H	8.965537	6.275137	12.99045
H	11.35508	3.79559	15.60945
H	12.10029	4.886725	14.41453
H	11.29243	5.542452	15.83784
H	6.860563	0.7727	13.44578
H	6.300081	1.833291	11.19153
H	7.1075	0.465448	10.42853
H	5.685145	0.19086	11.42929
H	7.117142	-1.52909	12.43403
H	8.711945	-1.05108	11.86319
H	8.369983	-1.09505	13.5975
H	9.537765	-1.79141	17.39344
H	5.319876	-1.28561	17.15553
H	10.35413	1.001865	15.13972
H	11.09776	-1.87853	15.81122
H	10.33525	-1.39077	14.29725
H	11.93051	-0.77226	14.71943
H	11.2718	-0.26645	17.74871
H	12.2042	0.810524	16.70406
H	10.77271	1.426607	17.54015
H	5.506487	1.526209	14.76413
H	3.753835	0.054751	13.96933
H	5.282899	-0.82985	13.87378
H	4.147674	-1.16718	15.17446
H	4.561265	2.132211	16.93915
H	3.287788	1.587674	15.85327
H	3.869114	0.515633	17.1221
H	4.365386	6.303697	17.0645
H	11.13117	2.525218	10.50719
H	7.274562	-2.46183	18.08005

Table 24. Cartesian coordinates (Å) of the HAR structure of **3**.

Sb	11.12156	8.106451	13.61406
O	10.48157	6.454877	13.10566
C	12.39684	7.702609	15.31305
C	11.61023	8.693351	10.63688
C	12.35058	8.50065	16.4766
C	13.17253	6.520013	15.27906
C	9.375959	10.71713	13.60118
C	9.381752	9.349785	13.96919

C	12.16609	8.932238	11.92057
C	13.82284	6.132412	16.45528
H	14.41792	5.215604	16.43227
C	13.3544	5.613018	14.06716
H	12.97856	6.143579	13.17005
C	11.61097	9.819947	16.56275
H	11.30021	10.11264	15.52482
C	14.82372	5.246396	13.82182
H	15.45731	6.109065	13.79669
H	14.89842	4.706191	12.8694
H	15.20116	4.583702	14.63172
C	14.0561	10.0431	10.91346
H	15.01256	10.55116	11.00552
C	10.7316	12.72568	14.27765
H	11.68365	13.27046	14.12247
H	10.7045	12.40711	15.31305
H	9.920337	13.44658	14.12121
C	8.185465	8.709762	14.37182
C	10.62298	11.53801	13.30855
H	11.51499	10.90695	13.46669
C	13.00575	8.053328	17.63002
H	12.95753	8.655792	18.53578
C	12.32372	9.143211	9.520618
H	11.91909	8.966921	8.528293
C	13.72682	6.86887	17.62348
H	14.23993	6.530672	18.53325
C	13.99772	10.02263	13.40103
H	13.26052	9.81521	14.20038
C	8.1505	11.39048	13.54967
H	8.141057	12.45262	13.226
C	10.3488	9.656177	17.40707
H	9.729062	8.847139	17.01309
H	9.745578	10.5427	17.38068
H	10.60085	9.430147	18.42684
C	12.55476	4.322313	14.25316
H	12.86911	3.824235	15.17518
H	12.75105	3.620538	13.4403
H	11.44679	4.553249	14.2652
C	15.23962	9.175863	13.69324
H	15.01776	8.110681	13.65566
H	15.63596	9.409676	14.6919
H	16.05576	9.381591	12.97665
C	13.37707	9.64315	12.06519
C	6.991198	9.437591	14.31693
H	6.056428	8.934607	14.59794

C	8.094152	7.27525	14.87242
H	9.119563	6.853644	14.96109
C	10.26123	8.026258	10.38014
H	9.986945	7.448495	11.26627
C	13.5428	9.786448	9.65152
H	14.12145	10.09876	8.767507
C	12.4935	10.94519	17.11127
H	12.8429	10.73828	18.15891
H	11.9526	11.92866	17.12816
H	13.39016	11.06869	16.49434
C	14.35037	11.51382	13.47683
H	14.666	11.80837	14.49132
H	13.49263	12.1601	13.21481
H	15.16562	11.77402	12.80838
C	6.9663	10.75604	13.886
H	6.007256	11.30285	13.81401
C	10.32294	7.018937	9.227143
H	10.43168	7.507878	8.273033
H	9.365254	6.426962	9.17626
H	11.14685	6.297705	9.385071
C	10.67822	12.04015	11.8606
H	9.82499	12.69523	11.6254
H	10.67511	11.21165	11.12016
H	11.58123	12.64566	11.71957
C	7.315149	6.401753	13.89044
H	6.275135	6.718973	13.83744
H	7.31899	5.317284	14.21347
H	7.769728	6.479916	12.91458
C	7.440481	7.196918	16.25977
H	7.920348	7.868748	16.96221
H	7.484884	6.141041	16.6339
H	6.348558	7.456446	16.2205
C	9.16678	9.055743	10.10292
H	8.989566	9.723004	10.97575
H	8.205045	8.574583	9.875532
H	9.468964	9.693566	9.242767
F	6.389627	1.567151	10.34889
N	11.05614	4.360379	11.2498
C	9.919099	2.271972	10.73442
H	10.87729	1.770003	10.68417
C	8.743346	1.57155	10.49964
H	8.743644	0.479637	10.29569
C	9.895722	3.634918	11.0545
C	8.655875	4.283062	11.12502
H	8.636737	5.341308	11.3845

C	7.544467	2.24744	10.58241
C	7.474962	3.594822	10.88897
H	6.486579	4.111171	10.94344
H	11.8764	3.776187	11.40582
H	10.94725	5.160789	11.91487

Table 25. Cartesian coordinates (Å) of the IAM structure **4** (C–H bond lengths were normalized to 1.089 Å).

Sb	8.775948	7.422464	13.15301
Cu	6.919309	10.33837	12.48473
Cl	5.768952	12.09644	12.54987
O	7.90887	8.814726	12.2777
C	7.830573	5.638943	12.39389
C	8.255995	7.492664	15.24445
C	7.49613	6.774847	10.08239
H	8.305923	7.404322	10.44834
C	8.431798	4.261392	14.52248
H	9.119851	5.092766	14.6685
C	11.23769	5.223167	12.31449
H	10.34935	5.200863	12.944
C	6.904789	7.705699	15.57084
C	6.273592	7.661285	9.850363
H	5.44416	7.050896	9.49626
H	6.511139	8.418779	9.104913
H	5.994871	8.146845	10.78442
C	9.232958	7.278071	16.23313
C	10.56638	10.31498	13.03294
H	9.737137	9.969143	13.64831
C	11.29012	9.097061	12.47435
C	6.069471	3.625692	11.64499
H	5.328041	2.873581	11.37941
C	10.82433	7.76498	12.56114
C	7.218911	5.700636	11.13322
C	6.323298	4.687755	10.79098
H	5.810795	4.7323	9.83115
C	10.00273	11.18735	11.90871
H	10.82332	11.61977	11.33812
H	9.398196	11.98542	12.33713
H	9.385	10.57749	11.25113
C	12.81857	6.962575	11.47933
H	13.42389	6.145214	11.09019
C	11.61207	6.679079	12.10553
C	6.762367	3.528024	12.83154
H	6.607599	2.656268	13.46559
C	9.267626	2.971142	14.38287

H	8.605948	2.106538	14.40662
H	9.97773	2.907564	15.20606
H	9.80768	2.988988	13.43739
C	6.575489	7.7467	16.9227
H	5.542263	7.9352	17.21051
C	7.65397	4.515126	13.24121
C	10.6961	6.992148	15.92892
H	10.77713	6.780885	14.86368
C	13.26694	8.267015	11.34025
H	14.20705	8.469656	10.82932
C	12.51597	9.312022	11.85343
H	12.896	10.32902	11.76852
C	7.543543	4.158902	15.76519
H	6.864101	3.3148	15.6567
H	6.968122	5.076695	15.87686
H	8.167102	4.011768	16.64579
C	4.871172	6.577157	14.57441
H	4.469608	6.428101	15.57564
H	5.455024	5.705241	14.28321
H	4.051059	6.716629	13.87164
C	12.34817	4.468914	13.05724
H	13.2561	4.47505	12.45594
H	12.03554	3.440413	13.2315
H	12.54247	4.955871	14.01172
C	11.23914	5.777152	16.68557
H	12.26384	5.582613	16.37241
H	10.62105	4.90773	16.46651
H	11.21834	5.977277	17.75582
C	7.946287	6.141769	8.757815
H	7.167494	5.474681	8.391214
H	8.862189	5.575172	8.919098
H	8.128587	6.925227	8.023728
C	8.843101	7.315408	17.56686
H	9.582348	7.154529	18.35016
C	11.50764	11.16105	13.91599
H	12.03469	10.51045	14.61231
H	10.92353	11.89187	14.47333
H	12.22969	11.67809	13.28574
C	7.521986	7.555028	17.90874
H	7.230668	7.593012	18.95737
C	5.768259	7.819747	14.5642
H	6.209349	7.904854	13.57217
C	10.8877	4.521511	10.99977
H	10.09594	5.071891	10.4937
H	10.54803	3.508143	11.20866

H	11.76945	4.484215	10.36177
C	4.914047	9.073263	14.80891
H	5.558172	9.950617	14.8446
H	4.384759	8.972969	15.75533
H	4.193393	9.185014	14.00015
C	11.54407	8.229109	16.22028
H	11.54198	8.42617	17.2913
H	11.1292	9.086362	15.69214
H	12.56569	8.055736	15.88538

Table 26. Cartesian coordinates (Å) of the optimized (BP86/def2-SVP) structure of **5**.

Sb	2.48505	12.93775	2.827401
Sb	7.444245	8.448203	5.848493
Ag	4.605151	10.36704	4.445411
O	3.11338	11.14801	3.191413
O	5.674375	9.224628	5.84631
C	4.327154	14.15609	2.825946
C	8.671497	9.965289	4.812007
C	1.795387	12.86182	0.724018
C	-0.37663	13.97632	3.813526
C	8.070491	8.485419	7.971134
C	7.179058	6.351774	5.102482
C	6.547922	12.79964	5.875651
H	7.024009	13.74382	5.540017
H	5.831424	13.05506	6.682884
H	5.96473	12.39882	5.017577
C	-1.52115	13.93523	4.640281
H	-2.43627	14.44924	4.308596
C	2.062281	11.97115	6.135223
H	2.92379	12.14179	5.453056
C	5.336161	7.149476	3.410053
H	5.772036	8.143414	3.649737
C	0.79148	13.28988	4.252623
C	7.579483	11.77557	6.39066
H	6.993215	10.9024	6.742559
C	5.643957	16.08341	3.495416
H	5.740724	17.03013	4.049002
C	6.496348	11.36439	1.729748
H	7.520006	11.76385	1.577323
H	6.423863	10.40819	1.172397
H	6.388862	11.13384	2.812255
C	2.03103	13.94742	-0.17331
C	8.516304	11.31126	5.264776
C	6.460819	3.687105	4.545217

H	6.168149	2.645961	4.340304
C	9.293006	12.30246	4.623546
H	9.200698	13.34775	4.95643
C	4.422764	15.37553	3.54962
C	5.418294	13.64417	2.059329
C	10.62871	8.783693	7.404244
H	10.2777	8.468444	6.396979
C	6.726192	15.60832	2.747074
H	7.665693	16.1812	2.709809
C	6.128402	6.091839	4.175452
C	5.407644	12.34214	1.244201
H	4.439637	11.82787	1.413582
C	-0.33615	12.66235	6.311854
H	-0.31972	12.17121	7.296678
C	7.042446	8.32837	8.944577
C	2.406213	15.37952	0.235383
H	2.448697	15.41703	1.346225
C	9.561575	9.623838	3.758258
C	5.792071	4.743353	3.921266
H	4.979887	4.524299	3.211395
C	2.442373	12.57113	7.504354
H	2.571198	13.67019	7.452008
H	3.393997	12.12795	7.863207
H	1.6745	12.35994	8.27658
C	0.83919	12.65564	5.527822
C	6.608702	14.40551	2.04182
H	7.463557	14.03969	1.452527
C	8.335735	12.35287	7.604628
H	8.95514	13.23169	7.328129
H	8.997748	11.60311	8.077578
H	7.610721	12.68774	8.374364
C	1.852754	10.44617	6.23547
H	2.769891	9.950942	6.617598
H	1.613291	10.01125	5.245987
H	1.024724	10.20178	6.933279
C	7.521991	3.963742	5.410768
H	8.074602	3.131273	5.872685
C	10.30288	10.66419	3.155253
H	10.99859	10.42318	2.336658
C	5.297789	6.508184	9.267057
H	6.004624	5.727096	8.922238
H	4.274611	6.18186	8.988858
H	5.345775	6.540881	10.37543
C	5.610199	7.881613	8.637673
H	5.499418	7.783943	7.538087

C	7.918348	5.289068	5.69604
C	3.290518	15.97337	4.385727
H	2.405813	15.30692	4.291292
C	3.861926	7.181076	3.862863
H	3.346036	6.228314	3.621306
H	3.314086	7.997509	3.347623
H	3.782793	7.351092	4.954026
C	1.254252	11.6239	0.273083
C	3.776421	15.84911	-0.29021
H	4.60905	15.24431	0.111367
H	3.953652	16.90409	0.002204
H	3.818775	15.80232	-1.39811
C	-0.47755	14.8147	2.541006
H	0.511574	14.7845	2.039045
C	11.31832	10.15226	7.240587
H	10.6542	10.90678	6.782004
H	12.21184	10.0508	6.591585
H	11.66346	10.54604	8.218927
C	9.413574	8.796692	8.343688
C	-1.5126	13.27333	5.870463
H	-2.41814	13.25176	6.495865
C	0.854854	10.45998	1.184707
H	1.082173	10.74087	2.233995
C	10.17525	11.98977	3.583373
H	10.77054	12.78369	3.106372
C	-0.77443	16.29183	2.881015
H	-1.77675	16.40716	3.341437
H	-0.76055	16.91499	1.964415
H	-0.03129	16.7078	3.589437
C	1.317076	16.37834	-0.22011
H	1.321333	16.49699	-1.32267
H	1.50494	17.38091	0.216173
H	0.299886	16.05592	0.072239
C	7.373711	8.567981	10.2971
H	6.595273	8.45059	11.06672
C	-1.51643	14.25394	1.551257
H	-1.27234	13.21711	1.248286
H	-1.56058	14.8725	0.631574
H	-2.53369	14.25021	1.993824
C	5.447072	6.960525	1.88339
H	6.502768	6.909229	1.551001
H	4.959274	7.807718	1.359226
H	4.942641	6.032675	1.543905
C	2.868108	17.36736	3.877481
H	2.02198	17.75871	4.478449

H	2.551015	17.34195	2.815699
H	3.696365	18.10055	3.961663
C	5.550498	12.58147	-0.27321
H	6.497966	13.1029	-0.52385
H	4.711682	13.17676	-0.68038
H	5.557005	11.60905	-0.8067
C	1.685971	9.196015	0.890182
H	1.51769	8.826735	-0.14291
H	1.403311	8.379355	1.585322
H	2.766939	9.396967	1.020447
C	4.576884	8.940294	9.06974
H	4.578028	9.095505	10.16881
H	3.555008	8.619215	8.782155
H	4.779492	9.912726	8.580265
C	9.669519	9.048189	9.709945
H	10.69313	9.305656	10.02276
C	1.809712	13.71591	-1.5492
H	1.99729	14.5314	-2.26459
C	9.160643	5.464019	6.56543
H	9.353538	6.552502	6.660464
C	11.67224	7.744539	7.877361
H	12.46186	7.609567	7.109871
H	11.21983	6.757519	8.089697
H	12.1755	8.082329	8.805936
C	10.39661	4.841821	5.879466
H	10.29834	3.740703	5.79074
H	11.3142	5.043581	6.467883
H	10.54686	5.247319	4.85958
C	3.659838	16.02508	5.882117
H	4.491531	16.73578	6.06868
H	3.978381	15.03254	6.257437
H	2.793143	16.36233	6.486702
C	8.9707	4.90096	7.986453
H	8.124989	5.389334	8.508623
H	9.882003	5.060268	8.598313
H	8.775924	3.809082	7.965601
C	9.772215	8.203357	3.232768
H	9.133166	7.518719	3.831815
C	1.03632	11.46683	-1.11412
H	0.620034	10.51754	-1.48519
C	-0.65691	10.1637	1.098319
H	-1.26705	11.05866	1.333509
H	-0.93261	9.367813	1.82015
H	-0.95239	9.81126	0.088356
C	8.663525	8.950126	10.67712

H	8.893397	9.149018	11.73517
C	11.23257	7.736428	3.403796
H	11.3492	6.689023	3.057887
H	11.56248	7.787307	4.460744
H	11.93072	8.357575	2.80586
C	1.334851	12.48765	-2.02132
H	1.172447	12.33579	-3.09948
C	9.323902	8.067388	1.763177
H	9.967509	8.667079	1.086683
H	8.279914	8.409865	1.620992
H	9.388906	7.010596	1.432619

Table 27. Cartesian coordinates (Å) of the IAM structure **6**-bent (C–H bond lengths were normalized to 1.089 Å).

Au	5.193869	3.40907	9.526264
Sb	7.250118	1.736031	11.74881
P	3.567757	3.473879	8.015051
O	6.698654	3.351115	10.92929
C	3.12711	0.793651	8.616839
H	3.745797	0.98205	9.492997
C	7.91344	2.241046	13.72095
C	5.406647	0.645538	11.91933
C	9.16617	1.63986	9.32367
C	7.677963	1.38563	14.83132
C	5.057953	3.386153	5.682522
H	5.48452	2.484126	6.118767
C	2.571146	-0.46491	8.423535
H	2.751584	-1.26016	9.145289
C	8.166911	3.538636	8.004112
H	8.256062	4.155671	8.896995
H	7.284295	3.839199	7.441466
H	9.053417	3.666871	7.38477
C	2.898919	1.820771	7.694976
C	9.016218	1.157669	10.63439
C	8.037436	2.064912	8.411847
H	7.101743	1.947418	8.956452
C	4.080524	4.090402	6.379464
C	5.35805	-0.71301	11.55235
C	2.510161	5.672272	9.307337
H	3.544709	5.88184	9.57513
C	7.981743	1.160958	7.174855
H	8.912129	1.253504	6.616521

H	7.147515	1.460919	6.542393
H	7.847822	0.126293	7.487034
C	3.537955	5.247387	5.811691
H	2.778265	5.814253	6.34784
C	4.290133	1.324406	12.45444
C	8.617195	3.44827	13.85701
C	8.097246	1.827934	16.08944
H	7.913301	1.205513	16.9639
C	1.786427	-0.70194	7.309868
H	1.350148	-1.68811	7.158057
C	6.549873	-1.43486	10.96061
H	7.443879	-0.88766	11.25599
C	2.202243	4.540381	8.548059
C	4.238554	2.796035	12.87566
H	5.13705	3.278491	12.49374
C	10.45512	1.656733	8.770133
H	10.60123	2.059309	7.768881
C	10.09992	0.56662	11.32129
C	2.110959	1.563122	6.566773
H	1.937663	2.3505	5.834705
C	1.549585	0.309222	6.378265
H	0.925887	0.114573	5.50704
C	1.504061	6.527874	9.719867
H	1.750335	7.41995	10.29386
C	11.53785	1.173198	9.469772
H	12.53928	1.257071	9.050233
C	3.037781	-0.74644	12.23506
H	2.101957	-1.2901	12.35583
C	0.870889	4.277036	8.217345
H	0.618674	3.406015	7.614328
C	4.14768	-1.39021	11.70535
H	4.072359	-2.43469	11.40649
C	11.35449	0.58367	10.69987
H	12.20324	0.120657	11.20102
C	3.112371	0.572836	12.60533
H	2.23168	1.052426	13.02995
C	7.959424	-1.06226	15.42367
H	7.962331	-0.90503	16.50125
H	8.974721	-0.97094	15.04058
H	7.576825	-2.05775	15.20336
C	3.96992	5.671694	4.560463
H	3.546547	6.571458	4.116502
C	9.053275	4.330894	12.68535
H	8.499026	4.005881	11.80609
C	-0.12848	5.13078	8.661194

H	-1.1693	4.917366	8.422304
C	5.494657	3.819387	4.439686
H	6.271205	3.268757	3.910819
C	9.994148	-0.1629	12.65014
H	8.971035	-0.06213	13.00931
C	7.061691	-0.00849	14.754
H	6.96694	-0.27241	13.70172
C	0.189172	6.253375	9.405637
H	-0.60107	6.921526	9.744778
C	4.937844	4.954992	3.87551
H	5.262863	5.286032	2.89027
C	9.002148	3.8326	15.146
H	9.520488	4.779918	15.28683
C	3.03371	3.513556	12.25904
H	2.98615	3.292564	11.19376
H	3.137873	4.588147	12.4016
H	2.119702	3.170037	12.74122
C	4.213249	3.00237	14.39137
H	3.404458	2.415989	14.82488
H	4.055199	4.057157	14.61129
H	5.163314	2.682285	14.81663
C	10.28131	-1.65847	12.44185
H	11.31625	-1.78977	12.12949
H	9.618797	-2.05197	11.67234
H	10.11345	-2.19484	13.37462
C	10.9311	0.430496	13.71106
H	10.81079	-0.1154	14.64564
H	10.68315	1.479087	13.86885
H	11.96266	0.347002	13.3722
C	10.55354	4.127069	12.3894
H	11.13712	4.401679	13.26687
H	10.84618	4.753369	11.54796
H	10.73657	3.08177	12.14492
C	8.735712	5.79853	12.90938
H	7.684858	5.906902	13.17373
H	8.940267	6.357143	11.99722
H	9.355495	6.184726	13.71724
C	8.738363	3.03783	16.24349
H	9.040489	3.367665	17.23638
C	5.668316	-0.0824	15.38186
H	5.717065	0.256575	16.41561
H	5.311535	-1.11094	15.35448
H	4.983431	0.553956	14.82338
C	6.478552	-1.40667	9.443399
H	5.601987	-1.96089	9.11113

H	7.376199	-1.86278	9.028526
H	6.405173	-0.37477	9.103246
C	6.698298	-2.86867	11.47533
H	6.7861	-2.85714	12.56073
H	7.590425	-3.31915	11.04278
H	5.822679	-3.44889	11.188

Table 28. Cartesian coordinates (Å) of the IAM structure **6**-linear (C–H bond lengths were normalized to 1.089 Å).

Au	7.27995	12.60924	27.84537
Sb	7.27995	12.60924	31.67518
P	7.27995	12.60924	25.64069
O	7.27995	12.60924	29.80524
C	5.639049	11.31554	32.18663
C	5.153477	12.3911	23.80502
H	5.562019	13.2697	23.30795
C	5.767176	11.90313	24.93562
C	5.189148	10.7809	25.54544
H	5.649882	10.37604	26.44529
C	4.215091	12.39236	30.29042
H	5.039498	13.10357	30.26895
C	4.496825	11.34706	31.3587
C	4.024356	11.77956	23.28868
H	3.564659	12.17666	22.38485
C	5.806488	10.40767	33.21485
C	4.109532	11.72281	28.91945
H	4.947899	11.04154	28.78189
H	4.127724	12.48434	28.1412
H	3.175768	11.16561	28.86002
C	3.552616	10.35219	31.57236
H	2.644312	10.32399	30.97227
C	6.891929	10.44928	34.26534
H	7.488693	11.33924	34.07099
C	4.068036	10.17818	25.0469
H	3.644489	9.311394	25.55209
C	4.814959	9.416583	33.34839
H	4.91929	8.661455	34.12609
C	3.771014	9.386321	32.56053
H	3.047087	8.580996	32.67586
C	3.476904	10.67499	23.89849
H	2.589066	10.19805	23.48594
C	7.841234	9.27662	34.2965
H	7.276416	8.349587	34.20983
H	8.389417	9.279155	35.23746

H	8.542841	9.353763	33.46721
C	2.895964	13.16153	30.56639
H	2.062646	12.46067	30.58385
H	2.733194	13.90113	29.78382
H	2.964283	13.66362	31.53032
C	6.246925	10.63085	35.66301
H	5.41984	11.33596	35.59465
H	6.991913	11.01367	36.35898
H	5.877434	9.671	36.0209
C	8.532101	10.87673	23.80502
H	7.56694	10.79124	23.30795
C	8.647853	11.6522	24.93562
C	9.90874	11.71273	25.54544
H	10.02899	12.31416	26.44529
C	9.626278	10.20466	23.28868
H	9.512222	9.607998	22.38485
C	10.99127	11.04318	25.0469
H	11.9537	11.10977	25.55209
C	10.85659	10.28284	23.89849
H	11.71355	9.752414	23.48594
C	8.154272	14.55989	23.80502
H	8.710891	13.76678	23.30795
C	7.424821	14.2724	24.93562
C	6.741962	15.3341	25.54544
H	6.160977	15.13752	26.44529
C	8.189216	15.84351	23.28868
H	8.762969	16.04307	22.38485
C	6.780545	16.60637	25.0469
H	6.241659	17.40657	25.55209
C	7.506356	16.86991	23.89849
H	7.537239	17.87726	23.48594
C	9.220785	11.83504	32.18663
C	9.000202	10.06344	30.29042
H	7.972077	10.42179	30.26895
C	9.764597	10.83008	31.3587
C	9.9233	12.43398	33.21485
C	9.63283	10.3068	28.91945
H	9.80365	11.37348	28.78189
H	8.964237	9.94179	28.1412
H	10.58226	9.776733	28.86002
C	11.09828	10.5098	31.57236
H	11.57686	9.737292	30.97227
C	9.344544	13.35319	34.26534
H	8.275436	13.42502	34.07099
C	11.27737	12.07083	33.34839

H	11.87916	12.53875	34.12609
C	11.82555	11.18188	32.56053
H	12.88495	10.9576	32.67586
C	9.885444	14.76164	34.2965
H	10.97069	14.73601	34.20983
H	9.609158	15.23511	35.23746
H	9.467834	15.33068	33.46721
C	8.99365	8.536458	30.56639
H	10.01727	8.16521	30.58385
H	8.434521	8.025693	29.78382
H	8.52467	8.34458	31.53032
C	9.509799	12.70381	35.66301
H	9.3127	11.63498	35.59465
H	8.80578	13.15759	36.35898
H	10.5258	12.86375	36.0209
C	6.980016	14.67716	32.18663
C	8.624557	15.37193	30.29042
H	8.828275	14.30237	30.26895
C	7.578428	15.65059	31.3587
C	6.110062	14.98609	33.21485
C	8.097488	15.79812	28.91945
H	7.088301	15.41271	28.78189
H	8.747889	15.40161	28.1412
H	8.081819	16.88539	28.86002
C	7.188951	16.96574	31.57236
H	7.618679	17.76645	30.97227
C	5.603378	14.02526	34.26534
H	6.075721	13.06347	34.07099
C	5.747521	16.34032	33.34839
H	5.041395	16.62753	34.12609
C	6.243285	17.25953	32.56053
H	5.907817	18.28913	32.67586
C	4.113172	13.78947	34.2965
H	3.592747	14.74213	34.20983
H	3.841275	13.31346	35.23746
H	3.829176	13.14329	33.46721
C	9.950236	16.12974	30.56639
H	9.759938	17.20185	30.58385
H	10.67214	15.90091	29.78382
H	10.3509	15.81953	31.53032
C	6.083126	14.49306	35.66301
H	7.10731	14.85679	35.59465
H	6.042157	13.65648	36.35898
H	5.436615	15.29298	36.0209

Table 29. Cartesian coordinates (Å) of the HAR structure of **7a**.

Sb	5.436782	10.44764	15.34091
O	6.371831	12.09034	15.05337
C	6.056114	9.277732	13.67497
C	3.399892	11.08595	15.68205
C	6.505083	9.637544	17.00585
C	6.731916	8.241207	17.02572
C	5.866863	7.21743	16.3029
H	5.082598	7.765408	15.723
C	8.554194	9.897169	14.20289
H	8.110474	10.37961	15.06863
C	5.112361	8.559185	12.91964
C	5.15394	6.332982	17.34367
H	5.863215	5.666503	17.8206
H	4.647418	6.948469	18.12571
H	4.424233	5.657526	16.86715
C	7.444399	9.23087	13.39095
C	2.629	10.4649	16.69031
C	6.825318	11.98153	18.06248
H	5.946573	12.2074	17.43922
C	7.189405	10.51535	17.87219
C	7.761275	7.756071	17.84409
H	7.98529	6.693512	17.88484
C	2.899906	12.19896	14.96666
C	3.032004	9.215429	17.4342
H	4.017067	8.867826	17.07992
C	6.640945	6.322029	15.33138
H	7.114156	6.891014	14.52666
H	7.422191	5.783209	15.87555
H	5.937638	5.600071	14.84983
C	9.560134	8.859208	14.72759
H	9.056752	8.049092	15.30348
H	10.12089	8.381254	13.92849
H	10.30652	9.35799	15.33961
C	5.561048	7.824658	11.81724
H	4.850858	7.262676	11.19458
C	8.225829	9.982275	18.65262
H	8.777863	10.64714	19.30197
C	7.971337	12.91787	17.67447
H	8.820176	12.79093	18.33045
H	8.288127	12.75143	16.65638
H	7.658166	13.92746	17.72024
C	6.910695	7.784081	11.5015
H	7.234843	7.187266	10.64459

C	1.386544	11.02185	17.01508
H	0.793126	10.56096	17.79652
C	3.586575	12.87603	13.79842
H	4.417975	12.21997	13.40459
C	9.284659	10.97661	13.39636
H	9.778147	10.52684	12.52541
H	8.567527	11.77469	13.0473
H	10.04232	11.44433	14.02685
C	3.099957	7.117782	13.41563
H	3.179617	6.575011	12.48727
H	3.686676	6.535511	14.18141
H	2.030201	7.113652	13.67558
C	3.627484	8.541589	13.241
H	3.469931	9.065328	14.20751
C	3.175074	9.486725	18.93243
H	3.879018	10.28804	19.09723
H	2.192077	9.814039	19.36821
H	3.499936	8.573369	19.45252
C	7.835998	8.468693	12.28453
H	8.888749	8.418959	12.04768
C	0.918446	12.1609	16.38279
H	-0.02357	12.62036	16.67244
C	8.52743	8.625438	18.61207
H	9.322131	8.214275	19.21566
C	4.153412	14.23592	14.22597
H	3.345505	14.85931	14.56279
H	4.865575	14.1483	15.05859
H	4.628191	14.71388	13.33033
C	2.627298	13.05738	12.61252
H	2.105261	12.14636	12.37888
H	1.851517	13.82691	12.83854
H	3.166625	13.36368	11.71848
C	6.413969	12.22931	19.52217
H	5.571768	11.6131	19.8319
H	7.252852	12.03144	20.1932
H	6.170432	13.26852	19.67332
C	1.668059	12.72647	15.36169
H	1.278948	13.61684	14.85385
C	2.036935	8.078179	17.17968
H	1.039076	8.350731	17.53557
H	1.945702	7.839022	16.09836
H	2.325081	7.178289	17.69415
C	2.85079	9.311307	12.17293
H	3.249287	10.32216	12.0256
H	2.894662	8.743939	11.22068

H	1.794919	9.404672	12.45716
H	6.413521	12.34924	14.11919
S	6.073595	12.86663	11.16598
O	6.64135	12.99651	12.51457
O	5.226195	11.69928	11.03139
O	5.48013	14.10503	10.70099
C	9.682463	12.06861	8.488171
H	10.52632	11.8519	7.830387
C	8.093197	11.32152	10.14839
H	7.697291	10.56275	10.79313
C	7.482371	12.56847	10.12069
C	7.959776	13.56693	9.286867
H	7.438619	14.51997	9.285662
C	9.053164	13.30569	8.459267
H	9.399091	14.08367	7.782212
C	9.201906	11.07661	9.340662
H	9.697493	10.10131	9.349895

Table 30. Cartesian coordinates (Å) of the HAR structure of **7b**.

As	13.90984	2.242624	8.083231
O	14.37104	3.390316	6.859078
C	15.71124	1.4714	10.28302
C	13.35509	-0.55339	7.476915
C	15.56958	1.633195	8.874701
C	13.12194	0.778494	7.037154
C	12.13936	2.385895	5.208989
H	12.15831	3.042761	5.961782
C	13.03269	3.411771	9.353303
C	16.63527	1.377605	7.973949
C	13.73134	4.60765	9.688883
C	17.87721	1.049325	8.525296
H	18.61173	0.87536	7.947551
C	18.05612	0.973117	9.886381
H	18.91752	0.780252	10.23829
C	16.98859	1.174775	10.75537
H	17.13317	1.10899	11.69179
C	15.13131	5.020345	9.205015
H	15.48459	4.288749	8.62151
C	12.5981	1.039945	5.75046
C	13.71803	-0.95319	8.90054
H	13.79609	-0.12237	9.45085
C	15.06007	6.298294	8.37059

H	14.65376	7.013312	8.902318
H	14.51687	6.135901	7.570973
H	15.96466	6.563509	8.102142
C	12.4546	-0.05276	4.884764
H	12.14145	0.09737	4.001112
C	14.66704	2.589898	12.34522
H	15.54332	2.577083	12.78559
H	13.9629	2.47745	13.01668
H	14.54647	3.445514	11.88382
C	13.16206	-1.58864	6.573365
H	13.31372	-2.48365	6.853071
C	11.21512	3.992124	10.80474
H	10.37739	3.79083	11.20505
C	14.59089	1.439744	11.32317
H	13.7167	1.508378	10.84281
C	13.0724	2.906454	4.10465
H	13.98896	2.952402	4.445563
H	12.78133	3.800432	3.826228
H	13.03909	2.299135	3.335563
C	11.78583	3.084663	9.911068
C	13.07629	5.475248	10.56281
H	13.48958	6.300522	10.78623
C	16.85123	-0.04221	5.921624
H	16.66153	-0.07697	4.961311
H	16.31198	-0.71706	6.385809
H	17.802	-0.22315	6.074421
C	12.58725	-1.81609	9.481511
H	12.57421	-2.68502	9.028403
H	11.72878	-1.3637	9.345271
H	12.73844	-1.94933	10.4401
C	11.06507	1.79499	9.563802
H	11.75288	1.156391	9.217754
C	12.7547	-1.34478	5.279758
H	12.68025	-2.06158	4.660967
C	16.50201	1.350639	6.463096
H	15.54532	1.535707	6.237258
C	14.60563	0.118415	12.10329
H	14.69517	-0.6303	11.47706
H	13.77023	0.025383	12.60651
H	15.36363	0.114441	12.7259
C	11.8452	5.172761	11.1158
H	11.43288	5.785602	11.71315
C	15.06212	-1.70354	8.956498
H	15.20353	-2.05384	9.861134
H	15.79036	-1.08709	8.728833

H	15.05074	-2.44517	8.31692
C	16.11839	5.217314	10.37025
H	17.00267	5.443405	10.01498
H	16.17704	4.389871	10.89215
H	15.80178	5.94463	10.94648
C	10.05301	2.020097	8.438068
H	9.267953	2.488014	8.793711
H	9.77654	1.154879	8.069028
H	10.46314	2.560599	7.731966
C	10.69538	2.315549	4.679038
H	10.67926	1.805976	3.841123
H	10.36251	3.222225	4.515642
H	10.12552	1.87189	5.341164
C	10.40715	1.13843	10.7636
H	11.06096	1.049395	11.48716
H	10.07725	0.250361	10.50996
H	9.656092	1.690316	11.06694
C	17.35481	2.437482	5.808063
H	18.30209	2.253035	5.973451
H	17.11983	3.309899	6.188739
H	17.18629	2.446803	4.84273
H	13.75611	3.962813	6.747821
S	11.85298	6.237445	5.960959
O	12.28163	5.128209	6.820072
O	12.94794	7.148306	5.668005
O	11.12198	5.81408	4.786838
C	10.58013	6.925544	8.309695
H	11.08497	6.230598	8.714794
C	9.707064	7.702865	9.084871
H	9.624875	7.544224	10.01852
C	10.7052	7.172927	6.955193
C	8.968082	8.700603	8.482505
H	8.369958	9.224761	9.003206
C	9.964991	8.189424	6.35941
H	10.06044	8.366554	5.431189
C	9.090905	8.943296	7.126357
H	8.575296	9.629027	6.718674

Table 31. Cartesian coordinates (Å) of the optimized (PBE0/def2-TZVPP) structure of **8**.

Sb	3.237158	6.68854	13.73723
O	3.087019	8.571155	13.41437
O	1.568167	7.033607	15.31768
O	0.815815	9.004457	14.59458
C	1.460741	5.814863	12.83952

C	4.717042	6.384331	12.13201
C	4.404008	6.381961	15.57444
C	-1.00881	4.962954	11.90528
H	-1.97916	4.639678	11.54726
C	4.615115	5.34084	11.18369
C	0.838304	4.711379	13.44157
C	0.877716	6.48183	11.74652
C	-0.39976	4.303914	12.95664
H	-0.89652	3.456527	13.4145
C	0.583971	8.910367	11.18391
H	-0.38864	8.777663	10.7028
H	0.413502	9.131729	12.23694
H	1.064701	9.776693	10.72421
C	1.461863	7.67161	10.9981
H	2.439283	7.911449	11.41034
C	5.713221	7.37036	11.97671
C	3.756002	4.091491	11.32674
H	3.214555	4.149539	12.27165
C	-0.36876	6.028279	11.30861
H	-0.84408	6.535904	10.47782
C	3.732828	8.730299	16.5309
H	3.163593	8.810267	15.61495
C	5.325362	4.119556	14.69872
H	4.62178	4.303676	13.88234
C	5.42539	5.385399	10.05061
H	5.33797	4.599468	9.309489
C	5.838023	9.850968	12.43611
H	6.024774	10.59726	13.21181
H	6.533258	10.04731	11.61649
H	4.820268	9.988488	12.07383
C	6.028153	8.445892	13.00096
H	5.341265	8.349408	13.83999
C	5.185144	7.148772	17.7188
H	5.203099	7.910685	18.48825
C	5.912221	5.991875	17.89777
H	6.48592	5.837057	18.80389
C	5.911838	5.04332	16.89986
H	6.500321	4.142417	17.02141
C	6.349885	6.392108	9.858745
H	6.963482	6.408853	8.965644
C	5.17575	5.217528	15.72896
C	4.627099	2.83343	11.37009
H	4.009378	1.955603	11.57452
H	5.120092	2.666645	10.41043
H	5.40251	2.893059	12.13055

C	4.424294	7.380703	16.57449
C	5.008956	2.739948	15.27424
H	4.053952	2.729942	15.79947
H	4.971708	1.996029	14.47617
H	5.77792	2.420429	15.97984
C	1.637807	2.444492	14.15299
H	2.095607	1.871275	14.96057
H	0.677508	1.977897	13.92542
H	2.267567	2.34968	13.26751
C	-0.55081	7.755518	16.08436
H	-1.18105	7.031513	15.56192
H	-0.31077	7.337098	17.0615
H	-1.09974	8.687884	16.19356
C	0.699331	7.969685	15.2679
C	6.50841	7.356302	10.83264
H	7.26582	8.120873	10.70621
C	6.737543	4.138249	14.11156
H	6.900968	3.284145	13.45196
H	6.919847	5.047805	13.53934
H	7.48089	4.082383	14.90919
C	1.663413	7.39455	9.508476
H	2.409498	6.620304	9.331948
H	0.735354	7.09197	9.017798
H	2.011751	8.305467	9.016807
C	4.748966	9.872328	16.51256
H	5.339544	9.906115	17.43116
H	5.440971	9.784444	15.67391
H	4.227134	10.82699	16.41678
C	0.615672	3.973623	15.85102
H	0.528953	5.001334	16.19695
H	-0.38609	3.569111	15.68823
H	1.08758	3.380188	16.63773
C	1.443082	3.902832	14.57055
H	2.431386	4.320351	14.80331
C	7.439453	8.27171	13.56215
H	8.197907	8.378564	12.78353
H	7.636779	9.03112	14.32244
H	7.569399	7.292386	14.02562
C	2.760651	8.904712	17.69619
H	2.069349	8.064806	17.75866
H	3.283926	8.984291	18.65212
H	2.180497	9.819791	17.56061
C	2.716596	3.919781	10.22273
H	3.191046	3.881016	9.239482
H	2.17734	2.980457	10.36503

H	1.98543	4.723338	10.21919
H	2.215064	8.865027	13.81248
